# Site-specific protein functionalization by late-stage reversible cysteine masking

**DOI:** 10.1038/s41467-026-77718-3

**Published:** 2026-09-10

**Authors:** Manjeet Singh, Raj V. Nithun, Omer Harel, Muhammad Jbara

**Affiliations:** School of Chemistry, Raymond and Beverly Sackler Faculty of Exact Sciences, Tel Aviv University, Tel Aviv, Israel

**Keywords:** Chemical synthesis, Peptides, Organic chemistry, Chemical modification

## Abstract

Site-selective functionalization strategies are essential for generating complex proteins for basic research and biomedical applications. However, achieving precise transformations across protein sequences remains a significant challenge and often requires protein engineering with orthogonal handles or sequence interventions to achieve selectivity. Here, we report a modular strategy for site-selective protein functionalization that enables the selective targeting of desired Cys residues through late-stage reversible thiol caging. This strategy involves splitting the protein sequence into two segments and transiently masking native Cys residues to enable chemoselective ligation, thereby restoring the full-length protein with a single reactive Cys for late-stage functionalization. A subsequent decaging step regenerates the native Cys residues, yielding the desired site-specifically modified protein while preserving the native sequence. We demonstrate the versatility of this strategy through multiple transformations, including Cys arylation, alkylation, and elimination, and demonstrate the power of this approach by selective modification of 30 examples in high resolution. Furthermore, we apply this approach to edit Cys-rich zinc fingers, leading to the discovery of advanced analogs with enhanced DNA-binding activity. The versatility and modularity of the developed reversible thiol caging approach establish a powerful platform for site-selective protein modification, providing rapid access to structurally diverse proteins for fundamental research and therapeutic development.

## Abstract (web summary)

Site-selective functionalization strategies are essential for generating complex proteins for basic research and biomedical applications, but achieving precise transformations across protein sequences remains challenging. Here, the authors report a modular strategy for site-selective protein functionalization that enables the selective targeting of desired cysteine residues through late-stage reversible thiol caging (ReCag).

## Introduction

**Fig. 1:**
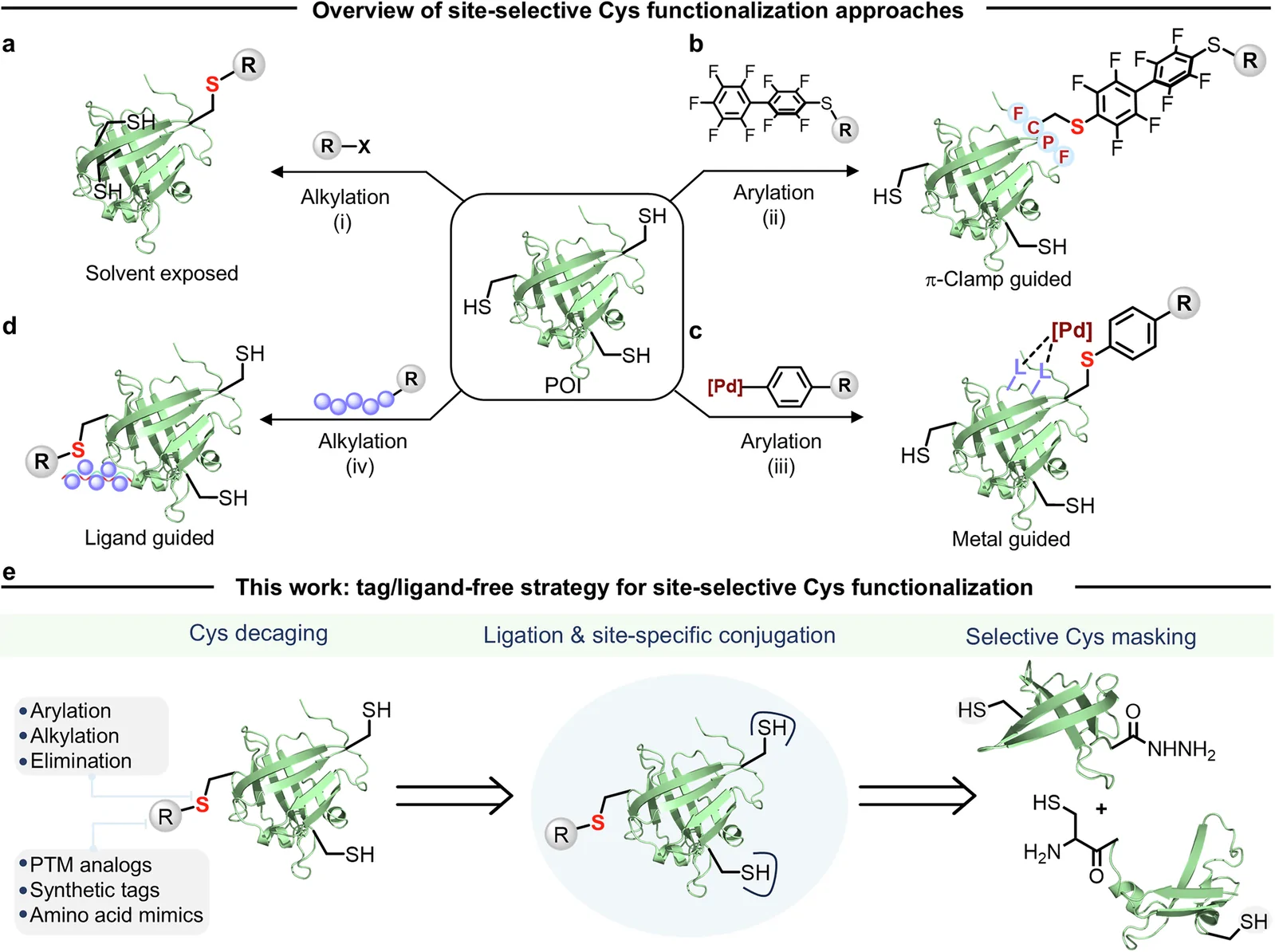
Site-selective Cys conjugation. Overview of the current strategies for selective Cys modification. **a** Modification of solvent-exposed Cys. **b** π-clamp-guided Cys arylation. **c** Metal guided Cys arylation. **d** Ligand-guided Cys alkylation. **e** The site-selective Cys functionalization approach developed in this work. POI: Protein of interest. (PDB; 1ija).

Site-selective protein diversification strategies are essential to produce homogeneously modified analogs to probe their function and design novel therapeutics^1–3^. The low abundance and orthogonal reactivity of the Cys residue within the human proteome have rendered Cys the preferred site for a variety of late-stage transformations^4,5^. Several elegant strategies have been developed for chemoselective protein conjugation through Cys, including alkylation, arylation, and elimination^6–8^. Such transformations have significantly expanded biomolecule modification and editing for diverse biomedical applications^9,10^. Despite these advances, conventional Cys-based bioconjugation strategies remain limited due to their lack of site-specificity^11^. Unfortunately, the current approaches rely on a novel solvent-exposed Cys residue to achieve regioselectivity and often result in mixtures of heterogeneous products (Fig. 1a).

To enhance site selectivity, sophisticated strategies have been developed for the site-selective conjugation of intact proteins. These methods largely rely on pre-engineering the protein sequence with a directing group or recognition element to achieve site-specificity. These methods include Cys arylation using the π-clamp (Phe–Cys–Pro–Phe) motif^12^, which reacts preferentially with perfluoroarenes for site-specific protein arylation (Fig. 1b). The use of metal-guided protein modification has also been demonstrated using a natural metal-binding sequence to direct metal-mediated site-specific Cys arylation (Fig. 1c)^13^. In addition, substrates tethered to peptide ligands have been used to guide the desired transformation to a specific binding domain (Fig. 1d)^14,15^. Although these approaches offer promising opportunities for modifying intact proteins, they largely depend on incorporating an exogenous sequence into the protein of interest to achieve selectivity. Despite these advances, site-selective protein diversification within naturally occurring sequences remains a significant challenge.

The use of protein synthesis employing chemoselective protein ligation technologies has provided a powerful solution for generating novel modified proteins bearing post-translational modifications (PTMs), non-canonical amino acids, and molecular probes for a wide range of applications^16–24^. However, a key limitation of protein synthesis and semisynthesis approaches is that introducing different modifications at the same site typically requires repeating the entire protein synthesis process, which is time-consuming and poses significant challenges for the preparation of multiple protein variants^25–28^. Therefore, the development of orthogonal and modular approaches for the late-stage, site-selective diversification of proteins remains highly desirable, allowing the efficient generation of multiple protein variants from a common intermediate without repeating the entire synthetic process. We therefore set out to develop a general and tag-free approach for site-selective protein functionalization by directing the modification site into the desired domain by temporal masking of the reactive native Cys residues (Fig. 1e). We envisioned that success in controlling the reactivity of specific Cys residues in a late-stage manner would provide sufficient flexibility to insert a broad range of transformations into proteins without sequence intervention or insertion of a directing ligand.

Nature employs novel covalent transformations, e.g., post-translational modifications, to modulate protein structure and function^29,30^. These modifications are dynamic and often employed in a reversible manner to control and alter the chemical properties of specific amino acid residues^17,31^. Inspired by these natural mechanisms, we envisioned that the reactivity of specific Cys residues could be turned on and off on-demand by a controlled masking process to achieve orthogonal transformations at the desired site.

In this work, we introduce a tag-free approach to modify native protein sequences through Cys using a reversible thiol-caging (ReCag) approach. By strategically splitting the protein sequence and temporarily masking the native Cys residues, our approach generates a synthetic intermediate bearing uniquely accessible Cys residue(s). This intermediate can then undergo diverse late-stage functionalization, followed by a decaging step to yield the desired site-specifically modified protein. The versatility of the approach has been demonstrated in the site-selective conjugation of peptides and proteins bearing 3-8 Cys residues with a broad scope of transformations, generating a library of modified proteins bearing synthetic tags, amino acid residues, and PTM mimics. Furthermore, the ReCag approach enables site-specific editing of Cys-rich transcription factors, facilitating the discovery of advanced analogs with enhanced DNA-binding activity. Finally, while our approach selectively diversifies masked proteins rather than intact proteins, the ReCag strategy enables the rapid and efficient generation of proteins with native amino acid sequences while overcoming the inherent limitations of existing methods, which often require sequence modifications or ligand-assisted recognition to achieve site selectivity.

## Results

### Development of site-selective Cys alkylation, arylation, and elimination via a ReCag strategy

**Fig. 2:**
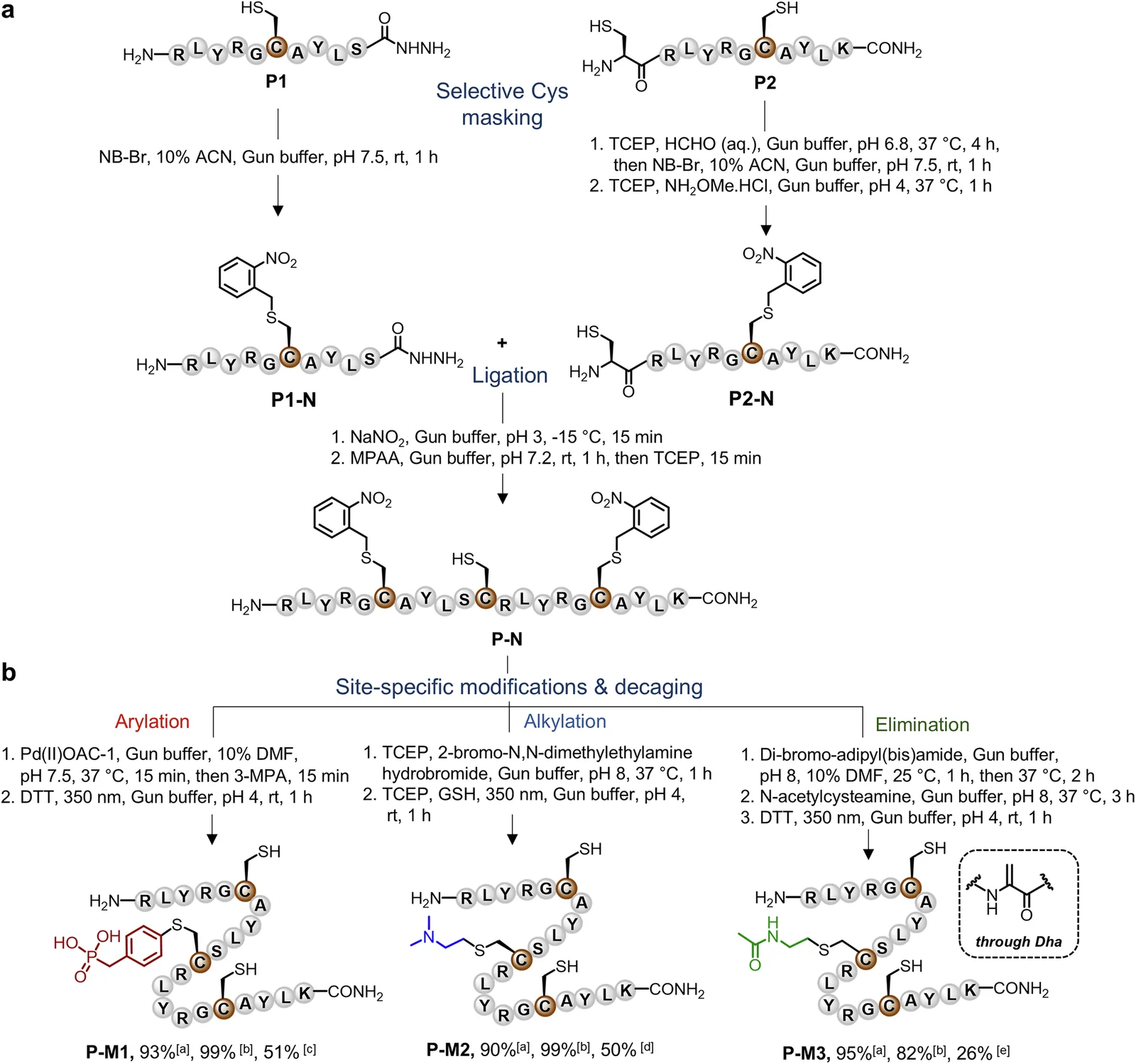
ReCag enables site-selective peptide functionalization. **a** Schematic representation of selective Cys masking and ligation. **b** Schematic representation of Cys alkylation, arylation, and elimination to generate site-specifically modified peptides. Gun buffer: 6 M Gun.HCl, 0.2 M Na_2_HPO_4_. Conversion yield of the modification step^[a]^; conversion yield of the decaging step^[b]^. Conversion yields were determined by LC-MS; isolated yield for the decaging step^[c]^; isolated yield for two steps, Cys-modification, and decaging^[d]^; and isolated yield for three steps: Cys-elimination, modification, and decaging ^[e]^.

Achieving orthogonal Cys transformations requires temporal deactivation of reactive groups to tune selectivity. In this regard, reversible and controlled Cys masking could be applied for temporal deactivation. We envisioned that this could be achieved by dividing the peptide sequence into two segments, followed by late-stage masking of internal Cys residues to allow peptide ligation at the desired modification site. This process would enable reassembling the desired peptide sequence bearing a single reactive Cys residue at the ligation junction, which could then undergo site-selective Cys functionalization, followed by unmasking of internal Cys residues to yield the modified native sequence (Fig. 2). To verify our hypothesis, we synthesized two model peptides, **P1** (RLYRG*C*AYLS) and **P2** (*C*RLYRG*C*AYLK), bearing a total of three Cys residues. We initially set out to site-selectively mask the internal Cys residue within the C-terminal peptide **P2** (Fig. 2a). Importantly, exposing **P2** to formaldehyde allows effective N-terminal Cys protection to form the thiazolidine ring, keeping the internal Cys residue intact (Supplementary section 4.2)^32^. Subsequently, internal Cys was protected in situ with the photoreactive group 2-Nitrobenzyl bromide (NB-Br)^33–35^ to generate masked **P2** in 45% isolated yield for both steps. Finally, the N-terminal thiazolidine was selectively converted to free N-terminal Cys using methoxyamine hydrochloride to provide the desired masked peptide **P2-N** in 70% isolated yield (Supplementary section 4.2). We employed the same caging approach to temporally mask internal Cys in segment **P1** using NB-Br to provide **P1-N** in 63% isolated yield (Supplementary section 4.1). Having both masked segments in hand, we ligated **P1-N** and **P2-N** via a native chemical ligation (NCL) reaction to generate the desired polypeptide **P-N** after 1 h^36,37^. The isolated **P-N** bearing a single free Cys at the ligation junction could be used for site-selective conjugation reactions to provide the desired modified native sequence after unmasking the internal Cys residues (Fig. 2b).

The ReCag approach enables site-selective Cys alkylation, arylation, and elimination to generate site-specifically modified native polypeptides. To explore the scope of transformation that could be employed using the ReCag strategy, we initially examined conventional Cys conjugation approaches, e.g., alkylation using electrophilic alkyl halides (Fig. 2b). We found that treating **P-N** with 2-bromo-N,N-dimethylethylamine hydrobromide reagent^38^, followed by one-pot UV irradiation at 350 nm, enabled the site-specific insertion of the Lys-dimethylation mimic to generate the native modified peptide sequence **P-M2** in 99% conversion yield (Supplementary section 6.3). Importantly, these findings establish the effectiveness and compatibility of the ReCag approach to alkylate a specific Cys residue to generate the native polypeptide sequence.

Having the established conditions for selective Cys alkylation, we explored the methodology to modify peptides with other versatile conjugation reactions. Cys elimination to Dha has emerged as a powerful approach to functionalize proteins with various transformations^8^. However, despite its potential, it is mainly employed for sequences bearing a single Cys residue due to challenges in achieving precise site-selectivity. To explore the compatibility of our approach to site-selectively convert Cys to Dha for further site-selective transformations, we initially set out to convert Cys to Dha using the ReCag approach. We found that reacting **P-N** with di-bromo-adipyl(bis)amide enables effective Cys elimination to Dha (Fig. 2b)^39^. The one-pot Michael addition reaction using the N-acetylcysteamine reagent enabled the site-selective insertion of Lys-acetylation mimic into **P-N**^40^, followed by UV irradiation to provide site-specifically acetylated peptide **P-M3** in 82% conversion yield (Supplementary section 6.4). These findings further highlighted the versatility of the ReCag approach for site-specifically functionalizing peptides bearing multiple Cys residues using Dha chemistry.

Organometallic chemistry has emerged as a powerful approach to insert a broad range of transformations into proteins through Cys arylation^41–43^. Despite the significant potential of transition metals for protein functionalization, this approach has been mainly applied to proteins bearing a single reactive Cys residue owing to challenges in tuning regioselectivity^44–46^. To investigate the compatibility of the ReCag approach with transition metal complexes, we examined the reactivity of **P-N** toward palladium-oxidative addition complexes (Pd(II)OACs)^41^, which have been shown to enable the introduction of a broad range of transformations into biomolecules through C–S cross-coupling^47^. Treating **P-N** with Pd(II)OAC-1 bearing a Tyr-phosphorylation mark^48^ led to effective arylation and the generation of the desired phosphorylated peptide after 15 min (Fig. 2b). Notably, despite the sensitivity of caged-Cys to transition metals^49,50^, the masked internal Cys residues were completely stable (Supplementary section 6.1). Finally, UV irradiation provided the desired arylation product **P-M1** in 99% conversion yield (Supplementary section 6.2). These experiments establish the flexibility and site-selectivity of the ReCag approach to insert broad transformations into peptides without the need for sequence intervention or a directing ligand. In principle, this method could facilitate rapid and efficient peptide diversification, providing unprecedented opportunities to generate novel libraries for a variety of applications.

### ReCag-mediated site-selective protein functionalization

**Fig. 3:**
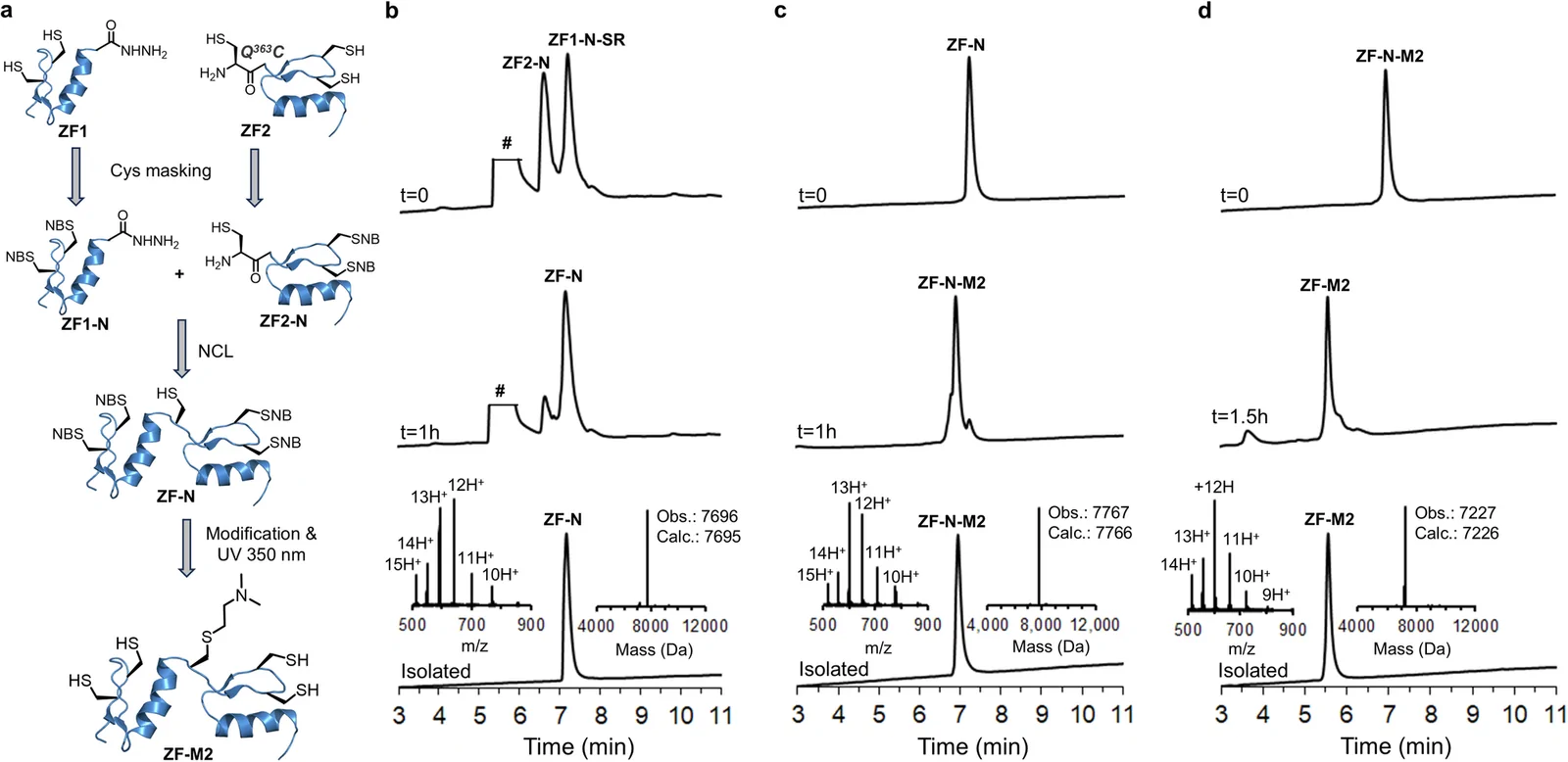
ReCag-mediated site-selective protein functionalization. **a** Schematic representation of the site-specific conjugation of the ZF protein. The Gln residue at position 363 was mutated to Cys for late-stage conjugation at this site. **b** LC-MS analysis of the ligation reaction; Conditions: 1) NaNO_2_, Gun buffer, pH 3, at −15 °C, for 15 min; 2) #: 4-Mercaptophenylacetic acid (MPAA), 6 M Gun.HCl, 0.2 M Na_2_HPO_4_, pH 7.2, at rt, for 1 h, then TCEP, for 15 min. ZF1-N-SR (Thioester of ZF1-N with MPAA). **c** LC-MS analysis of the Cys conjugation reaction; Conditions: DTT, 2-bromo-N,N-dimethylethylamine hydrobromide, 6 M Gun.HCl, 0.2 M Na_2_HPO_4_, pH 8, at 37 °C, for 1 h. **d** LC-MS analysis of the decaging reaction; Conditions: DTT, UV 350 nm, 6 M Gun.HCl, 0.2 M Na_2_HPO_4_, pH 4, at rt, for 1.5 h (PDB; 1ZAA). Source data.

With optimized conditions for site-selective Cys conjugation in hand, we next explored the power of ReCag to functionalize Cys-rich proteins. Zinc fingers (ZFs) constitute a large family of proteins characterized by zinc-stabilized structural motifs, such as the Cys2His2 motif^51^. This motif comprises conserved Cys residues that, together with His residues, coordinate a zinc ion, thereby conferring structural stability and functional specificity. These motifs enable ZFs to interact with DNA, RNA, or other proteins, allowing them to regulate a wide range of cellular events^52^. Given the central role of ZFs in regulating essential physiological processes and their potential as therapeutics, we sought to investigate the ReCag approach to generate site-specifically conjugated ZFs, without sequence intervention. To this end, we set out to generate site-specifically modified two ZF motifs bearing 5 Cys residues, derived from the Zif268 protein^53^. First, we divided the sequence of Zif268 into two segments, **ZF1** and **ZF2** (Supplementary sections 3.5 and 3.6). Next, using the established Cys masking approach, we were able to selectively cage the native Cys residues in both segments to generate **ZF1-N** and **ZF2-N** (Fig. 3a). Having both masked segments in hand, we ligated them using NCL to generate the desired ligation product, **ZF-N**, bearing a single reactive Cys in 57% isolated yield (Fig. 3b). Treating **ZF-N** with 2-bromo-N,N-dimethylethylamine hydrobromide provided the desired site-specifically methylated product after one hour (Fig. 3c). Finally, the photolysis step enabled the effective decaging of all four masked Cys residues, yielding the desired modified product, **ZF-M2**, in 61% isolated yield (Fig. 3d). Taken together, these results further demonstrate the versatility and selectivity of the ReCag strategy to insert desired transformations into Cys-rich proteins.

**Fig. 4:**
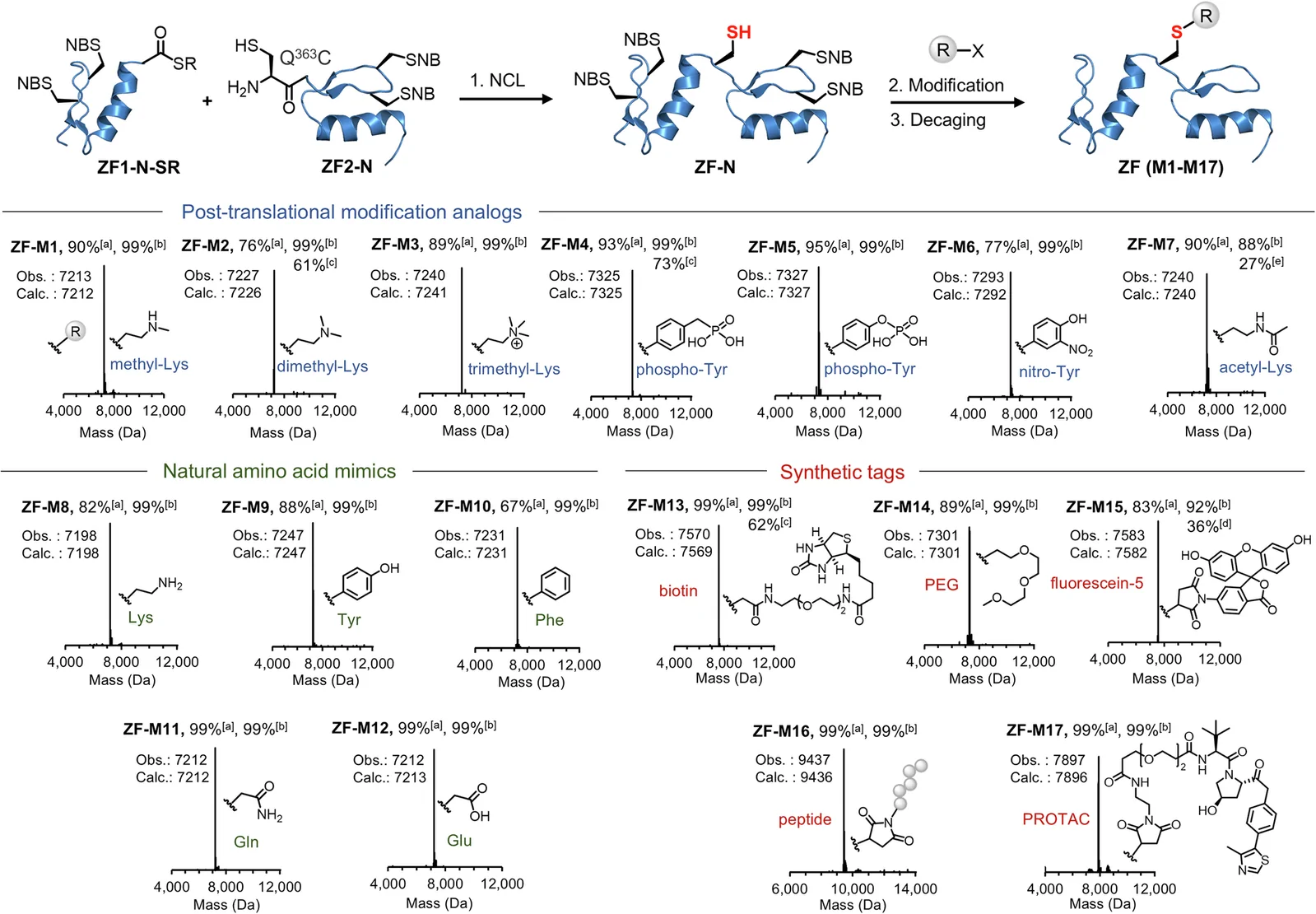
Site-selective protein functionalization with amino acid residues, post-translational modification mimics, and synthetic tags. The figure shows the substrate scope of the conjugation of ZF protein with several molecules and probes **M1–17**. ZF1-N-SR (Thioester of ZF1-N with MPAA). Conversion yield of the modification step^[a]^; conversion yield of the decaging step^[b]^. Conversion yields were determined by LC-MS; the isolated yield for the decaging step^[c]^; isolated yield for two steps: Cys-modification and decaging^[d]^, and isolated yield for three steps: Cys-elimination, modification, and decaging^[e]^. Conditions (Supplementary section 7) (PDB; 1ZAA). Source data.

Encouraged by these results, next, we assessed the scope of transformations that could be introduced into Cys-rich proteins using the ReCag approach. Representative proteinogenic and modified residues bearing PTMs were chosen for late-stage conjugation. For instance, treating **ZF-N** with 2-chloro-N-methylethanamine hydrochloride, 2-bromo-N,N-dimethylethylamine hydrobromide, and (2-bromoethyl)trimethylammonium bromide enabled the generation of site-specifically mono-, di-, and tri-methylated **ZF-M1-3** products (Supplementary section 7), respectively (Fig. 4). Treating **ZF-N** with Pd(II)OACs bearing phospho- and nitro-Tyr mimics enabled the production of site-specifically phosphorylated and nitrated **ZF-M4-6** analogs. On the other hand, treating **ZF-N** with di-bromo-adipyl(bis)amide to generate Dha enabled the site-selective insertion of Lys-acetylation mimic using N-acetylcysteamine to generate **ZF-M7**. Importantly, all post-translational modified analogs were obtained with high homogeneity. These findings further demonstrate the effectiveness of the ReCag approach to site-selectively insert novel PTM mimics into the target proteins in excellent yields (Fig. 4).

In addition to the effective insertion of PTM mimics, ReCag enabled the incorporation of proteinogenic residue mimics, including aromatic, polar, and nonpolar residues such as Lys, Tyr, Phe, Gln, and Glu (**ZF-M8-12**). Finally, the insertion of small molecules and probes commonly used for stabilization, detection, isolation, and other biologically relevant modifications has also been achieved. These include biotin (**M13**), polyethylene glycol (**M14**), fluorescein-5 fluorophore (**M15**), polypeptide (**M16**), and VHL proteolysis targeting chimeras (PROTAC, **M17**). Notably, the ReCag approach was compatible with maleimide chemistry, a versatile and widely used approach for protein modification^54^, further underscoring its broad versatility in introducing diverse modifications into proteins. Importantly, all desired products were obtained as **ZF-M1-17** in excellent yields and homogeneity (Supplementary section 7). Taken together, these results indicate the power and flexibility of the ReCag approach, enabling the incorporation of diverse complex molecules to generate the desired modified proteins in high resolution (Fig. 4).

### Late-stage editing of zinc-fingers reveals potent eZF-TF analogs with enhanced DNA binding

**Fig. 5:**
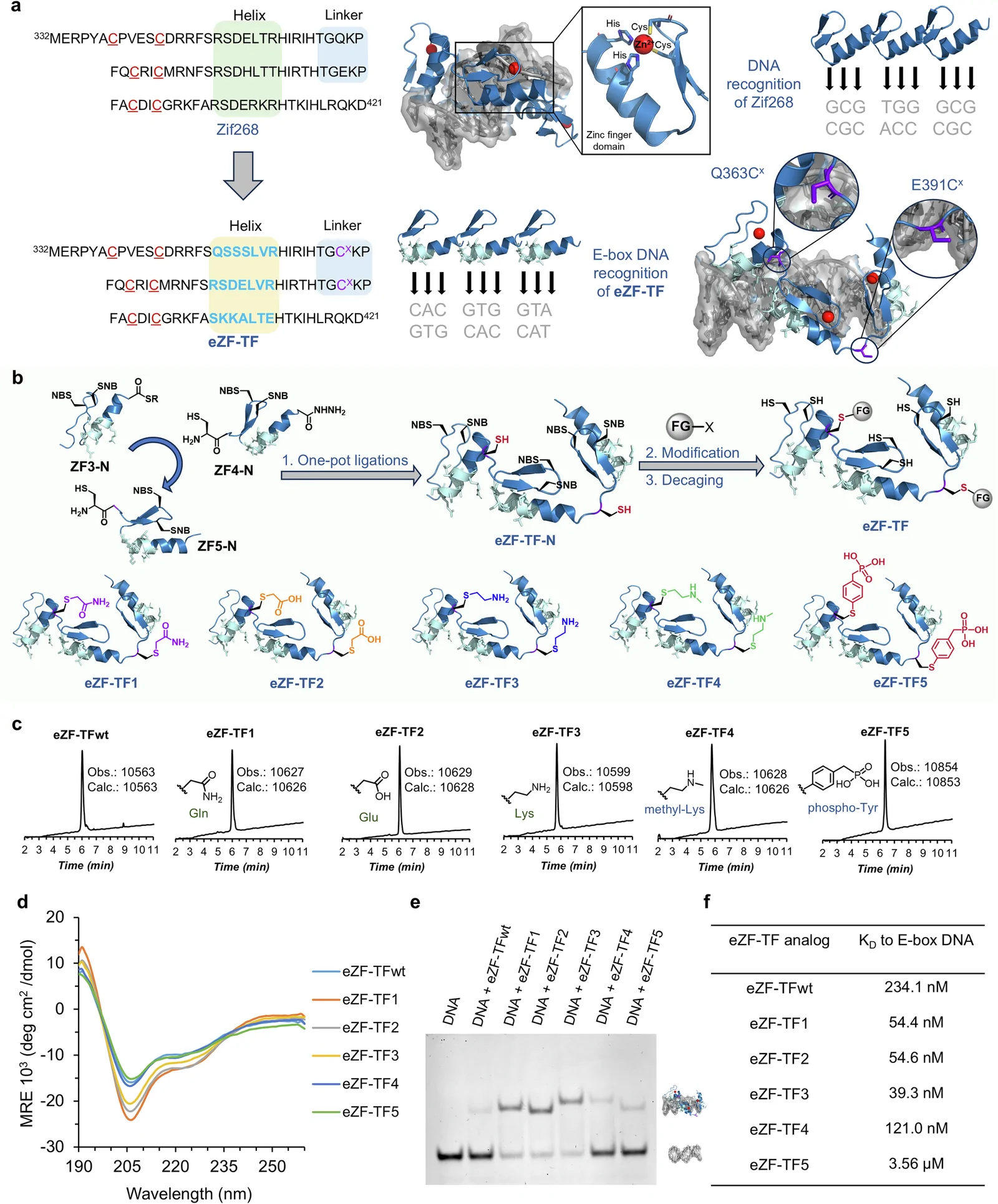
Late-stage editing of zinc fingers reveals potent eZF-TF analogs with enhanced DNA binding activity. **a** Schematic representation of the **eZF-TF** design. **b** Schematic representation of the late-stage editing of **eZF-TF-N** to generate **eZF-TF1-5** analogs; Conditions: Supplementary sections 5 and 8. **c** Analytical LC-MS analysis of the **eZF-TF1-5** and **eZF-TFwt** analogs. **d** CD analysis of the **eZF-TF1-5** and **eZF-TFwt** analogs; Conditions: Supplementary section 10. Experiments were performed in triplicate. **e** EMSA analysis of the **eZF-TFwt** and **eZF-TF1-5** analogs; Conditions: 0.5 μM DNA probe and 2 μM **eZF-TFwt, eZF-TF1-5** analogs in 10 mM Tris-HCl, 50 mM NaCl, 75 µM ZnCl_2_, 1 mM DTT, 5% glycerol, pH 7.3. Experiments were performed in duplicate. **f** Table summarizing the *K*_D_ values obtained from BLI analysis of the binding of **eZF-TF** analogs to the E-box DNA probe. FG: Functional group (PDB; 1ZAA). Source data.

Late-stage protein editing offers a powerful strategy for modulating protein structure and function^55,56^. Of particular interest are the engineered transcription factors (TFs), which have emerged as a powerful tool to regulate gene expression for biomedical applications and drug development^57,58^. Recent studies have shown that targeted modification of strategic sites, e.g., late-stage Cys editing of bHLHLz-TFs, can profoundly influence DNA-binding, cell-permeability, and overall activity^59–61^. Despite these advances, extending these novel transformations to ZF-TFs to generate advanced modified analogs remains highly challenging due to the essential role of native Cys residues in their folding and function^53^. We envisioned that the ReCag approach could be leveraged to enable a controlled diversification of ZF-TFs without altering the native Cys sites. To this end, we set out to engineer ZF-TF (**eZF-TF**) analogs derived from three ZF motifs of the Zif268 protein (Fig. 5a). The **eZF-TF** sequences were rationally designed to target the enhancer box (E-box) sequence, following the C. F. Barbas III rules (Fig. 5a)^57^. Each α-helical domain of a ZF motif binds to a 3-bp DNA sequence, and their modular nature allows the overall protein to be reprogrammed for precise targeting of specific DNA sites. Importantly, in addition to the sequence refinement, the peptide linker connecting the ZF motifs plays a significant role in tuning DNA binding by positioning the protein in an optimal orientation^62,63^. Upon structural analysis of the Zif268–DNA complex, we observed that the Gln^363^/Glu^391^ residues are not fully conserved across the Zif268 protein structures; therefore, we decided to insert Cys residues (C^x^) at the Gln^363^/Glu^391^ sites for late-stage modifications to probe the covalent diversification on the linker for ZF-DNA interactions (Fig. 5a).

To enable the structural diversification of **eZF-TF**, we first retrosynthetically disconnected the sequence into three segments, which were masked using the established ReCag approach, to generate the **ZF3-N,** **ZF4-N**, and **ZF5-N** segments (Supplementary sections 4.5–4.7). Subsequently, these segments were ligated by employing a one-pot NCL process to produce **eZF-TF-N** bearing two reactive Cys sites in 40% isolated yield (Fig. 5b). Since the linker in ZF motifs modulates TF–DNA interactions through electrostatic and hydrogen-bonding interactions with the DNA and nearby residues, we sought to diversify the linker using native and modified residues capable of modulating these vital interactions. This includes inserting Glu and Gln mimics via S-alkylation to probe the effect of side-chain elongation at the Gln363 and Glu391 sites. Lys mimics bearing ammonium and methylammonium residues were also selected to further tune the side-chain length and electronic properties. In addition, a phospho-Tyr mimic was incorporated, providing further structural and electronic diversification (Fig. 5b). To evaluate the feasibility of installing dual transformations, **eZF-TF-N** was independently treated with 2-bromoacetamide, 2-bromoacetic acid, 2-bromoethylamine hydrobromide, 2-chloro-N-methylethanamine hydrochloride, and Pd(II)OAC-1 reagents (Supplementary sections 8.1–8.7). Importantly, efficient Cys modification was achieved in all cases, enabling a subsequent photolysis step to decage the six native Cys residues, yielding the desired 10 kDa-modified analogs **eZF-TF1,** **eZF-TF2,** **eZF-TF3,** **eZF-TF4**, and **eZF-TF5**, respectively. The identity and purity of all isolated **eZF-TF1-5** analogs and wild-type analog (**eZF-TFwt**) were confirmed by LC-MS and SDS-PAGE analysis (Fig. 5c and Supplementary section 9). Finally, we characterized the folding of the engineered **eZF-TF** analogs by circular dichroism (CD) spectroscopy (Supplementary section 10). CD analysis revealed that all synthetic **eZF-TF** proteins retained the characteristic spectral features of the folded zinc finger ββα motif, exhibiting pronounced negative bands at 208 and 222 nm (Fig. 5d). Compared with **eZF-TFwt**, the **eZF-TF1–3** analogs exhibited stronger ellipticity, consistent with increased α-helical content and structural order. In contrast, the **eZF-TF4** and **eZF-TF5** analogs displayed similar ellipticity to that of **eZF-TFwt**, indicating preservation of the overall folded structure. Although all variants preserved the overall zinc finger fold, differences in ellipticity were observed, suggesting that the nature of the introduced modification influences protein stability and secondary-structure content. These findings demonstrate that the chemical modifications do not disrupt the global structural integrity of the zinc finger domain but can modulate its conformational stability.

Late-stage editing of **eZF-TFs** reveals potent TF analogs with enhanced DNA binding. To evaluate the effect of the covalent modifications, each **eZF-TF** analog was incubated separately with a DNA probe containing the canonical E-box sequence, and DNA binding was assessed using the electrophoretic mobility shift assay (EMSA). Interestingly, although most of the analogs were associated with DNA, as indicated by a significant upward shift in EMSA, we observed variations in their DNA binding properties, indicating the crucial role of the inserted modifications on DNA binding (Supplementary section 11, and Fig. 5e). We found that the Gln- (**eZF-TF1**), Glu- (**eZF-TF2**), and Lys-mimic (**eZF-TF3**) analogs exhibited potent DNA-binding activity when exposed to the E-box, outperforming **eZF-TFwt**. The enhanced binding observed can be attributed to the stabilization of **eZF-TF1-3** folding, as demonstrated by CD spectroscopy and chain extension, which can potentially stabilize the ZF–DNA interactions through the formation of salt bridges and hydrogen-bonding interactions between the DNA and ZF backbone, respectively^53^. In contrast, methyl-Lys (**eZF-TF4**) and phospho-Tyr (**eZF-TF5**) mimics exhibited reduced DNA binding activity compared with **eZF-TF1-3**. This reduction can be attributed to the steric and electrostatic changes introduced by these modifications, which may impair ZF–DNA interactions^64,65^. To further investigate the DNA-binding properties of the designed **eZF-TF** analogs, we analyzed their interactions with the E-box DNA sequence using bio-layer interferometry (BLI). Consistent with the EMSA results, the lead analogs **eZF-TF1–3** retained potent DNA-binding activity, exhibiting dissociation constants (*K*_D_) of 54.4, 54.6, and 39.3 nM, respectively (Supplementary section 12 and Fig. 5f). In contrast, **eZF-TF4** and **eZF-TF5** displayed markedly reduced DNA-binding affinities, with *K*_D_ values of 121.0 nM and 3.56 µM, respectively. These findings further emphasize the significant role of chemical modifications in tuning TF function. Overall, ReCag enables late-stage and efficient diversification at selected sites in Cys-rich TFs, providing insight into the structural diversification of key domains governing TF–DNA interactions and enabling the discovery of potent **eZF-TF** analogs that effectively bind to the E-box DNA sequence.

**Fig. 6:**
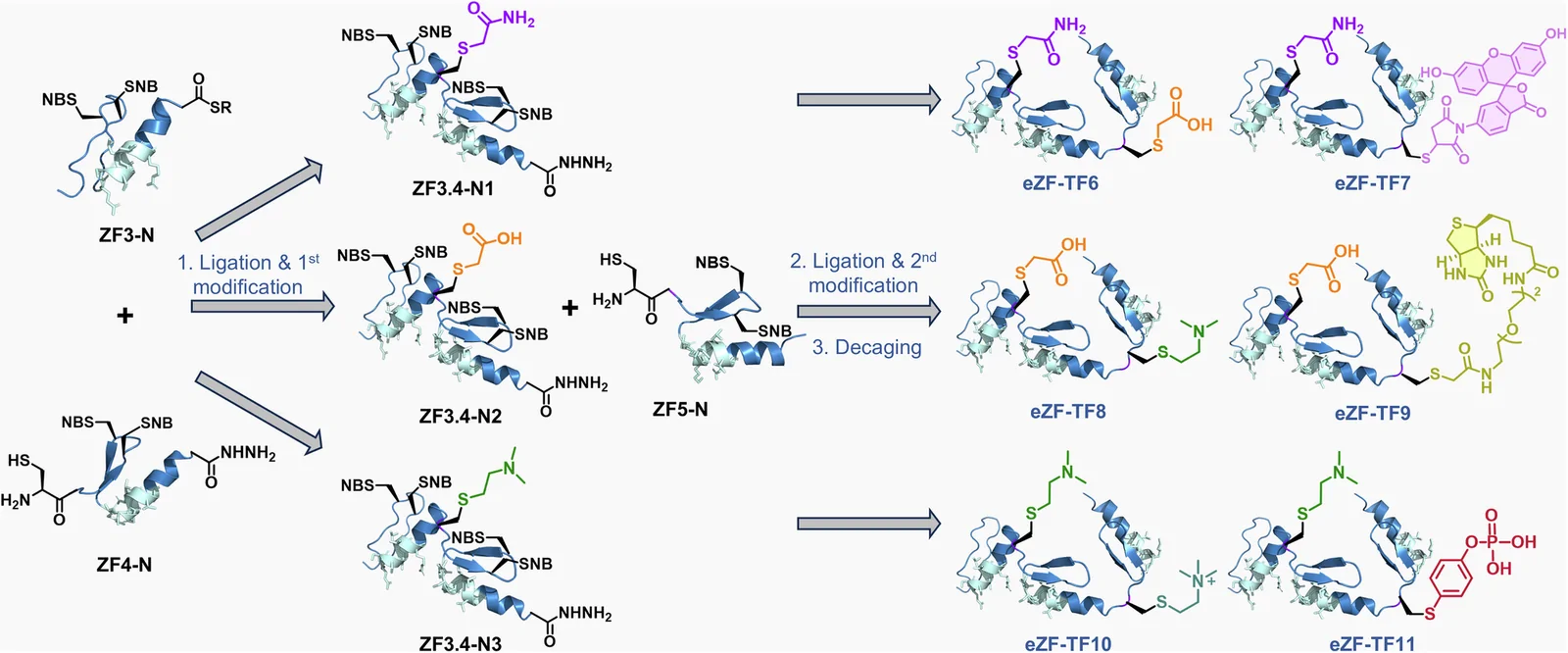
Late-stage installation of distinct modifications for the generation of dual-modified zinc finger proteins. Schematic representation of the late-stage dual functionalization of ZF to generate **eZF-TF6-11** analogs; Conditions: Supplementary section 8.

Finally, we demonstrated the power of the ReCag strategy by enabling the orthogonal, site-selective editing of two distinct sites within the target protein. Using the masked peptide segments **ZF3-N,** **ZF4-N**, and **ZF5-N**, we achieved the sequential incorporation of two distinct modifications in a site-selective manner (Fig. 6). This was accomplished by first assembling **ZF3-N** and **ZF4-N** via NCL, followed by late-stage functionalization to afford the monofunctionalized intermediates **ZF3.4-N1-3** (Supplementary section 8.8). These intermediates were subsequently ligated with **ZF5-N** and subjected to a second late-stage functionalization to furnish doubly functionalized zinc finger analogs. Finally, a global Cys-decaging step afforded the desired doubly modified analogs bearing native amino acid analogs, PTM mimics, and probes. Importantly, the ability to introduce two distinct modifications at defined sites through a modular, late-stage diversification strategy further establishes ReCag as a versatile platform for the rapid preparation of multifunctional proteins from common synthetic intermediates.

## Discussion

Site-selective functionalization strategies have emerged as a powerful strategy for protein modification, spurring significant advances in biomedical research and therapeutic development^9^. Although Cys-based chemical reactions have enabled diverse protein modifications, these methods have mainly been limited to proteins bearing a single reactive Cys^4,5^. Expanding this to Cys-rich proteins typically requires incorporating an exogenous peptide sequence into the target protein. To address these challenges, we developed a versatile ReCag strategy for site-selective modification of Cys-rich peptides and proteins, enabling the precise insertion of diverse functionalities. The ReCag workflow involves splitting the protein of interest into two segments for the strategic masking of reactive Cys sites, enabling ligation-based reassembly of the protein sequence. This control allows site-selective modification of Cys at the ligation junction, ultimately yielding the desired modified native sequence following photochemical decaging of internal Cys residues. Importantly, the ReCag strategy offers several key advantages, including a tag- and ligand-free approach, enabling efficient modification without sequence intervention. This method is fully compatible with a broad range of transformations, including alkylation, arylation, elimination, and maleimide chemistry, which are widely used in the bioconjugation of biomolecules. Importantly, this strategy provides a versatile platform for introducing a broad range of novel transformations, including amino acid mimics, PTM analogs, synthetic tags, and molecular probes into Cys-rich targets, enabling site-specific monolabeling and the orthogonal installation of distinct modifications to generate precisely dual-modified proteins, as demonstrated by the site-selective modification of 30 analogs in high yields. This includes the diversification of Cys-rich proteins, such as ZF-TFs bearing 5–8 Cys residues, which were site-selectively modified without altering the native sequence. Such control over ZF-TF modification enabled the discovery of potent **eZF-TF** analogs with enhanced DNA-binding activity.

Unlike conventional synthetic approaches, in which each new analog requires independent peptide synthesis and ligation, our strategy generates a common synthetic intermediate that can be diversified at a late stage through a broad range of Cys-selective transformations. This modular workflow enables the rapid generation of modified proteins from the same building blocks, facilitates the exploration of diverse chemical modifications, including combinations of PTM mimics, and substantially reduces the synthetic effort required to produce large numbers of protein variants. Importantly, the modular and orthogonal nature of ReCag is envisioned to facilitate the efficient diversification of Cys-rich proteins, establishing a versatile platform for the generation of diverse protein libraries for a wide range of applications. Because the reversible masking reaction is performed in solution, the methodology is also envisioned to be readily compatible with recombinantly produced protein or peptide segments, suggesting future integration with expressed protein ligation for the site-selective modification of larger protein targets^66,67^. Although the current approach requires pre-masking of the target peptide segment prior to ligation rather than direct functionalization of the intact protein, it offers a modular platform for the rapid generation of diverse protein variants from a common intermediate. Future efforts will aim to translate this concept to intact proteins by developing strategies for the selective masking and diversification of native protein targets. Taken together, we anticipate the generality and flexibility of the ReCag approach to drive the incorporation of novel transformations into other complex Cys-rich proteins with increased size and complexity for various applications^68,69^. Finally, in addition to modulating TF-DNA interactions, we envision that strategic modifications of ZFs to refine other key properties, such as cell penetration, can provide advanced analogs for modulating gene expression^57,70^. This and other research programs are currently under investigation in our laboratory.

## Methods

Methods, synthetic procedures, and biochemical and biophysical characterization of the synthesized compounds are described in detail in the Supplementary Information.

### General procedure for selective Cys masking

The targeted polypeptide (1.0 equiv.) was dissolved in 6 M Gun.HCl, 0.2 M Na_2_HPO_4_ buffer, followed by the addition of TCEP (5.0 equiv.). Aqueous formaldehyde (2.5 equiv.) was then added, and the reaction pH was adjusted to 6.8 using 5 *N* NaOH. The reaction mixture was then incubated at 37 °C for 4 h, with reaction progress monitored using LC-MS. Upon completion of thiazolidine ring formation, NB-Br (5.0 equiv.) was dissolved in acetonitrile and added directly to the reaction mixture. The pH of the reaction mixture was adjusted to 7.5 using 5 N NaOH, and the reaction mixture was incubated at rt for 1 h. The reaction progress was monitored by LC-MS. Upon complete masking of internal Cys residues, the resulting Cys-caged polypeptide was purified by RP-HPLC.

For selective conversion of the N-terminal thiazolidine to the corresponding free Cys residue, the purified Cys-caged polypeptide (1.0 equiv.) was dissolved in 6 M Gun·HCl, 0.2 M Na_2_HPO_4_ buffer, followed by addition of TCEP (5.0 equiv.) and NH_2_OMe.HCl (25 equiv.). The reaction pH was adjusted to 4 using 5 N HCl, and the reaction mixture was incubated at 37 °C for 1 h. Reaction progress was monitored by LC–MS. Upon completion, the resulting polypeptide bearing N-terminal free Cys was isolated by RP-HPLC purification.

### General procedure for peptide ligation

The peptide hydrazide bearing masked Cys residues (1.0 equiv.) was dissolved in 6 M Gun.HCl, 0.2 M Na_2_HPO_4_ buffer (pH 3.0) and cooled to −15 °C in an ice/salt bath. Aqueous NaNO_2_ (10 equiv.) was then added, and the reaction mixture was incubated at −15 °C for 15 min with gentle mixing every 5 min. Subsequently, MPAA (50 equiv.) in 6 M Gun.HCl, 0.2 M Na_2_HPO_4_ buffer (pH 6.8) was added, and the mixture was gently mixed for another 3 min. The second peptide segment bearing a free N-terminal Cys and masked internal Cys residues (1.0 equiv.) was then added, and the pH was adjusted to 7.2 using 5 *N* NaOH. The reaction mixture was incubated at rt for 1 h, followed by addition of TCEP (40 equiv.) in 6 M Gun.HCl, 0.2 M Na_2_HPO_4_ buffer (pH 7.2). The reaction was continued for an additional 15 min at rt and monitored using LC-MS. Upon completion, the ligated polypeptide was isolated by RP-HPLC purification.

### General procedure for site-selective modification and Cys decaging

The targeted polypeptide (1.0 equiv.) was dissolved in 6 M Gun.HCl, 0.2 M Na_2_HPO_4_ buffer (pH 7.5), followed by the addition of Pd(II)OAC (2.5–5.0 equiv.) dissolved in N,N-Dimethylformamide (10% v/v). The reaction mixture was vigorously mixed for several seconds and then incubated at 37 °C for 15 min. Reaction progress was monitored by LC–MS. Upon completion, the reaction was quenched by the addition of 3-MPA (10 equiv. relative to Pd(II)OAC), and the mixture was incubated at 37 °C for an additional 15 min. The reaction mixture was then centrifuged, and the modified polypeptide was purified by RP-HPLC.

The isolated site-selectively modified polypeptide was subsequently dissolved in 6 M Gun.HCl, 0.2 M Na_2_HPO_4_ buffer, followed by the addition of DTT (10.0 equiv. per caged-Cys residue). The pH of the reaction mixture was adjusted to 4 using 5 N HCl and then irradiated with 350 nm light at rt for 1–4 h. Reaction progress was monitored by LC–MS. Upon completion, the desired polypeptide bearing site-selective modification and regenerated free Cys residues was isolated by RP-HPLC purification.

### CD analysis

CD analysis was performed using a Chirascan circular dichroism spectrometer with a 0.1 mm path-length quartz cuvette. **eZF-TF** variants were prepared at a concentration of 20 µM in 50.0 µL of 10 mM Tris.HCl, 50 mM NaCl, 100 mM ZnCl_2_, 1 mM TCEP, 5% glycerol buffer (pH 7.3). CD spectra of all samples were recorded in triplicate at 20 °C from 190 nm to 260 nm in a 1.0 nm step with a 3.0 nm slit bandwidth and an averaging time of three seconds per wavelength.

### EMSA analysis

To a 0.2 mL Eppendorf tube, 36.0 µL of 10 mM Tris.HCl, 50 mM NaCl, 75 µM ZnCl2, 1 mM DTT, 5% glycerol buffer, pH 7.3 (folding buffer) were added (except for the well containing only DNA, in this case containing 38.0 µL of the folding buffer). E-box DNA probe (2.0 µL, 10.0 µM; 1 equiv.) as a solution in the folding buffer and **eZF-TFwt,** **eZF-TF1-5** analogs (2.0 µL, 40.0 µM; 4 equiv.) as a solution in the folding buffer were added. The final incubation concentration of the major components was the following: DNA (0.5 µM) and protein (2.0 µM) in the folding buffer. The Eppendorf tube was closed, mixed, and incubated at rt for 30 min. Next, the DNA-binding activity of each analog was analyzed by EMSA: 5.0 µL of each DNA-protein mixture was mixed with 1.0 µL DNA Loading Dye (6X). 5.0 µL of each mixture was loaded onto a 10% TBE gel, 1.0 mm × 10 well and run in 1x TBE at 90 V for 45 min. The gel was washed three times with water for 30 s and then stained using Ethidium Bromide in 1x TBE buffer for 15 min at rt. Bands were visualized on A2S Vilber Fusion FX imager.

### BLI assay

BLI assay was performed using an Octet Red R4 System (ForteBio; Menlo Park, CA) in 96-well plates. Streptavidin Octet biosensors (ForteBio; Menlo Park, CA) were dipped into 200 µL of 0.1% BSA, 0.02% Tween-20, 1x PBS (kinetic buffer) for 10 min and then 60 more seconds in the kinetic buffer to obtain the baseline. Then the sensors were dipped into 200 µL of 65 nM biotinylated E-box DNA probe in the kinetic buffer for the loading step (300 s). Sensors were then dipped into the kinetic buffer for 120 s. Next, the tips were loaded with folded **eZF-TF** analog prepared in kinetic buffer at the indicated concentrations for 210 s to obtain the association curve. Then the tips were dipped into the kinetic buffer for 150 s to obtain the dissociation curve. Measurements were carried out at 25 °C. Data were analyzed within the ForteBio Data Analysis software. The association and dissociation curves were fitted with Fortebio Biosystems with a 1:1 Binding Model (global fitting algorithm) to obtain the *K*_D_.

### Reporting summary

Further information on research design is available in the Nature Portfolio Reporting Summary linked to this article.

## Supplementary information

Supplementary information

Reporting Summary

Transparent Peer Review File

## Source data

Source Data

## Data Availability

All data are available in the main text or the supplementary information and are also available from the corresponding author upon request. Source data are provided with this paper.
