# Supplementary material for: Site-specific protein functionalization by late-stage reversible cysteine masking
Source: Nat Commun. 2026 Sep 10;17:10669. doi: 10.1038/s41467-026-77718-3 (PMC13655035; doi:10.1038/s41467-026-77718-3)
Supplement: Supplementary file 1 [file 41467_2026_77718_MOESM1_ESM.pdf]

Supplementary Information

Site-Specific Protein Functionalization by Late-Stage Reversible Cysteine Masking

Manjeet Singh,[1] Raj V. Nithun,[1] Omer Harel,[1] and Muhammad Jbara[1]*

[1]School of Chemistry, Raymond and Beverly Sackler Faculty of Exact Sciences, Tel Aviv University, Tel Aviv, 69978 Israel.

*Correspondence to: jbaram@tauex.tau.ac.il

Table of Contents

1. EXPERIMENTAL 3
2. PEPTIDE, PROTEIN, AND DNA SEQUENCES 5
3. GENERAL PROCEDURE FOR THE PEPTIDE SYNTHESIS 6
4. SELECTIVE MASKING OF PEPTIDE SEGMENTS 12
5. CHEMOSELECTIVE PEPTIDE LIGATION 23
6. SITE-SPECIFIC MODIFICATION OF MODEL PEPTIDE P 30
7. SITE-SPECIFIC MODIFICATION OF ZF PROTEIN 36
8. SITE-SPECIFIC MODIFICATION OF EZF-TF PROTEIN 67
9. SDS-PAGE ANALYSIS 78
10. CIRCULAR DICHROISM (CD) ANALYSIS 79
11. ELECTROPHORETIC MOBILITY-SHIFT ASSAY (EMSA) 79
12. OCTET BIOLAYER INTERFEROMETRY BINDING ASSAY (BLI) 80
13. SUPPLEMENTARY REFERENCES 81

1. Experimental

1.1 Materials

Fmoc-L-Ala-OH, Fmoc-L-Val-OH, Fmoc-L-Asn(Trt)-OH, Fmoc-L-Gln(Trt)-OH, Fmoc-L-Arg(Pbf)-OH, Fmoc-L-Tyr(*t*Bu)-OH, Fmoc-L-Glu(O*t*Bu)-OH, Fmoc-L-Phe-OH, Fmoc-L-Leu-OH, Fmoc-L-His(Trt)-OH, Fmoc-L-Asp(O*t*Bu)-OH, Fmoc-L-Pro-OH, Fmoc-L-Cys(Trt)-OH, Fmoc-L-Lys(Boc)-OH, Fmoc-L-Ile-OH, Fmoc-L-Ser(*t*Bu)-OH, Fmoc-Gly-OH, Fmoc-L-Nle-OH, Fmoc-L-Thr(*t*Bu)-OH, DL-methionine, 1,3-Diisopropylcarbodiimide (DIC), 4-Bromobenzylphosphonic acid, 2-Nitrobenzyl bromide (NB-Br), 4-Iodophenol, Iodobenzene, L-Glutathione reduced (GSH), 2-Bromoethylamine hydrobromide, 2-Chloro-N-methylethanamine hydrochloride, 2-Bromo-N,N-dimethylethylamine hydrobromide, (2-bromoethyl)trimethylammonium bromide, 2-Bromoacetic acid, 2-Bromoacetamide, Biotin polyethyleneoxide iodoacetamide, 1-(2-Bromoethoxy)-2-(2-methoxyethoxy)ethane, Cyclohexane (≥99.8 ReagentPlus®), and Formaldehyde solution (ACS reagent, 37 wt. % in H_2O, contain 10-15% methanol as stabilizer) were purchased from Sigma-Aldrich. (2-(1H-benzotriazol-1-yl)-1,1,3,3-tetramethyluronium hexafluorophosphate (HBTU), HOBt hydrate and Ethyl cyanohydroxyiminoacetate (oxyma) were purchased from Luxembourg Bio Technologies Ltd. HO-TCP(Cl)-ProTide resin and Rink Amide ProTide resin were obtained from CEM. 2-Dicyclohexylphosphino-2′,6′-diisopropoxybiphenyl (RuPhos), DL-Dithiothreitol (DTT) and Tris(2-carboxyethyl)phosphine hydrochloride (TCEP.HCl) were purchased from Angene chemical private limited. 4-Mercaptophenylacetic acid (MPAA) was purchased from Combi-Blocks. 4-Bromo-2-nitrophenol (98%) was purchased from Thermo Scientific. Diethyl ether (Et_2O, stabilized/BHT, AR grade), Methanol (MeOH, HPLC Grade), Dichloromethane (CH_2Cl_2, stabilized with amylene, peptide synthesis grade), N,N-dimethylformamide (DMF, peptide synthesis and AR grade), Acetonitrile (LC/MS and HPLC Grade), Trifluoroacetic acid (TFA, ≥99% ReagentPlus®), Diisopropylethylamine (DIEA, ≥99% ReagentPlus®), Piperidine (≥99% ReagentPlus®), Triisopropylsilane (TIS, 98%), Formic acid (98-100% for LC/MS), n-Pentane (AR), and Dimethyl sulfoxide (DMSO, ≥99.5% ReagentPlus®) were purchased from Bio-Lab ltd. TBE Running buffer (5X), Acrylamide solution (40%), Tetramethylethylenediamine (TEMED), Ammonium Persulfate (APS), and 6X DNA Loading Dye were purchased from Thermo Fisher Scientific. E-box DNA probe was purchased from Integrated DNA Technologies. Water for all reactions carried out on proteins and for reverse-phase purification was obtained via filtration of deionized water through a MilliporeSigma™ Milli-Q™ Ultrapure Water System. All chemicals obtained from the supplier were used as received without further purification.

1.2 Analytical LC-MS analysis

Analytical LC was acquired using Thermo Scientific Vanquish HPLC. The mobile phases used are solvent A (0.1% formic acid in water) and solvent B (0.1% formic acid in acetonitrile), and mass spectrometry using a Thermo Scientific ISQ EM Mass spectrometer.

Method A: bioZen™ 2.6 µm-C4 Widepore LC column (150 x 2.1 mm); LC conditions: 5% B from 0–1.0 min, then a linear gradient from 5% to 50% B from 1.0–11.0 min (i.e., 4.5% per min), 0.3 mL/min flow rate.

Method B: XBridge® Protein BEH 2.5 µm-C4 300 Å LC column (150 x 2.1 mm); LC conditions: 5% B from 0–1.0 min, then a linear gradient from 5% to 50% B from 1.0–11.0 min (i.e., 4.5% per min), 0.3 mL/min flow rate.

All peptide and protein samples were analysed at least once (n=1) using an LC-MS instrument.

1.3 Preparative RP-HPLC purification

Preparative RP-HPLC was performed using Thermo Scientific DIONEX UltiMate 3000 Variable Wavelength Detector, equipped with a column of choice. Mobile phases used for LC analysis were solvent A (0.05% TFA in water) and solvent B (0.05% TFA in acetonitrile). The following LC methods were used:

Method A: XBridge® Protein BEH C4 OBD™ Prep Column, 300 Å, 5 µm, 250 mm x 10 mm, LC conditions: 5% B from 0–5.0 min, then a linear gradient from 5% to 60% B from 5-65 min (i.e. 1% per min), 4 mL/min flow rate at 30 °C.

Method B: XBridge® Protein BEH C4 OBD™ Prep Column, 300 Å, 5 µm, 250 mm x 10 mm, LC conditions: 5% B from 0–5 min, then a linear gradient from 5% to 20% B from 5-10 min (i.e. 3% per min), followed by another linear gradient from 20% to 60% B from 10-50 min (i.e. 1% per min), 4 mL/min flow rate at 30 °C.

Method C: Jupiter® 5 µm C18 300 Å LC column (250 x 10 mm), LC conditions: 5% B from 0–5.0 min, then a linear gradient from 5% to 60% B from 5-65 min (i.e. 1% per min), 4 mL/min flow rate at 30 °C.

Method D: Jupiter® 5 µm C18 300 Å LC column (250 x 10 mm), LC conditions: 5% B from 0–5 min, then a linear gradient from 5% to 20% B from 5-10 min (i.e. 3% per min), followed by another linear gradient from 20% to 60% B from 10-50 min (i.e. 1% per min), 4 mL/min flow rate at 30 °C.

1.4 UV Irradiation

The UV irradiation reactions were carried out using a Rayonet photochemical chamber mini reactor (RMR600) equipped with eight Rayonet lamps (350 nm, 4 W), each 6 inches in length.

2. Peptide, protein, and DNA sequences

Model peptide (P):

RLYRGCAYLS**C**RLYRGCAYLK

Zif268 protein (ZF):

E^{333}RPYACPVESCDRRFSRSDELTRHIRIHTG**Q**^{363}KPFQCRICMRNFSRSDHLTTHIRTHTGEK^{392}

eZF-TF:

M^{332}ERPYACPVESCDRRFSQSSSLVRHIRIHTG**Q**^{363}KPFQCRICMRNFSRSDELVRHIRTHTG**E**^{391} KPFACDICGRKFASKKALTEHTKIHLRQKD^{421}

Met residues at positions 332 and 372 were mutated to the homologous norleucine (Nle) residue to avoid Met oxidation. The Gln and Glu residues at positions 363 and 391 were mutated to Cys for late-stage modification using ReCag.

E-box DNA probe:

5’-CCGGCTGACACGTGGTATTAAT-3’

3. General procedure for the peptide synthesis

3.1 Manual solid phase peptide synthesis (SPPS):

The synthesis of peptide segments was carried out using manual Fmoc-SPPS. The resin was pre-swollen in DMF for 30 min using a fritted syringe. The amino acids were then manually coupled using Fmoc-L-AA-OH (5 equiv.) and coupling reagents (HBTU/HOBt 5 equiv. and DIEA 10 equiv.; DIC/HOBt, 5 equiv.; HATU 5 equiv. and DIEA 10 equiv.; or DIC/OxymaPure, 5 equiv.) in DMF for 15–45 min at room temperature (rt). After each coupling step, the resin was washed three times with DMF and subjected to Fmoc deprotection using 20% piperidine and 0.05% formic acid in DMF (3 min × 3), followed by washing with DMF (10 mL × 3). After completion of the coupling of all amino acids, the peptide-bound resin was washed with DMF (10 mL × 3) and DCM (10 mL × 3), and then dried under vacuum.

3.2 Resin cleavage:

A mixture of TFA/H_2O/TIS (95:2.5:2.5, 10 mL for a 0.05 mmol scale) was added to the resin and shaken for 2-4 h at rt. The resin was removed by filtration and washed with TFA (1 mL × 2). To precipitate the peptide, the combined filtrate was added dropwise to cold diethyl ether, followed by centrifugation at 2610 x g for 10 min. The diethyl ether was then decanted, and the peptide was dissolved in 10 mL of 25% acetonitrile/water and lyophilized to afford a white powder.

3.3 Synthesis of P1

The hydrazino resin was prepared as described previously.[1] The synthesis of **P1** was carried out using manual Fmoc-SPPS, as described in section 3.1. After completion of the synthesis, the resin was cleaved using a TFA cocktail described in section 3.2 to afford **P1** (31% isolated yield based on 0.1 mmol resin) as a white powder after RP-HPLC purification (Method A, as described in Section 1.3).

A

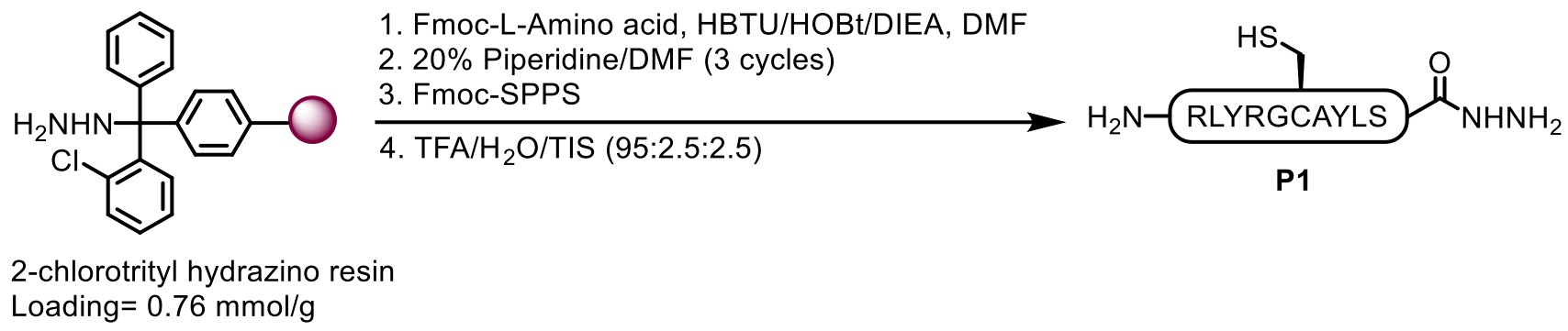

B

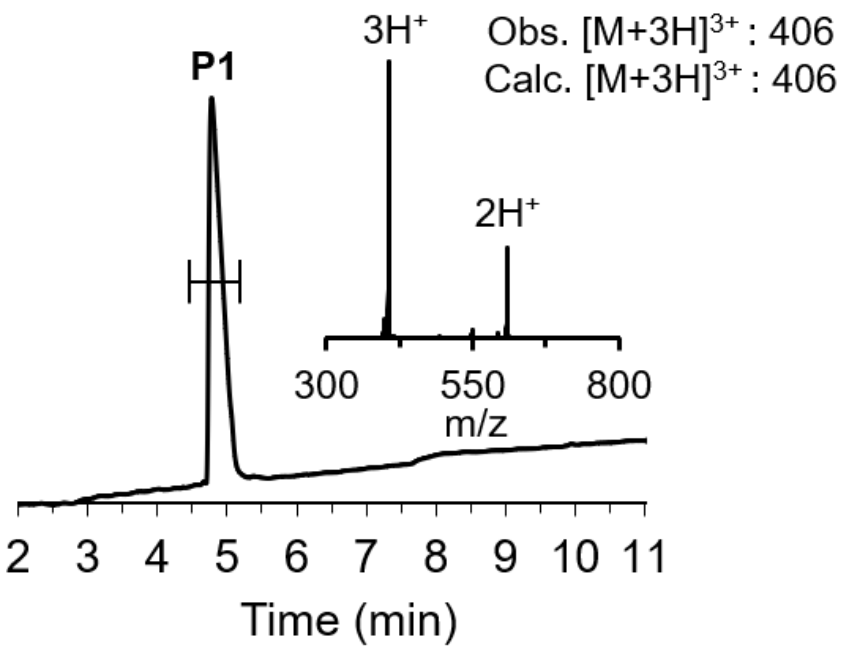

Supplementary Fig. 1 A Schematic representation for the SPPS of **P1**. **B** LC-MS analysis of isolated **P1** using method A as described in section 1.2.

3.4 Synthesis of P2

The synthesis of **P2** was carried out using manual Fmoc-SPPS, as described in section 3.1, using Rink amide ProTide™ resin. After completion of the synthesis, the resin was cleaved using a TFA cocktail described in section 3.2 to afford **P2** (38% isolated yield based on 0.1 mmol resin) as a white powder after RP-HPLC purification (Method A, as described in Section 1.3).

A

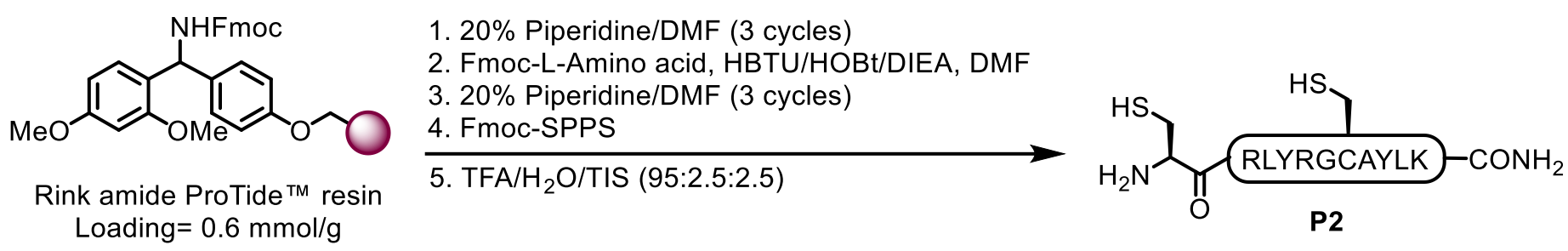

B

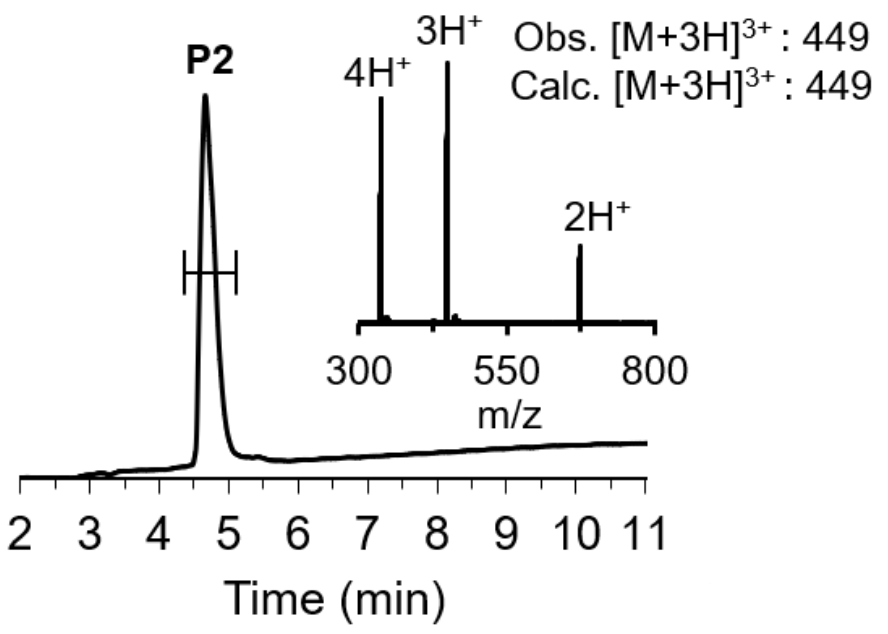

Supplementary Fig. 2 A Schematic representation for the SPPS of **P2**. **B** LC-MS analysis of isolated **P2** using method A as described in section 1.2.

3.5 Synthesis of ZF1

The synthesis of **ZF1** was carried out using manual Fmoc-SPPS, as described in section 3.1, using hydrazino resin. After completion of synthesis, resin was cleaved using a TFA cocktail described in section 3.2 to afford **ZF1** (13% isolated yield based on 0.1 mmol resin) as a white powder after RP-HPLC purification (Method A as described in Section 1.3).

A

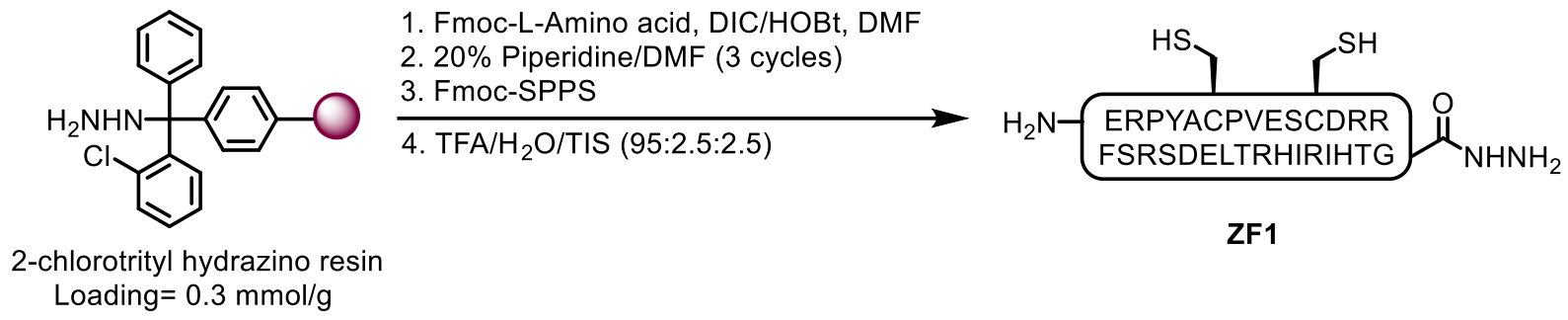

B

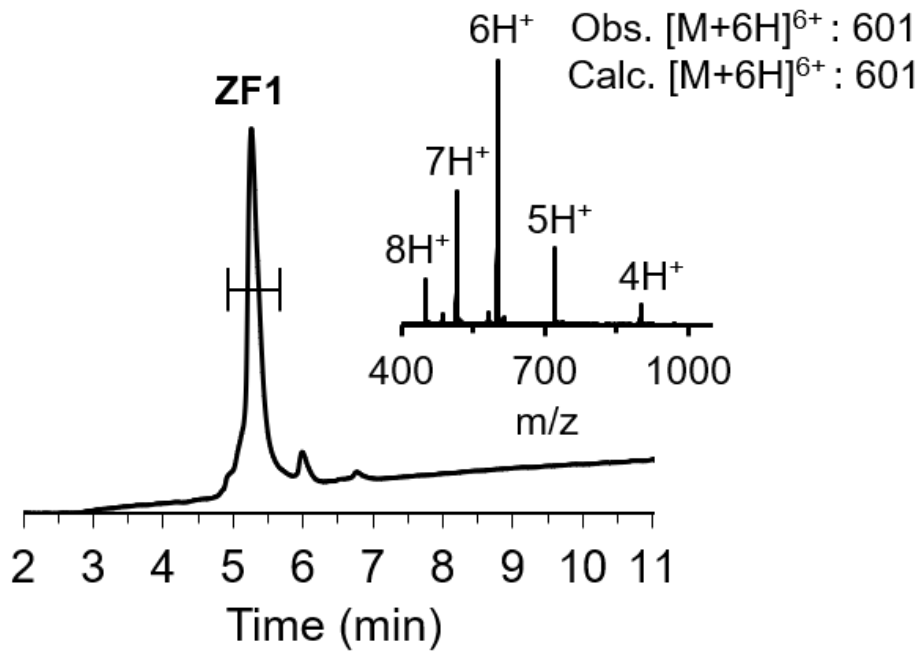

Supplementary Fig. 3 A Schematic representation for the SPPS of **ZF1**. **B** LC-MS analysis of isolated **ZF1** using method A as described in section 1.2.

3.6 Synthesis of ZF2

The synthesis of **ZF2** was carried out using manual Fmoc-SPPS, as described in section 3.1, using Rink amide ProTide™ resin. After completion of synthesis, the resin was cleaved using a TFA cocktail described in section 3.2 to afford **ZF2** (22% isolated yield based on 0.1 mmol resin) as a white powder after RP-HPLC purification (Method A as described in Section 1.3).

A

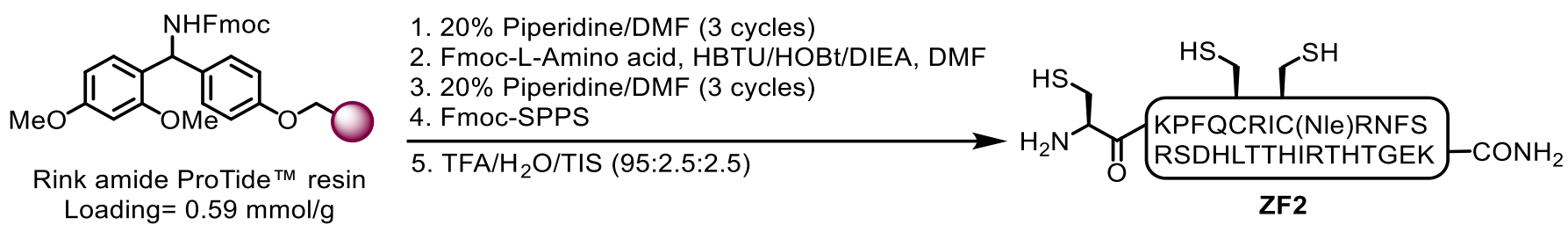

B

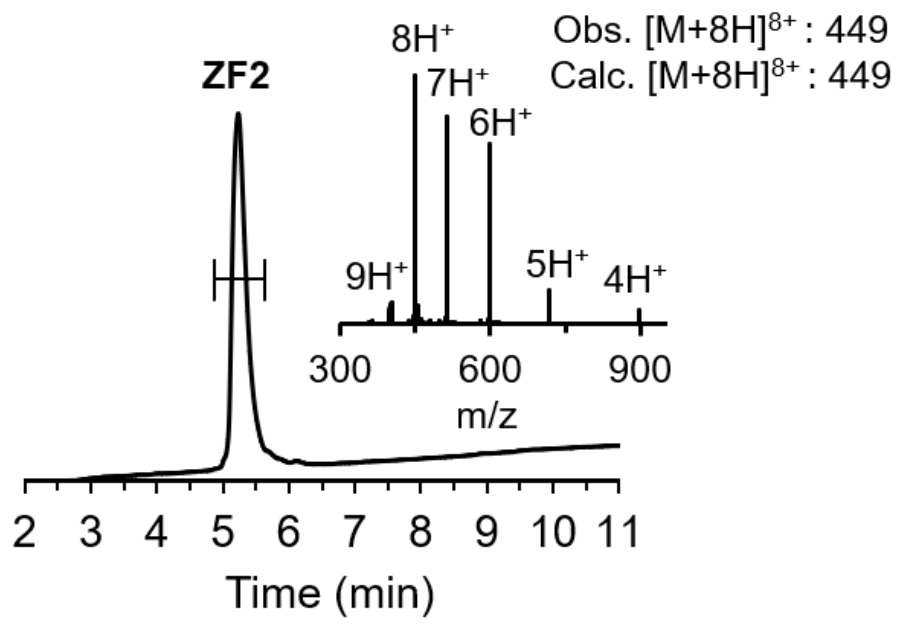

Supplementary Fig. 4 A Schematic representation for the SPPS of **ZF2**. **B** LC-MS analysis of isolated **ZF2** using method A as described in section 1.2.

3.7 Synthesis of ZF3

The synthesis of **ZF3** was carried out using manual Fmoc-SPPS, as described in section 3.1, using hydrazino resin. After completion of the synthesis, the resin was cleaved using a TFA cocktail, as described in section 3.2, to afford **ZF3** (7% isolated yield based on 0.1 mmol resin) as a white powder after RP-HPLC purification (Method A, as described in Section 1.3).

A

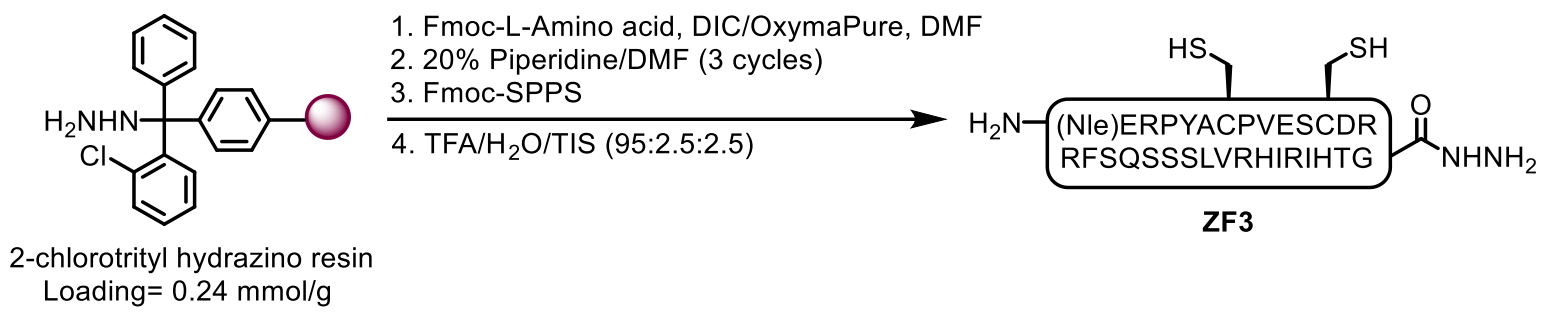

B

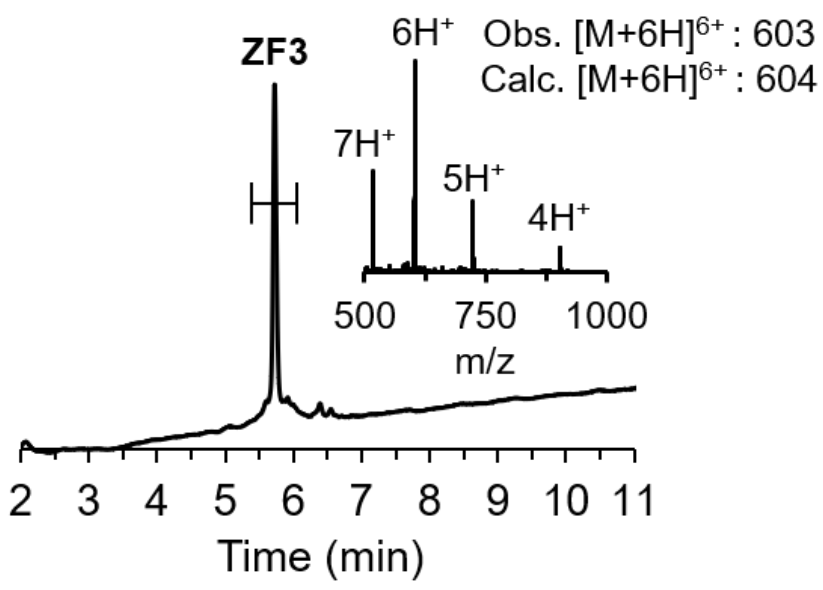

Supplementary Fig. 5 A Schematic representation for the SPPS of **ZF3**. **B** LC-MS analysis of isolated **ZF3** using method B as described in section 1.2.

3.8 Synthesis of ZF4

The synthesis of **ZF4** was carried out using manual Fmoc-SPPS, as described in section 3.1, using hydrazino resin. After completion of the synthesis, the resin was cleaved using a TFA cocktail, as described in section 3.2, to afford **ZF4** (31% isolated yield based on 0.1 mmol resin) as a white powder after RP-HPLC purification (Method A, as described in Section 1.3).

A

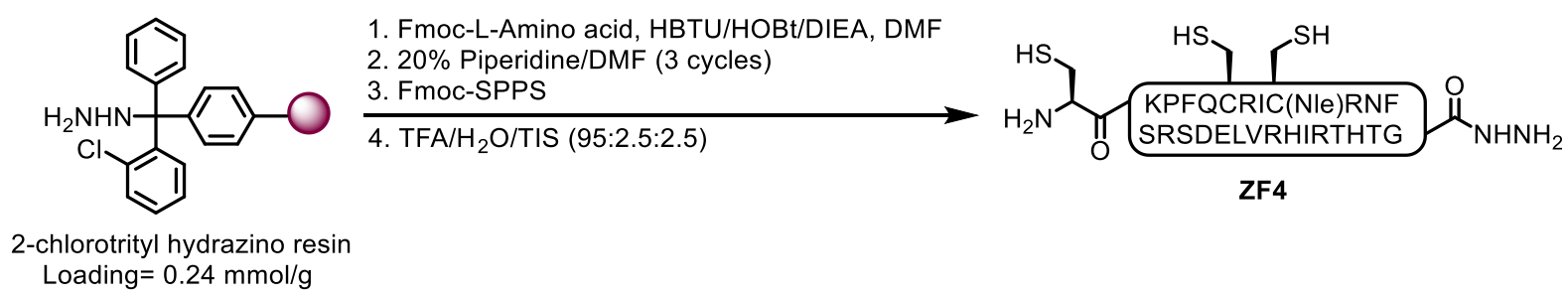

B

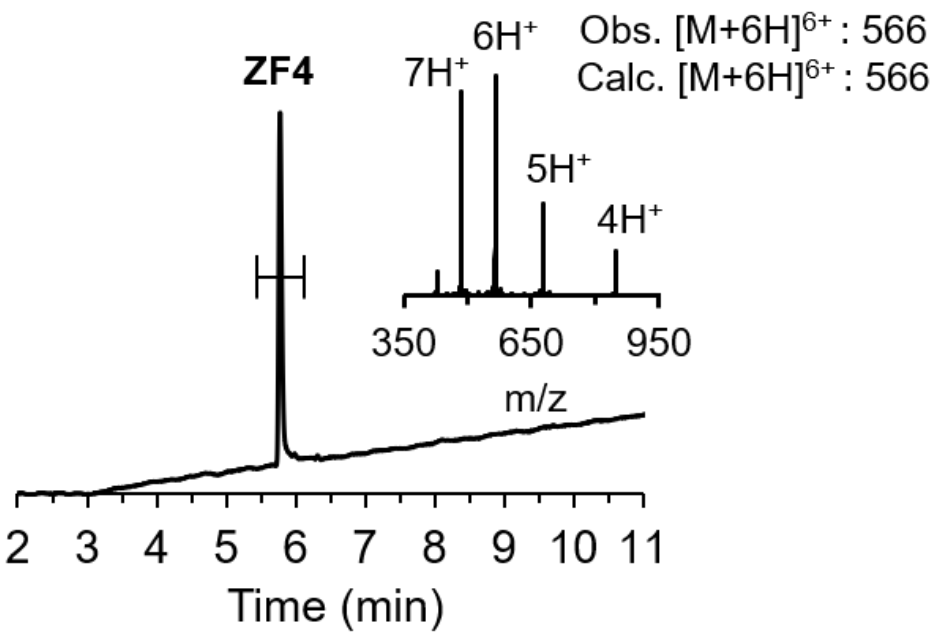

Supplementary Fig. 6 A Schematic representation for the SPPS of **ZF4**. **B** LC-MS analysis of isolated **ZF4** using method B as described in section 1.2.

3.9 Synthesis of ZF5

The synthesis of the **ZF5** was carried out using manual Fmoc-SPPS, as described in section 3.1, using Rink amide ProTide™ resin. After completion of the synthesis, the resin was cleaved using the TFA cocktail described in section 3.2 to afford the **ZF5** (25% isolated yield based on 0.1 mmol resin) as a white powder after purification by RP-HPLC (Method A, as described in Section 1.3).

A

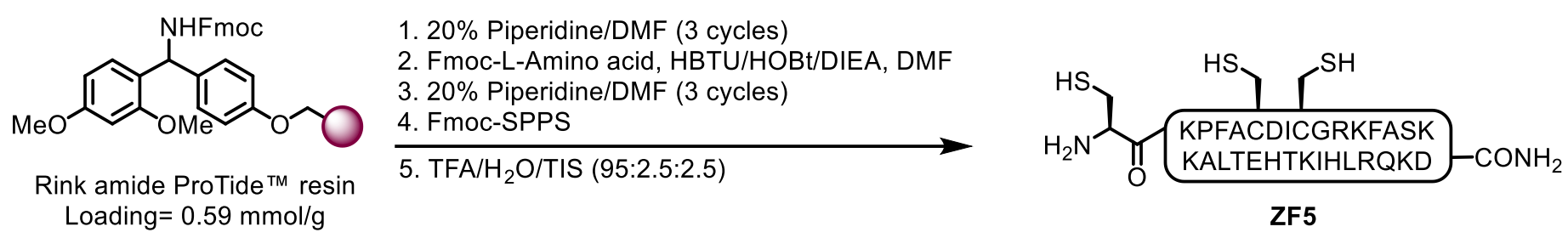

B

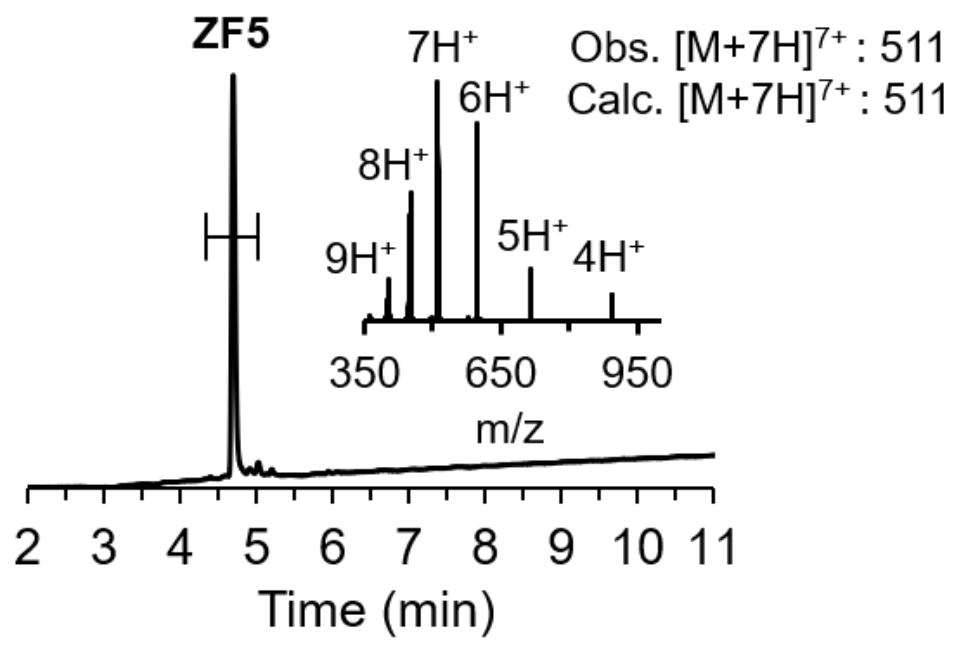

Supplementary Fig. 7 **A** Schematic representation for the SPPS of **ZF5**. **B** LC-MS analysis of isolated **ZF5** using method B as described in section 1.2.

3.10 Synthesis of SID-maleimide peptide

The synthesis of SID-maleimide was carried out using manual Fmoc-SPPS, as described in section 3.1, using Rink amide ProTide™ resin. Alloc was removed using a mixture of $Pd(PPh_3)_4$ (1 equiv.), piperidine (640 µL), and DCM (2.56 mL) that was shaken with the resin for 30 minutes at rt under the exclusion of light. The resin was washed with DCM (6 mL x 6) and DMF (6 mL x 6). Then, 2-maleimidoacetic acid (5 equiv., 0.25 mmol, 125.0 mM) was coupled using HATU (5 equiv., 0.25 mmol, 125.0 mM), and DIEA (10 equiv., 0.5 mmol, 250.0 mM) in 2 mL DMF for 1 hour at 30 °C. After completion of synthesis, resin was cleaved using a TFA cocktail described in section 3.2 to provide SID-maleimide (4% isolated yield, based on 0.05 mmol resin) as a white powder.

A

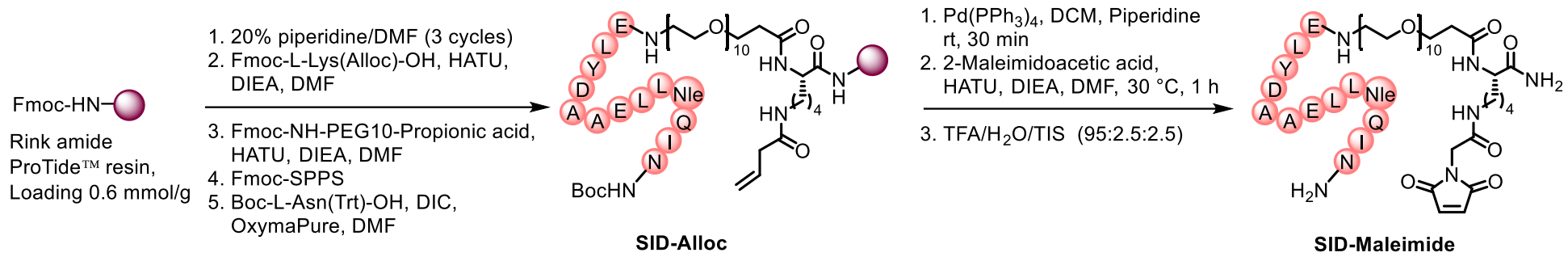

B

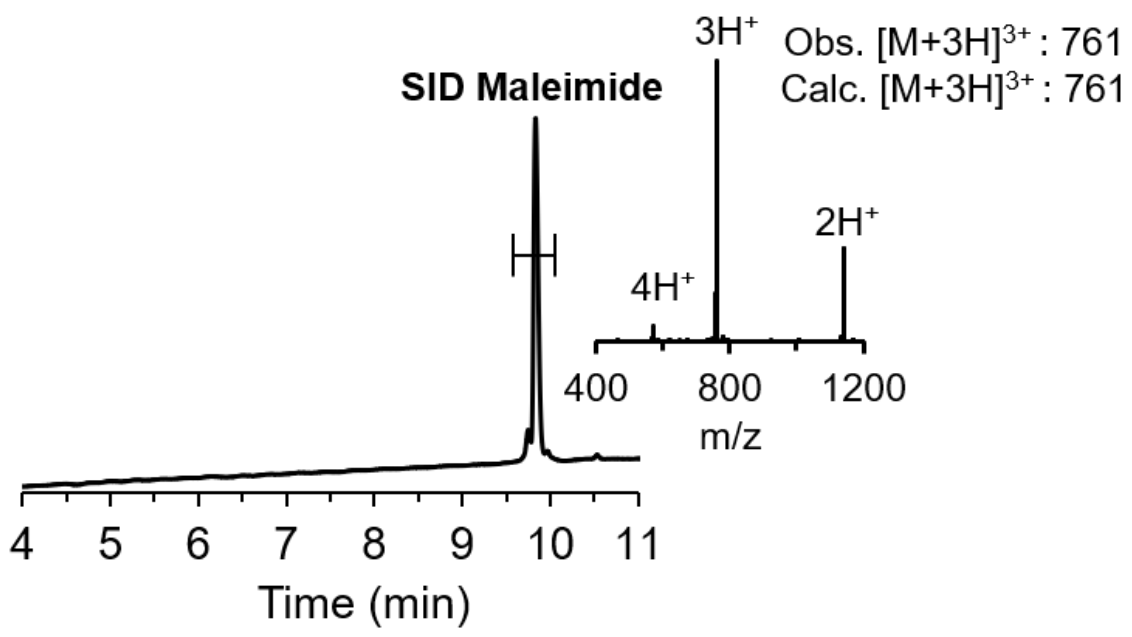

Supplementary Fig. 8 **A** Schematic representation for the SPPS of SID-maleimide. **B** LC-MS analysis of isolated SID-maleimide using method B as described in section 1.2.

3.11 Synthesis of 1-(2-(tert-butyl)-16-(2,5-dioxo-2,5-dihydro-1H-pyrrol-1-yl)-4,13-dioxo-7,10-dioxa-3,14-diazahexadecanoyl)-4-hydroxy-N-(4-(4-methylthiazol-5-yl)benzyl)pyrrol- idine-2-carboxamide (AHPC-maleimide)

In a round-bottom flask, AHPC-NHS (19.5 mg, 27.2 µmol, 1.0 equiv., 56.6 mM) was dissolved in 480 µL of acetonitrile. A solution of 1-(2-aminoethyl)maleimide hydrochloride (32.6 µmol, 1.2 equiv., 0.27 M) in DMSO (120 µL) was added, followed by diisopropylethylamine (7.10 µL, 40.8 µmol, 1.5 equiv.). The reaction mixture was stirred for 20 min at rt, and the solvents were removed by rotary evaporation. The product was purified by RP-HPLC (Method A, Section 1.3) and lyophilized to afford AHPC-maleimide as a white powder (86% isolated yield). HRMS (ESI) m/z calculated for $[M+H]^+$ $C_{36}H_{49}N_6O_9S$: 741.3282; found: 741.3289.

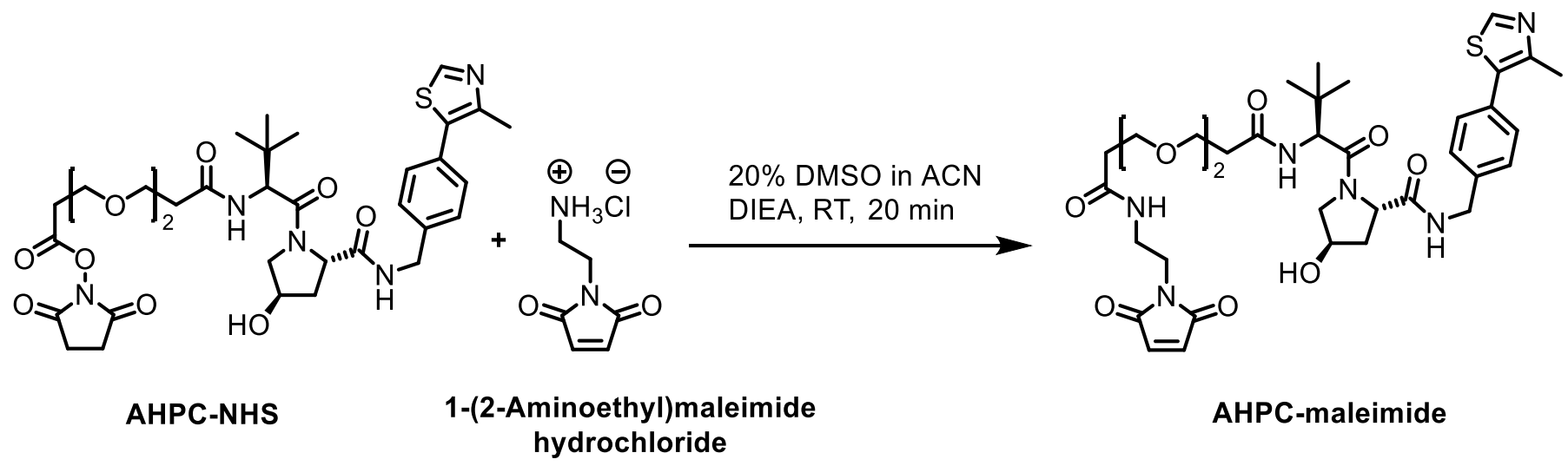

Supplementary Fig. 9 Schematic representation for the synthesis of AHPC-maleimide.

4. Selective masking of peptide segments

4.1 Late-stage masking of P1 peptide

In a 15 mL Falcon tube, **P1** (23 mg, 1.0 equiv., 18.3 µmol, 5.4 mM) was dissolved in 6 M Gun.HCl, 0.2 M Na_2HPO_4 buffer (3.4 mL). To this solution, NB-Br (20.4 mg, 5.2 equiv., 94.5 µmol, 0.25 M; based on **P1**) dissolved in ACN (0.38 mL, v = 10%) was directly added and the pH was adjusted to 7.5 using 5 *N* NaOH. The reaction mixture was then incubated for 1 h at rt. The progress of the reaction was monitored by LC-MS using Method A (Section 1.2). After the completion of the reaction, the reaction mixture was purified using RP-HPLC (Method A described in Section 1.3), affording the final product **P1-N** (63% isolated yield) as a white powder.

A

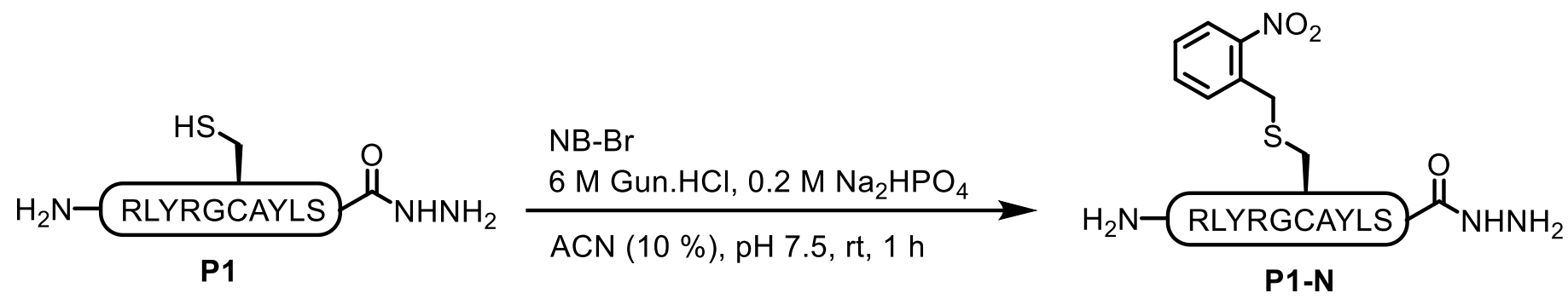

B

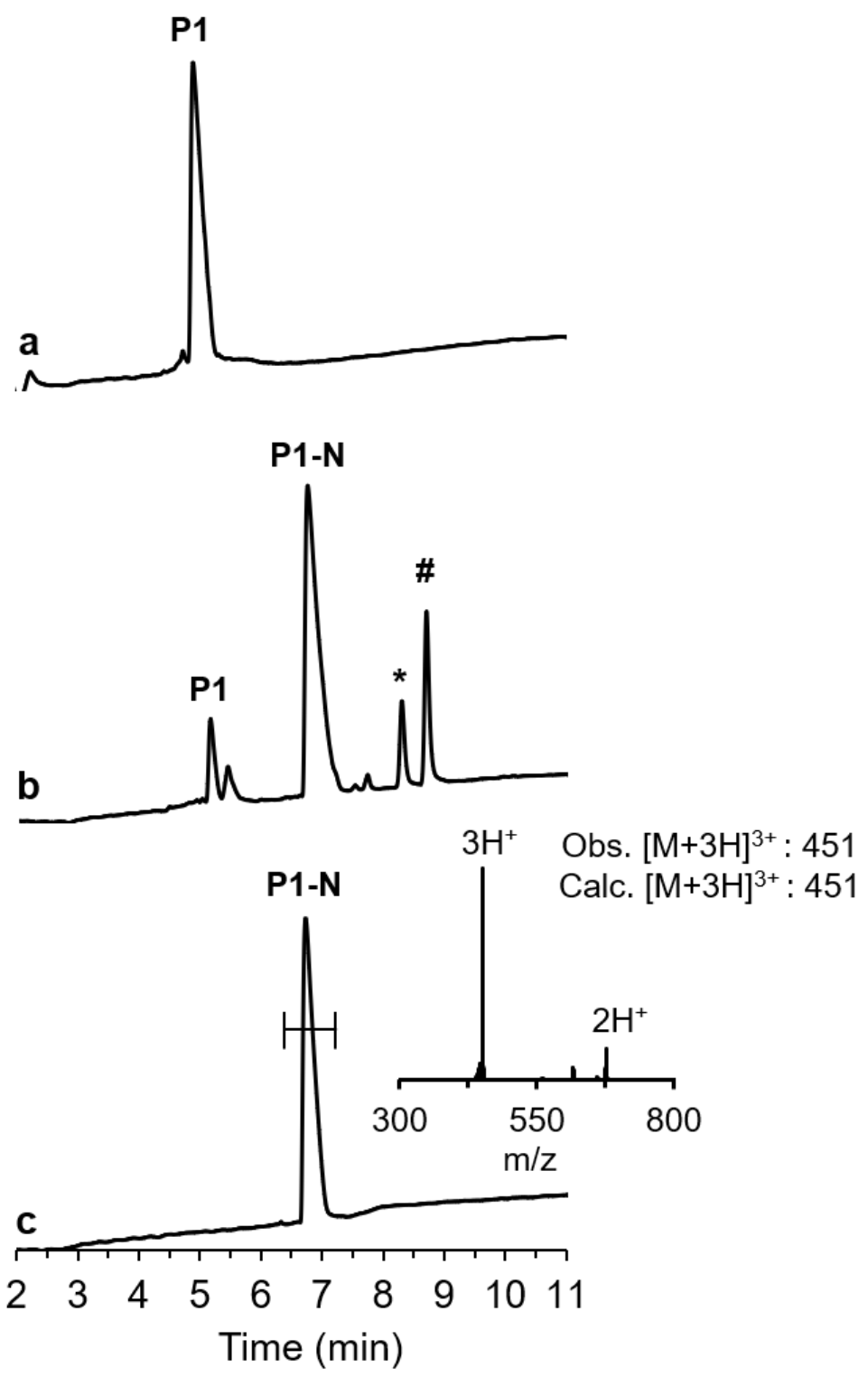

Supplementary Fig. 10 A Schematic representation of late-stage masking of **P1**. **B** (a) LC-MS analysis of **P1** at t = 0. (b) NB-protected **P1** (**P1-N**) at t = 1 h. (c) Isolated **P1-N**. # unreacted NB-Br and * hydrolyzed NB-Br.

4.2 Late-stage masking of P2 peptide

In a 15 mL Falcon tube, **P2** (78 mg, 1.0 equiv., 58.0 µmol, 8.3 mM) was dissolved in 6 M Gun.HCl, 0.2 M Na_2HPO_4 buffer (7.0 mL) followed by addition of TCEP (83.2 mg, 5.0 equiv., 290.0 µmol, 1.4 M; based on **P2**), which was dissolved in 6 M Gun.HCl, 0.2 M Na_2HPO_4 buffer (200 µL). To this solution, the aqueous formaldehyde solution (11.8 µL, 2.5 equiv., 145 µmol, 12.3 M, based on **P2**) was added, then the reaction mixture pH was adjusted to 6.8 using 5 *N* NaOH. The reaction was incubated at 37 °C for 4 h. The reaction was monitored using LC-MS (Method A described in Section 1.2). Then NB-Br (62.6 mg, 5.0 equiv., 290.0 µmol, 0.36 M; based on **P2**) was dissolved in ACN (0.8 mL v = 10%), directly added to the reaction mixture and the pH adjusted to 7.5 using 5 *N* NaOH. The reaction mixture was then incubated for 1 h at rt. The progress of the reaction was monitored by LC-MS using Method A (Section 1.2). After the completion of the reaction, the reaction mixture was purified using RP-HPLC (Method A described in Section 1.3), affording the final product **P2-T-N** (45% isolated yield) as a white powder.

A

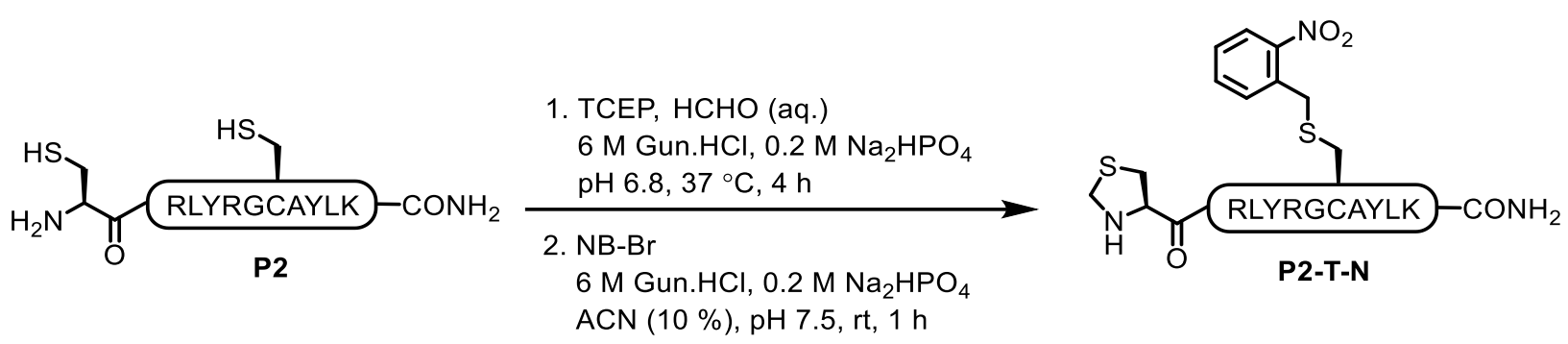

B

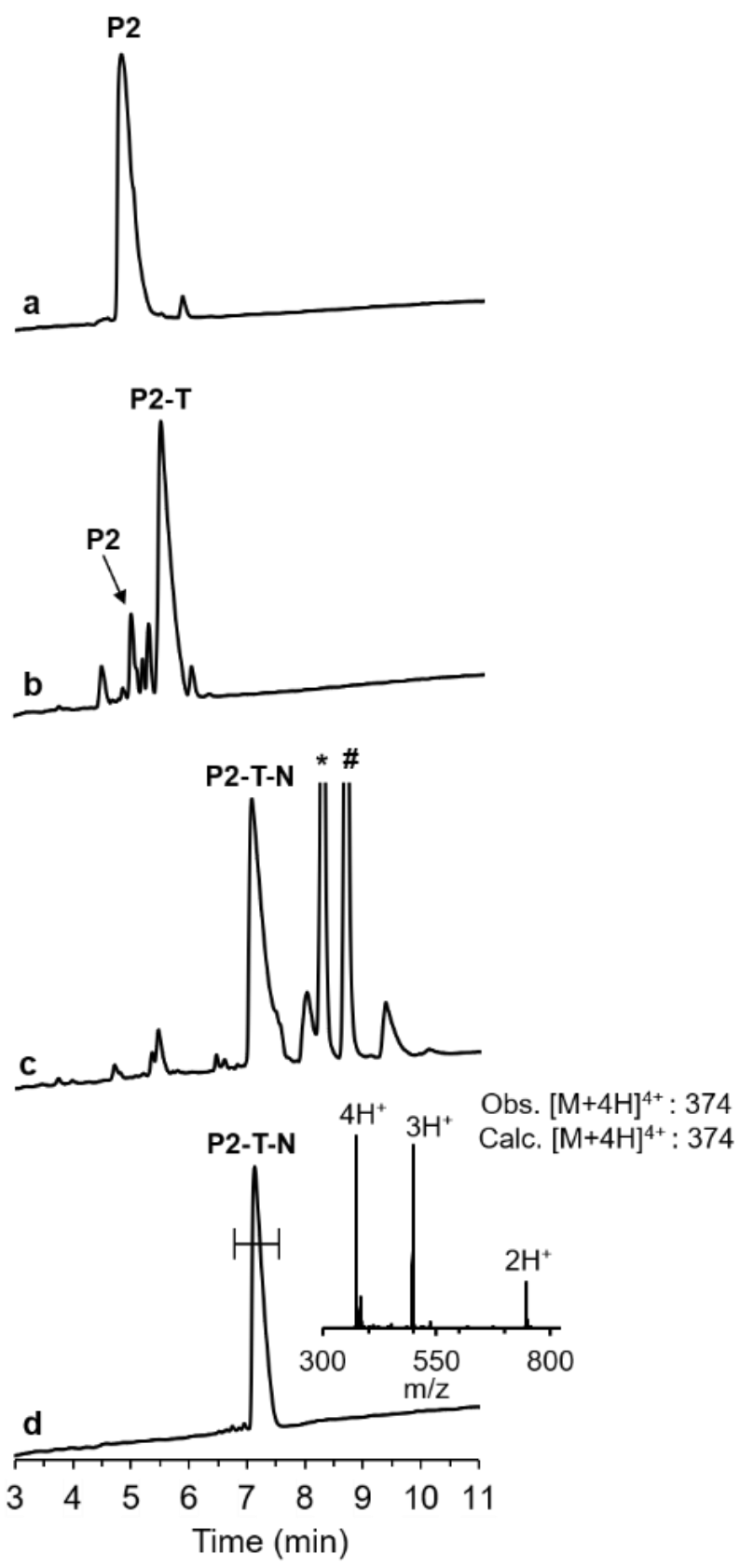

Supplementary Fig. 11 A Schematic representation of late-stage masking of **P2**. **B** (a) LC-MS analysis of **P2** at t = 0. (b) Thz-protected **P2** (**P2-T**) at t = 4 h. (c) Thz- and NB-protected **P2** (**P2-T-N**) at t = 5 h. (d) Isolated **P2-T-N**. # unreacted NB-Br and * hydrolyzed NB-Br.

In a 15 mL Falcon tube, **P2-T-N**, (31.1 mg, 1.0 equiv., 20.8 µmol, 9.1 mM) was dissolved in 6 M Gun.HCl, 0.2 M Na_2HPO_4 buffer (2.3 mL) followed by addition of TCEP (29.8 mg, 5.0 equiv., 104.0 µmol, 0.69 M; based on **P2-T-N**) dissolved in 6 M Gun.HCl, 0.2 M Na_2HPO_4 buffer (150 µL). To this solution, $NH_2OMe.HCl$ (43.5 mg, 25 equiv., 520 µmol, 3.45 M; based on **P2-T-N**) dissolved in 6 M Gun.HCl, 0.2 M Na_2HPO_4 buffer (150 µL) was added, then the pH was adjusted to 4 using 5 *N* HCl. The reaction was incubated at 37 °C for 1 h. The progress of the reaction was monitored by LC-MS using Method A (Section 1.2).[2] After the completion of the reaction, the reaction mixture was purified using RP-HPLC (Method A described in Section 1.3), affording the final product **P2-N** (70% isolated yield) as a white powder.

A

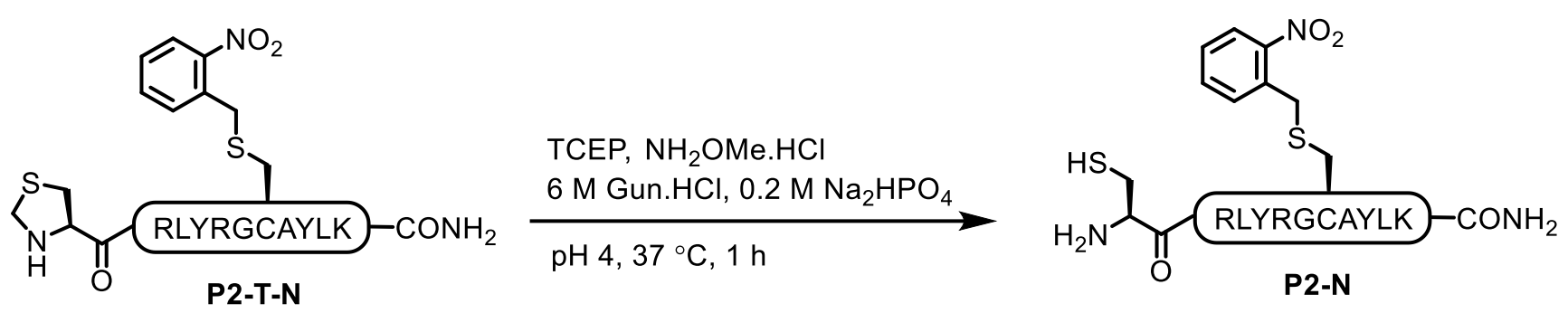

B

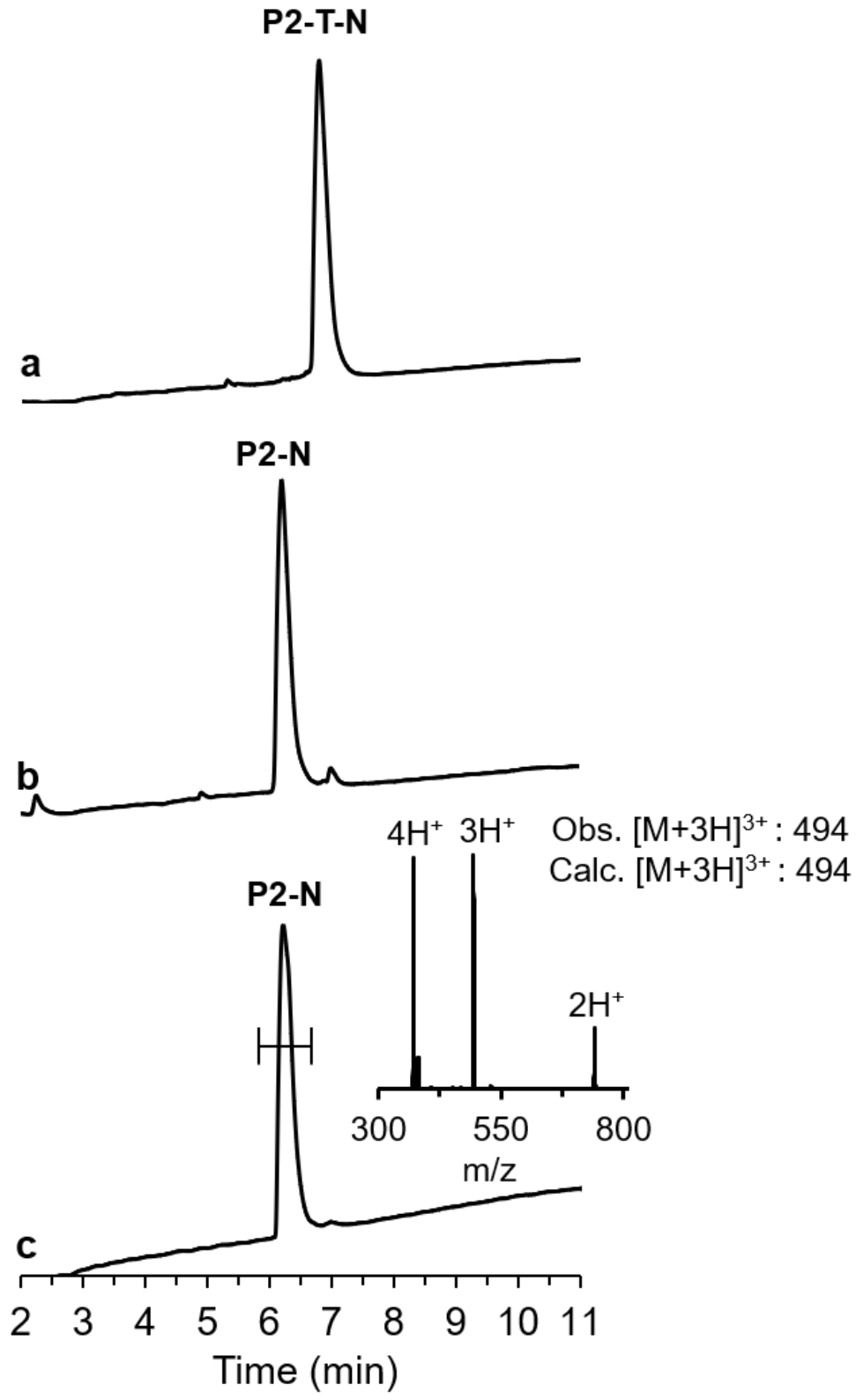

Supplementary Fig. 12 A Schematic representation of late-stage unmasking of **P2-T-N**. **B** (a) LC-MS analysis of **P2-T-N** at t = 0. (b) NB-protected **P2** (**P2-N**) at t = 1 h. (c) Isolated **P2-N**.

4.3 Late-stage masking of ZF1 peptide

In a 15 mL Falcon tube, **ZF1** (33.8 mg, 1.0 equiv., 9.38 µmol, 5.5 mM) was dissolved in 6 M Gun.HCl, 0.2 M Na_2HPO_4 buffer (1.71 mL). To this solution, NB-Br (20.3 mg, 10.0 equiv., 93.8 µmol, 0.5 M; based on **ZF1**) dissolved in ACN (0.19 mL, v = 10%) was added and then the pH was adjusted to 7.5 using 5 *N* NaOH. The reaction mixture was then incubated for 1 h at rt. The progress of the reaction was monitored by LC-MS using Method A (Section 1.2). After the completion of the reaction, the reaction mixture was purified using RP-HPLC (Method B described in Section 1.3), affording the final product **ZF1-N** (45% isolated yield) as a white powder.

A

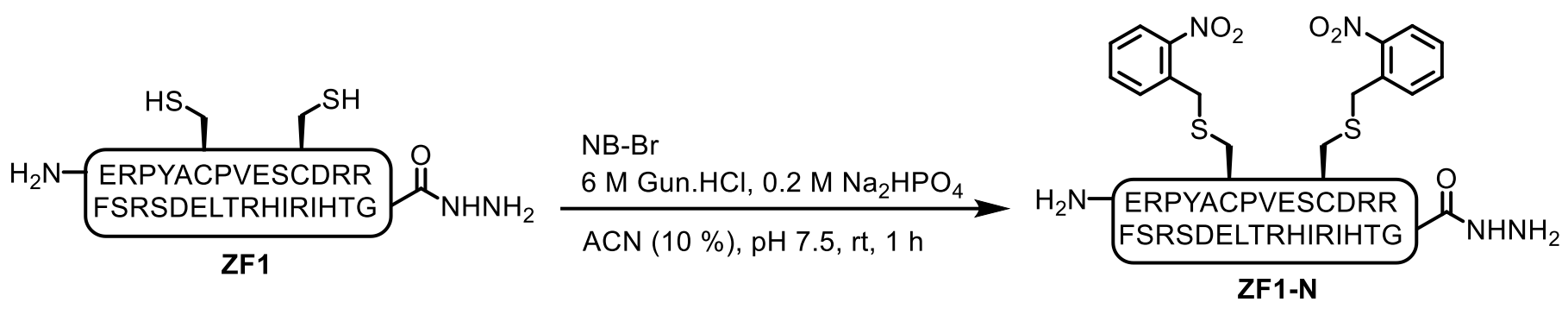

B

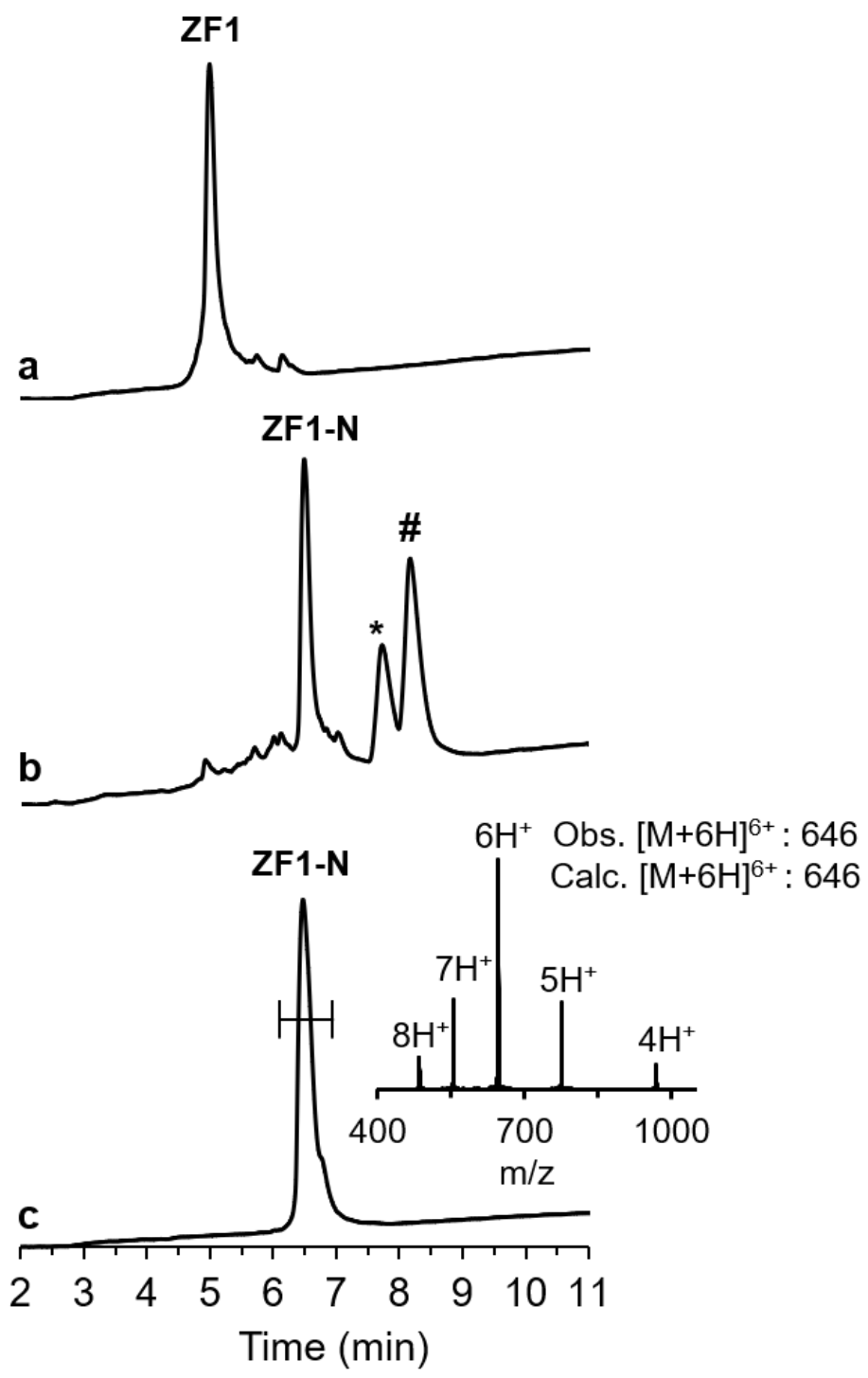

Supplementary Fig. 13 A Schematic representation of late-stage masking of **ZF1**. **B** (a) LC-MS analysis of **ZF1** at t = 0. (b) NB-protected **ZF1** (**ZF1-N**) at t = 1 h. (c) Isolated **ZF1-N**. # unreacted NB-Br and * hydrolyzed NB-Br.

4.4 Late-stage masking of ZF2 peptide

In a 15 mL Falcon tube, **ZF2** (61 mg, 1.0 equiv., 17.0 µmol, 5.6 mM) was dissolved in 6 M Gun.HCl, 0.2 M Na_2HPO_4 buffer (3.0 mL), followed by the addition of TCEP (24.4 mg, 5.0 equiv., 85.0 µmol, 0.85 M, based on **ZF2**) dissolved in 6 M Gun.HCl, 0.2 M Na_2HPO_4 buffer (100 µL). To this solution, the aqueous formaldehyde solution (4.8 µL, 3.5 equiv., 59.5 µmol, 12.3 M; based on **ZF2**) was added and then the pH was adjusted to 6.8 using 5 *N* NaOH. The reaction was incubated at 37 °C for 4 h. The reaction was monitored using LC-MS (Method A described in Section 1.2). Then NB-Br (36.7 mg, 10.0 equiv., 170.0 µmol, 0.5 M; based on **ZF2**) was dissolved in ACN (0.34 mL, v = 10%), added to the reaction mixture and the pH was adjusted to 7.5 using 5 *N* NaOH. The reaction mixture was then incubated for 1 h at rt. The progress of the reaction was monitored by LC-MS using Method A (Section 1.2). After the completion of the reaction, the reaction mixture was purified using RP-HPLC (Method B described in Section 1.3), affording the final product **ZF2-T-N** (49% isolated yield) as a white powder.

A

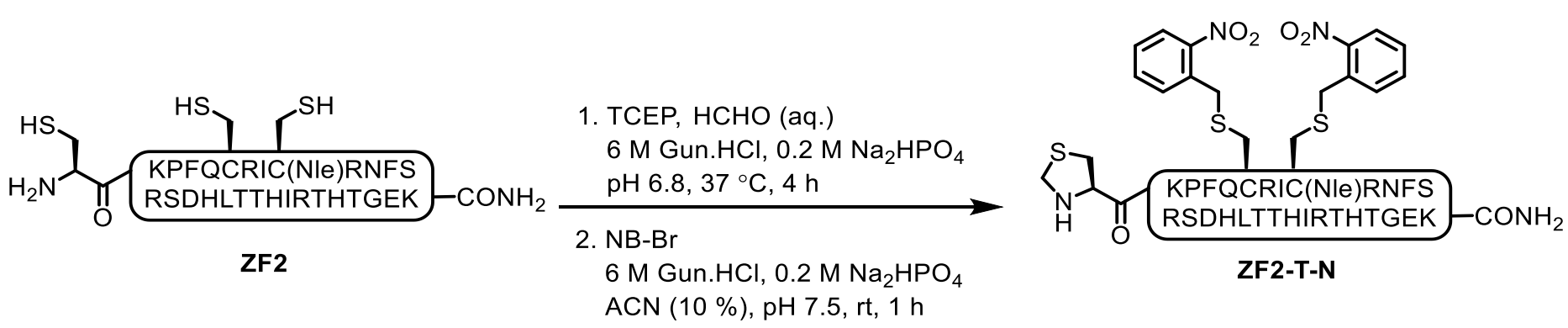

B

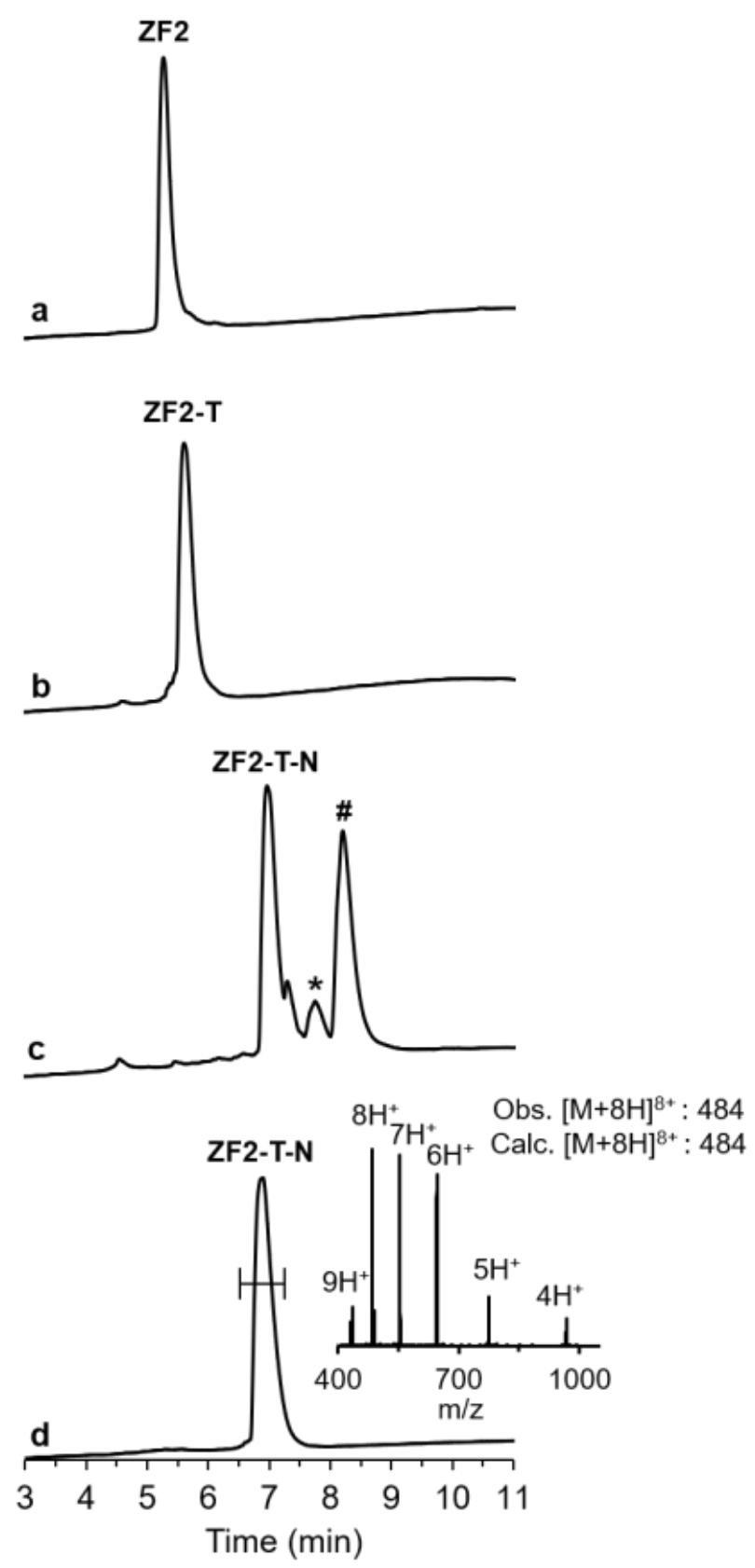

Supplementary Fig. 14 A Schematic representation of late-stage masking of **ZF2**. **B** (a) LC-MS analysis of **ZF2** at t = 0. (b) Thz-protected **ZF2** (**ZF2-T**) at t = 4 h. (c) Thz- and NB-protected **ZF2** (**ZF2-T-N**) at t = 5 h. (d) Isolated **ZF2-T-N**. # unreacted NB-Br and * hydrolyzed NB-Br.

In a 15 mL Falcon tube, **ZF2-T-N** (32 mg, 1.0 equiv., 8.3 µmol, 5.5 mM) was dissolved in 6 M Gun.HCl, 0.2 M Na_2HPO_4 buffer (1.5 mL) followed by the addition of TCEP (11.9 mg, 5.0 equiv., 41.5 µmol, 0.52 M; based on **ZF2-T-N**) dissolved in 6 M Gun.HCl, 0.2 M Na_2HPO_4 buffer (80 µL). To this solution, $NH_2OMe.HCl$ (17.2 mg, 25 equiv., 207.5 µmol, 2.6 M; based on **ZF2-T-N**) dissolved in 6 M Gun.HCl, 0.2 M Na_2HPO_4 buffer (80 µL) was added, then the pH was adjusted to 4 using 5 *N* HCl. The reaction was incubated at 37 °C for 1 h. The progress of the reaction was monitored by LC-MS using Method A (Section 1.2). After the completion of the reaction, the reaction mixture was purified using RP-HPLC (Method B described in Section 1.3), affording the final product **ZF2-N** (65% isolated yield) as a white powder.

A

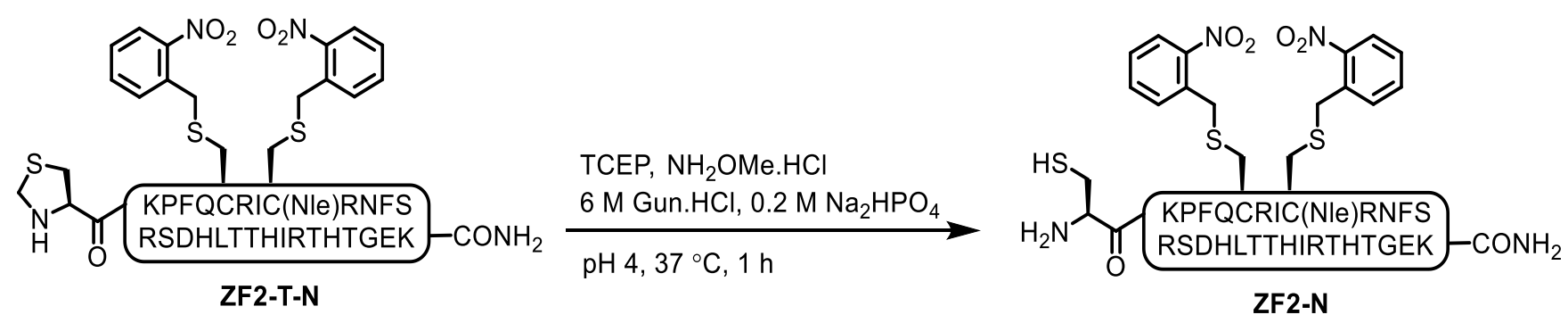

B

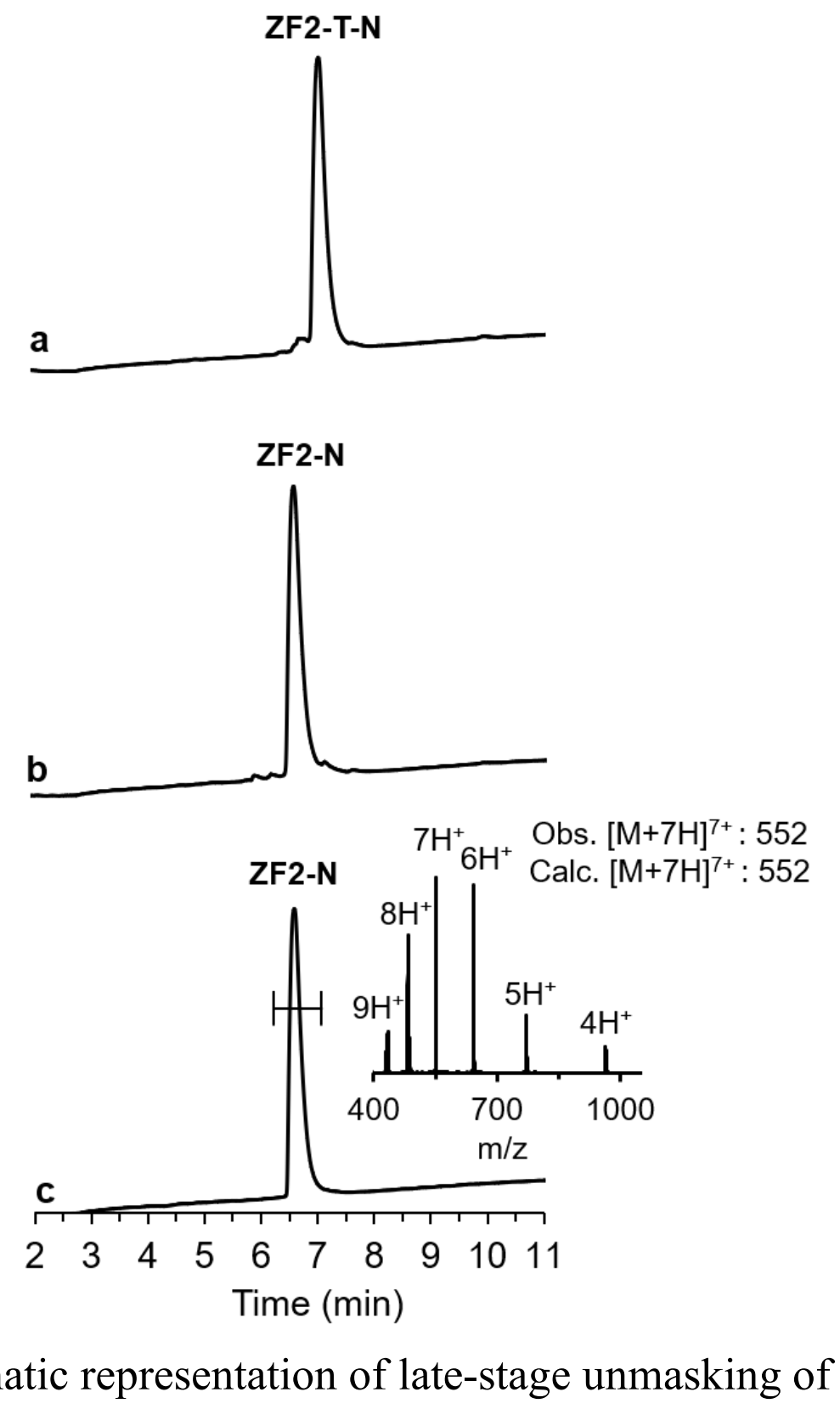

Supplementary Fig. 15 A Schematic representation of late-stage unmasking of **ZF2-T-N**. **B** (a) LC-MS analysis of **ZF2-T-N** at t = 0. (b) NB-protected **ZF2** (**ZF2-N**) at t = 1 h. (c) Isolated **ZF2-N**.

4.5 Late-stage masking of ZF3 peptide

The masking of the **ZF3** was carried out as described in section 4.3, reaction progress monitored by LC-MS using Method B (Section 1.2), affording the final product, **ZF3-N** (50% isolated yield), as a white powder.

A

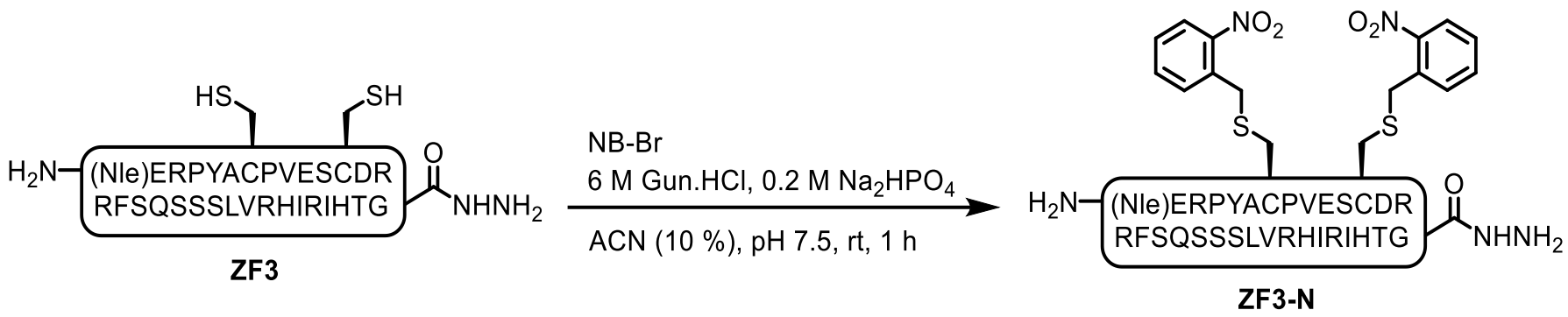

B

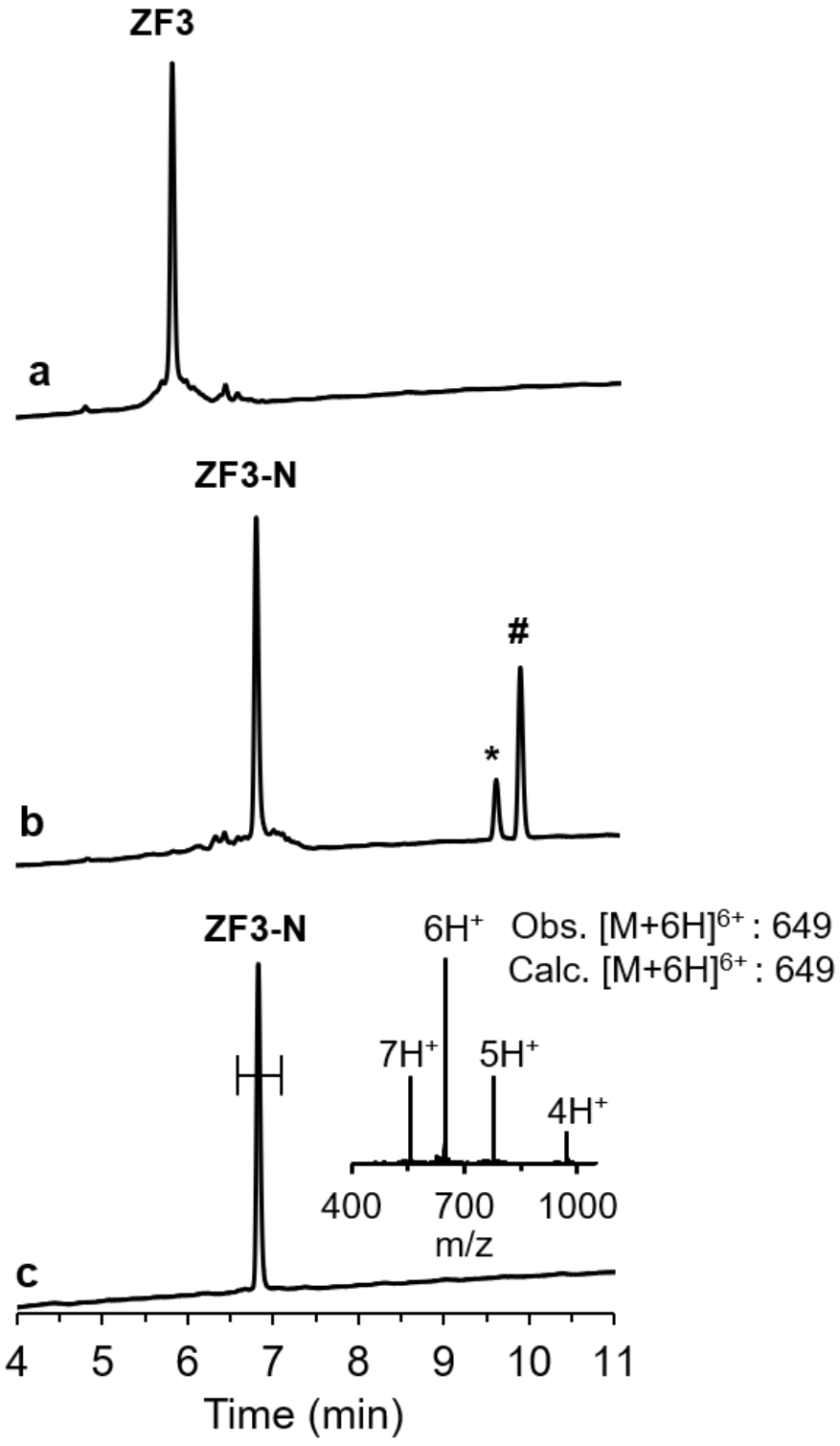

Supplementary Fig. 16 A Schematic representation of late-stage masking of **ZF3**. **B** (a) LC-MS analysis of **ZF3** at t = 0. (b) NB-protected **ZF3** (**ZF3-N**) at t = 1 h. (c) Isolated **ZF3-N**. # unreacted NB-Br and * hydrolyzed NB-Br.

4.6 Late-stage masking of ZF4 peptide

The masking of the **ZF4** was carried out as described in section 4.4, reaction progress monitored by LC-MS using Method B (Section 1.2), affording the final product, **ZF4-T-N** (49% isolated yield), as a white powder.

A

B

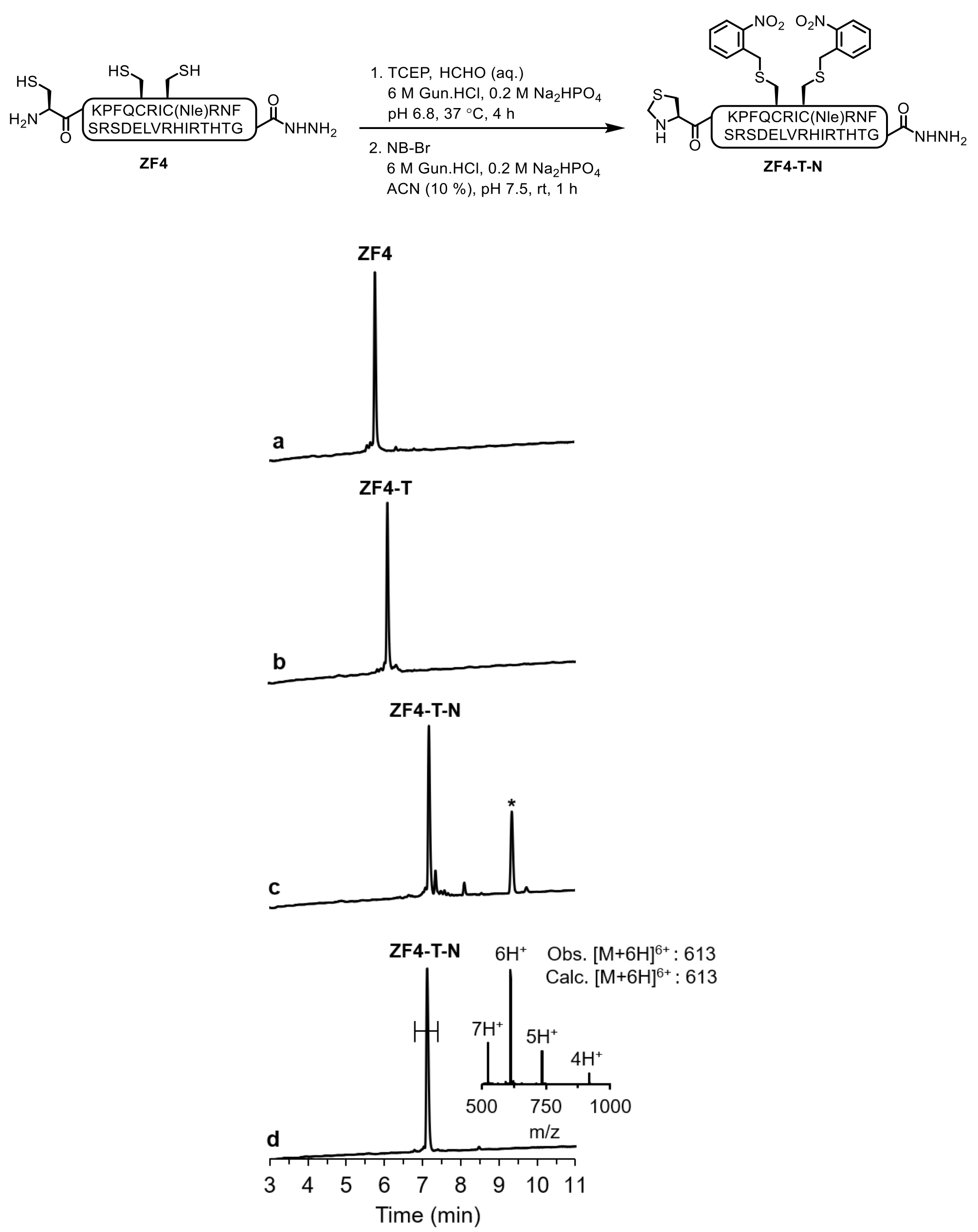

Supplementary Fig. 17 A Schematic representation of late-stage masking of **ZF4**. **B** (a) LC-MS analysis of **ZF4** at t = 0. (b) Thz-protected **ZF4** (**ZF4-T**) at t = 4 h. (c) Thz- and NB-protected **ZF4** (**ZF4-T-N**) at t = 5 h. (d) Isolated **ZF4-T-N**. * hydrolyzed NB-Br.

The synthesis of **ZF4-N** was carried out as described in section 4.4, with reaction progress monitored by LC-MS using Method B (Section 1.2), affording the final product, **ZF4-N** (70% isolated yield), as a white powder.

A

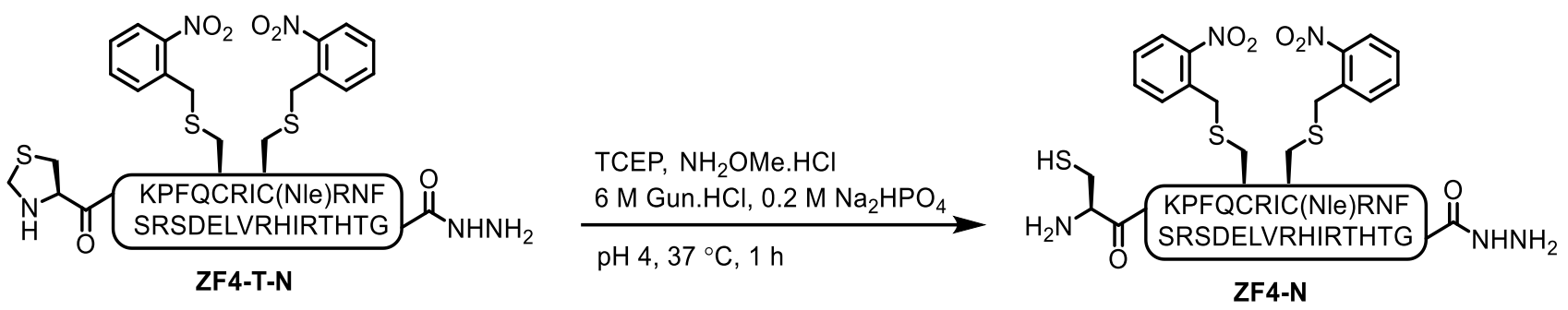

B

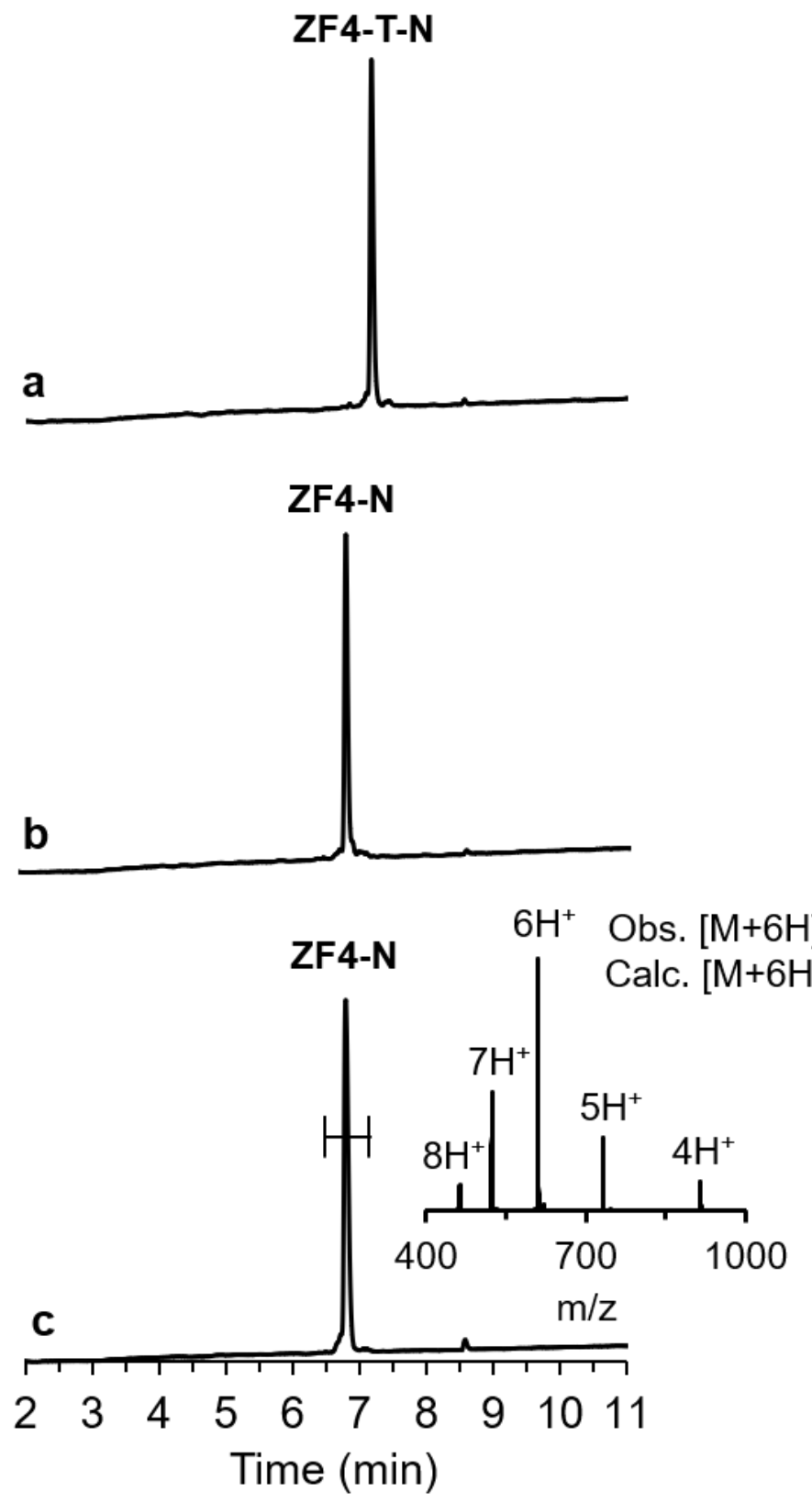

Supplementary Fig. 18 A Schematic representation of late-stage unmasking of **ZF4-T-N**. **B** (a) LC-MS analysis of **ZF4-T-N** at t = 0. (b) NB-protected **ZF4** (**ZF4-N**) at t = 1 h. (c) Isolated **ZF4-N**.

4.7 Late-stage masking of ZF5 peptide

The masking of the **ZF5** was carried out as described in section 4.4, reaction progress monitored by LC-MS using Method B (Section 1.2), affording the final product, **ZF5-T-N** (50% isolated yield), as a white powder.

A

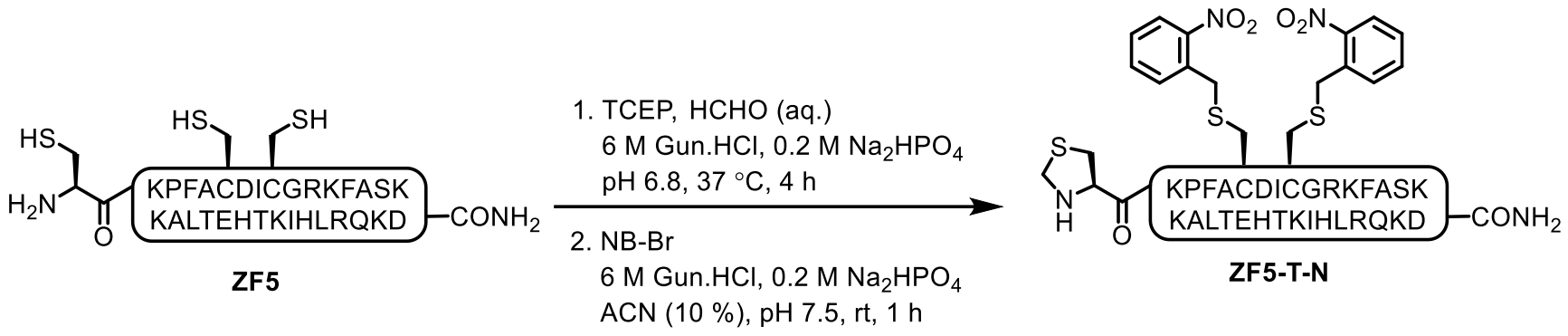

B

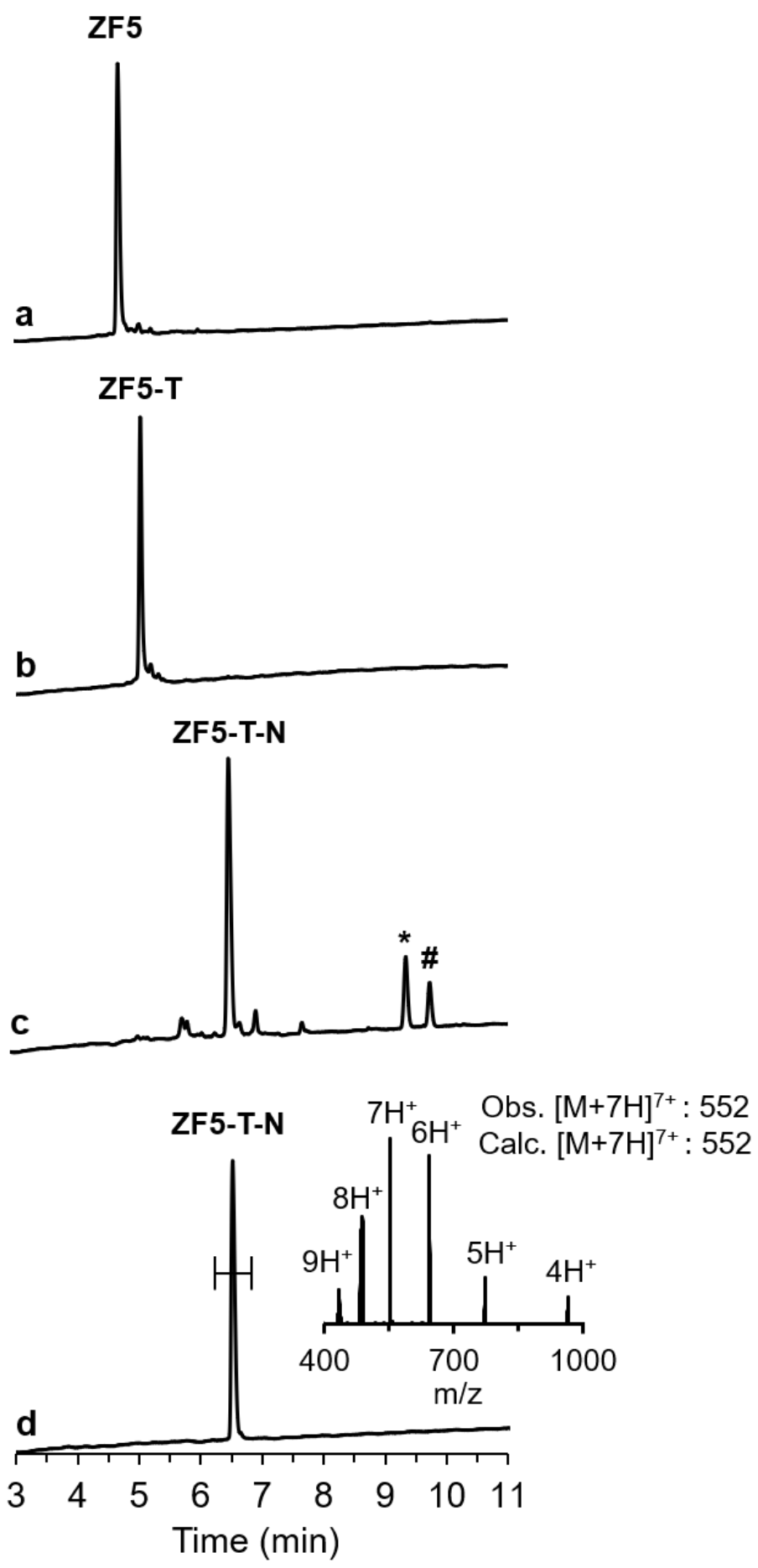

Supplementary Fig. 19 A Schematic representation of late-stage masking of **ZF5**. **B** (a) LC-MS analysis of **ZF5** at t = 0. (b) Thz-protected **ZF5** (**ZF5-T**) at t = 4 h. (c) Thz- and NB-protected **ZF5** (**ZF5-T-N**) at t = 5 h. (d) Isolated **ZF5-T-N**. # unreacted NB-Br and * hydrolyzed NB-Br.

The synthesis of the **ZF5-N** was carried out as described in section 4.4, reaction progress monitored by LC-MS using Method B (Section 1.2), affording the final product, **ZF5-N** (84% isolated yield), as a white powder.

A

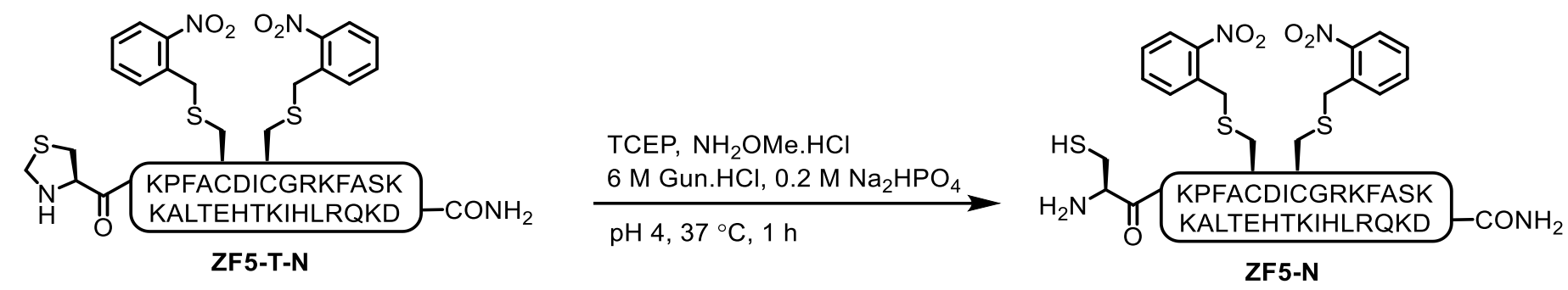

B

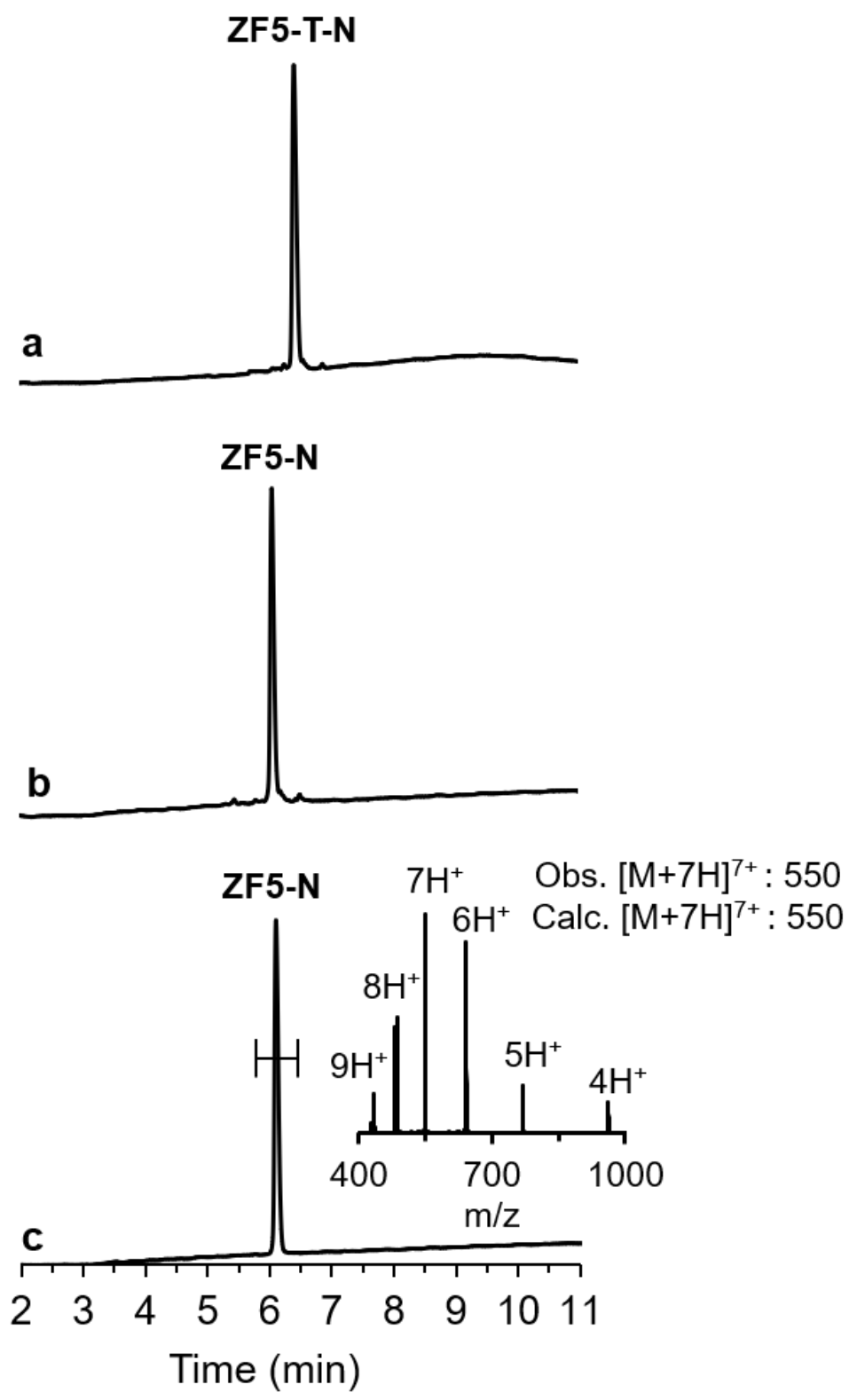

Supplementary Fig. 20 A Schematic representation of late-stage unmasking of **ZF5-T-N**. **B** (a) LC-MS analysis of **ZF5-T-N** at t = 0. (b) NB-protected **ZF5** (**ZF5-N**) at t = 1 h. (c) Isolated **ZF5-N**.

5. Chemoselective peptide ligation

5.1 Synthesis of peptide P-N

In a 15 mL Falcon tube, **P1-N** (17.8 mg, 1.0 equiv., 13.1 µmol, 10.1 mM) was dissolved in 6 M Gun.HCl, 0.2 M Na_2HPO_4 buffer (1.3 mL) at pH 3.0 and cooled to -15 °C by placing in an ice/salt bath. 50 µL of $NaNO_2$ (9.0 mg, 10 equiv., 131.0 µmol, 2.62 M; based on **P1-N**), dissolved in water, was added to the reaction mixture and allowed to react for 15 min at -15 °C with gentle mixing at 5 min intervals. After 15 min, 1.3 mL of MPAA (109.3 mg, 50 equiv., 0.65 mmol, 0.5 M; based on **P1-N**) in 6 M Gun.HCl, 0.2 M Na_2HPO_4 buffer at pH 6.8 was added to the mixture and gently mixed for three min. Then, **P2-N** (19.4 mg, 1.0 equiv., 13.1 µmol, 5.0 mM) was added to the reaction mixture and the pH was adjusted to 7.2 using 5 *N* NaOH. The mixture was then incubated for 1 h at rt and then 1.3 mL of TCEP (149 mg, 40 equiv., 0.52 mmol, 0.4 M; based on **P1-N**) in 6 M Gun.HCl, 0.2 M Na_2HPO_4 buffer at pH 7.2 was added and continued incubating for 15 min at rt. The reaction was monitored using LC-MS (Method A described in Section 1.2).[3,4] After the completion of the reaction, the reaction mixture was purified using RP-HPLC (Method A described in Section 1.3), affording the final product **P-N** (34% isolated yield) as a white powder.

A

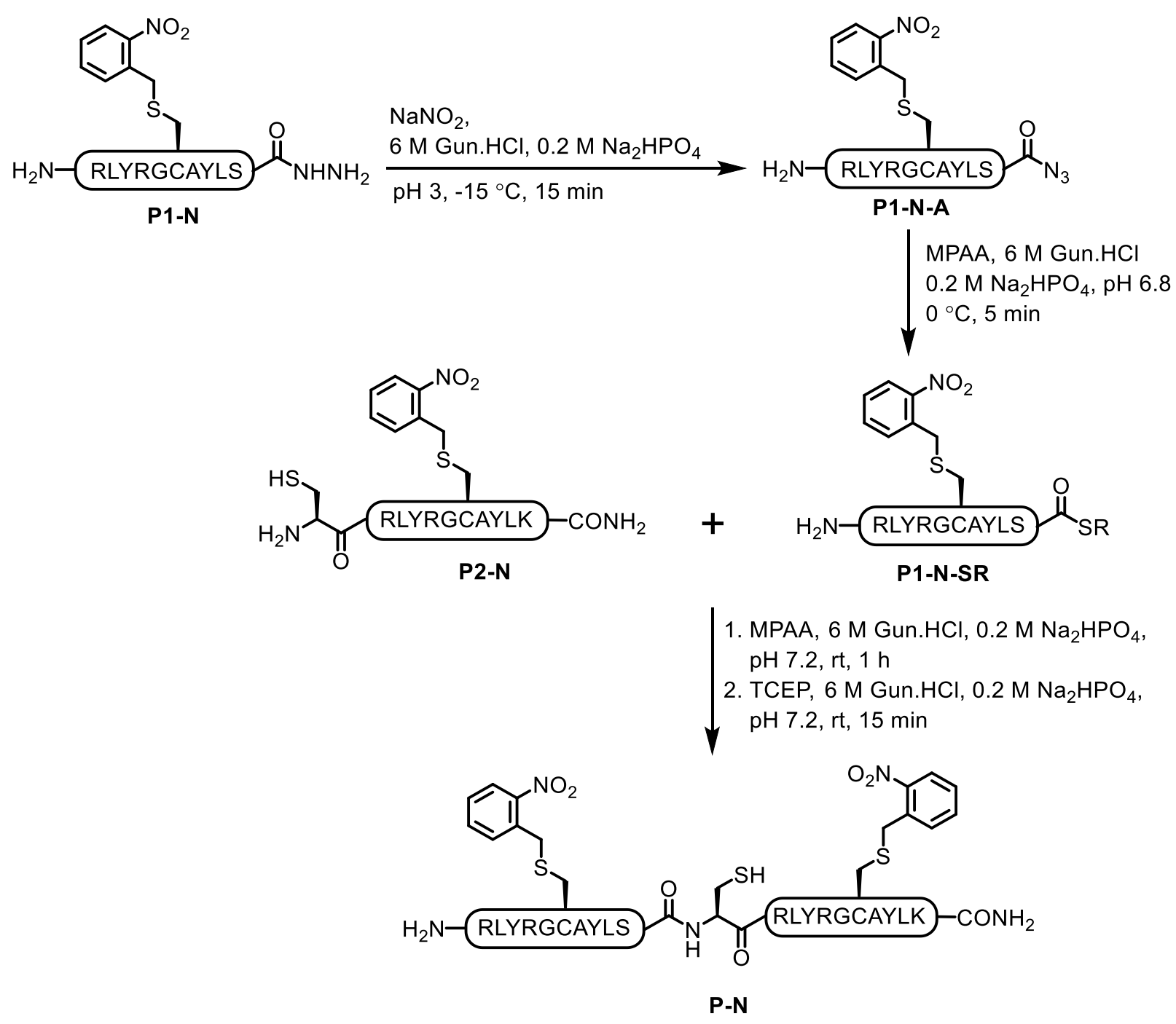

B

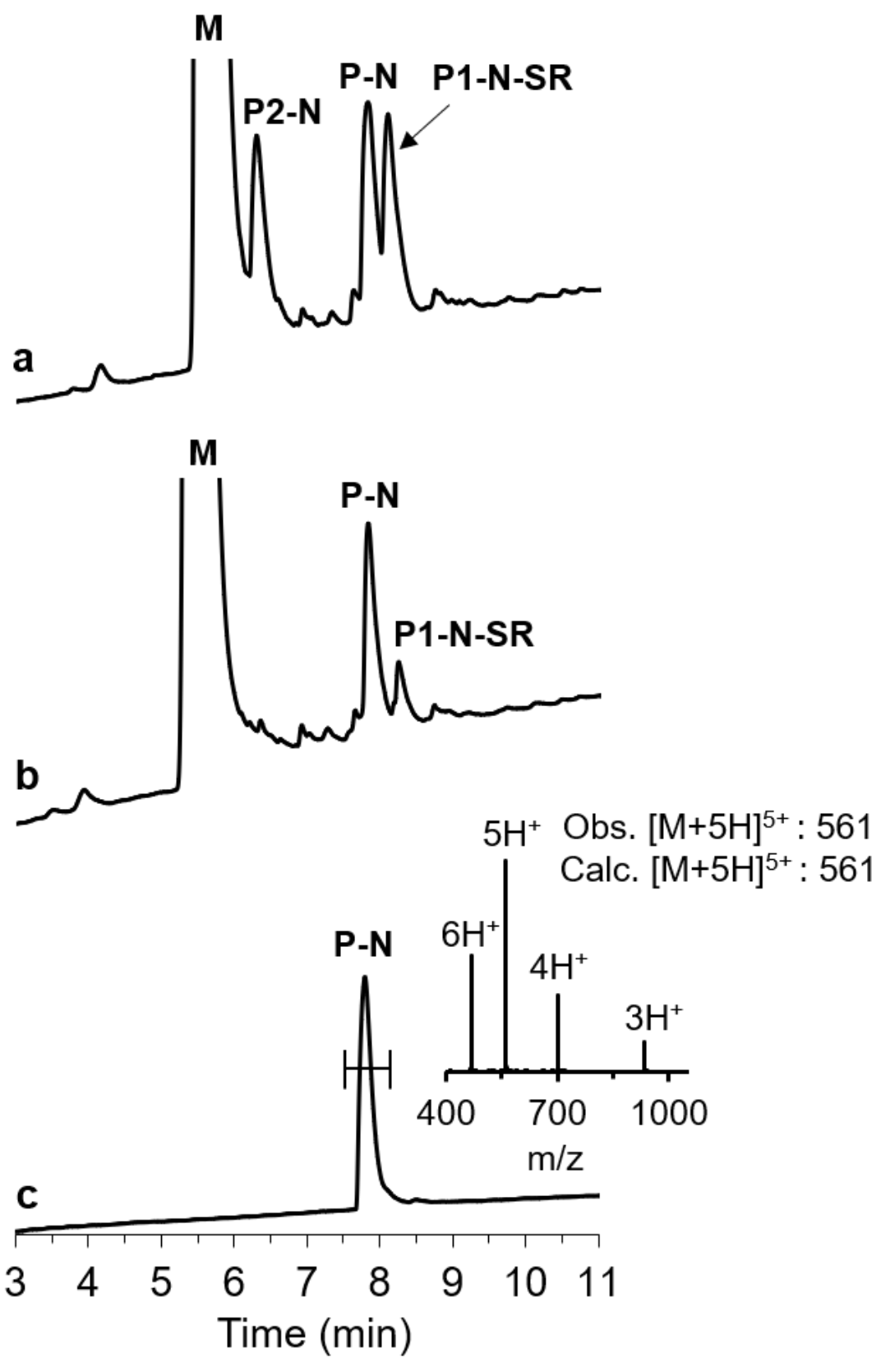

Supplementary Fig. 21 **A** Schematic representation of synthesis of **P-N** peptide. **B** LC-MS analysis of **P-N** synthesis via NCL. (a) Ligation at t = 0; thioester of **P1-N** with MPAA (**P1-N-SR**), Cys-segment (**P2-N**), ligated product (**P-N**), and M = MPAA. (b) Ligation at t = 1 h. (c) Isolated ligated product **P-N**.

5.2 Synthesis of ZF-N protein

In a 15 mL Falcon tube, **ZF1-N** (10.4 mg, 1.0 equiv., 2.69 µmol, 3.8 mM) was dissolved in 6 M Gun.HCl, 0.2 M Na_2HPO_4 buffer (0.7 mL) at pH 3.0 and cooled to -15 °C by placing in an ice/salt bath. 25 µL of $NaNO_2$ (1.85 mg, 10 equiv., 26.9 µmol, 1.1 M; based on **ZF1-N**), which was dissolved in water, was added to the reaction mixture and allowed to react for 15 min at -15 °C with gentle mixing in repeated intervals. After 15 min, 0.7 mL of MPAA (22.6 mg, 50 equiv., 134.5 µmol, 0.19 M; based on **ZF1-N**) in 6 M Gun.HCl, 0.2 M Na_2HPO_4 buffer at pH 6.8 was added to the mixture and gently mixed for three min. Then, **ZF2-N** (11.4 mg, 1.1 equiv., 2.96 µmol, 2.1 mM) was added to the reaction mixture and the pH was adjusted to 7.2 using 5 *N* NaOH. The mixture was incubated for 1 h at rt and then 0.7 mL of TCEP (30.8 mg, 40 equiv., 107.6 µmol, 0.14 M; based on **ZF1-N**) in 6 M Gun.HCl, 0.2 M Na_2HPO_4 buffer at pH 7.2 was added and continued incubating for 15 min at rt. The reaction was monitored using LC-MS (Method A described in Section 1.2). After the completion of the reaction, the reaction mixture was purified using RP-HPLC (Method A described in Section 1.3), affording the final product **ZF-N** (57% isolated yield) as a white powder.

A

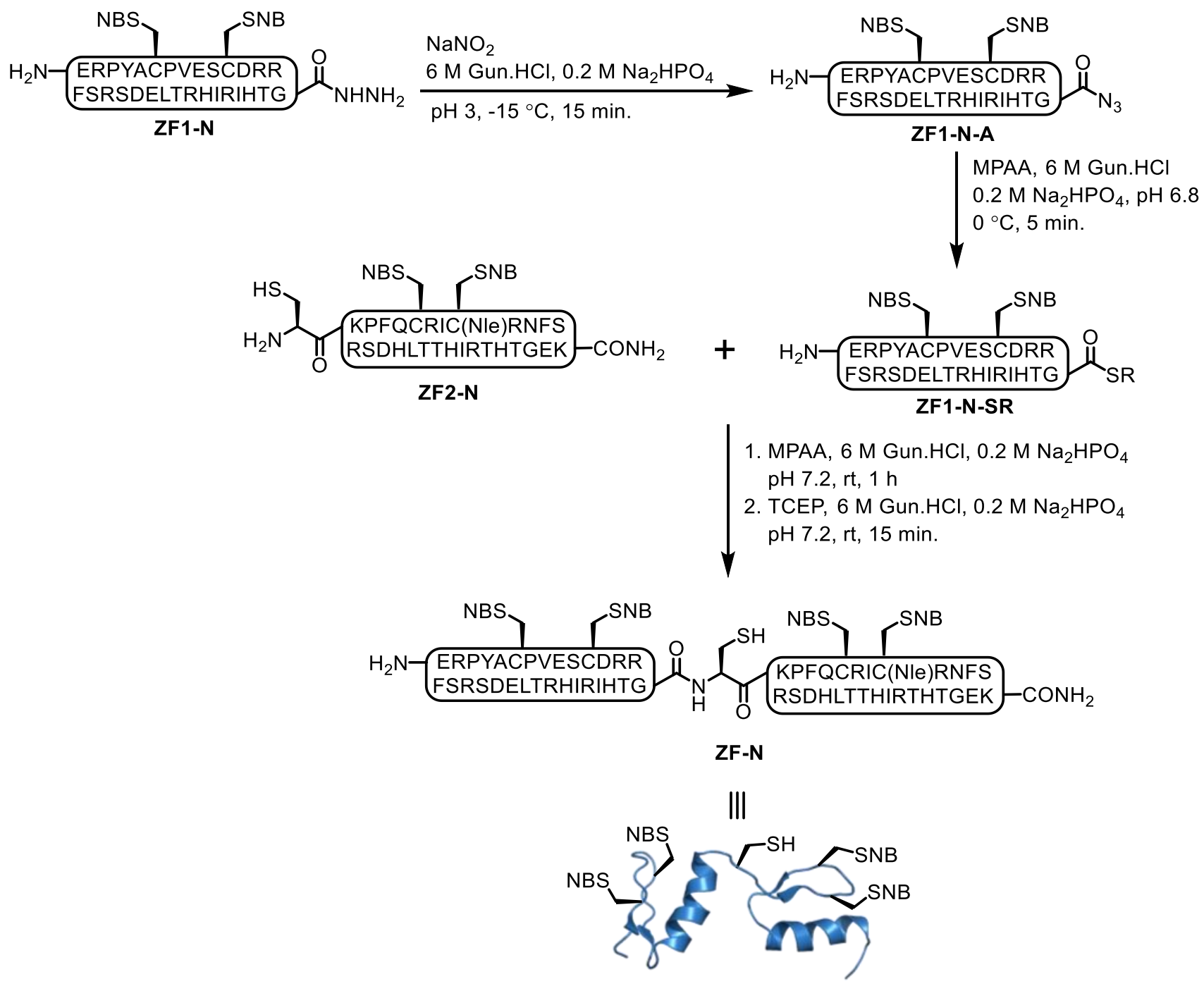

B

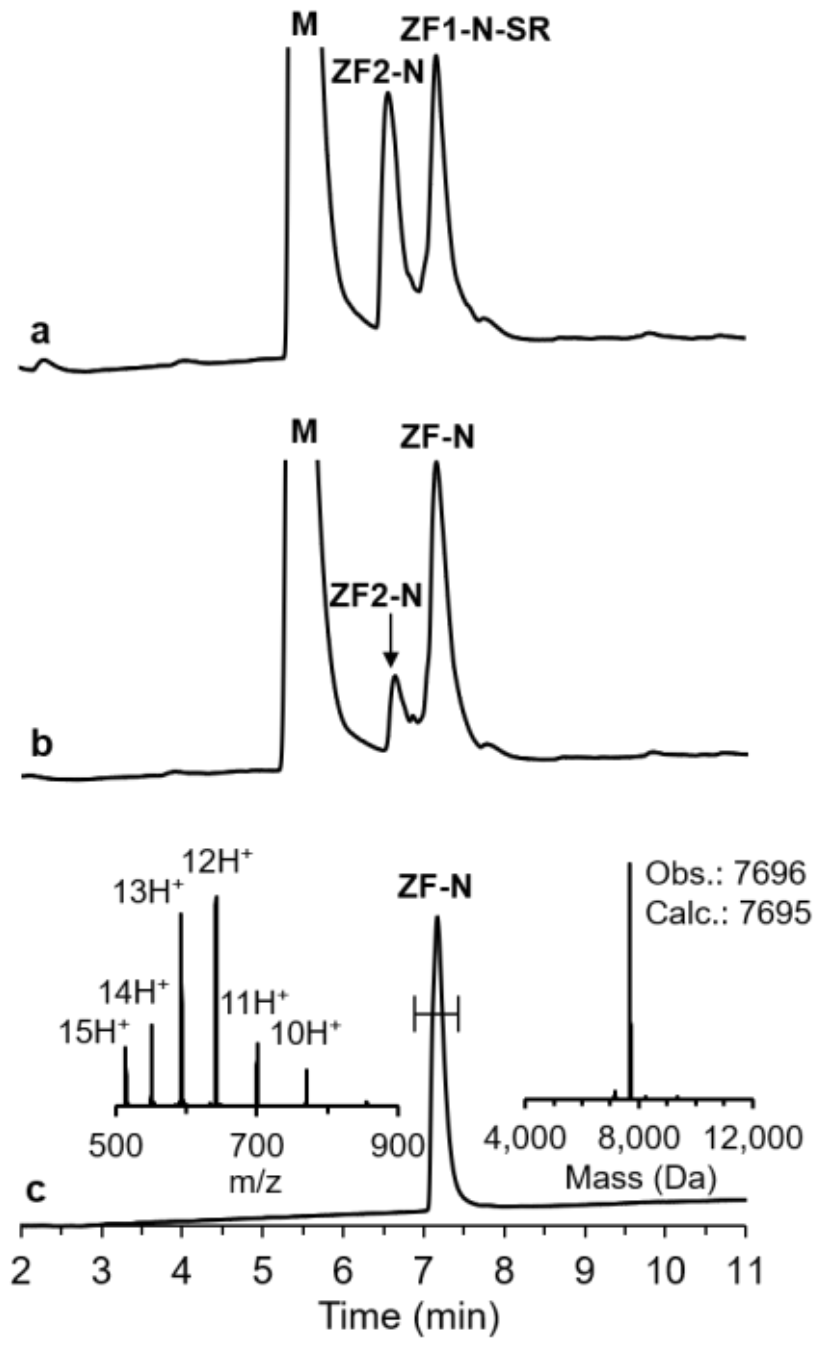

Supplementary Fig. 22 **A** Schematic representation of synthesis of **ZF-N** protein. **B** LC-MS analysis of **ZF-N** protein synthesis via NCL. (a) Ligation at t = 0; thioester of **ZF1-N** with MPAA (**ZF1-N-SR)**, Cys-segment (**ZF2-N**), and M = MPAA. (b) Ligation at t = 1 h. (c) Isolated ligated product **ZF-N**.

5.3 One-pot synthesis of eZF-TF-N

Segment **ZF3-N** (8.8 mg, 1.1 equiv., 2.26 µmol, 9 mM) was dissolved in 6 M Gun.HCl, 0.2 M Na_2HPO_4 buffer (250 µL) at pH 3.0 and cooled to -15 °C by placing in an ice/salt bath. 10 µL of $NaNO_2$ (10 equiv., 22.6 µmol, 2.26 M; based on **ZF3-N**) dissolved in water was added to the reaction mixture and allowed to react for 15 min at -15 °C with gentle mixing in repeated intervals. After 15 min, 250 µL of MPAA (50 equiv., 113 µmol, 0.45 M; based on **ZF3-N**) in 6 M Gun.HCl, 0.2 M Na_2HPO_4 buffer at pH 6.8 was added to the mixture and gently mixed for three min. Then, **ZF4-N** (7.5 mg, 1.0 equiv., 2 µmol, 4 mM) was added to the reaction mixture and the pH was adjusted to 6.8 using 5 N NaOH. The mixture was then incubated for 1 h at 25 °C. The reaction was monitored using LC-MS (Method B described in Section 1.2). After 1 h of ligation, the reaction mixture was desalted by pipetting the reaction mixture into a 3 kDa molecular weight cutoff spin filter (Amicon® Ultra-2mL, 3K). The reaction mixture was diluted with a 6 M Gun.HCl, 0.2 M Na_2HPO_4 buffer (pH 7.2) to 2.0 mL and concentrated to 0.5 mL by centrifuging the spin filter at 5000 rpm for 30 min. This dilution and concentration process was repeated three times until all MPAA residuals were removed. Then the mixture was diluted with a 6 M Gun.HCl, 0.2 M Na_2HPO_4 buffer (pH 7.2) to 1.0 mL, the reaction mixture was collected by reverse centrifuge and the pH was adjusted to 3 using 5 N HCl. The reaction mixture was then cooled to -15 °C by placing it in an ice/salt bath. 20 µL of $NaNO_2$ (20 equiv., 40 µmol, 2 M; based on **ZF4-N**) was dissolved in water, added to the reaction mixture, and allowed to react for 15 min at -15 °C with gentle mixing at 5 min intervals. After 15 min, 200 µL of MPAA (100 equiv., 200 µmol, 1 M; based on **ZF4-N**) in 6 M Gun.HCl, 0.2 M Na_2HPO_4 buffer at pH 6.8 was added to the mixture and gently mixed for three min. The **ZF5-N** (7.7 mg, 1 equiv., 2 µmol, 1.6 mM; based on **ZF4-N**) was added to the reaction mixture and the pH was adjusted to 6.8 using 5 N NaOH. The mixture was then incubated for 1 h at 25 °C and then 250 µL of TCEP (80 equiv., 160 µmol, 0.64 M; based on **ZF4-N**) in 6 M Gun.HCl, 0.2 M Na_2HPO_4 buffer at pH 6.8 was added and continued incubating for 30 min at 25 °C. The reaction was monitored using LC-MS (Method B described in Section 1.2). The ligation was completed in 1 h. After the completion of the reaction, purification was carried out using RP-HPLC (Method B described in Section 1.3), affording the final product **eZF-TF-N** as a white powder (40% isolated yield, based on limiting **ZF4-N**).

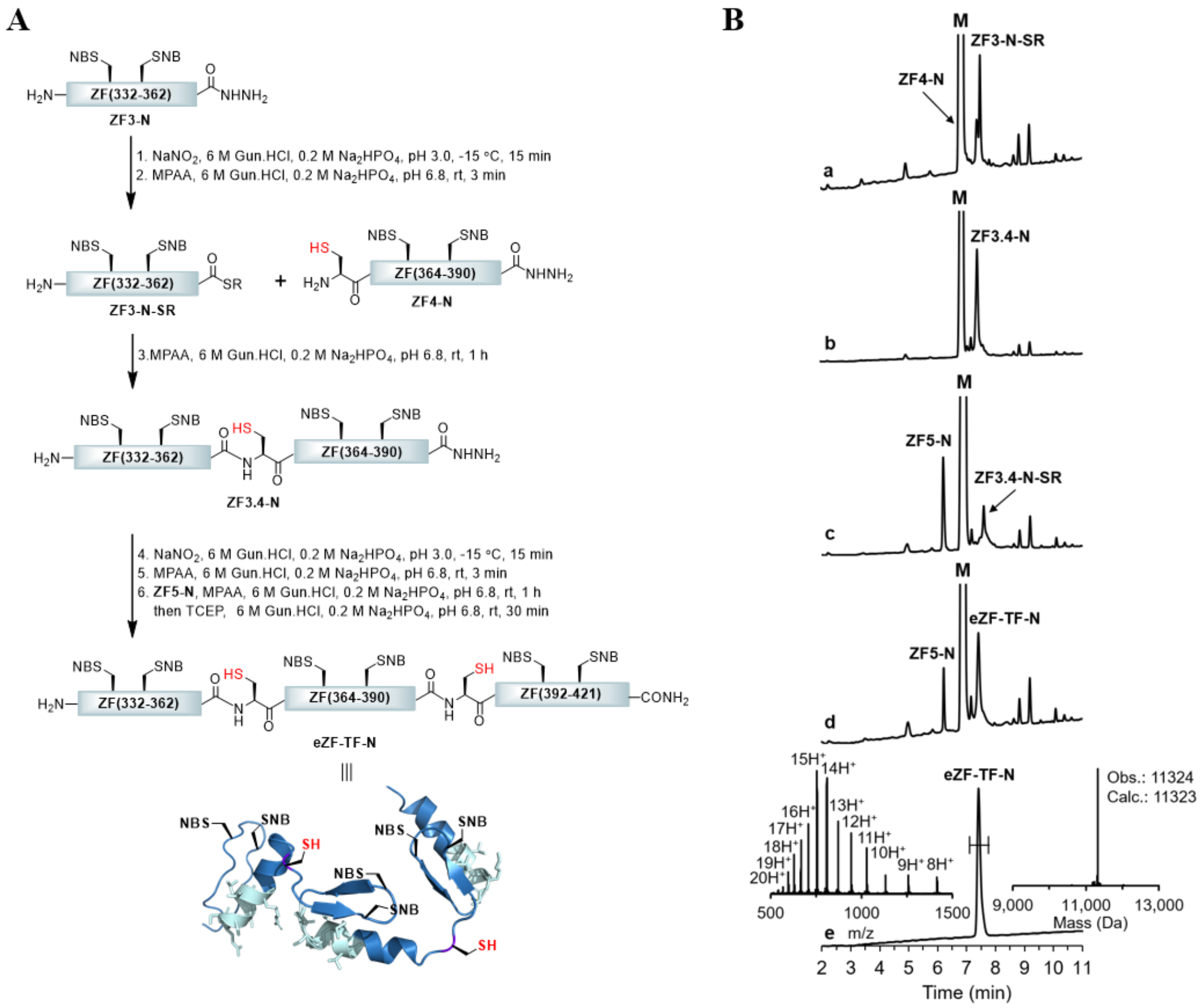

Supplementary Fig. 23 A Schematic representation of synthesis of **eZF-TF-N** protein. **B** LC-MS analysis of **eZF-TF-N** protein synthesis via NCL. (a) First ligation at t = 0; thioester of **ZF3-N** with MPAA (**ZF3-N-SR**), Cys-segment (**ZF4-N**), and M = MPAA. (b) First ligation at t = 1 h. (c) Second ligation at t = 0; thioester of **ZF3.4-N** with MPAA (**ZF3.4-N-SR**) and Cys-segment (**ZF5-N**). (d) Second ligation at t = 1 h; ligated product **eZF-TF-N**. (e) Isolated ligated product **eZF-TF-N**.

5.4 Synthesis of eZF-TFwt

The fragments **WT1** and **WT2** were synthesized similarly to the procedures described in sections 3.5 and 3.6, respectively. The synthesis of **eZF-TFwt** was carried out using NCL, following a procedure similar to that described in section 5.2, affording the final product **eZF-TFwt** (20% isolated yield), as a white powder.

A

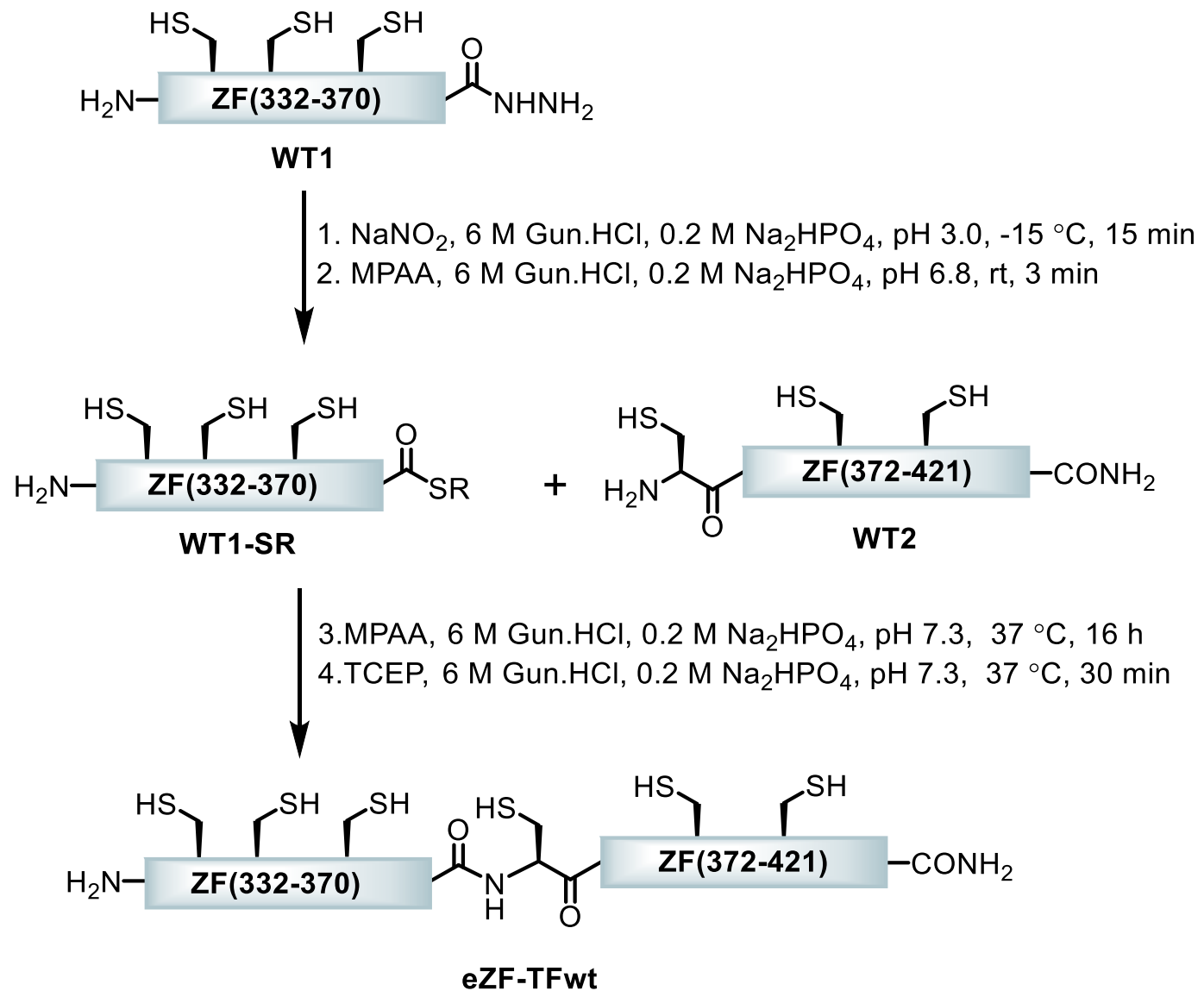

B

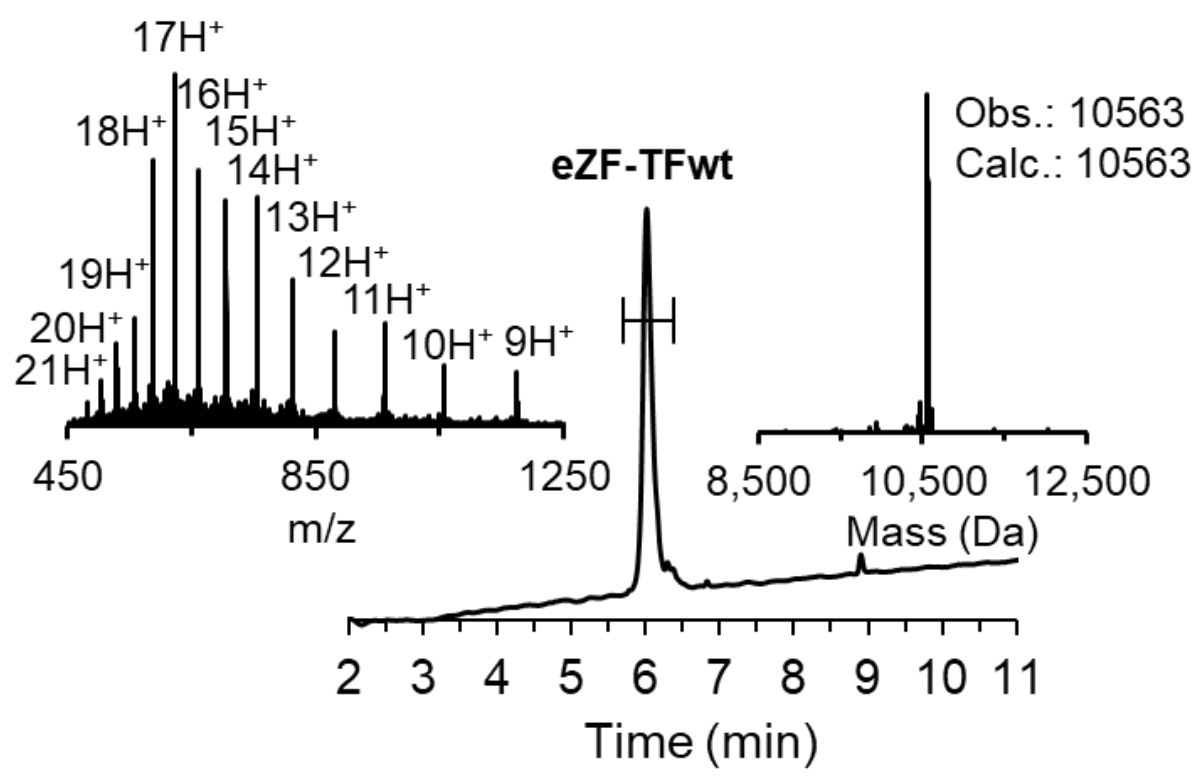

Supplementary Fig. 24 A Schematic representation for the synthesis of **eZF-TFwt** protein. **B** LC-MS analysis of isolated **eZF-TFwt** protein.

6. Site-specific modification of model peptide P

6.1 Site-specific insertion of phospho-Tyr to P-N peptide

In a 15 mL Falcon tube, **P-N** (6 mg, 1.0 equiv., 2.14 µmol, 0.55 mM) was dissolved in 6 M Gun.HCl, 0.2 M Na_2HPO_4 buffer (3.9 mL, pH 7.5). To this solution, Pd(II)OAC-1 (5.14 mg, 2.5 equiv., 5.35 µmol, 12.4 mM; based on **P-N**) dissolved in DMF (0.43 mL, 10% v/v) was added. The reaction mixture was vigorously mixed for a few seconds and then incubated at 37 °C for 15 min. The reaction progress was monitored by LC–MS (Method A, Section 1.2). After confirming completion of the reaction, the mixture was quenched with 3-MPA (4.6 µL, 53.5 µmol, 10 equiv. relative to Pd(II)OAC-1) and incubated at 37 °C for an additional 15 min.[5,6] The reaction mixture was then centrifuged, and the product was purified by RP-HPLC (Method C, Section 1.3) to afford the final product **P-N-M1** (38% isolated yield) as a white powder.

A

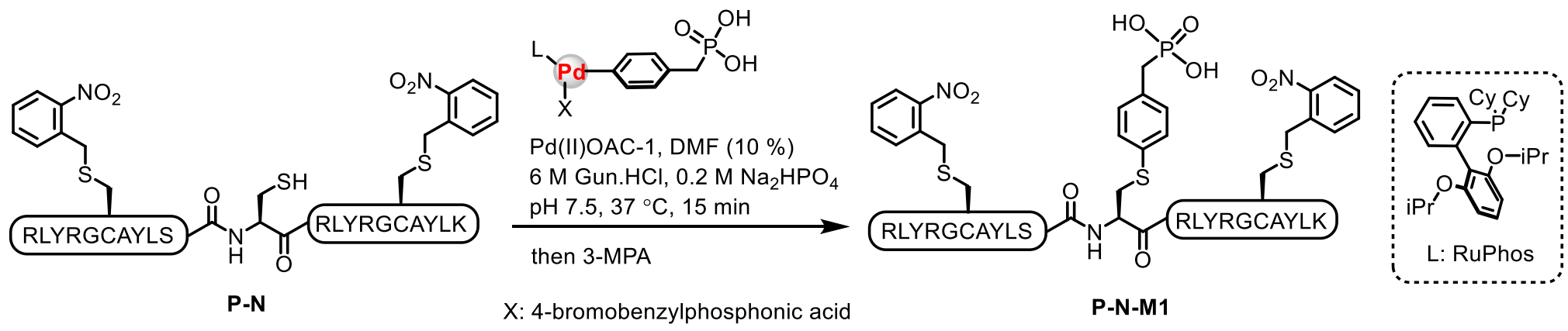

B

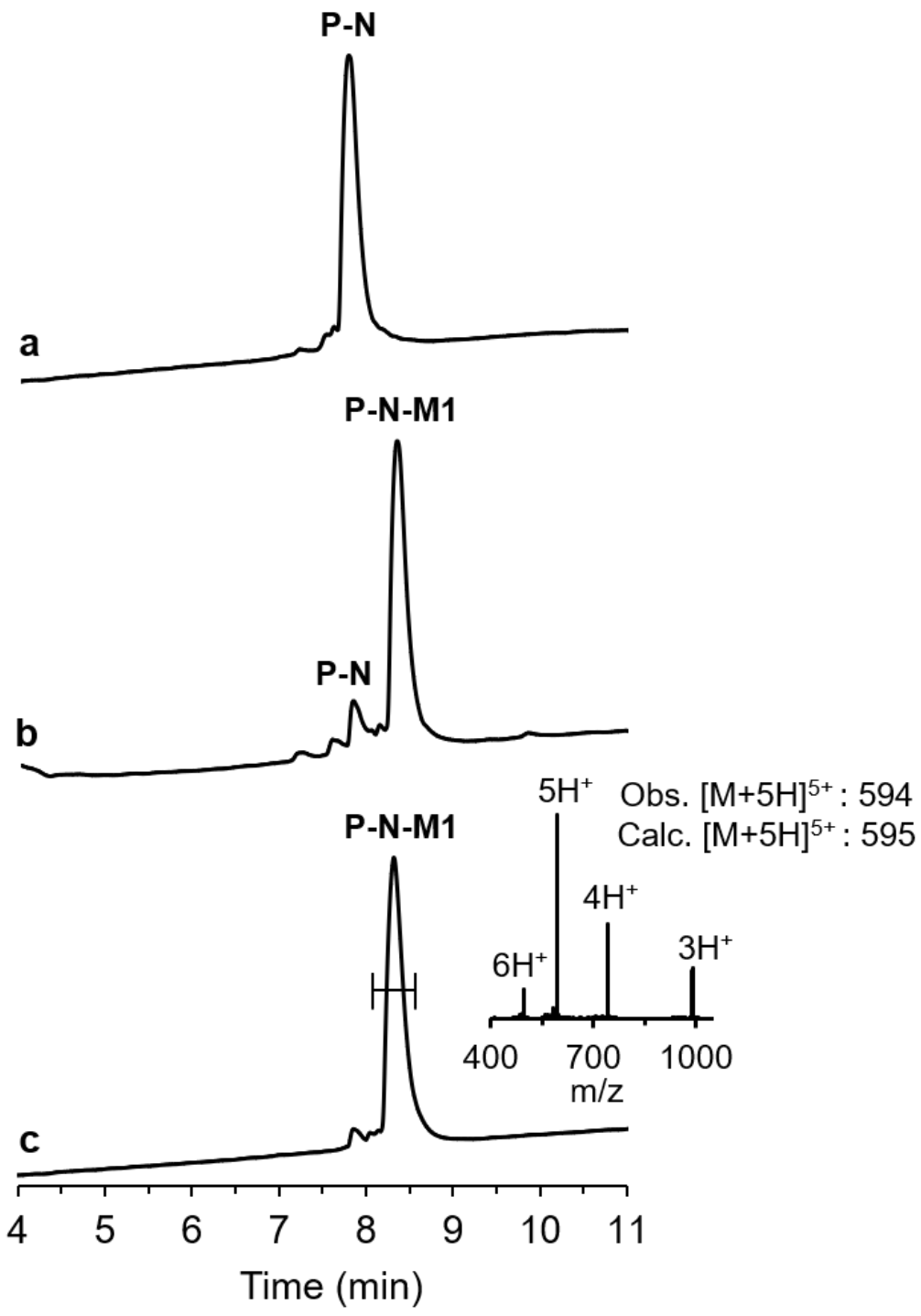

Supplementary Fig. 25 A Schematic representation of site-specific insertion of phospho-Tyr to **P-N**. **B** LC-MS analysis of the phospho-Tyr modification of **P-N**. (a) **P-N** at t = 0. (b) Arylation at t = 15 min; phospho-Tyr analog of **P-N** (**P-N-M1**). (c) Isolated **P-N-M1**.

6.2 Decaging of P-N-M1 peptide

In a 2.0 mL Eppendorf tube, **P-N-M1** (2.4 mg, 1.0 equiv., 0.81 µmol, 0.51 mM) was dissolved in 6 M Gun.HCl, 0.2 M Na_2HPO_4 buffer (1.6 mL). To this solution, DTT (2.5 mg, 20.0 equiv., 16.2 µmol, 0.81 M; based on **P-N-M1**) dissolved in 6 M Gun.HCl, 0.2 M Na_2HPO_4 buffer (20 µL) was added, and the pH was adjusted to 4 using 5 *N* HCl. The reaction mixture was then placed in a UV chamber and irradiated with 350 nm light for 1 h at rt. The progress of the reaction was monitored by LC–MS (Method A, Section 1.2). After completion, the mixture was purified by RP-HPLC (Method C, Section 1.3) to afford the final product **P-M1** (51% isolated yield) as a white powder.

A

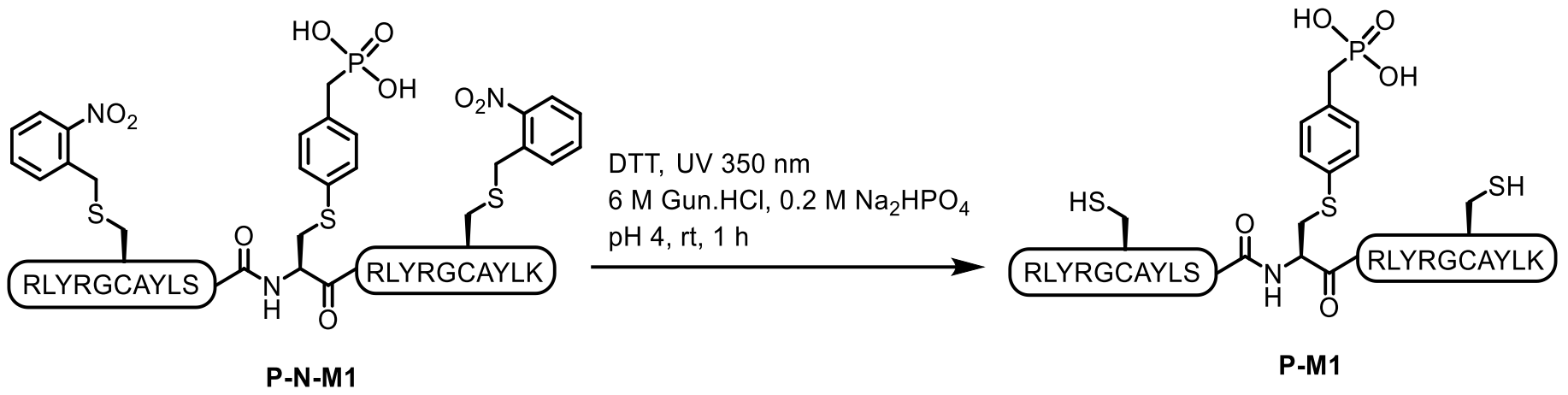

B

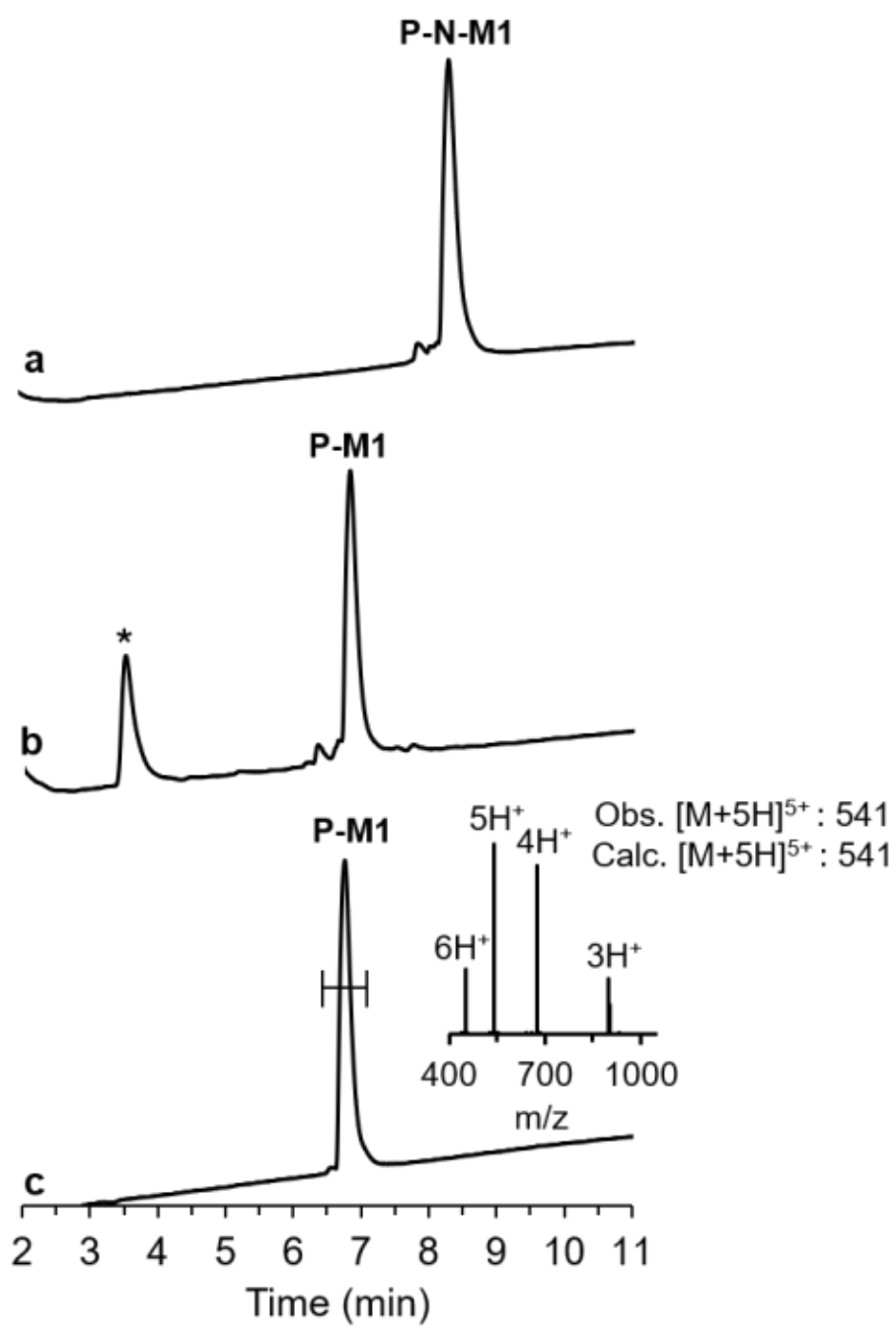

Supplementary Fig. 26 A Schematic representation of decaging of **P-N-M1**. **B** LC-MS analysis of decaging of **P-N-M1**. (a) **P-N-M1** at t = 0. (b) Decaging at t = 1 h; phospho-Tyr analog of **P** (**P-M1**). (c) Isolated **P-M1**. * NB decomposition byproduct.

6.3 Site-specific insertion of dimethyl-Lys to the P peptide

In a 15 mL Falcon tube, **P-N** (5 mg, 1.0 equiv., 1.8 µmol, 0.67 mM) was dissolved in 6 M Gun.HCl, 0.2 M Na_2HPO_4 buffer (2.70 mL), followed by the addition of TCEP (5.2 mg, 10.0 equiv., 18.0 µmol, 0.72 M; based on **P-N**) dissolved in 6 M Gun.HCl, 0.2 M Na_2HPO_4 buffer (25 µL). Separately, 2-bromo-N,N-dimethylethylamine hydrobromide (12.4 mg, 30.0 equiv., 54.0 µmol, 60 mM; based on **P-N**) and NaI (270 mg, 33.3 equiv., 1.8 mmol, 2.0 M; based on 2-bromo-N,N-dimethylethylamine hydrobromide) were dissolved in MeOH (0.9 mL, 25% v/v) and activated for 10 min at 37 °C. The resulting solution was added in one portion to the Falcon tube containing **P-N**, and the pH was adjusted to 8 using 5 *N* NaOH. The mixture was incubated at 37 °C for 1 h, and the reaction progress was monitored by LC–MS (Method A, Section 1.2). After completion, TCEP (46.2 mg, 90.0 equiv., 162.0 µmol, 0.72 M; based on **P-N**) in 6 M Gun.HCl, 0.2 M Na_2HPO_4 buffer (225 µL) and GSH (83.0 mg, 150.0 equiv., 270.0 µmol, 1.1 M; based on **P-N**) in 6 M Gun.HCl, 0.2 M Na_2HPO_4 buffer (250 µL) was added to the reaction mixture. The pH was adjusted to 4 using 5 *N* HCl. Then, the reaction mixture was placed in a UV chamber and irradiated with 350 nm light for 1 h at rt. The reaction was monitored by LC-MS (Method A described in Section 1.2).[7] After the completion of the reaction, the reaction mixture was purified using RP-HPLC (Method A described in Section 1.3), affording the final product **P-M2** (50% isolated yield) as a white powder.

A

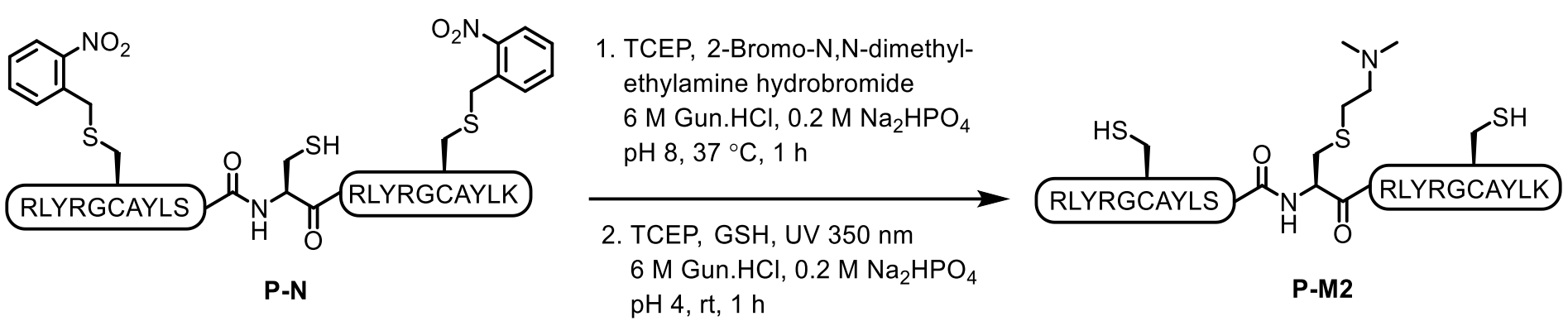

B

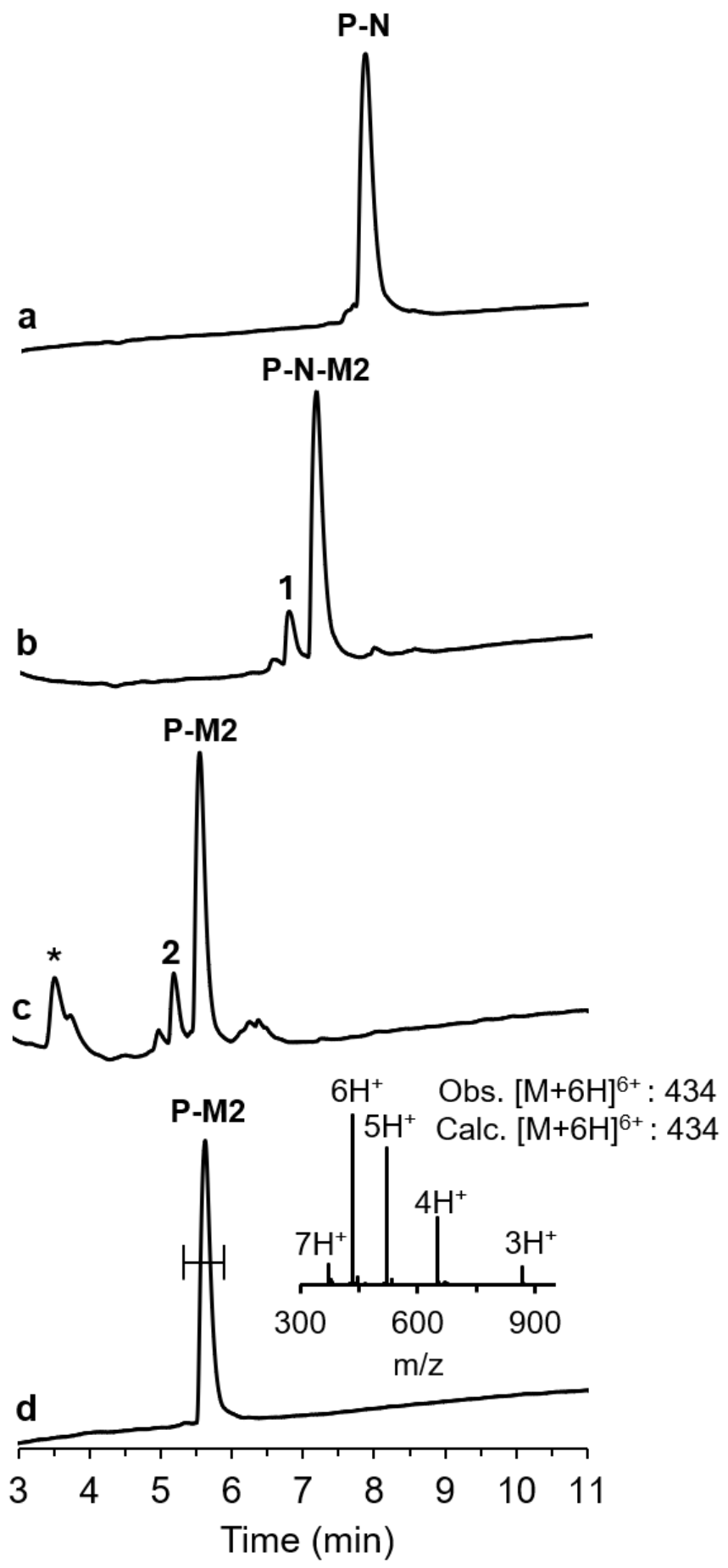

Supplementary Fig. 27 A Schematic representation of site-specific insertion of dimethyl-Lys to **P-N** and decaging. **B** LC-MS analysis of one-pot dimethylation and decaging of **P-N**. (a) **P-N** at t = 0. (b) Alkylation at t = 1 h; dimethyl-Lys mimic of **P-N** (**P-N-M2**) (c) Decaging at t = 2 h; dimethyl-Lys mimic of **P** (**P-M2**). (d) Isolated **P-M2**. * NB decomposition byproduct, **1** dialkylated product of **P-N** and **2** decaged dialkylated products.

6.4 Site-specific insertion of acetyl-Lys to the P model peptide

In a 2 mL Eppendorf tube, **P-N** (2.5 mg, 1.0 equiv., 0.89 µmol, 0.55 mM) was dissolved in 6 M Gun.HCl, 0.2 M Na_2HPO_4 buffer (1.62 mL). To this solution di-bromo-adipyl(bis)amide[8,9] (26.7 mg, 100.0 equiv., 89.0 µmol, 0.49 M; based on **P-N**) dissolved in DMF (V = 0.18 mL, 10%) was added. The pH was adjusted to 8 using 5 *N* NaOH. The mixture was then incubated for 1 h at 25 °C, then further incubated for 2 h at 37 °C. The reaction was monitored by LC-MS (Method A described in Section 1.2). After completion, N-acetylcysteamine (56.8 µL, 600.0 equiv., 534.0 µmol; based **on P-N**) was added to the reaction mixture, and the pH was adjusted to 8 using 5 *N* NaOH. The mixture was then incubated at 37 °C for 3 h, with the reaction progress monitored by LC–MS (Method A, Section 1.2). The formation of **P-N-M3** was monitored using LC-MS (Method A described in Section 1.2), and after completion of the reaction, decaging of **P-N-M3** was performed as described in section 6.2, affording the final product **P-M3** (26% isolated yield) as a white powder.

A

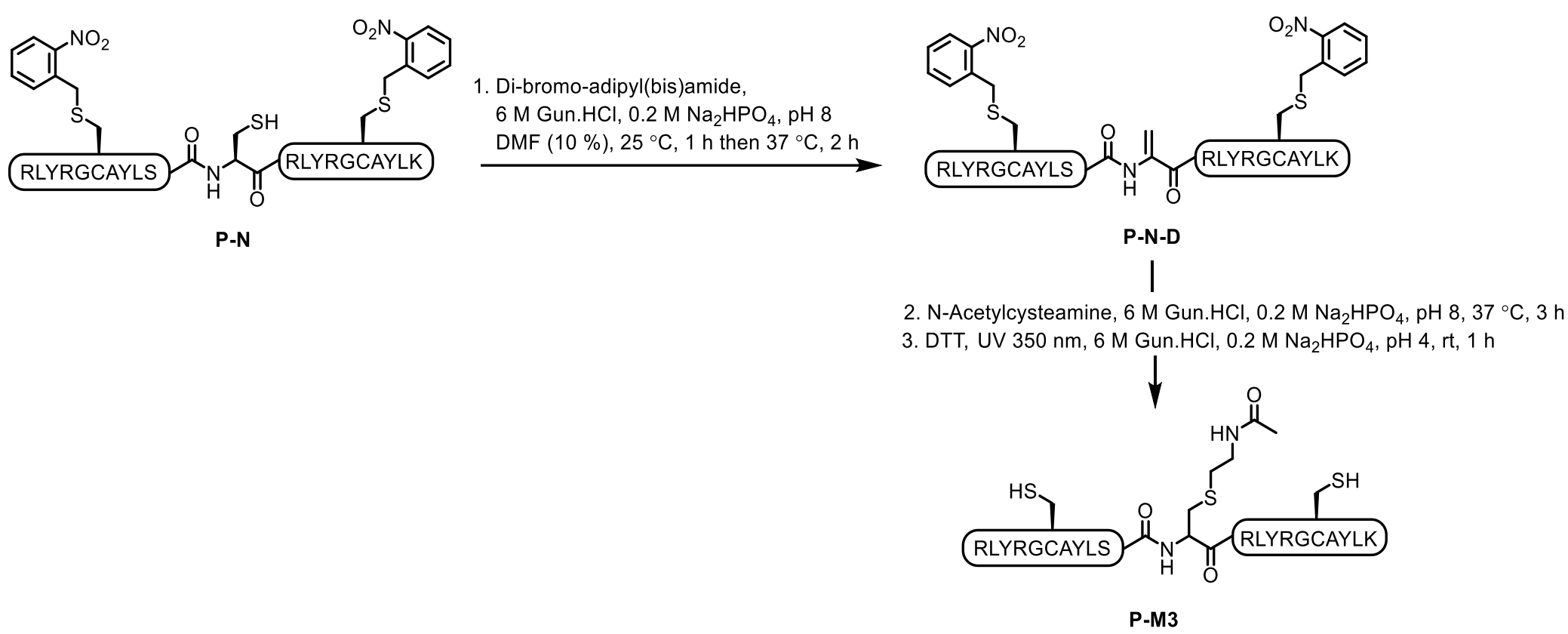

B

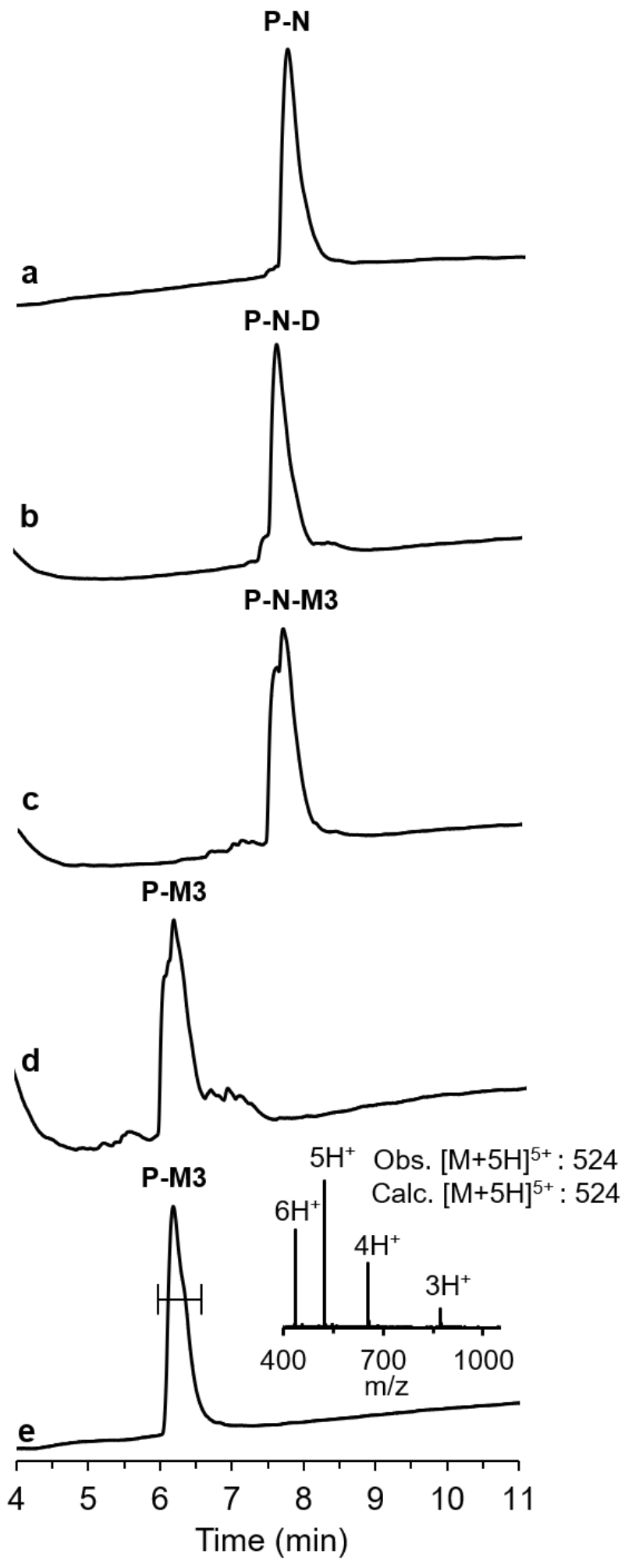

Supplementary Fig. 28 A Schematic representation of site-specific insertion of acetyl-Lys to **P-N** and decaging. **B** LC-MS analysis of one-pot acetylation and decaging of **P-N**. (a) **P-N** at t = 0. (b) Cys to dehydroalanine conversion at t = 3 h; dehydroalanine analog of **P-N** (**P-N-D**). (c) Michael addition to **P-N-D** at t = 6 h; Michael addition product of **P-N-D** (**P-N-M3**). (d) Decaging at t = 7 h; acetyl-Lys mimic of **P (P-M3)**. (e) Isolated **P-M3**.

7. Site-specific modification of ZF protein

7.1 Site-specific insertion of monomethyl-Lys to ZF-N protein

In a 1.5 mL Eppendorf tube, **ZF-N** (2.0 mg, 1.0 equiv., 0.26 µmol, 0.5 mM) was dissolved in 4 M Gun.HCl, 1 M HEPES, 10 mM DL-methionine buffer (0.52 mL). To this solution, DTT (1.2 mg, 30.0 equiv., 7.8 µmol, 0.78 M; based on **ZF-N**) dissolved in the same buffer (10 µL) was added, and the **ZF-N** protein was reduced for 30 min at 37 °C. Separately, 2-chloro-N-methylethanamine hydrochloride (16.9 mg, 500.0 equiv., 130 µmol, 2.6 M; based on **ZF-N**) was dissolved in 4 M Gun.HCl, 1 M HEPES, 10 mM DL-methionine buffer (50 µL). This solution was added in one portion to the Eppendorf tube containing **ZF-N**, and the pH was adjusted to 8 using 5 *N* NaOH. The mixture was then incubated at 37 °C for 8 h. The reaction progress was monitored by LC–MS (Method A, Section 1.2). After completion, the mixture was purified by RP-HPLC (Method C, Section 1.3) to afford the final product **ZF-N-M1** (35% isolated yield) as a white powder.

A

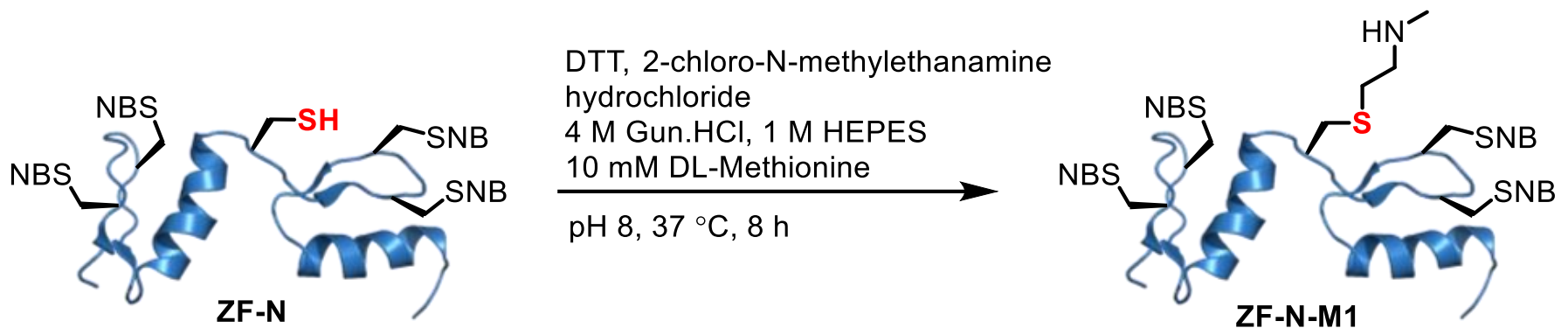

B

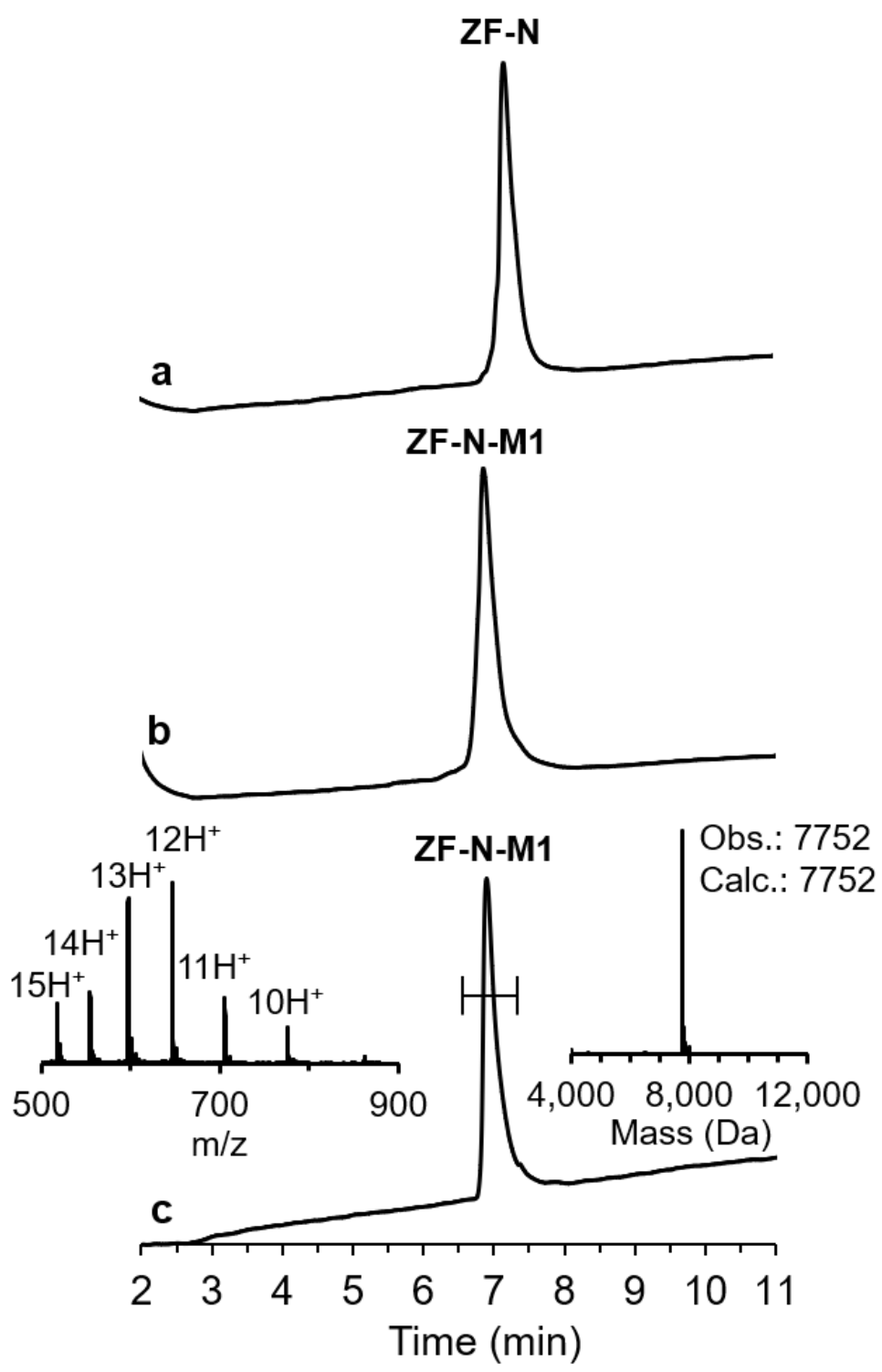

Supplementary Fig. 29 A Schematic representation of site-specific insertion of monomethyl-Lys to **ZF-N**. **B** LC-MS analysis of monomethylation of ZF-N. (a) **ZF-N** at t = 0. (b) Alkylation at t = 8 h; monomethyl-Lys analog of **ZF-N** (**ZF-N-M1**). (c) Isolated **ZF-N-M1**.

7.2 Decaging of ZF-N-M1 protein

In a 1.5 mL Eppendorf tube, **ZF-N-M1** (0.1 mg, 1.0 equiv., 0.013 µmol, 0.52 mM) was dissolved in 6 M Gun.HCl, 0.2 M Na_2HPO_4 buffer (25 µL). To this solution, DTT (80 µg, 40.0 equiv., 0.52 µmol, 0.52 M; based on **ZF-N-M1**) dissolved in 6 M Gun.HCl, 0.2 M Na_2HPO_4 buffer (1 µL) was added, and the pH was adjusted to 4 using 5 *N* HCl. The reaction mixture was then placed in a UV chamber and irradiated with 350 nm light for 1.5 h at rt. The reaction progress was monitored by LC–MS (Method A, Section 1.2). After completion, LC–MS analysis indicated a 99% conversion to the final product **ZF-M1**.

A

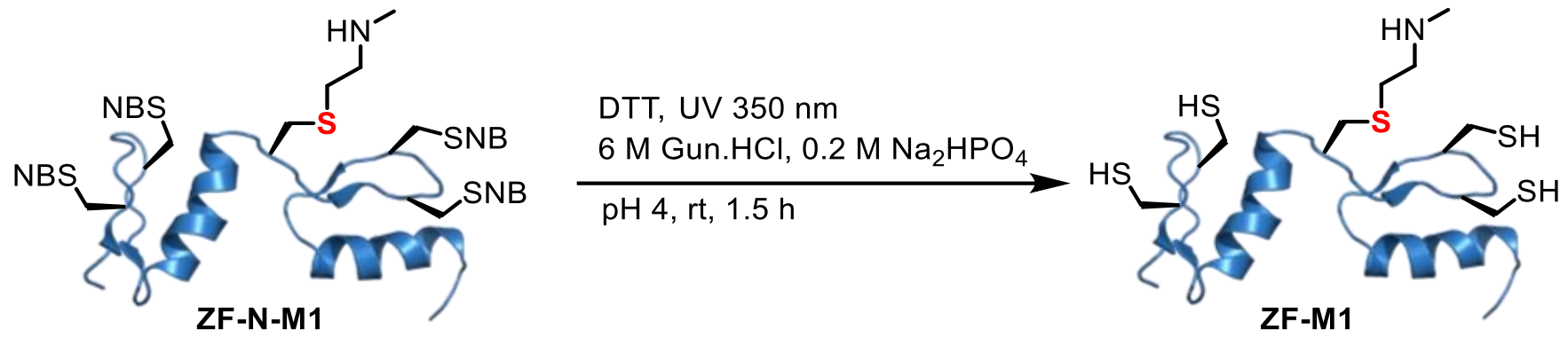

B

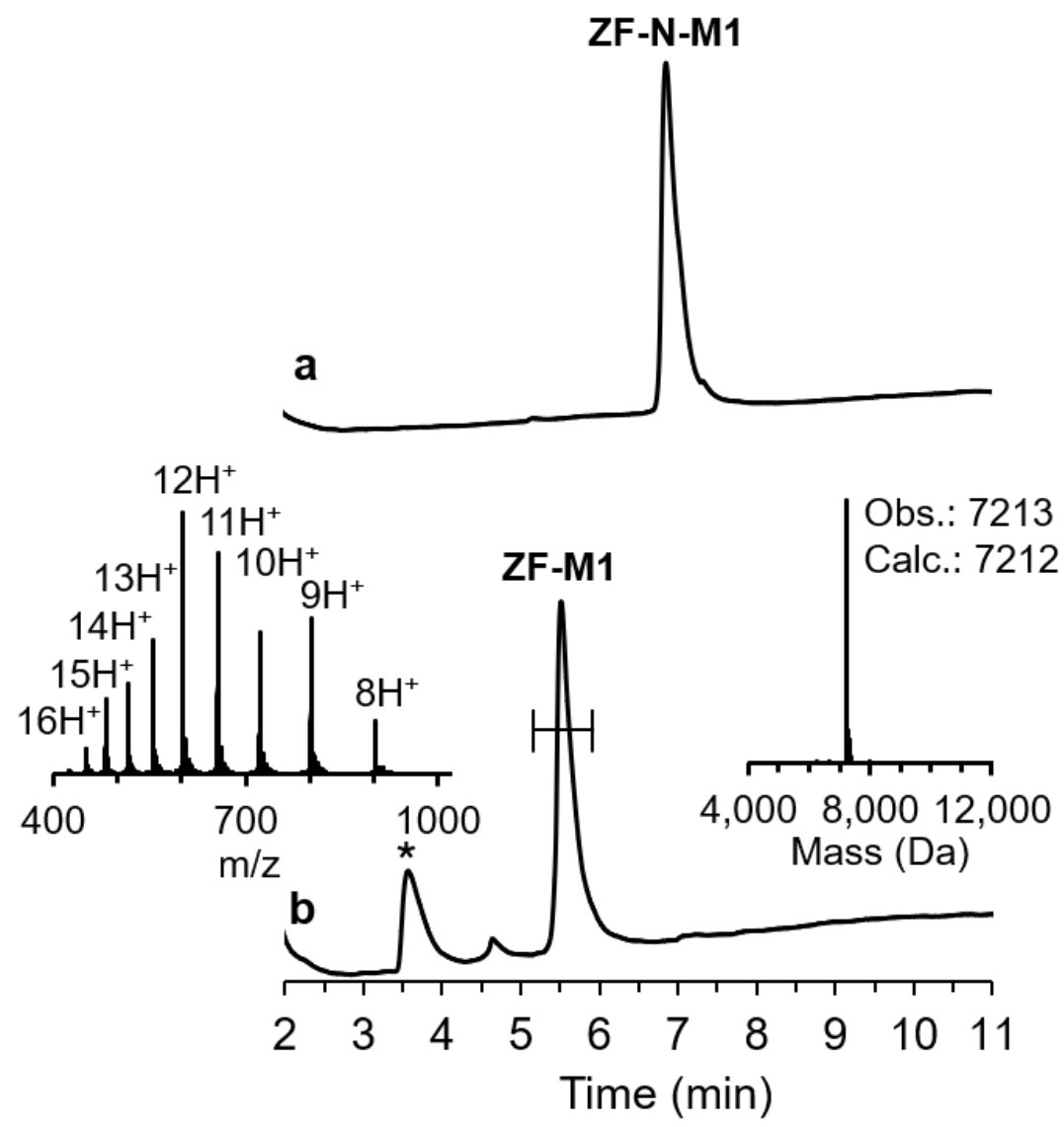

Supplementary Fig. 30 A Schematic representation of decaging of **ZF-N-M1**. **B** LC-MS analysis of decaging of **ZF-N-M1**. (a) **ZF-N-M1** at t = 0. (b) Decaging at t = 1.5 h; monomethyl-Lys analog of **ZF** (**ZF-M1**) and * NB decomposition byproduct.

7.3 Site-specific insertion of dimethyl-Lys to ZF-N protein

In a 2 mL Eppendorf tube, **ZF-N** (4 mg, 1.0 equiv., 0.52 µmol, 0.67 mM) was dissolved in 6 M Gun.HCl, 0.2 M Na_2HPO_4 buffer (0.75 mL). To this solution, DTT (0.4 mg, 5.0 equiv., 2.6 µmol, 1.3 M; based on **ZF-N**) dissolved in the same buffer (2 µL) was added, and the **ZF-N** protein was reduced for 30 min at 37 °C. Separately, 2-bromo-N,N-dimethylethylamine hydrobromide (3.6 mg, 30.0 equiv., 15.6 µmol, 62.4 mM; based on **ZF-N**) and NaI (116 mg, 50 equiv., 0.78 mmol, 1.9 M; based on 2-bromo-N,N-dimethylethylamine hydrobromide) were dissolved in MeOH (0.25 mL, 25% v/v) and activated for 10 min at 37 °C. The resulting solution was added in one portion to the Eppendorf tube containing **ZF-N**, and the pH was adjusted to 8 using 5 *N* NaOH. The mixture was then incubated at 37 °C for 1 h. The reaction progress was monitored by LC–MS (Method A, Section 1.2). After completion, the reaction mixture was purified by RP-HPLC (Method C, Section 1.3) to afford the final product **ZF-N-M2** (50% isolated yield) as a white powder.

A

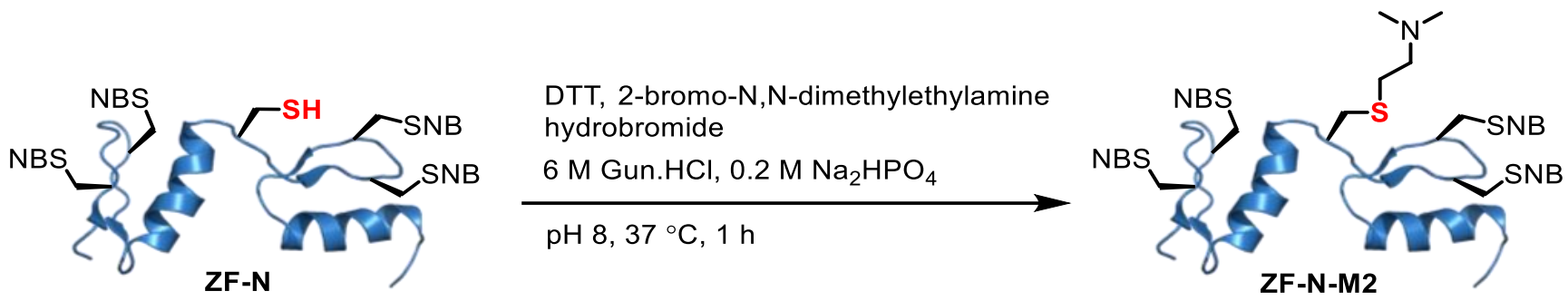

B

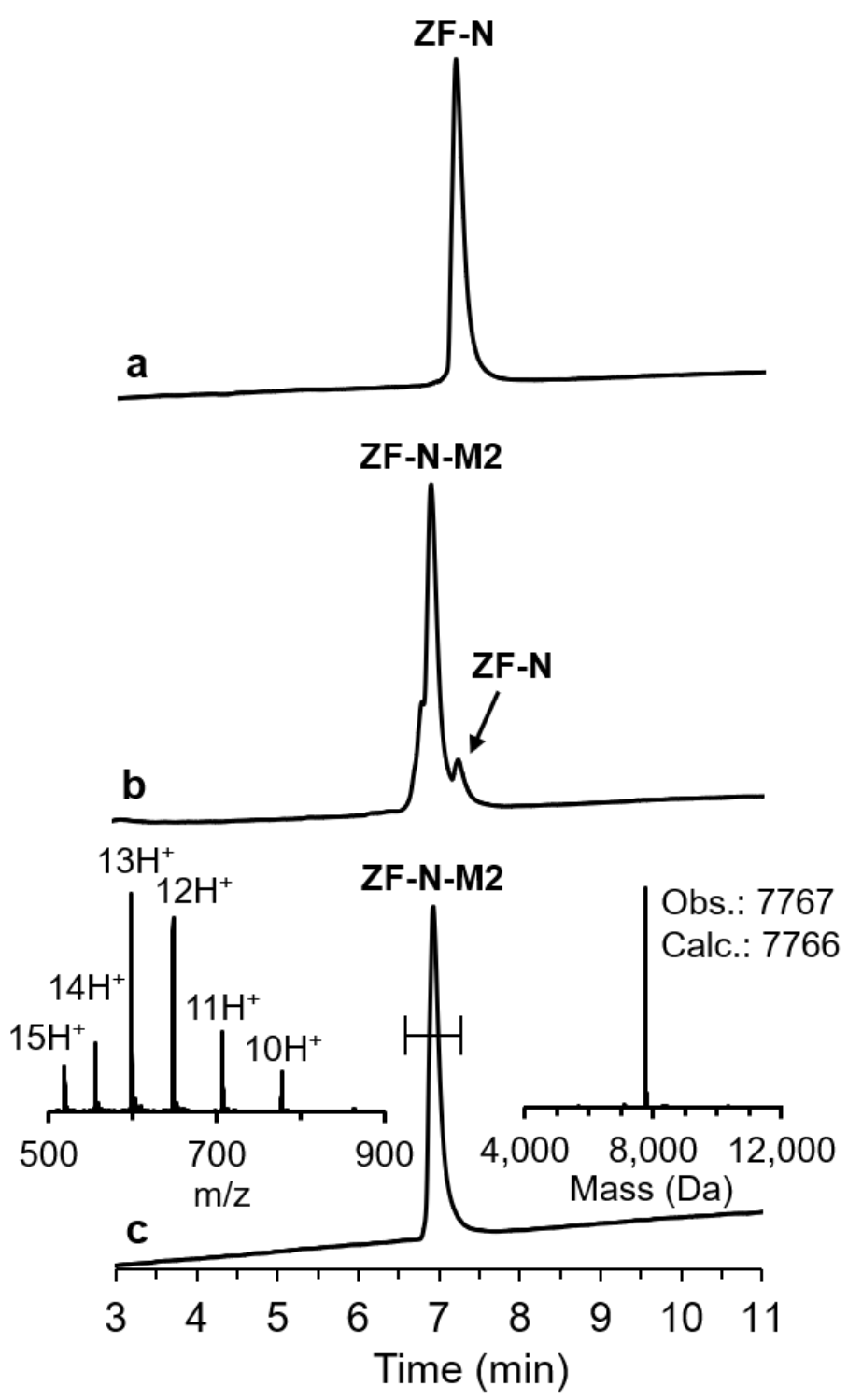

Supplementary Fig. 31 A Schematic representation of site-specific insertion of dimethyl-Lys to **ZF-N**. **B** LC-MS analysis of dimethylation of **ZF-N**. (a) **ZF-N** at t = 0. (b) Alkylation at t = 1 h; dimethyl-Lys analog of **ZF-N** (**ZF-N-M2**). (c) Isolated **ZF-N-M2**.

7.4 Decaging of ZF-N-M2 protein

The decaging of **ZF-N-M2** was performed as described in section 7.2, affording the final product **ZF-M2** (61% isolated yield) as a white powder after purification by RP-HPLC (Method C, Section 1.3).

A

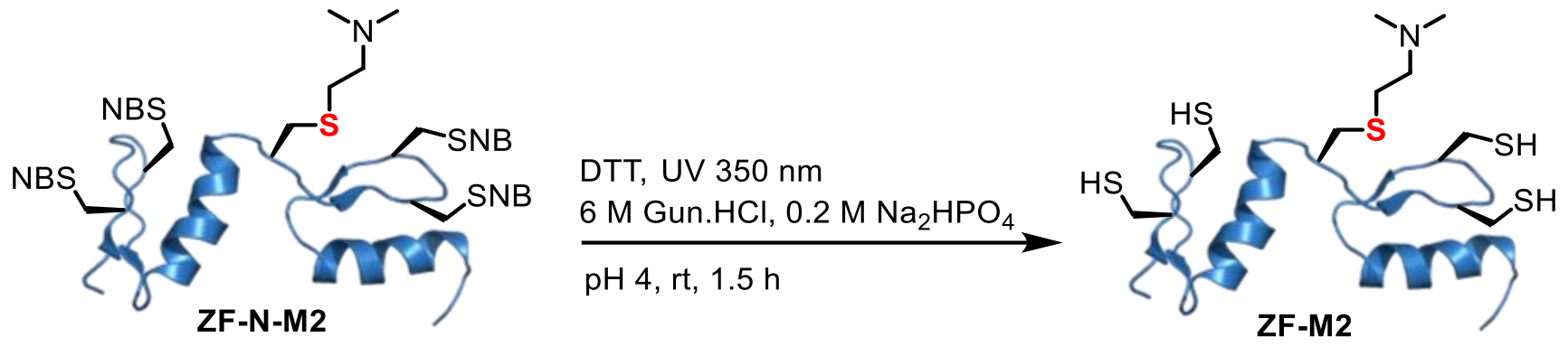

B

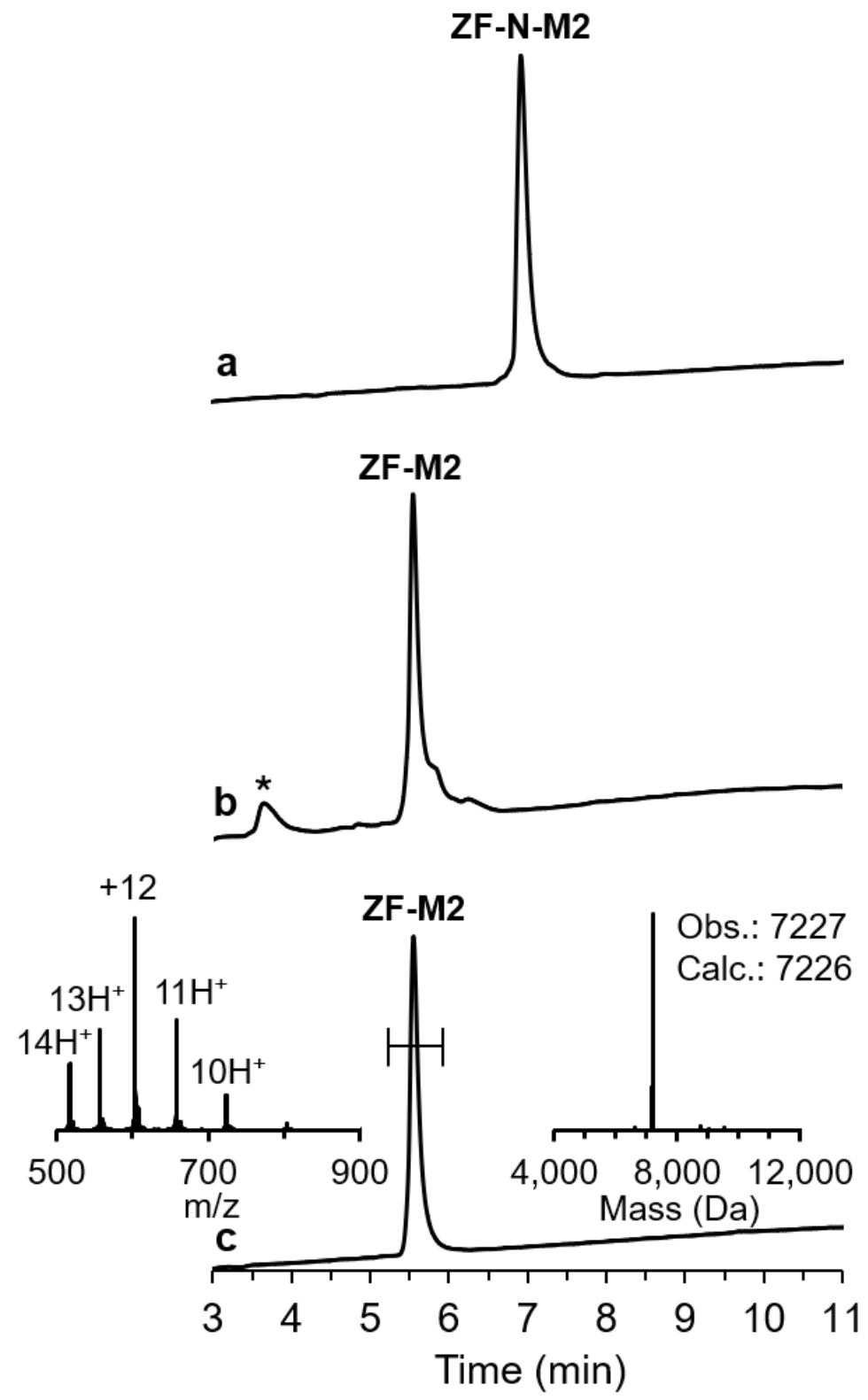

Supplementary Fig. 32 A Schematic representation of decaging of **ZF-N-M2**. **B** LC-MS analysis of decaging of **ZF-N-M2**. (a) **ZF-N-M2** at t = 0. (b) Decaging at t = 1.5 h; dimethyl-Lys analog of **ZF** (**ZF-M2**). (c) Isolated **ZF-M2**. * NB decomposition byproduct.

7.5 Site-specific insertion of trimethyl-Lys to ZF-N protein

The trimethyl-Lys modification of **ZF-N** protein using (2-bromoethyl)trimethylammonium bromide was performed as described in section 7.1, affording the final product **ZF-N-M3** (35% isolated yield) as a white powder.

A

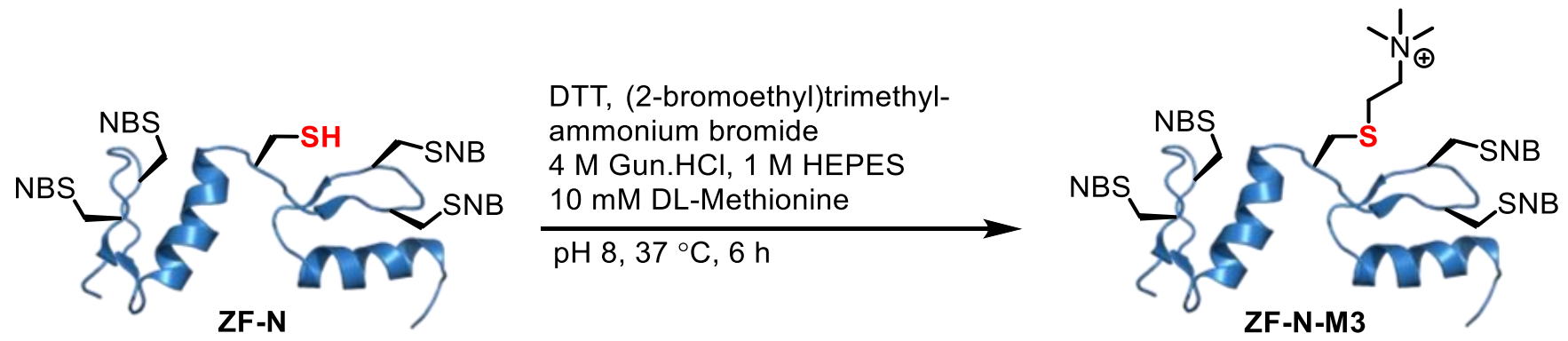

B

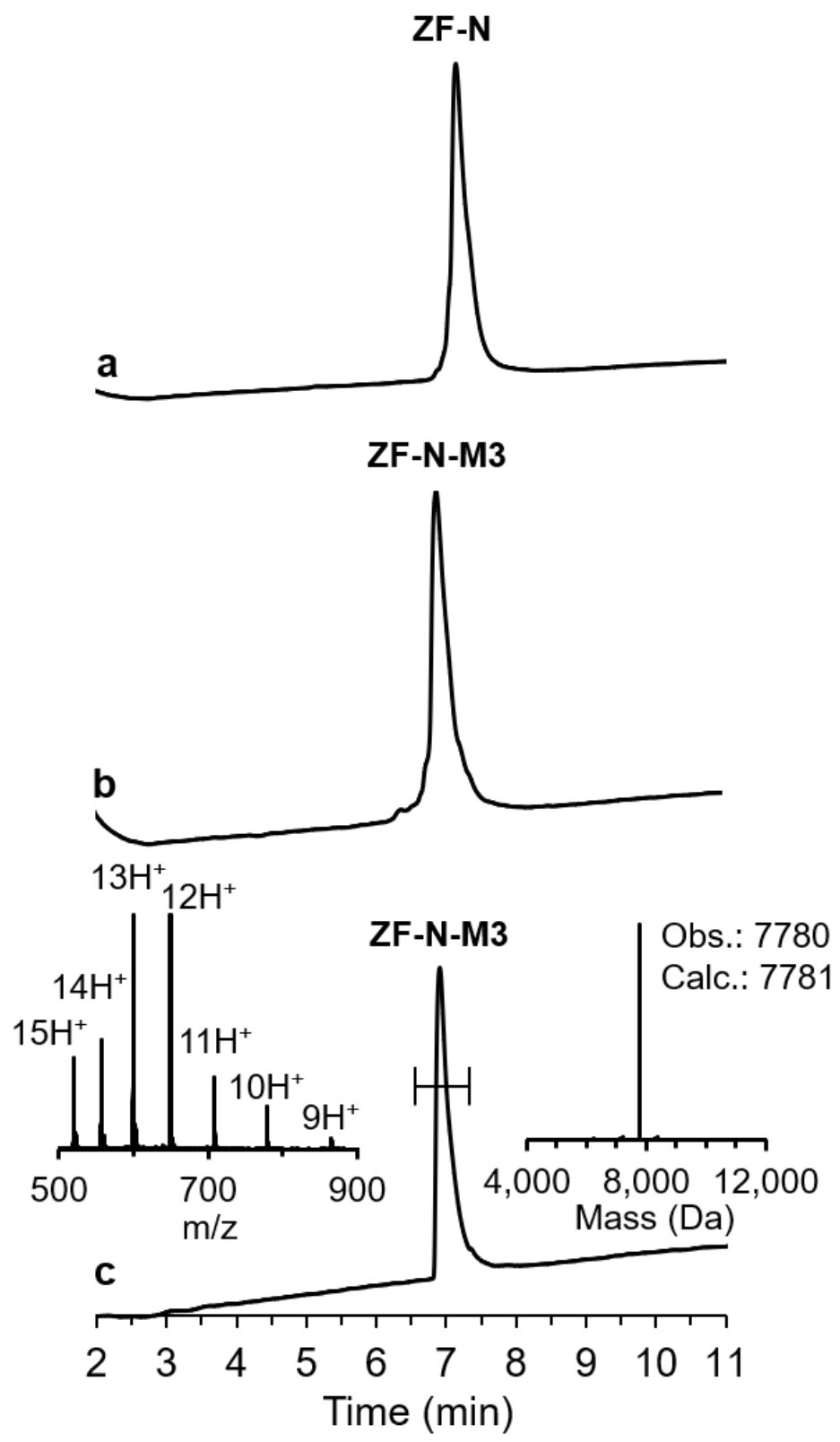

Supplementary Fig. 33 A Schematic representation of site-specific insertion of trimethyl-Lys to **ZF-N**. **B** LC-MS analysis of trimethylation of **ZF-N**. (a) **ZF-N** protein at t = 0. (b) Alkylation at t = 6 h; trimethyl-Lys analog of **ZF-N** (**ZF-N-M3**). (c) Isolated **ZF-N-M3**.

7.6 Decaging of ZF-N-M3 protein

The decaging of **ZF-N-M3** to form **ZF-M3** was performed as described in section 7.2.

A

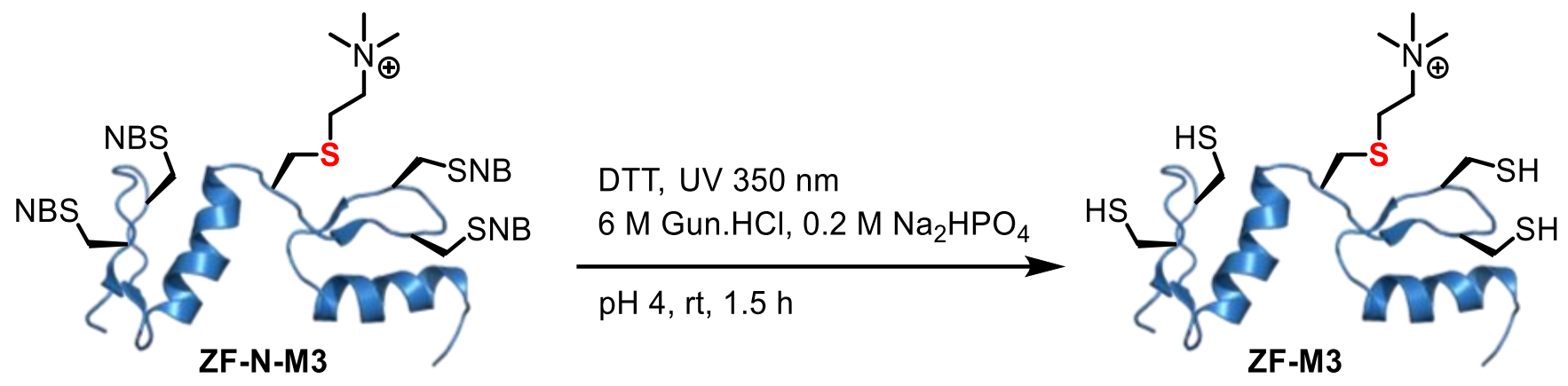

B

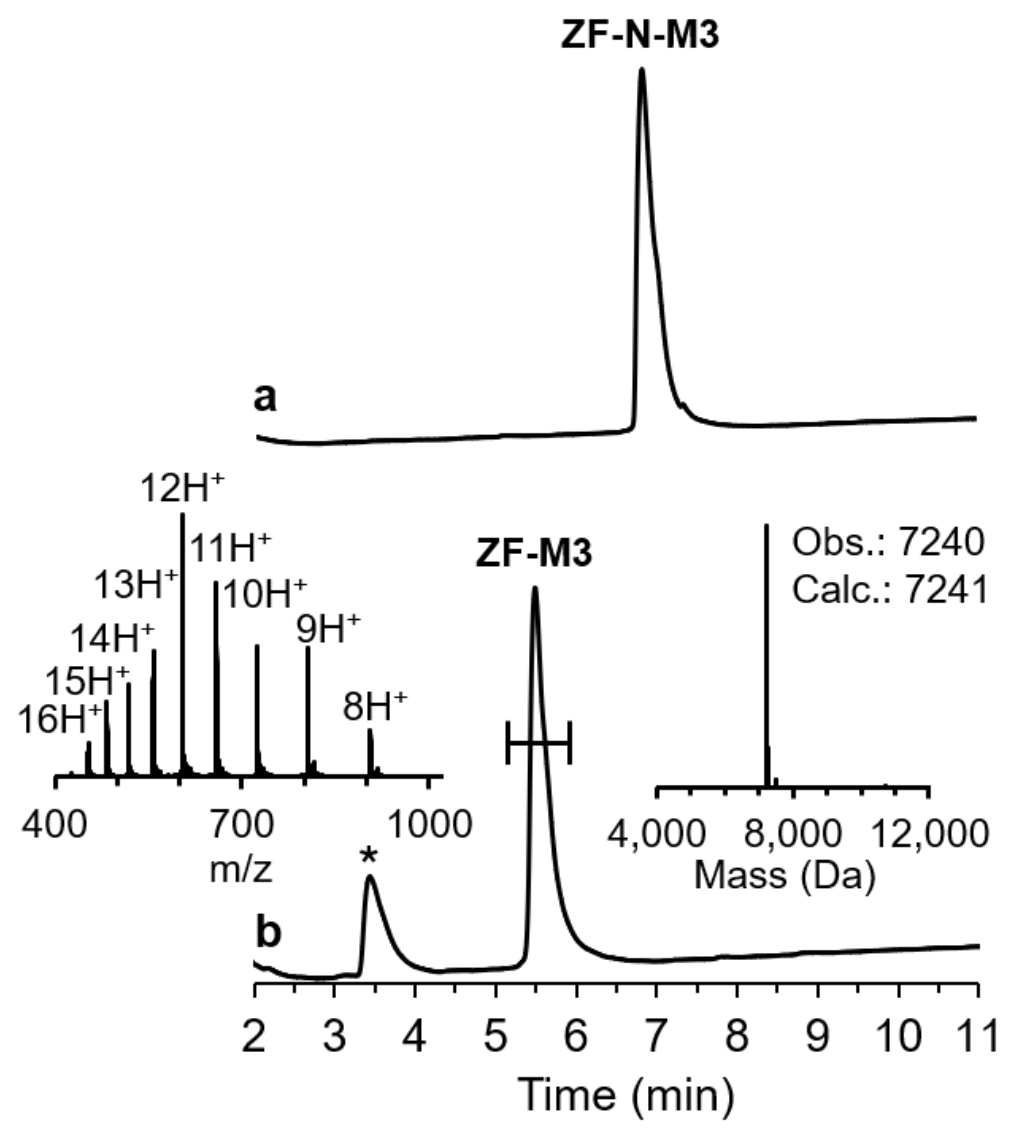

Supplementary Fig. 34 A Schematic representation of decaging of **ZF-N-M3**. **B** LC-MS analysis of decaging of **ZF-N-M3**. (a) **ZF-N-M3** at t = 0. (b) Decaging at t = 1.5 h; trimethyl-Lys analog of **ZF** (**ZF-M3**) and * NB decomposition byproduct.

7.7 Site-specific insertion of phospho-Tyr to ZF-N protein

In a 15 mL Falcon tube, **ZF-N** (4.0 mg, 1.0 equiv., 0.51 µmol, 0.2 mM) was added in 6 M Gun.HCl, 0.2 M Na_2HPO_4 buffer (2.5 mL, pH 7.5) and followed by Pd(II)OAC-1 (1.56 mg, 3 equiv., 1.53 µmol, 11.8 mM; based on **ZF-N**) in DMF solvent (V = 0.13 mL, 5%). The reaction mixture was vigorously mixed for a few seconds and the mixture was then incubated for 15 min at 37 °C. The reaction was monitored by LC-MS (Method A, as described in Section 1.2). After confirming the completion of the reaction, the reaction mixture was quenched with 3-MPA (1.3 µL, 15.3 µmol, 10 equiv. relative to Pd(II)OAC-1) and incubated at 37 °C for 15 min. The reaction mixture was then spun down, and the mixture was purified using RP-HPLC (Method C, Section 1.3), affording the final product **ZF-N-M4** (47% isolated yield) as a white powder.

A

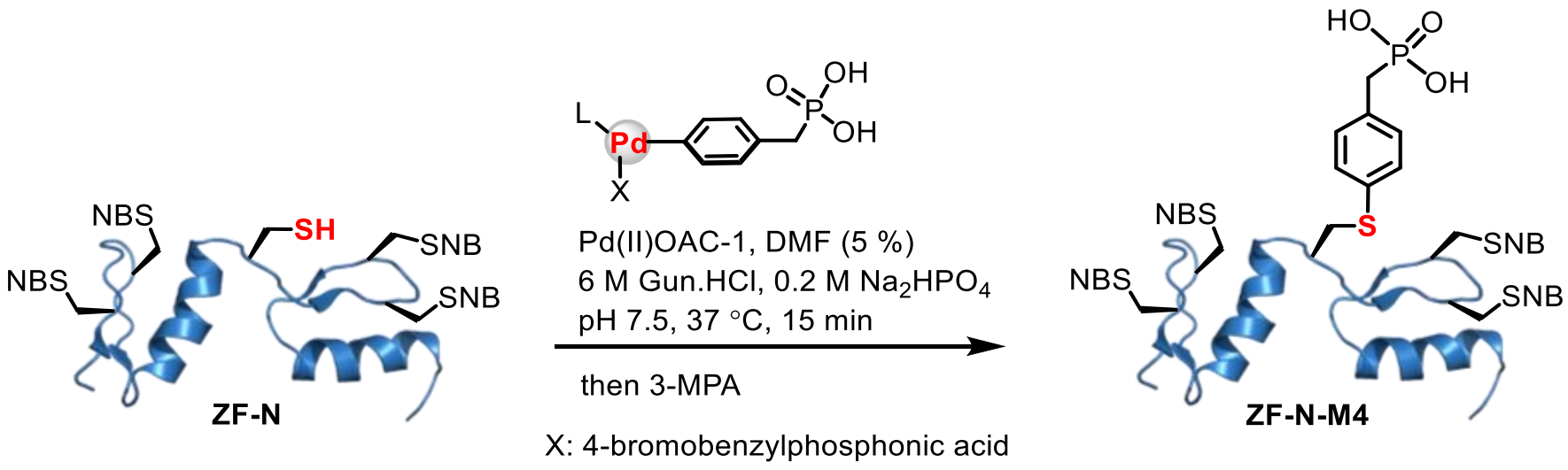

B

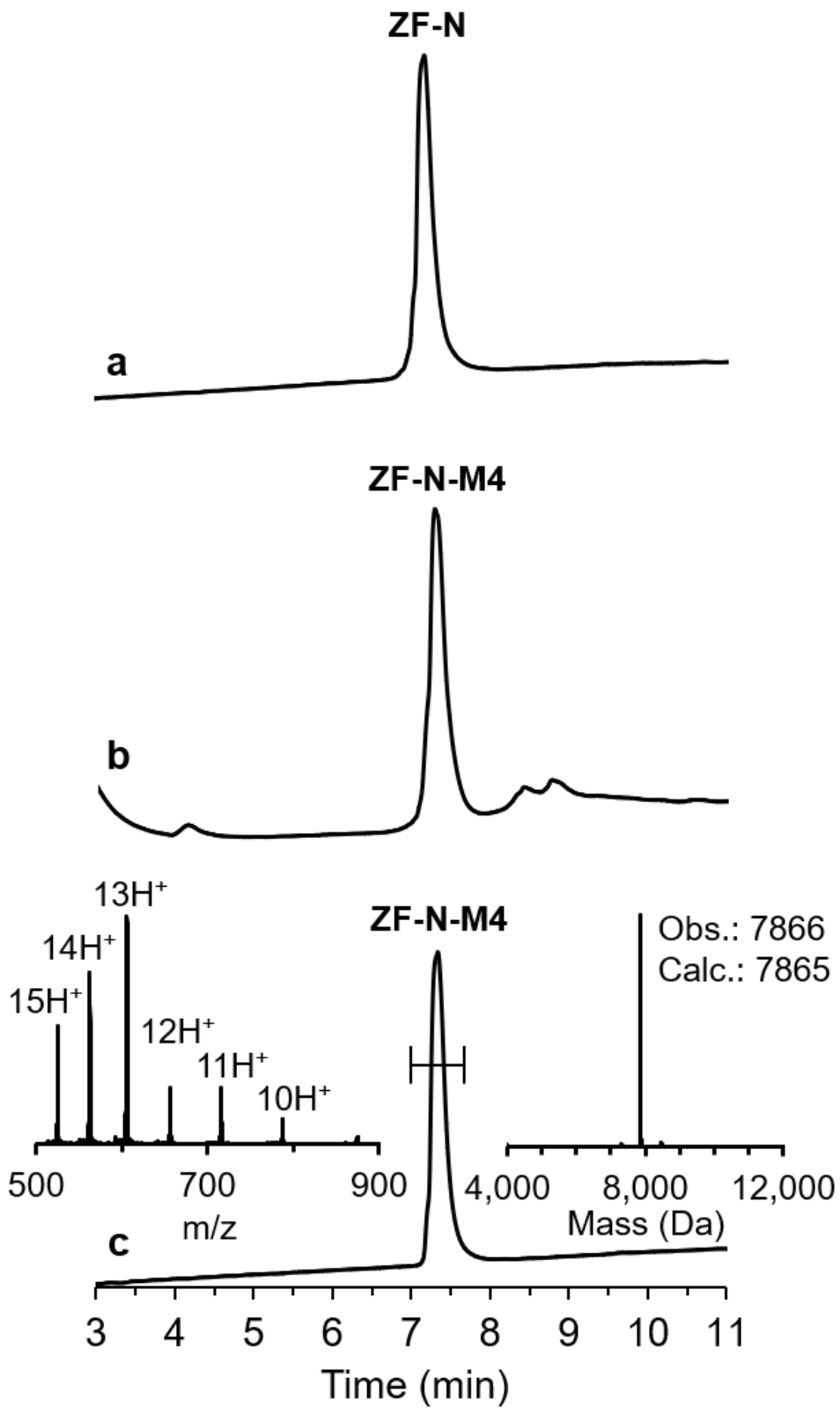

Supplementary Fig. 35 A Schematic representation of site-specific insertion of phospho-Tyr to **ZF-N**. **B** LC-MS analysis of the phospho-Tyr modification of **ZF-N**. (a) **ZF-N** at t = 0. (b) Arylation at t = 15 min, phospho-Tyr analog of **ZF-N** (**ZF-N-M4**). (c) Isolated **ZF-N-M4**.

7.8 Decaging of ZF-N-M4 protein

The decaging of **ZF-N-M4** was performed as described in section 7.2, affording the final product **ZF-M4** (73% isolated yield) as a white powder after purification by RP-HPLC (Method C, Section 1.3).

A

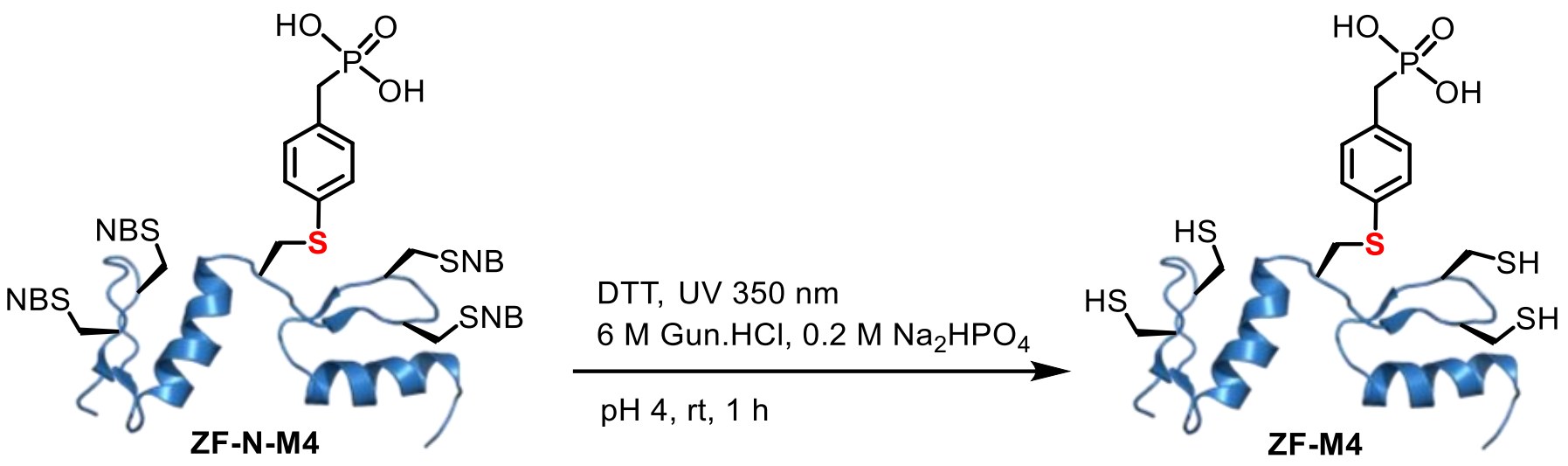

B

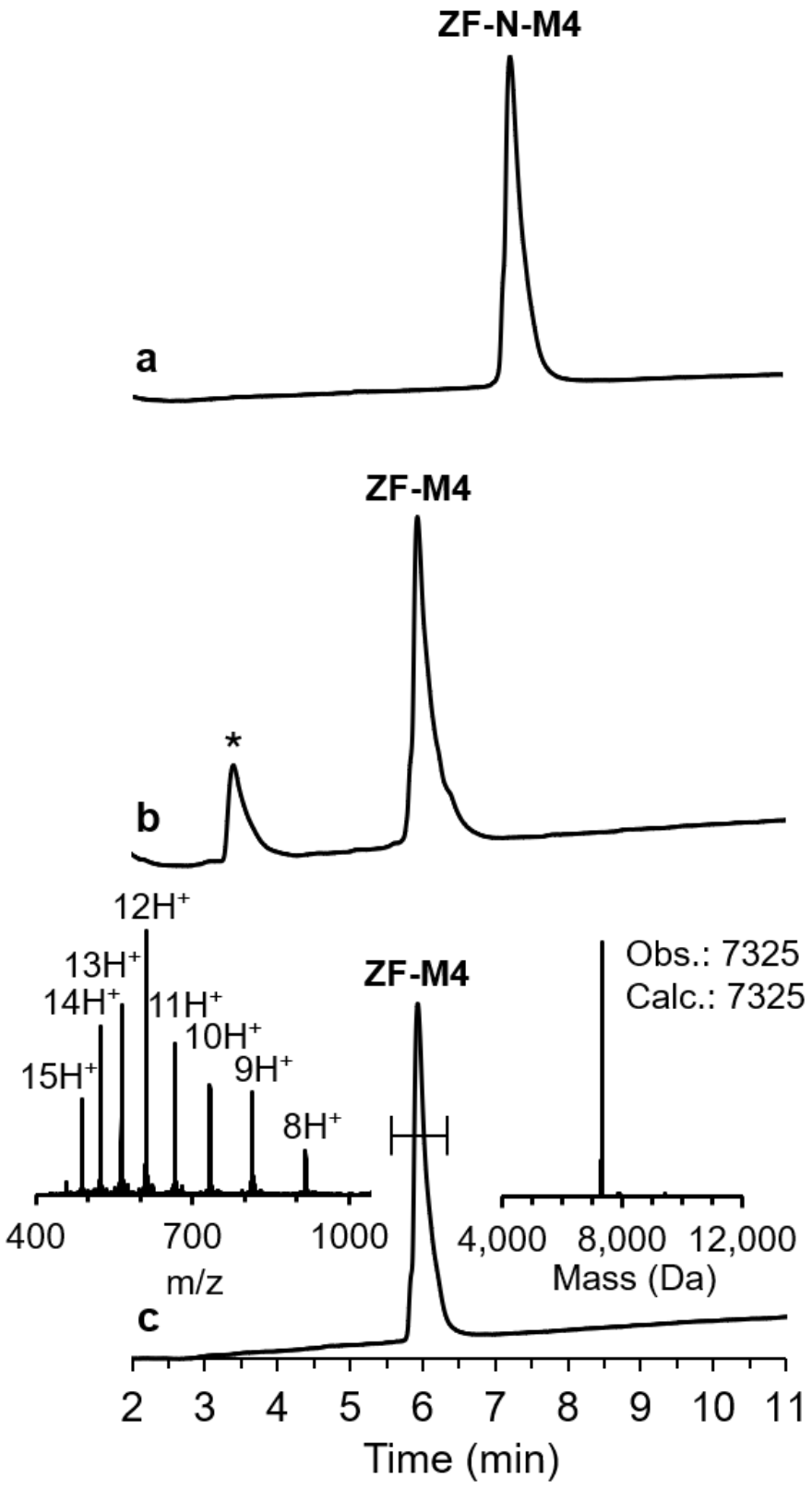

Supplementary Fig. 36 A Schematic representation of decaging of **ZF-N-M4**. **B** LC-MS analysis of decaging of **ZF-N-M4**. (a) **ZF-N-M4** at t = 0. (b) Decaging at t = 1 h; Phospho-Tyr analog of **ZF** (**ZF-M4**). (c) Isolated **ZF-M4**. * NB decomposition byproduct.

7.9 Site-specific insertion of phospho-Tyr to ZF-N protein

The Tyr-phosphorylation modification of **ZF-N** protein using Pd(II)OAC-2 was performed as described in section 7.7,[10] affording the final product **ZF-N-M5** (44% isolated yield) as a white powder.

A

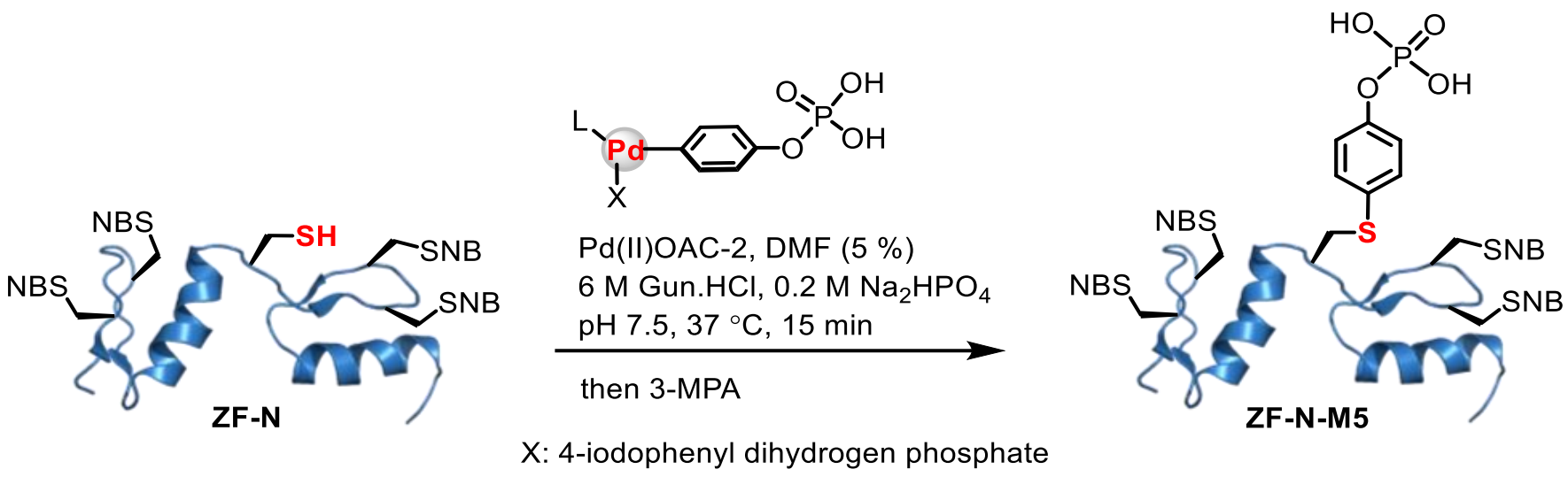

B

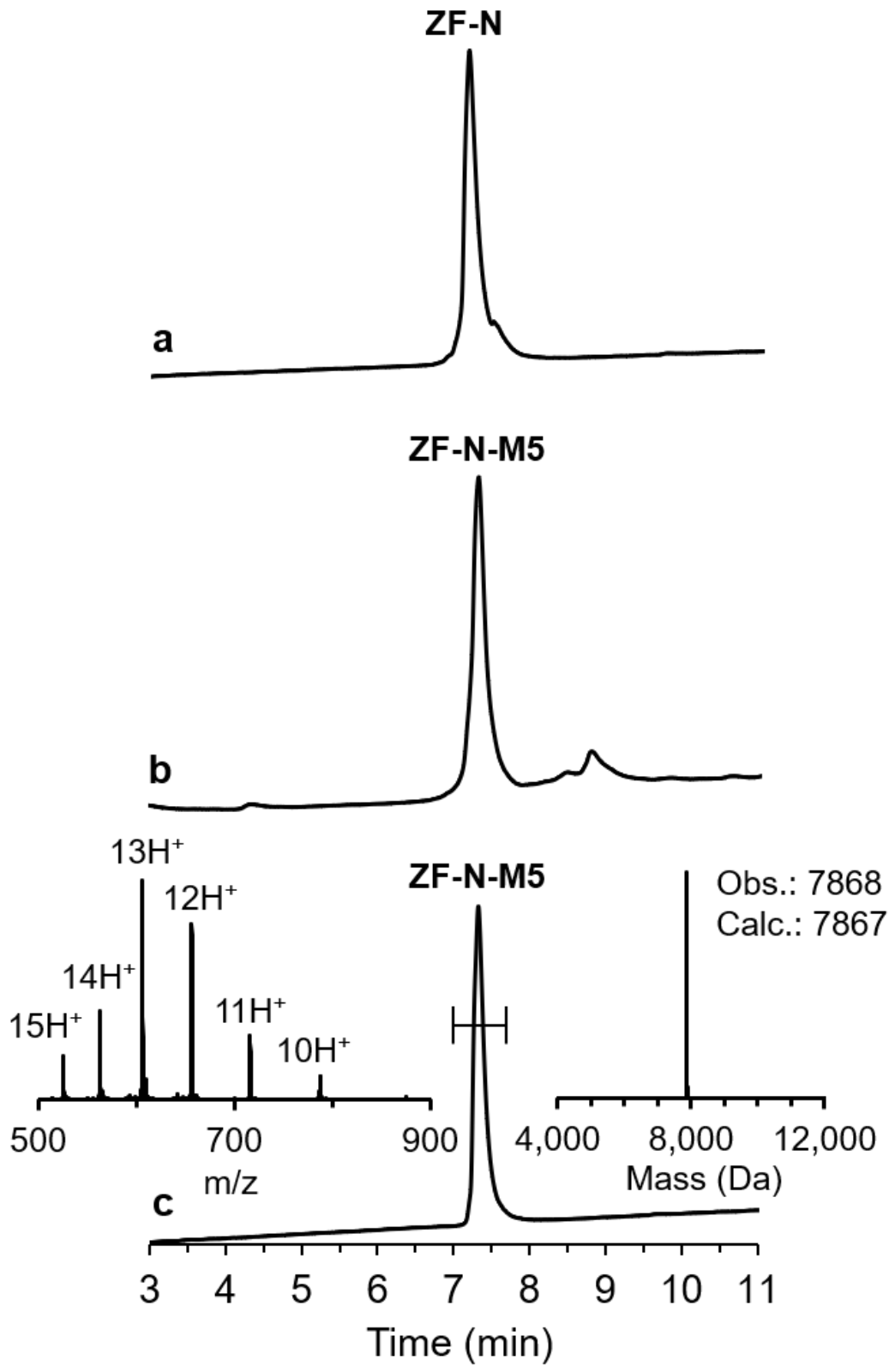

Supplementary Fig. 37 A Schematic representation of site-specific insertion of phospho-Tyr to **ZF-N**. **B** LC-MS analysis of the phospho-Tyr modification of ZF-N. (a) **ZF-N** at t = 0. (b) Arylation at t = 15 min; phospho-Tyr analog of **ZF-N** (**ZF-N-M5**). (c) Isolated **ZF-N-M5**.

7.10 Decaging of ZF-N-M5 protein

The decaging of **ZF-N-M5** to form **ZF-M5** was performed as described in section 7.2.

A

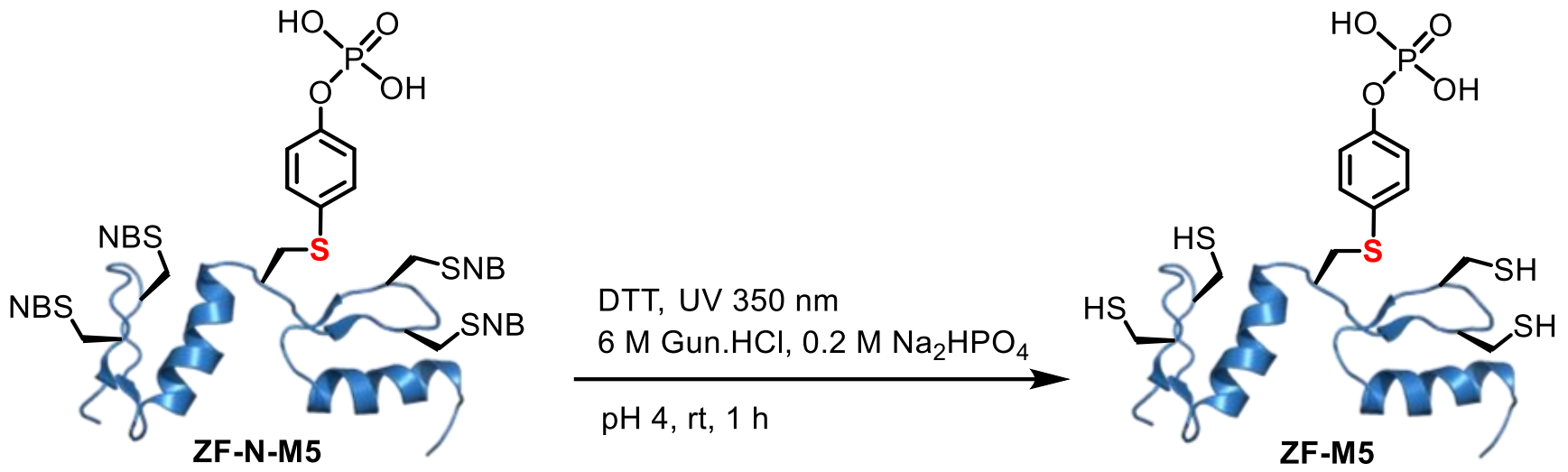

B

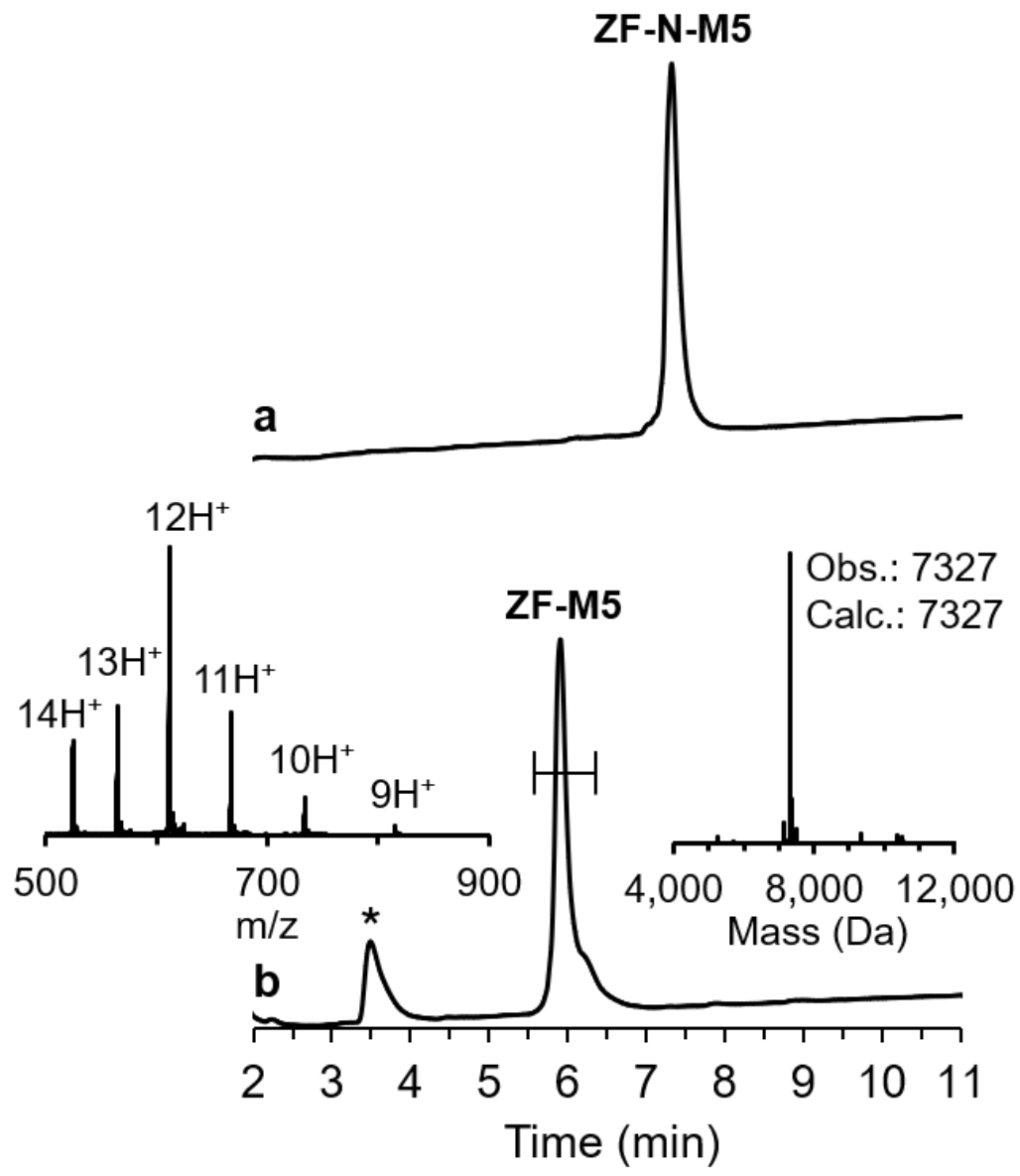

Supplementary Fig. 38 A Schematic representation of decaging of **ZF-N-M5**. **B** LC-MS analysis of decaging of **ZF-N-M5**. (a) **ZF-N-M5** at t = 0. (b) Decaging at t = 1 h; phospho-Tyr analog of **ZF** (**ZF-M5**). (c) Isolated **ZF-M5**. * NB decomposition byproduct.

7.11 Site-specific insertion of nitro-Tyr to ZF-N protein

The Tyr-nitration modification of **ZF-N** protein using Pd(II)OAC-3 was performed as described in section 7.7,[5,6] affording the final product **ZF-N-M6** (25% isolated yield) as a white powder.

A

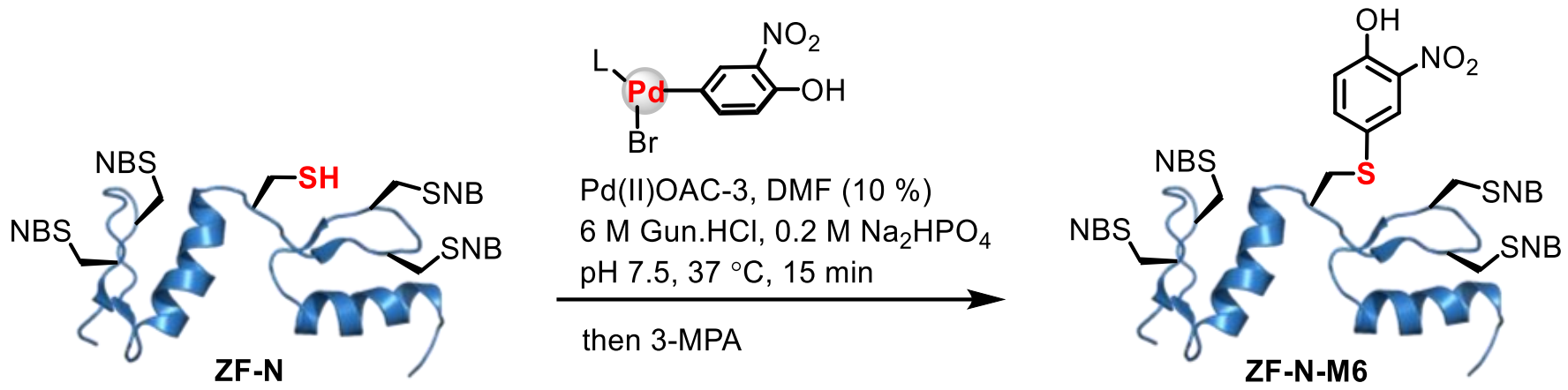

B

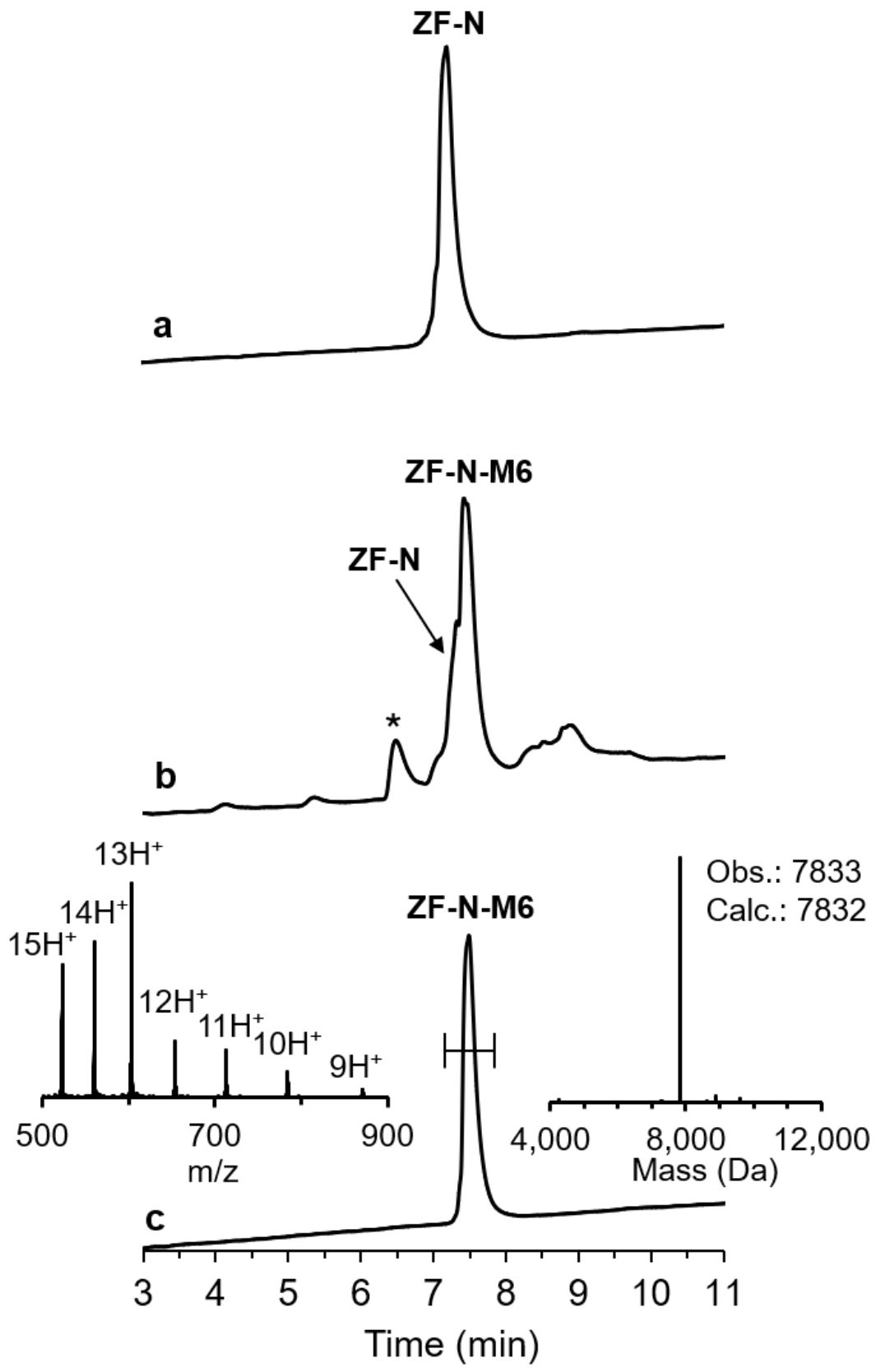

Supplementary Fig. 39 A Schematic representation of site-specific insertion of nitro-Tyr to **ZF-N**. **B** LC-MS analysis of the nitro-Tyr modification of **ZF-N**. (a) **ZF-N** at t = 0. (b) Arylation at t = 15 min; nitro-Tyr analog of **ZF-N** (**ZF-N-M6**). (c) Isolated **ZF-N-M6**. * Pd(II)OAC-3 decomposition byproduct.

7.12 Decaging of ZF-N-M6 protein

The decaging of **ZF-N-M6** to form **ZF-M6** was performed similarly as described in section 7.2.

A

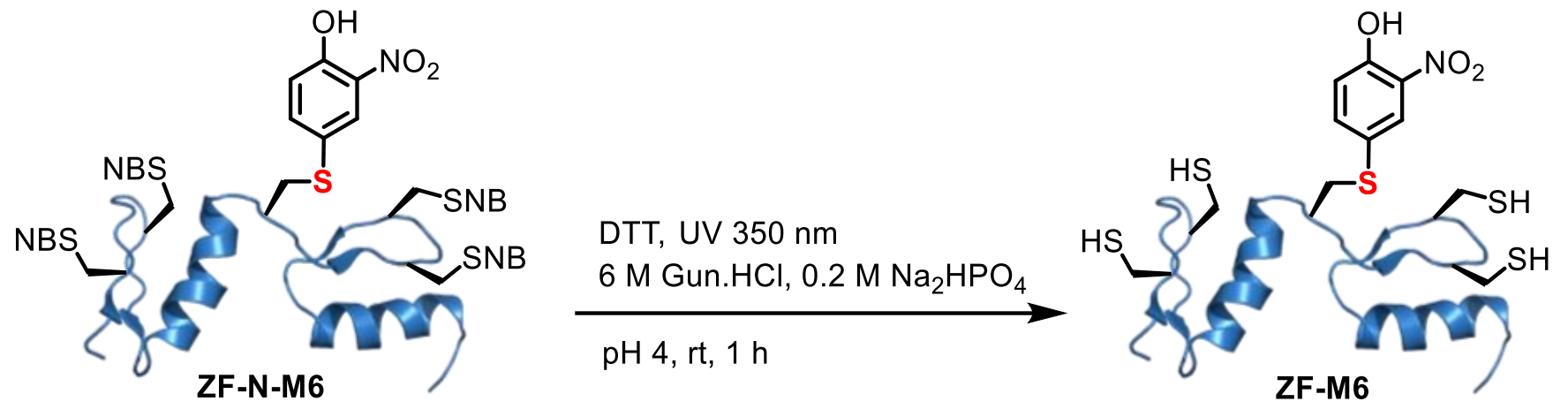

B

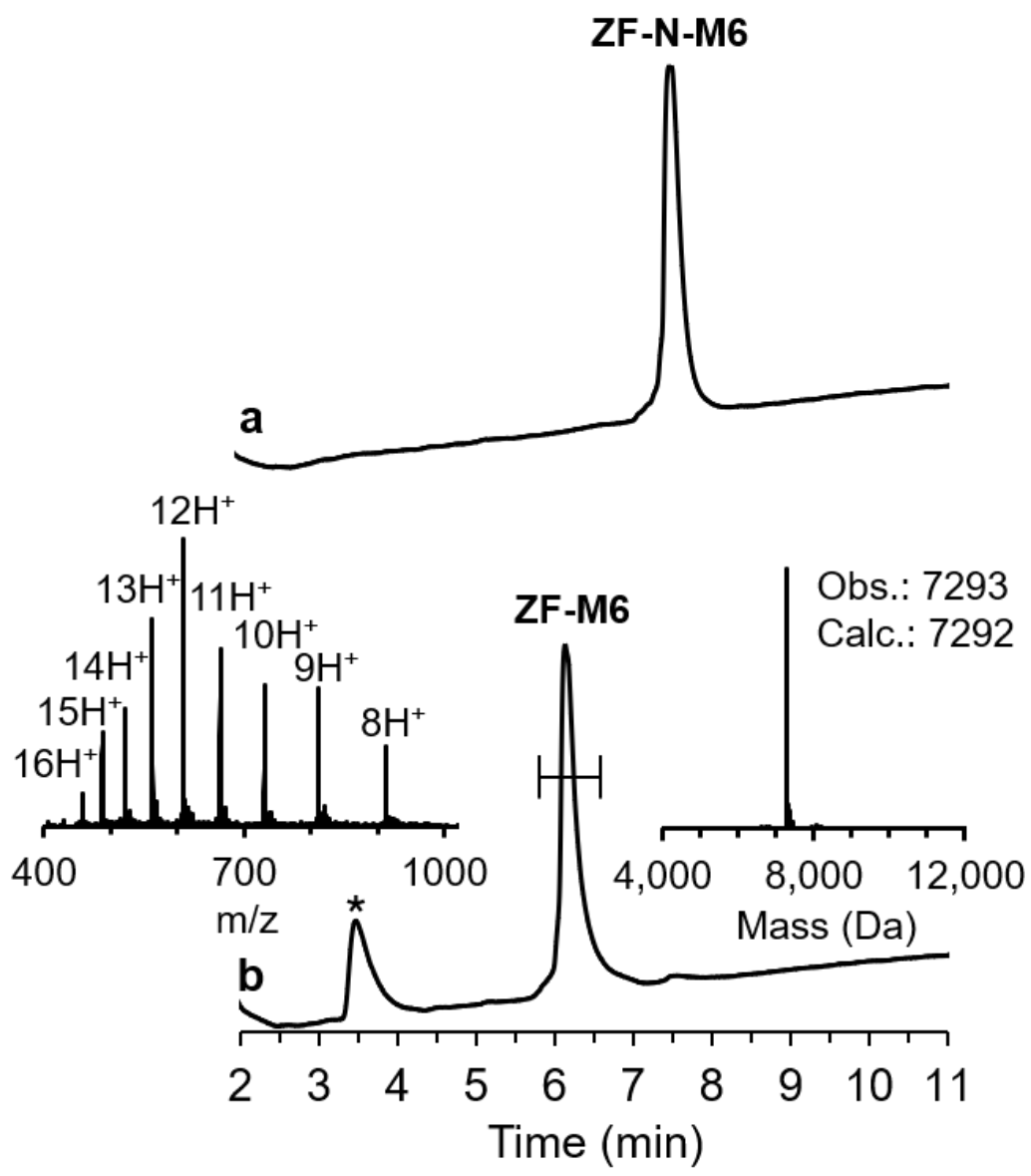

Supplementary Fig. 40 A Schematic representation of decaging of **ZF-N-M6**. **B** LC-MS analysis of decaging of **ZF-N-M6**. (a) **ZF-N-M6** at t = 0. (b) Decaging at t = 1 h; nitro-Tyr analog of **ZF** (**ZF-M6**). (c) Isolated **ZF-M6**. * NB decomposition byproduct.

7.13 Site-specific insertion of acetyl-Lys to ZF protein

The acetyl-Lys modification of **ZF-N** protein was performed as described in section 6.4, affording the final product **ZF-M7** (27% isolated yield) as a white powder.

A

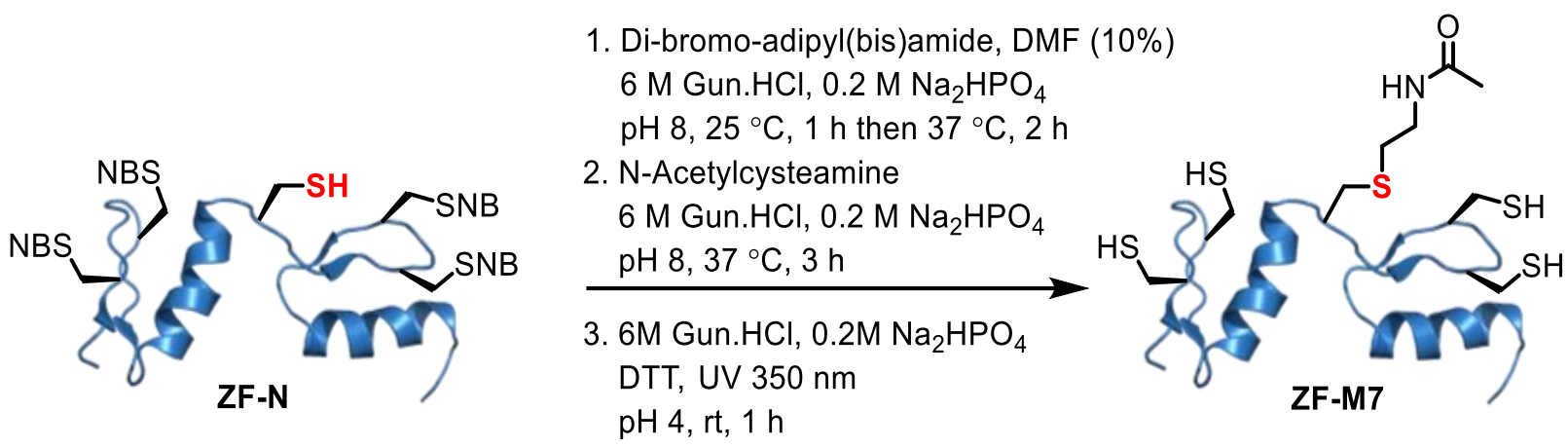

B

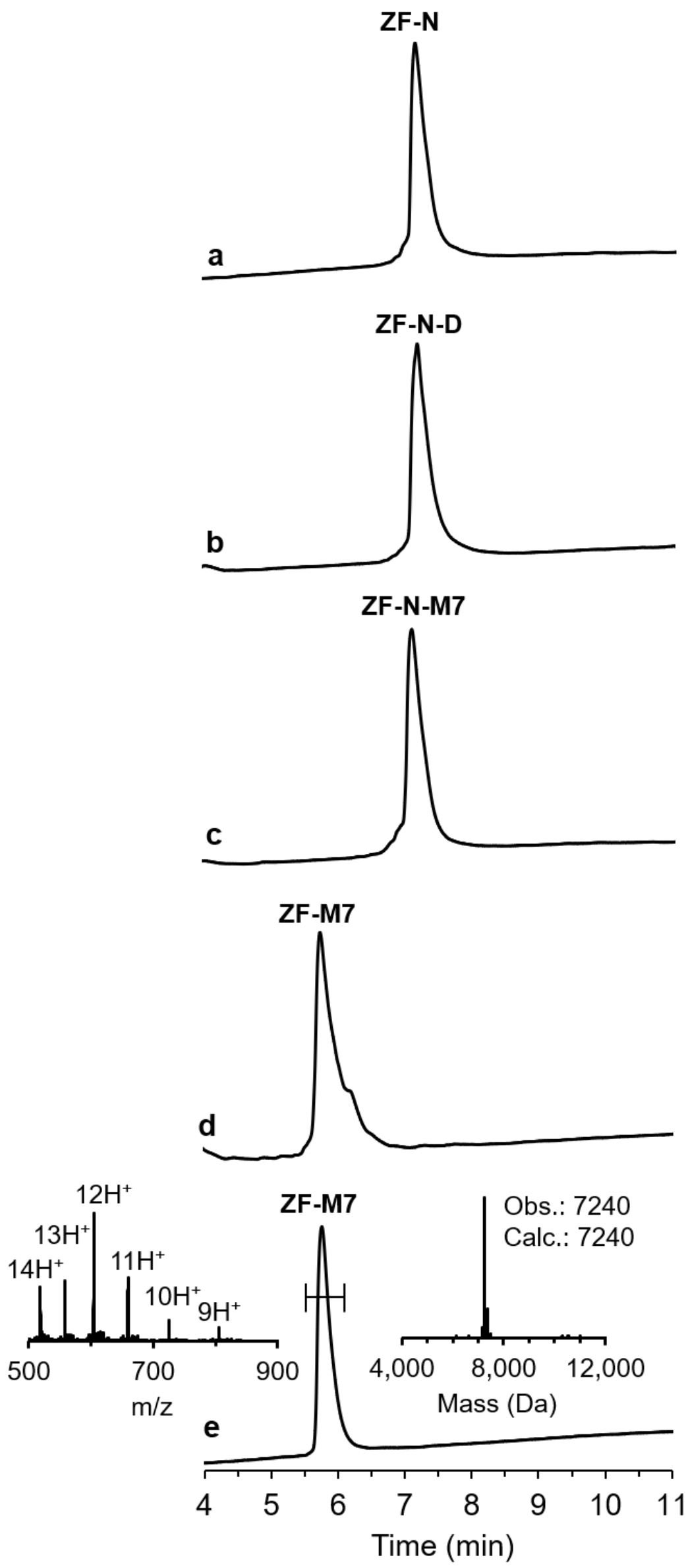

Supplementary Fig. 41 A Schematic representation of site-specific insertion of acetyl-Lys to **ZF-N** and decaging. **B** LC-MS analysis of one-pot acetylation and decaging of **ZF-N**. (a) **ZF-N** at t = 0. (b) Cys to dehydroalanine conversion at t = 3 h; dehydroalanine analog of **ZF-N** (**ZF-N-D**). (c) Michael addition to **ZF-N-D** at t = 6 h; Michael addition product **ZF-N-M7**. (d) Decaging at t = 7 h; acetyl-Lys analog of **ZF** (**ZF-M7**). (e) Isolated **ZF-M7**.

7.14 Site-specific insertion of Lys-mimic to ZF-N protein

The Lys-mimic modification of **ZF-N** protein using 2-bromoethylamine hydrobromide was performed as described in section 7.1, affording the final product **ZF-N-M8** (35% isolated yield) as a white powder.

A

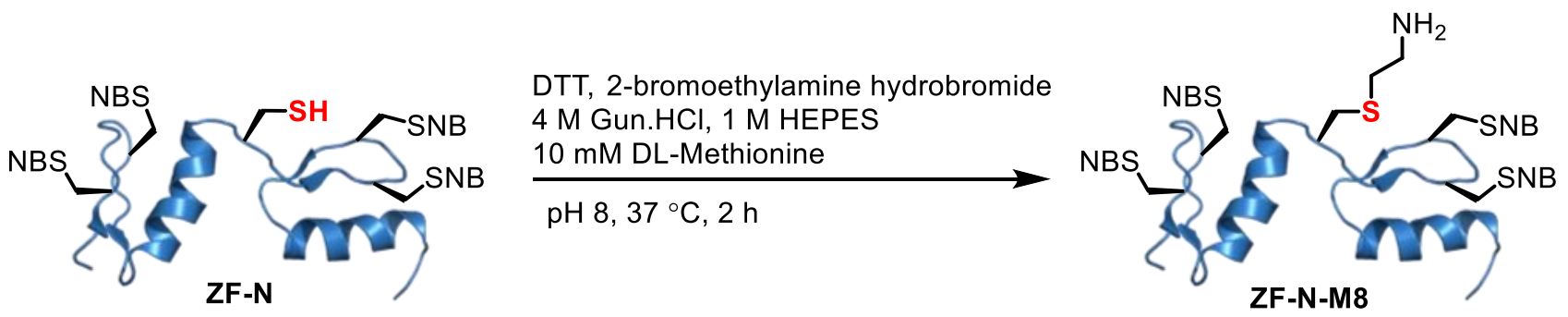

B

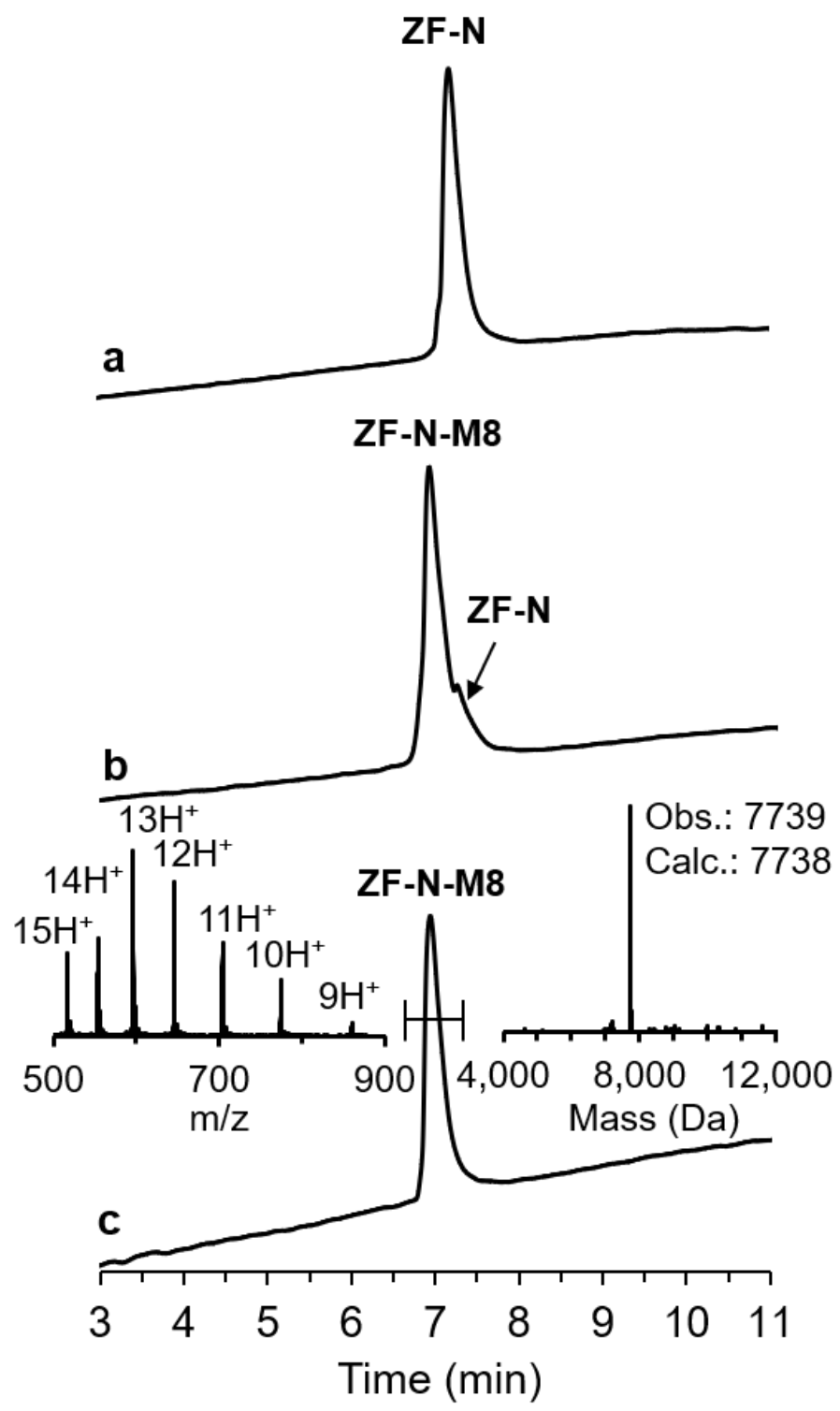

Supplementary Fig. 42 A Schematic representation of site-specific insertion of Lys-mimic to **ZF-N**. **B** LC-MS analysis of Lys-mimic modification of **ZF-N**. (a) **ZF-N** at t = 0. (b) Alkylation at t = 2 h; Lys-mimic of **ZF-N** (**ZF-N-M8**). (c) Isolated **ZF-N-M8**.

7.15 Decaging of ZF-N-M8 protein

The decaging of **ZF-N-M8** to form **ZF-M8** was performed as described in section 7.2.

A

B

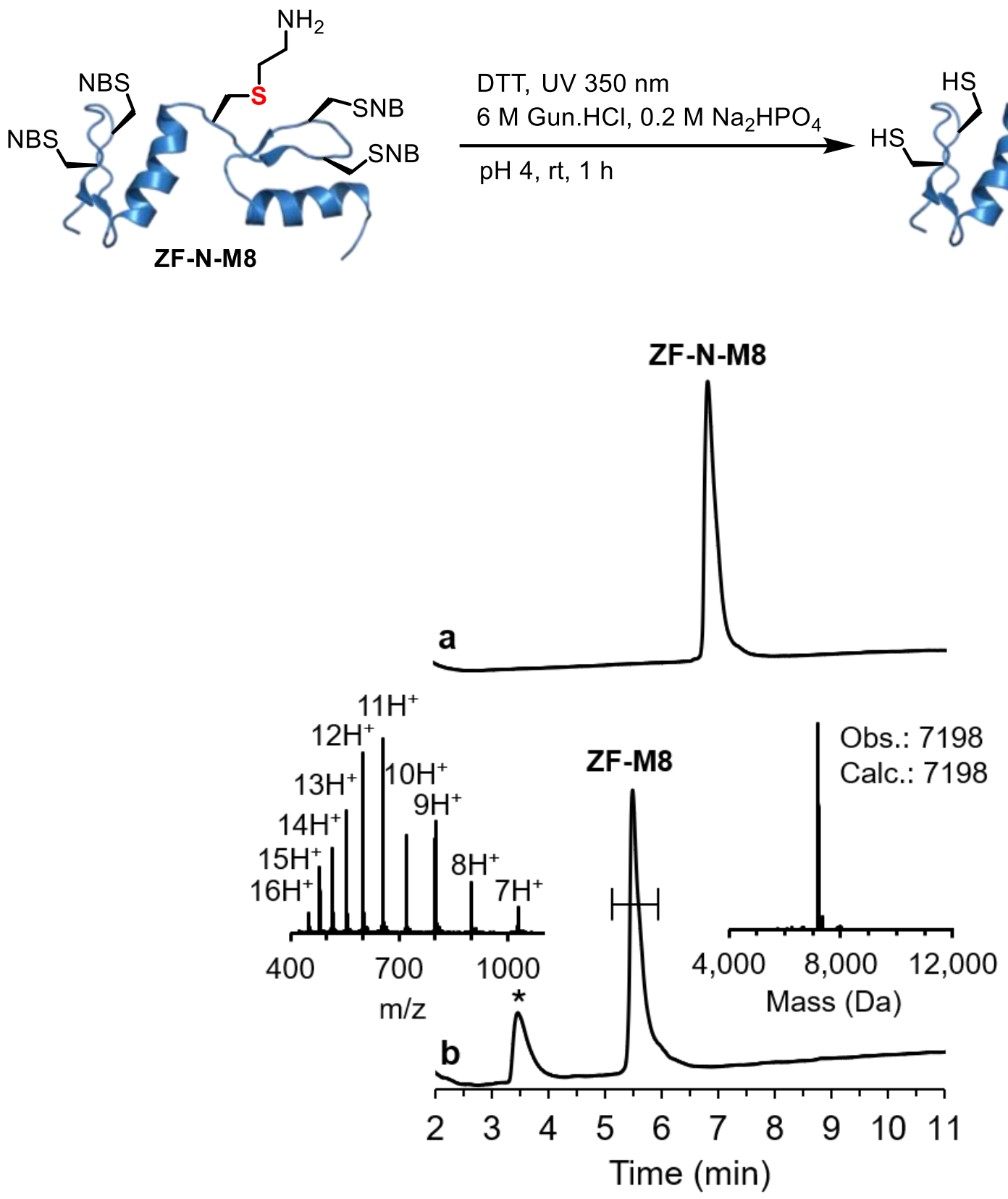

Supplementary Fig. 43 A Schematic representation of decaging of **ZF-N-M8**. **B** LC-MS analysis of decaging of **ZF-N-M8**. (a) **ZF-N-M8** at t = 0. (b) Decaging at t = 1 h; Lys-mimic of **ZF** (**ZF-M8**) and * NB decomposition byproduct.

7.16 Site-specific insertion of Tyr-mimic to ZF-N protein

The Tyr-mimic modification of **ZF-N** protein using Pd(II)OAC-4 was performed as described in section 7.7,[11] affording the final product **ZF-N-M9** (35% isolated yield) as a white powder.

A

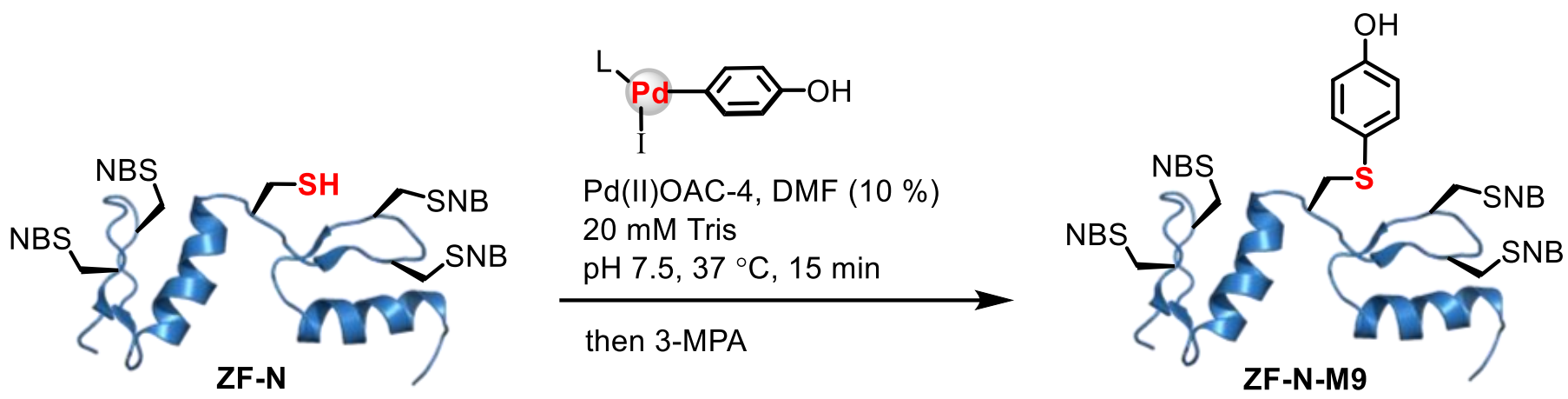

B

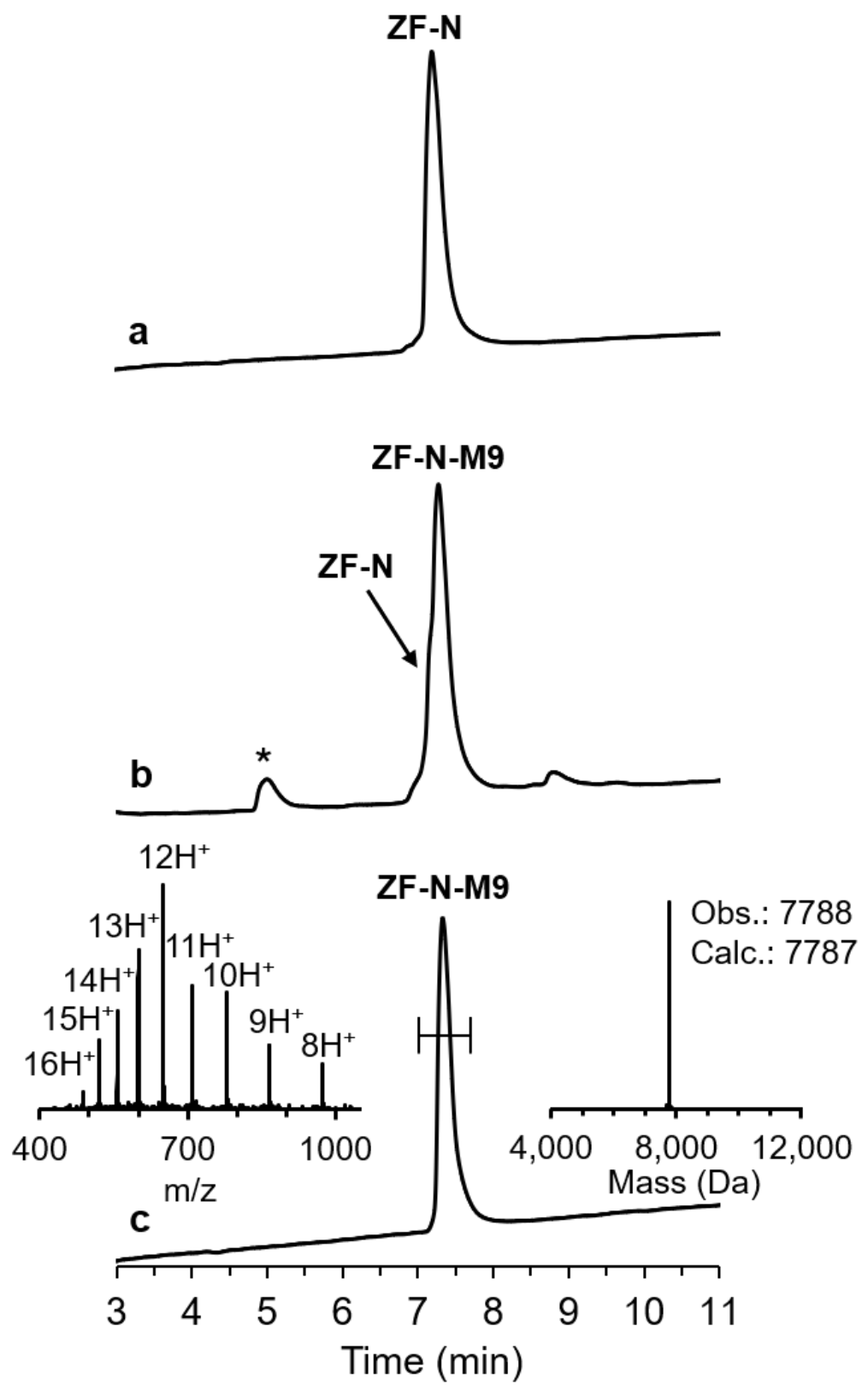

Supplementary Fig. 44 A Schematic representation of site-specific insertion of Tyr-mimic to **ZF-N**. **B** LC-MS analysis of the Tyr-mimic modification of **ZF-N**. (a) **ZF-N** at t = 0. (b) Arylation at t = 15 min; Tyr-mimic of **ZF-N** (**ZF-N-M9**). (c) Isolated **ZF-N-M9**. * Pd(II)OAC-4 decomposition byproduct.

7.17 Decaging of ZF-N-M9 protein

The decaging of **ZF-N-M9** to form **ZF-M9** was performed as described in section 7.2.

A

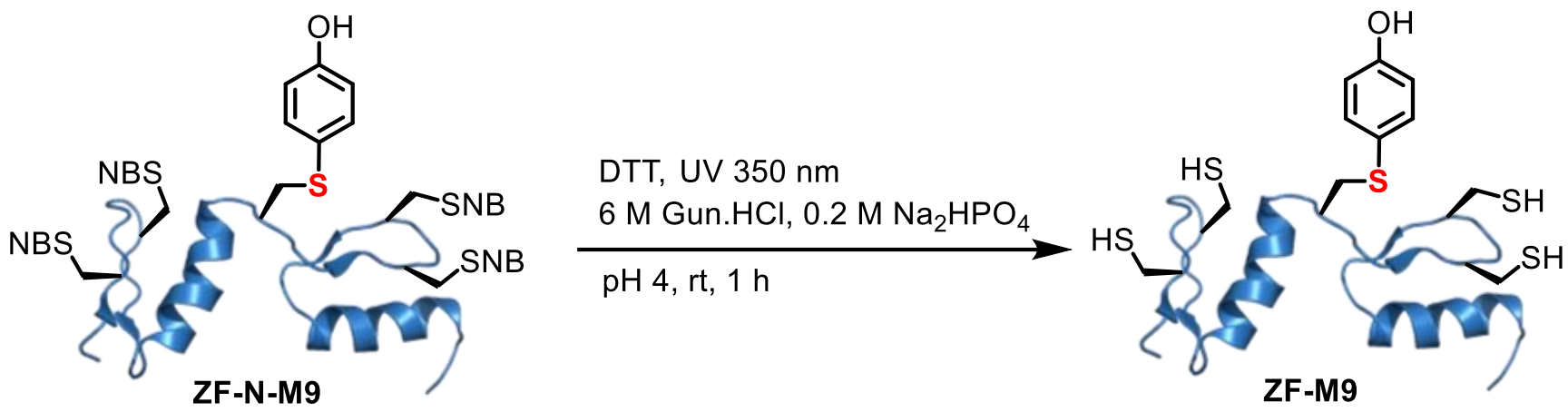

B

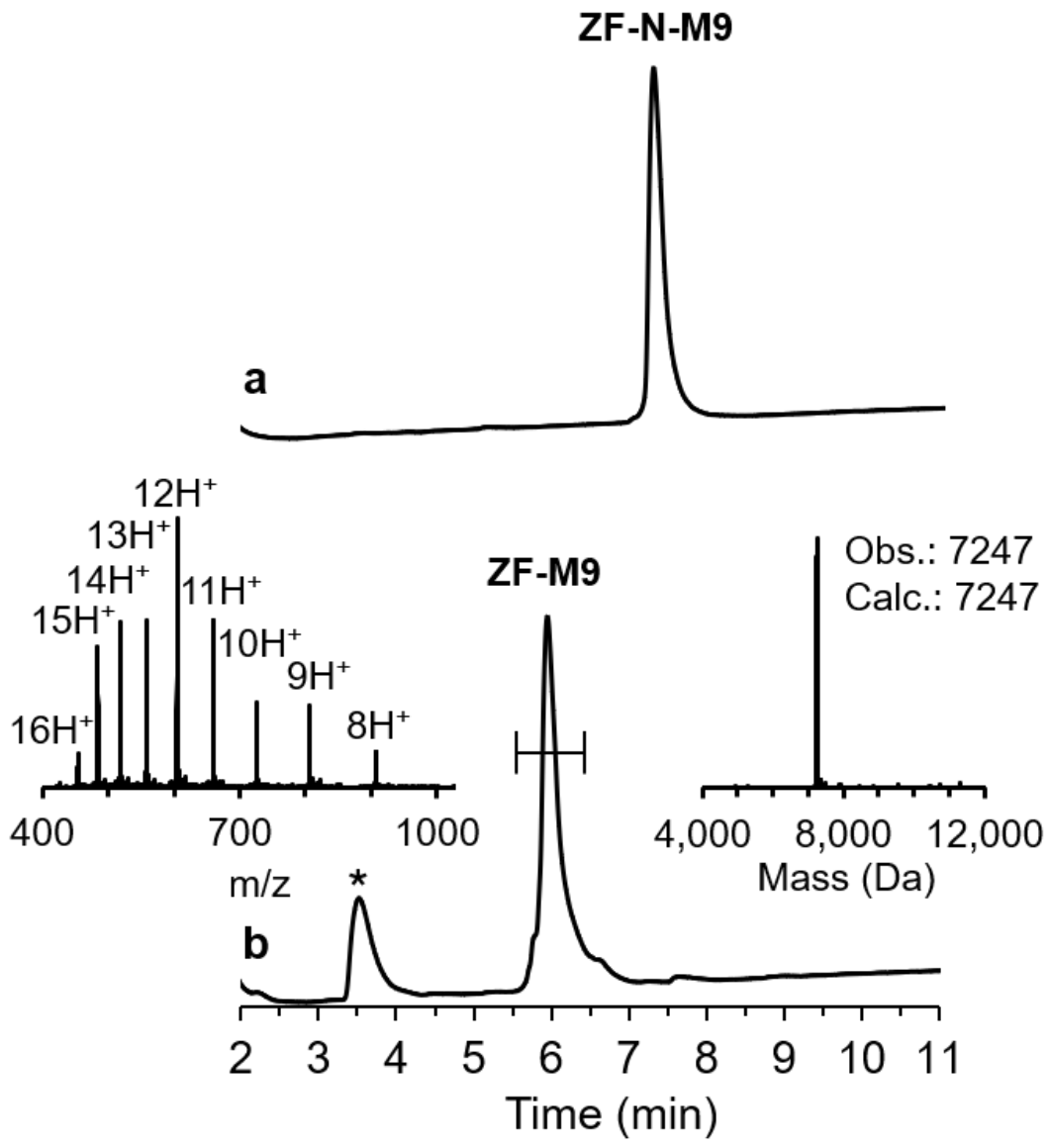

Supplementary Fig. 45 A Schematic representation of decaging of **ZF-N-M9**. **B** LC-MS analysis of decaging of **ZF-N-M9**. (a) **ZF-N-M9** at t = 0. (b) Decaging at t = 1 h; Tyr-mimic of **ZF** (**ZF-M9**) and * NB decomposition product.

7.18 Site-specific insertion of Phe-mimic to ZF-N protein

The Phe-mimic modification of **ZF-N** protein using Pd(II)OAC-5 was performed as described in section 7.7,[11] affording the final product **ZF-N-M10** (20% isolated yield) as a white powder.

A

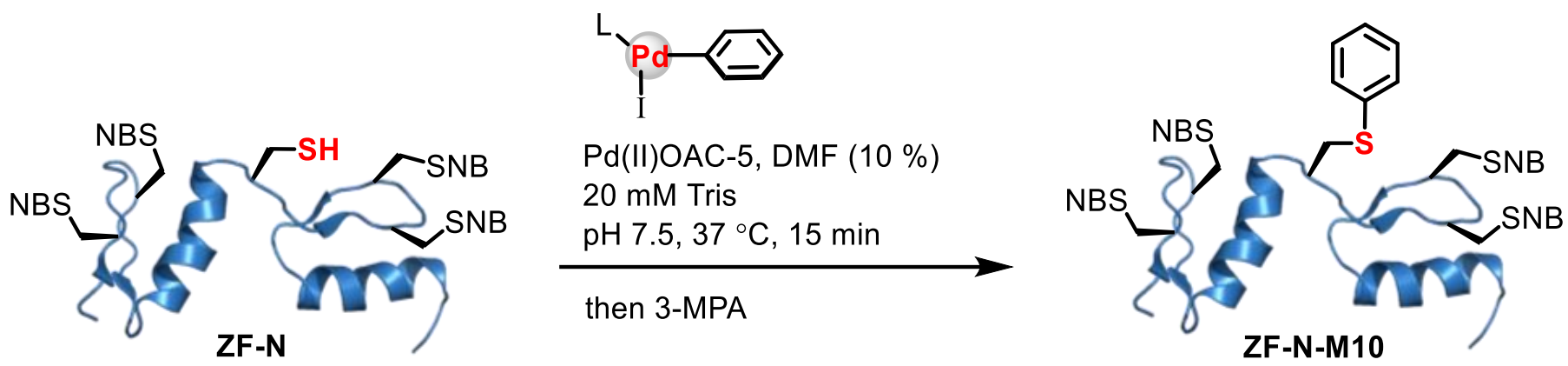

B

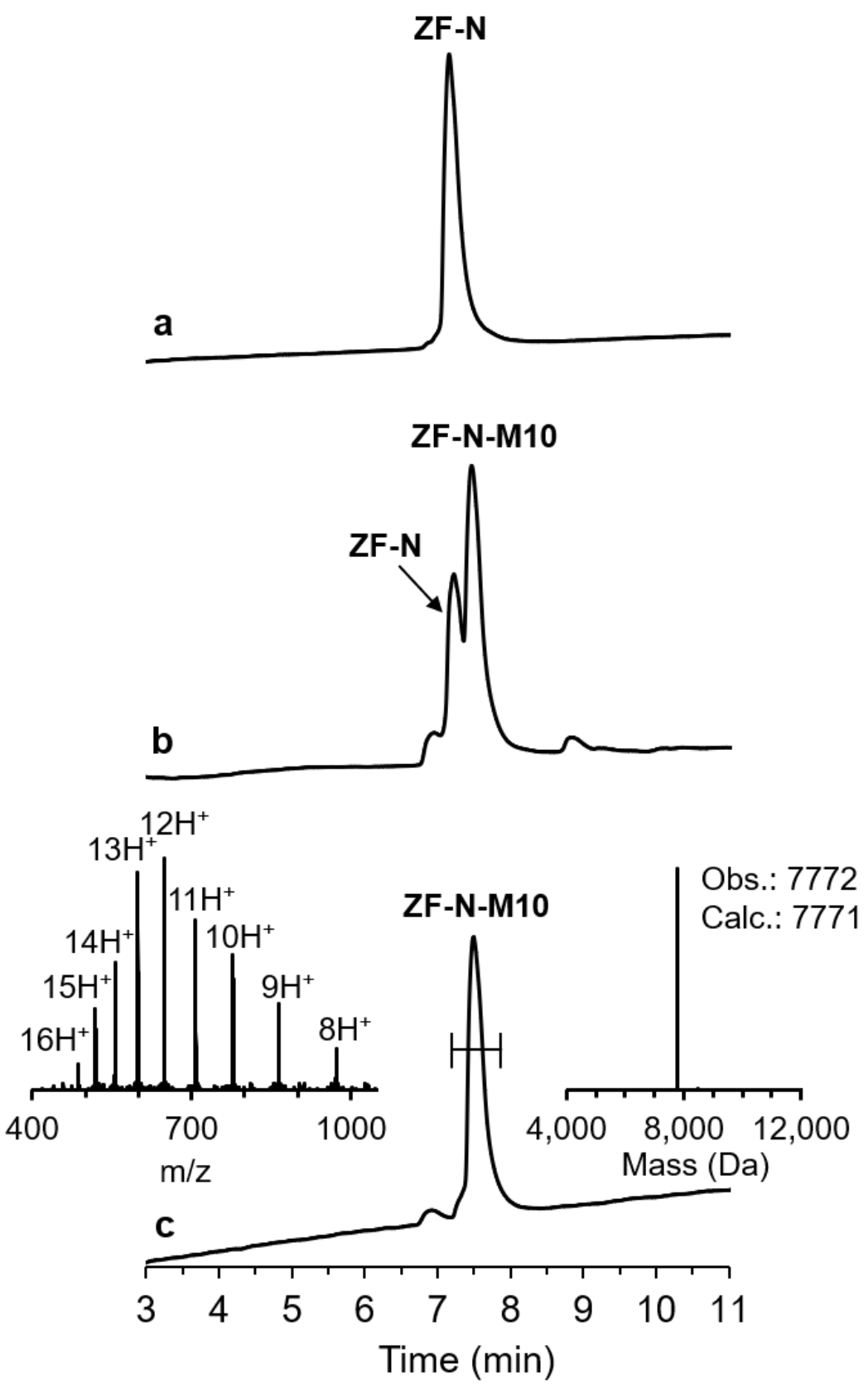

Supplementary Fig. 46 A Schematic representation of site-specific insertion of Phe-mimic to **ZF-N**. **B** LC-MS analysis of the Phe-mimic modification of **ZF-N.** (a) **ZF-N** at t = 0. (b) Arylation at t = 15 min; Phe-mimic of **ZF-N** (**ZF-N-M10**). (c) Isolated **ZF-N-M10**.

7.19 Decaging of ZF-N-M10 protein

The decaging of **ZF-N-M10** to form **ZF-M10** was performed as described in section 7.2.

A

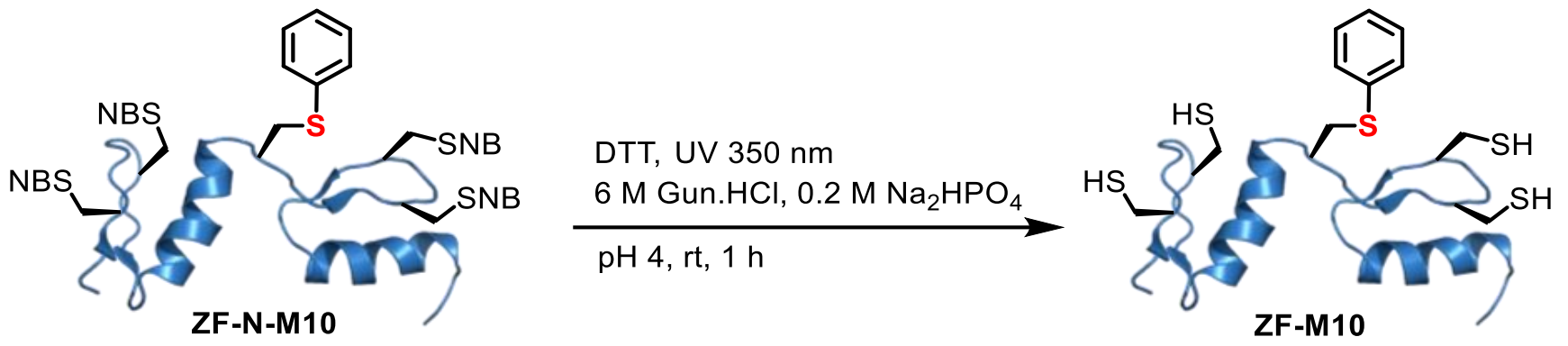

B

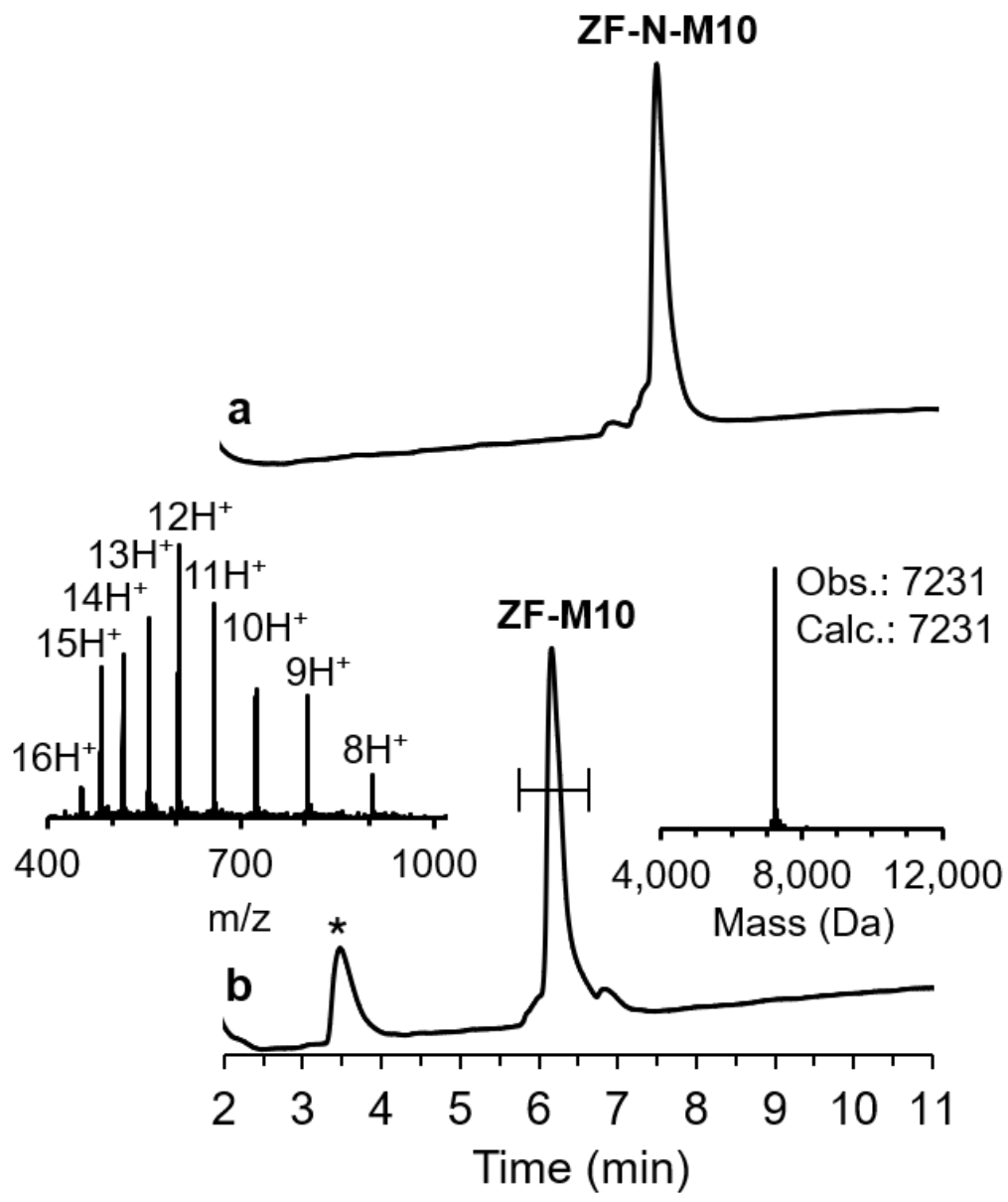

Supplementary Fig. 47 A Schematic representation of decaging of **ZF-N-M10**. **B** LC-MS analysis of decaging of **ZF-N-M10**. (a) **ZF-N-M10** at t = 0. (b) Decaging at t = 1 h; Phe-mimic of **ZF** (**ZF-M10**) and * NB decomposition byproduct.

7.20 Site-specific insertion of Gln-mimic to ZF-N protein

In a 1.5 mL Eppendorf tube, **ZF-N** (2.0 mg, 1.0 equiv., 0.26 µmol, 0.5 mM) was dissolved in 6 M Gun.HCl, 0.2 M Na_2HPO_4 buffer (0.52 mL). To this solution, 2-bromoacetamide (0.36 mg, 10.0 equiv., 2.6 µmol, 0.26 M; based on **ZF-N**) dissolved in the same buffer (10 µL) was added. The pH was adjusted to 7.5 using 5 *N* NaOH. The mixture was then incubated for 30 min at 37 °C. The reaction was monitored by LC-MS (Method A described in Section 1.2). After completion of the reaction, the reaction mixture was purified using RP-HPLC (Method C described in Section 1.3), affording the final product **ZF-N-M11** (46% isolated yield) as a white powder.

A

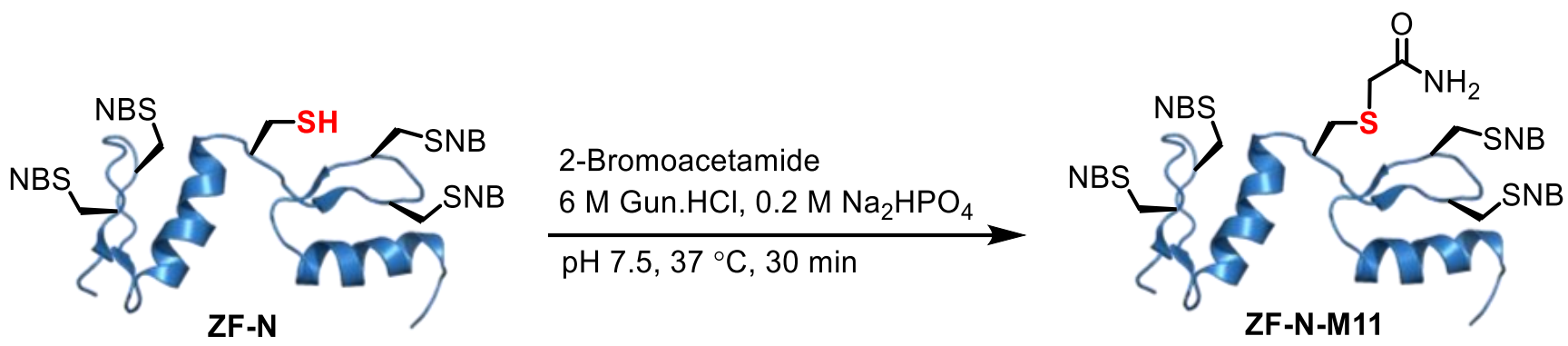

B

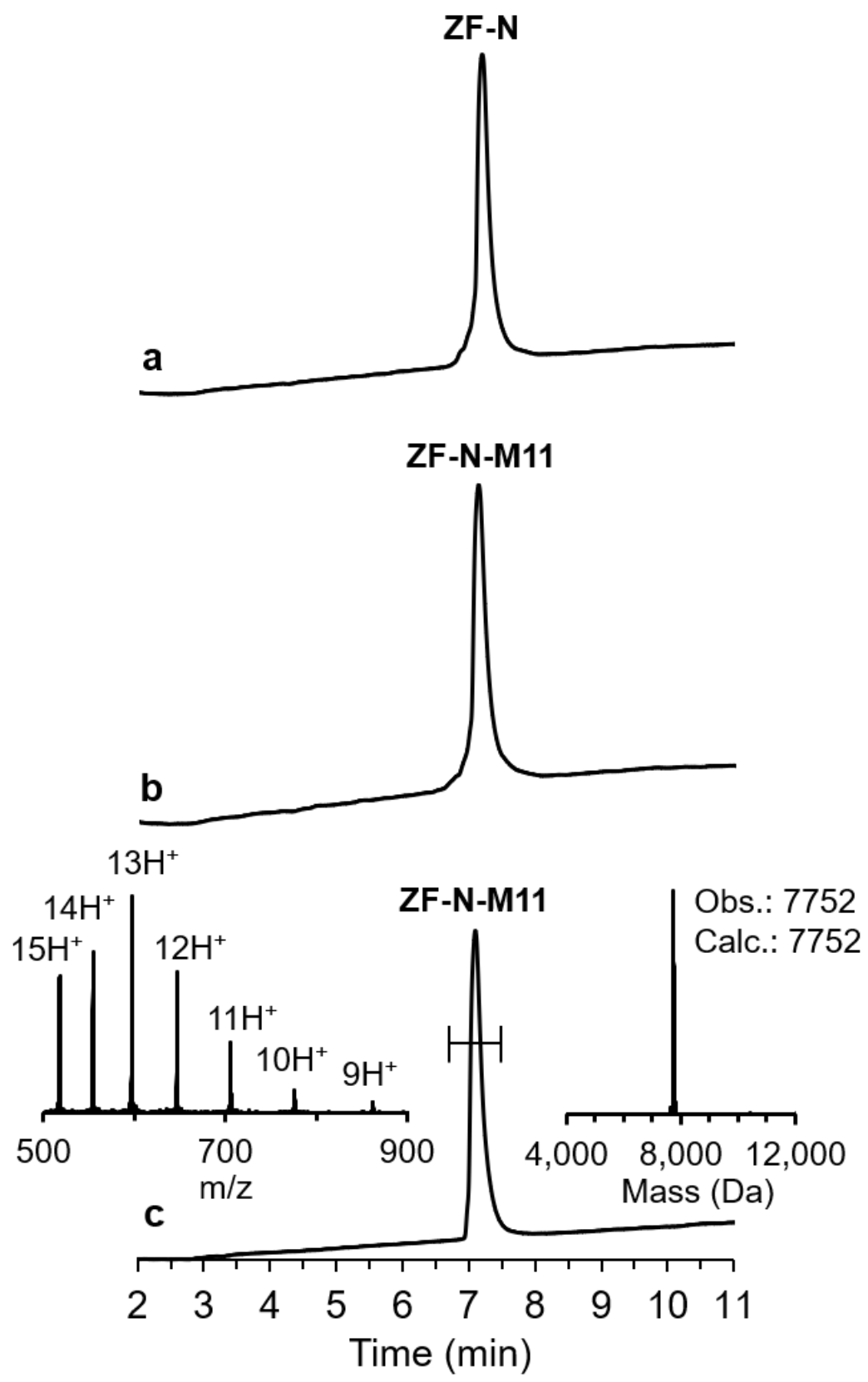

Supplementary Fig. 48 A Schematic representation of site-specific insertion of Gln-mimic to **ZF-N**. **B** LC-MS analysis of the Gln-mimic modification of **ZF-N**. (a) **ZF-N** at t = 0. (b) Alkylation at t = 30 min; Gln-mimic of **ZF-N** (**ZF-N-M11**). (c) Isolated **ZF-N-M11**.

7.21 Decaging of ZF-N-M11 protein

The decaging of **ZF-N-M11** to form **ZF-M11** was performed as described in section 7.2.

A

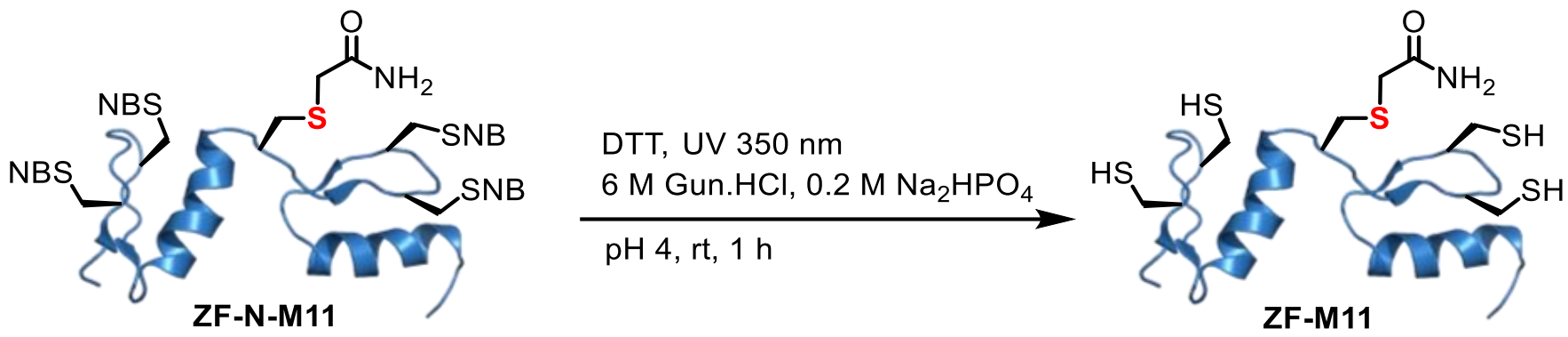

B

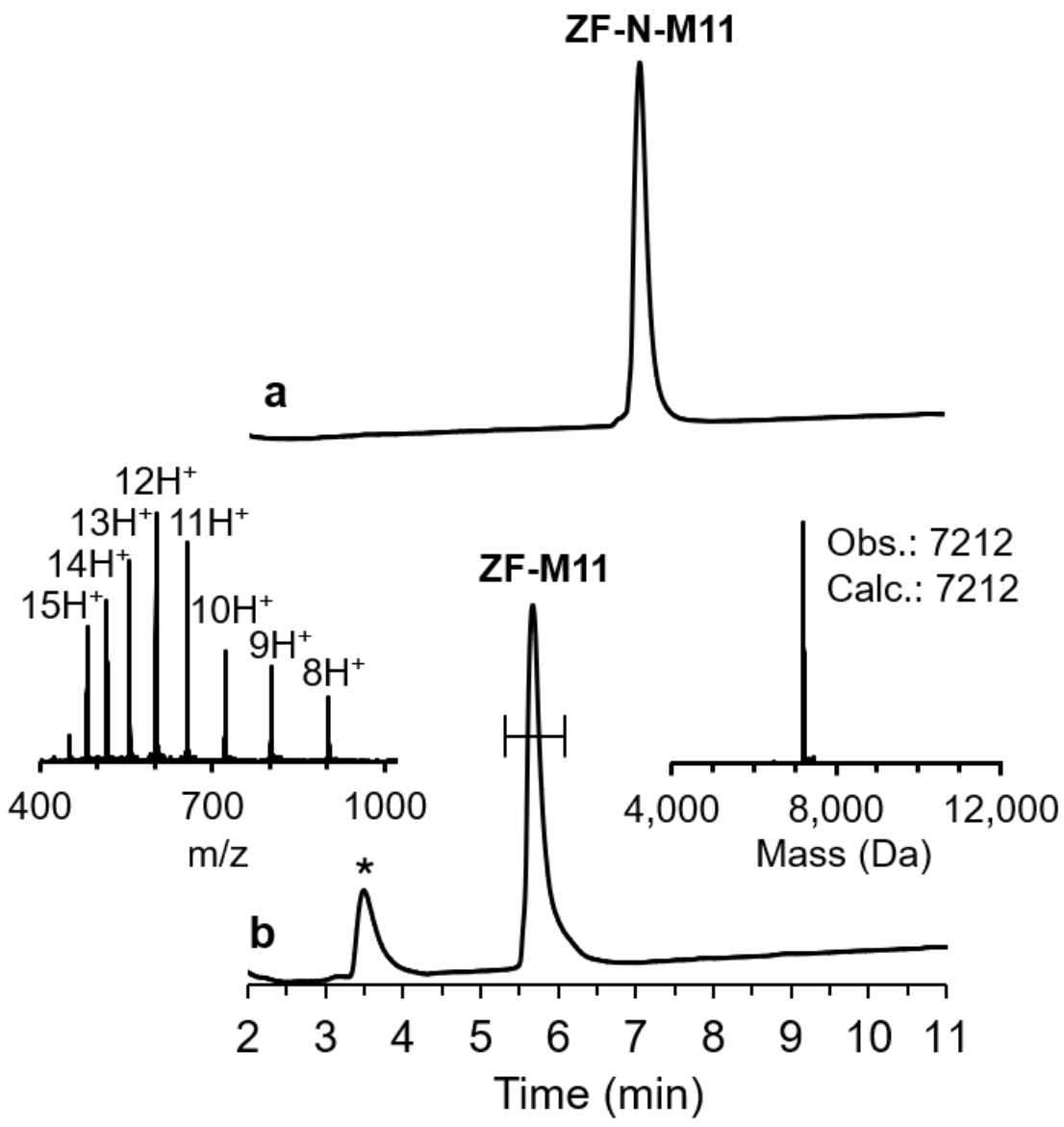

Supplementary Fig. 49 A Schematic representation of decaging of **ZF-N-M11**. **B** LC-MS analysis of decaging of **ZF-N-M11**. (a) **ZF-N-M11** at t = 0. (b) Decaging at t = 1 h; Gln-mimic of **ZF** (**ZF-M11**) and * NB decomposition byproduct.

7.22 Site-specific insertion of Glu-mimic to ZF-N protein

The Glu-mimic modification of **ZF-N** using 2-bromoacetic acid was performed as described in section 7.20, affording the final product, **ZF-N-M12** (46% isolated yield), as a white powder.

A

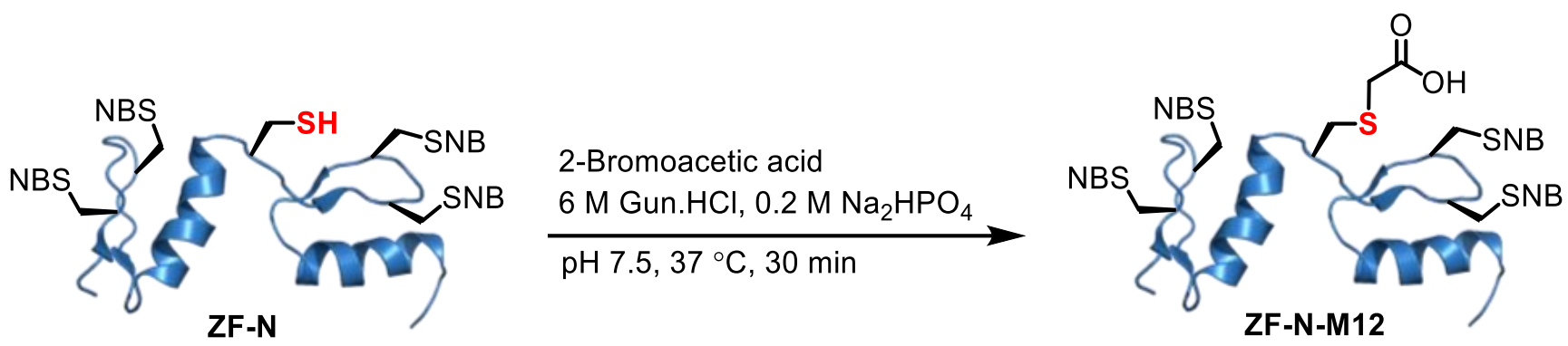

B

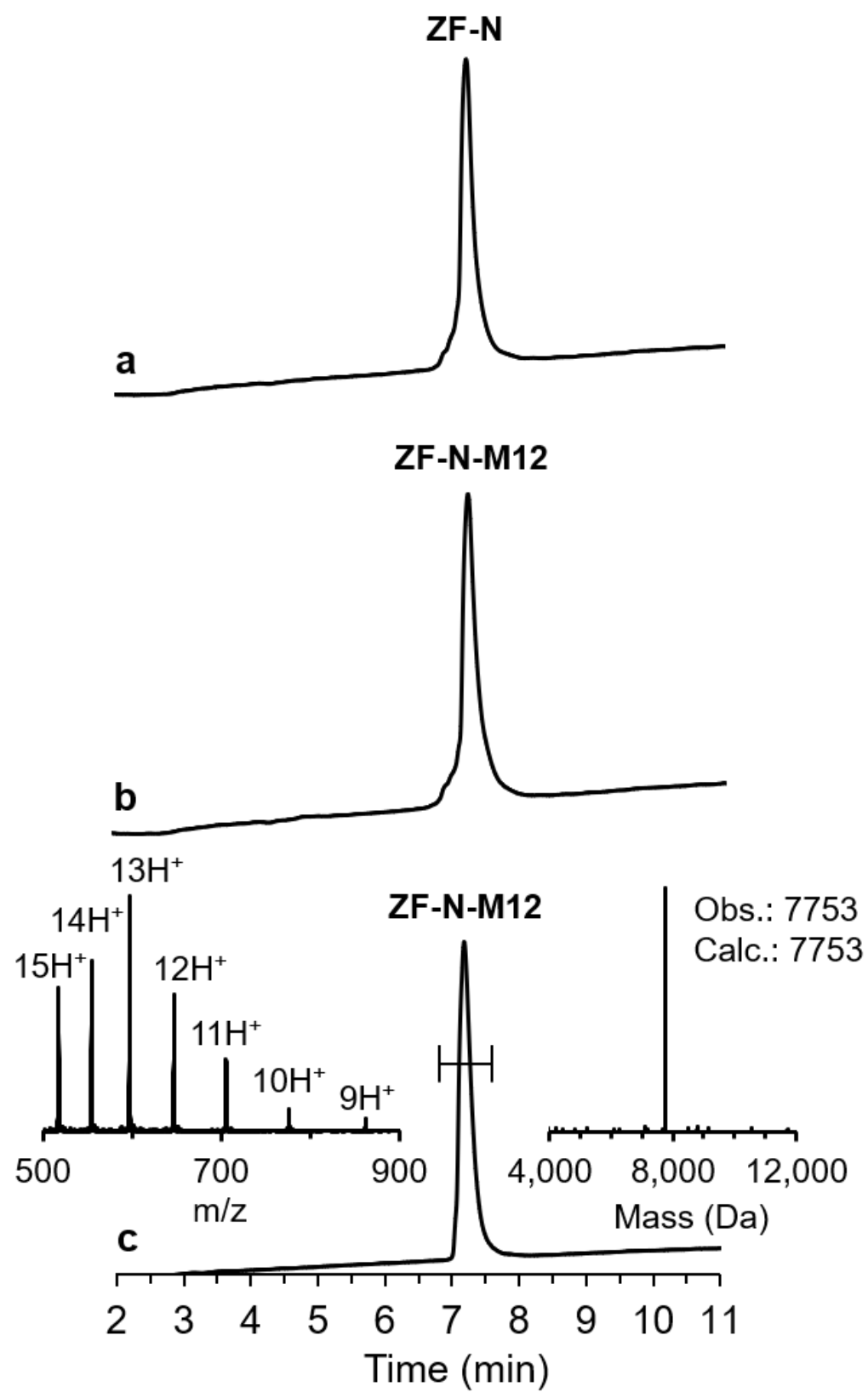

Supplementary Fig. 50 A Schematic representation of site-specific insertion of Glu-mimic to **ZF-N**. **B** LC-MS analysis of the Glu-mimic modification **ZF-N.** (a) **ZF-N** at t = 0. (b) Alkylation at t = 30 min; Glu-mimic of **ZF-N** (**ZF-N-M12**). (c) Isolated **ZF-N-M12**.

7.23 Decaging of ZF-N-M12 protein

The decaging of **ZF-N-M12** to form **ZF-M12** was performed as described in section 7.2.

A

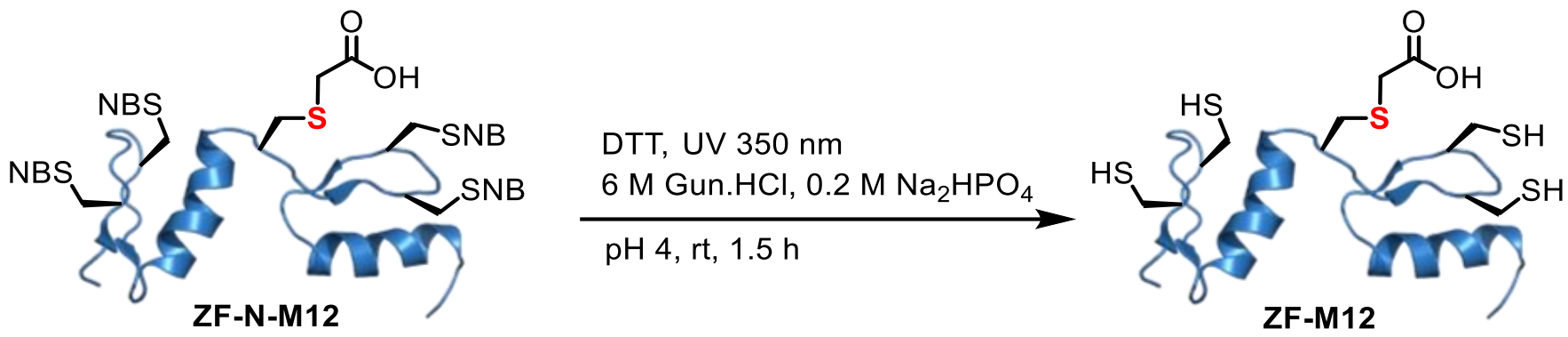

B

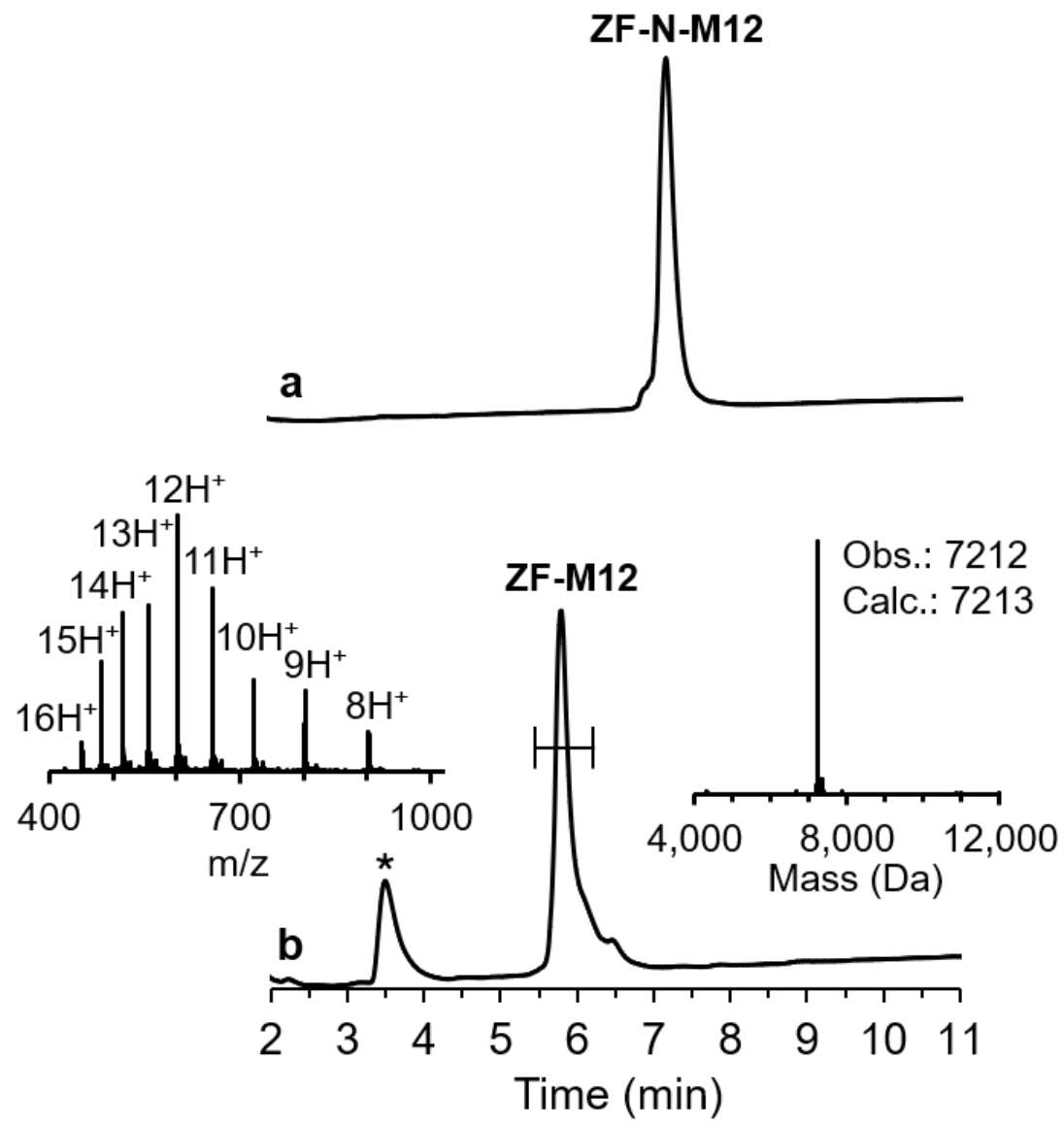

Supplementary Fig. 51 A Schematic representation of decaging of **ZF-N-M12**. **B** LC-MS analysis of decaging of **ZF-N-M12**. (a) **ZF-N-M12** at t = 0. (b) Decaging at t = 1 h; Glu-mimic of **ZF** (**ZF-M12**) and * NB decomposition byproduct.

7.24 Site-specific insertion of biotin tag to ZF-N protein

The biotin modification of **ZF-N** using biotin polyethyleneoxide iodoacetamide was performed as described in section 7.20, affording the final product **ZF-N-M13** (75% isolated yield) as a white powder.

A

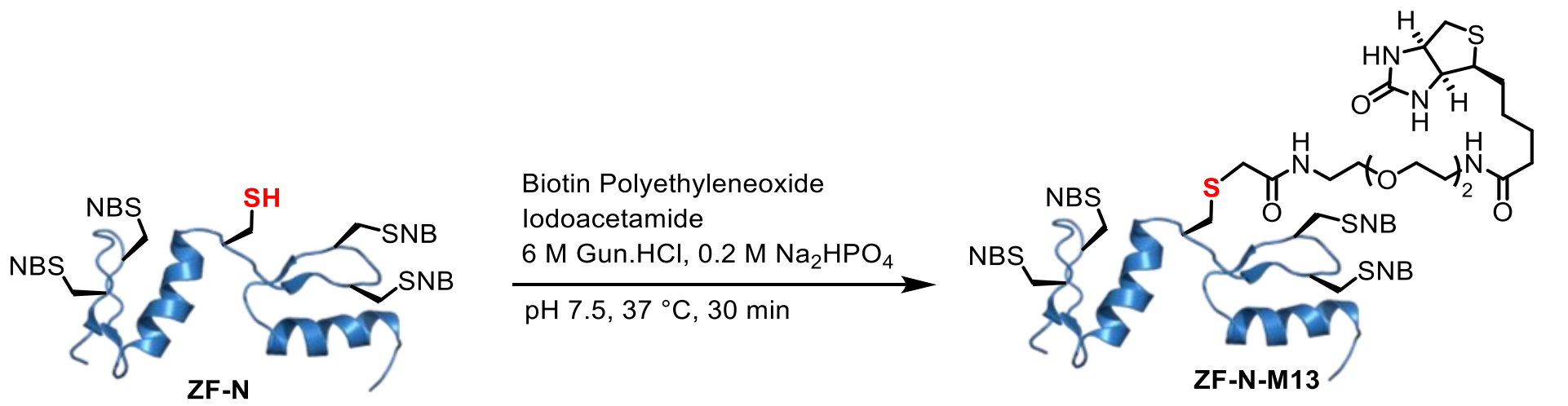

B

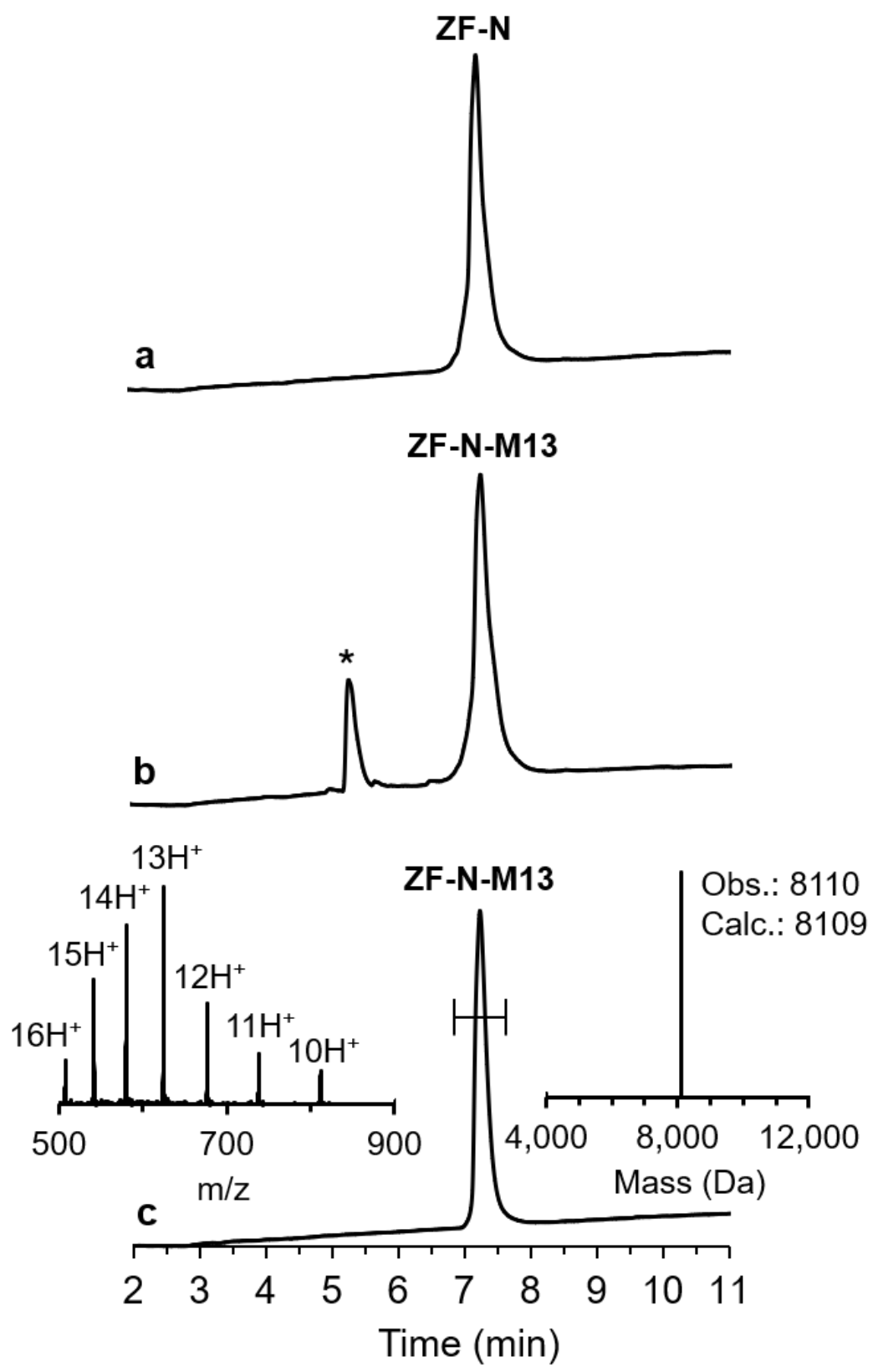

Supplementary Fig. 52 A Schematic representation of site-specific insertion of a biotin tag to **ZF-N**. **B** LC-MS analysis of biotin modification of **ZF-N** . (a) **ZF-N** at t = 0. (b) Alkylation at t = 30 min; **ZF-N** with biotin tag (**ZF-N-M13**). (c) Isolated **ZF-N-M13**. * Excess amount of biotin polyethyleneoxide iodoacetamide.

7.25 Decaging of ZF-N-M13 protein

The decaging of **ZF-N-M13** was performed as described in section 7.2, affording the final product **ZF-M13** (62% isolated yield) as a white powder after purification by RP-HPLC (Method C, Section 1.3).

A

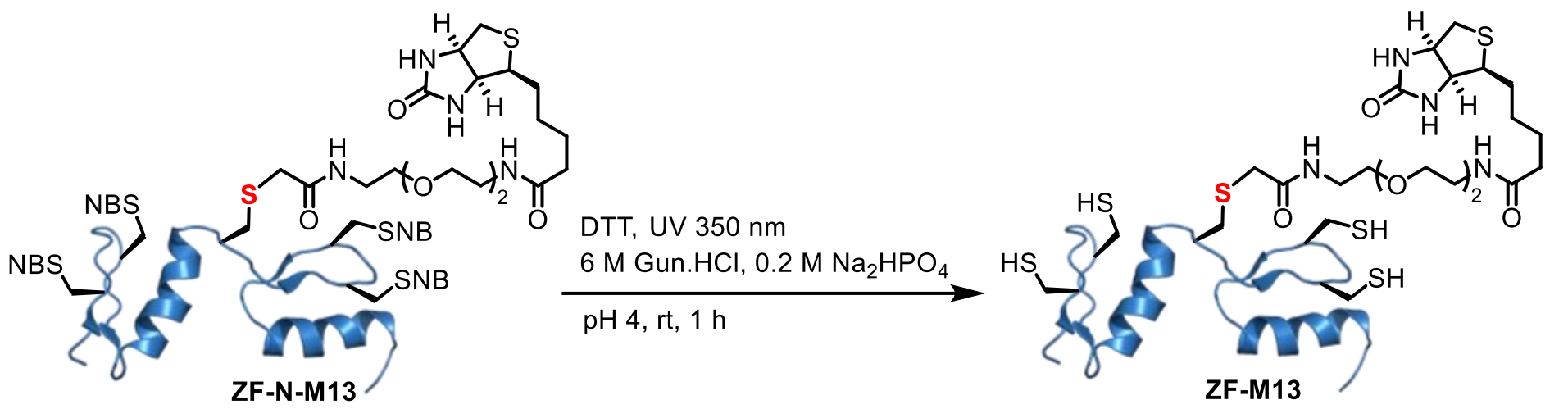

B

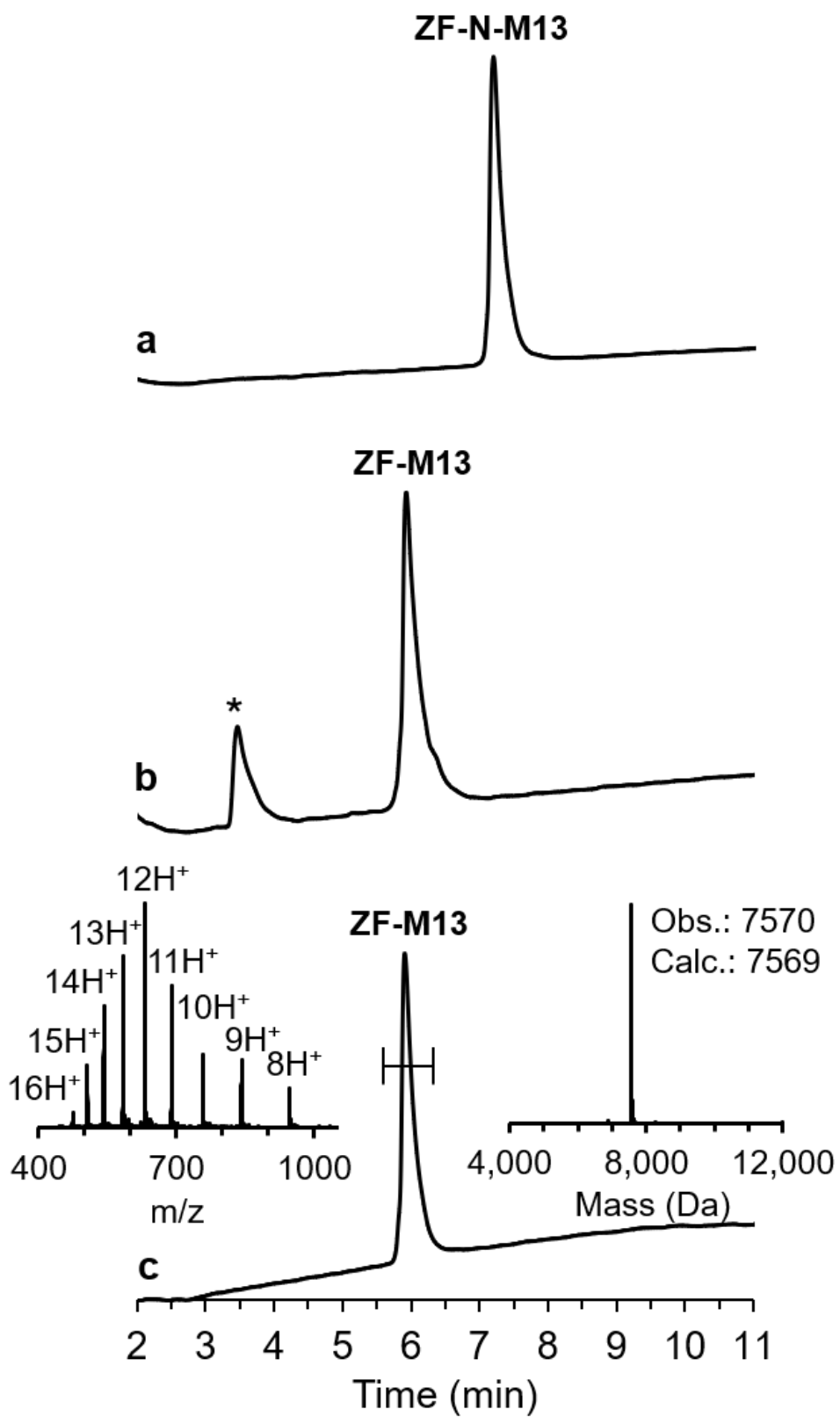

Supplementary Fig. 53 A Schematic representation of decaging of **ZF-N-M13**. **B** LC-MS analysis of decaging of **ZF-N-M13**. (a) **ZF-N-M13** at t = 0. (b) Decaging at t = 1 h; **ZF** with biotin tag (**ZF-M13**). (c) Isolated **ZF-M13**. * NB decomposition byproduct.

7.26 Site-specific insertion of PEG tag to ZF-N protein

The PEG modification of **ZF-N** protein using 1-(2-bromoethoxy)-2-(2-methoxyethoxy)ethane was performed as described in section 7.1, affording the final product **ZF-N-M14** (35% isolated yield) as a white powder.

A

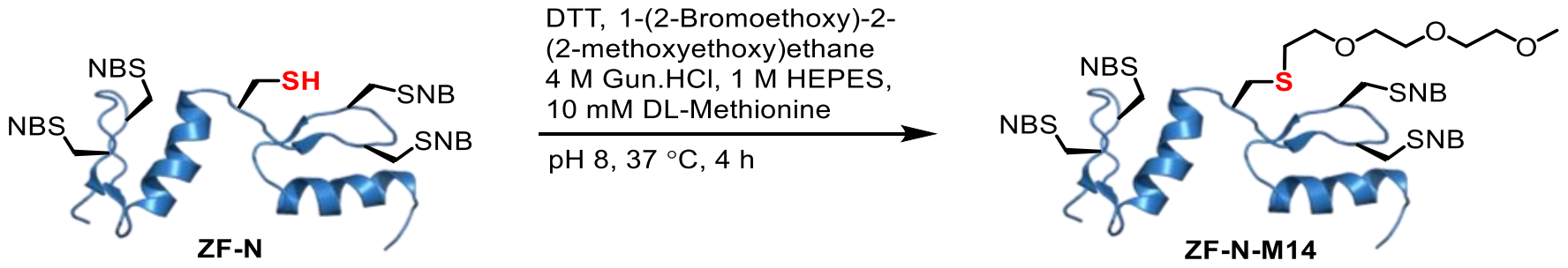

B

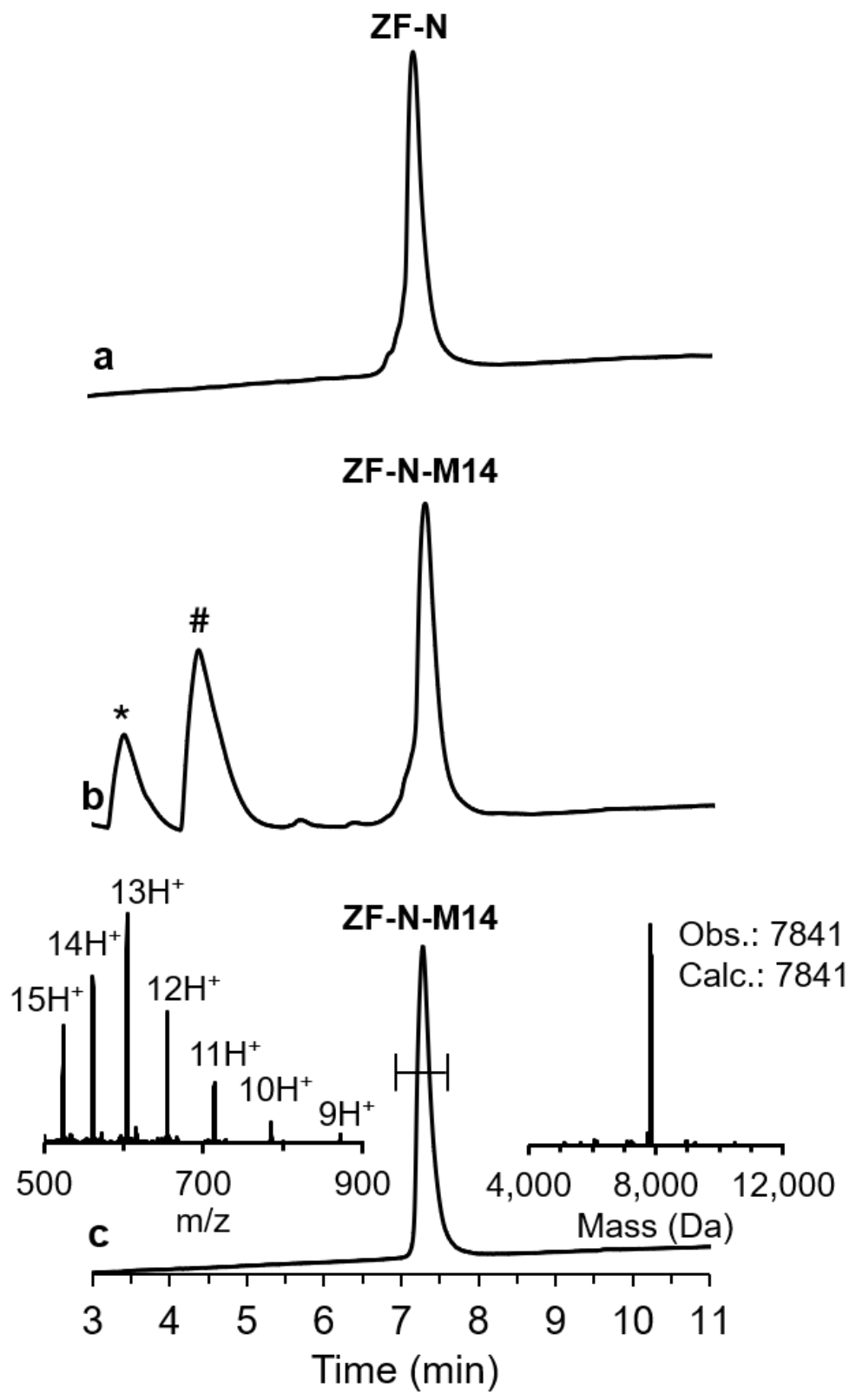

Supplementary Fig. 54 A Schematic representation of site-specific insertion of a PEG tag to **ZF-N**. **B** LC-MS analysis of the PEG modification of the **ZF-N.** (a) **ZF-N** at t = 0. (b) Alkylation at t = 4 h; **ZF-N** with PEG tag (**ZF-N-M14**). (c) Isolated **ZF-N-M14**. # excess amount of 1-bromo-2-(2-(2-methoxyethoxy)ethoxy)ethane and *2-(2-(2-methoxyethoxy)ethoxy)ethan-1-ol.

7.27 Decaging of ZF-N-M14 protein

The decaging of **ZF-N-M14** to form **ZF-M14** was performed as described in section 7.2.

A

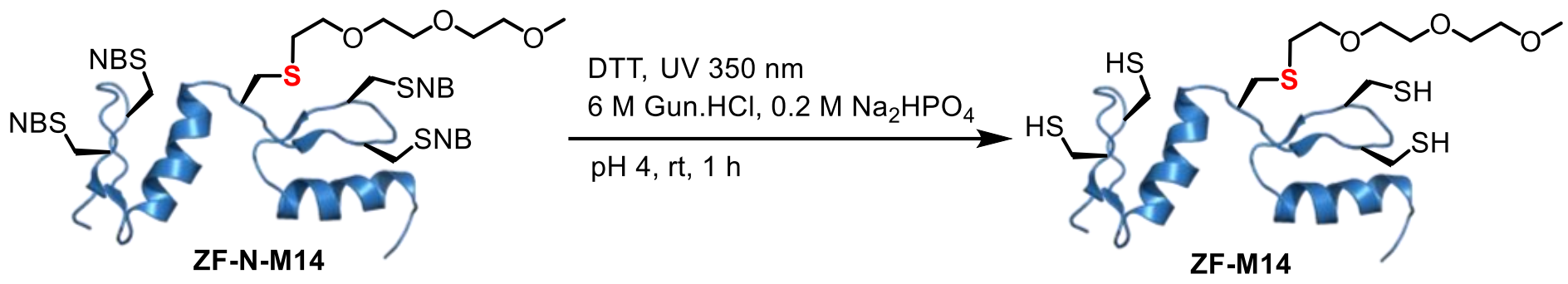

B

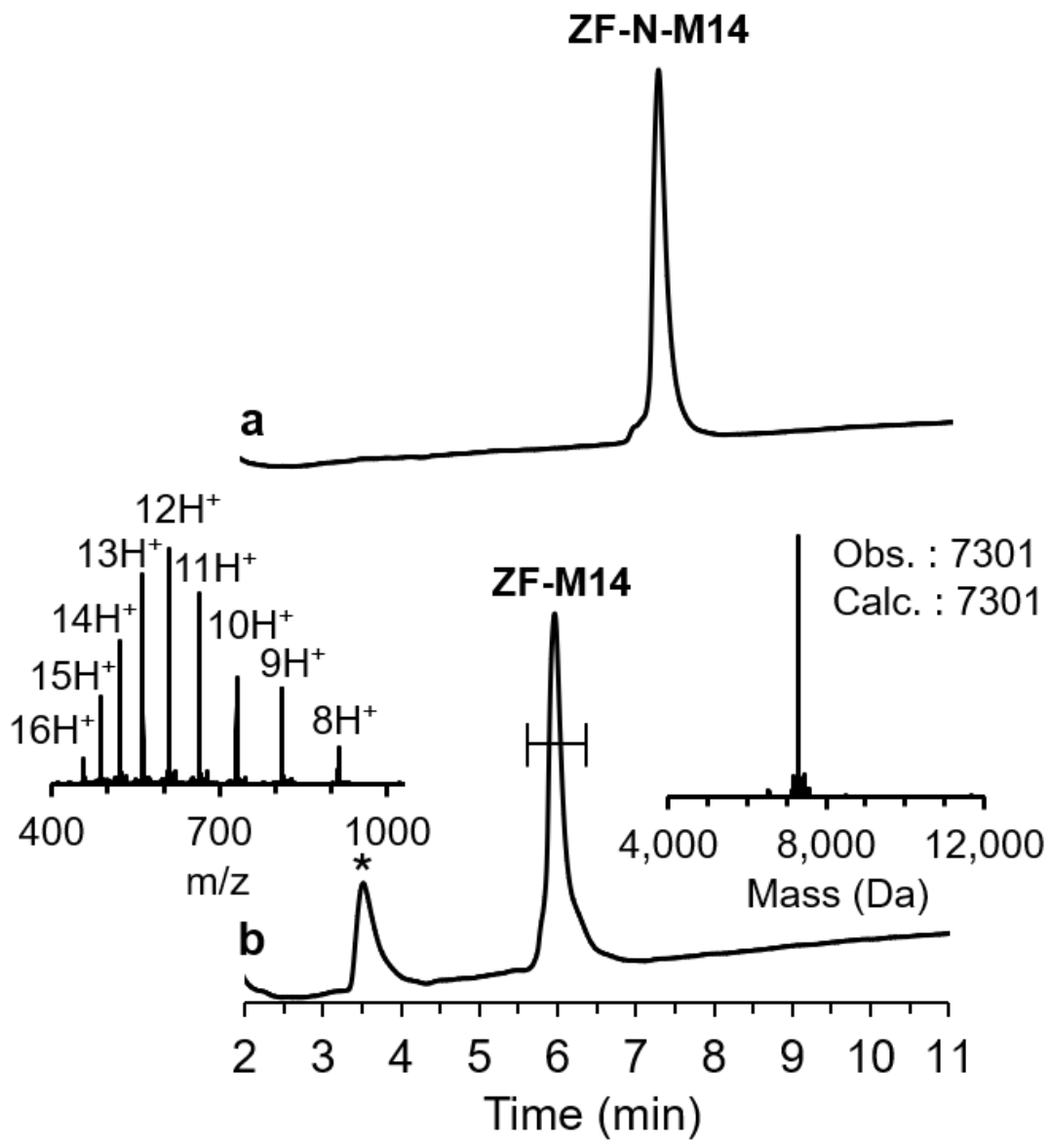

Supplementary Fig. 55 **A** Schematic representation of decaging of **ZF-N-M14**. **B** LC-MS analysis of decaging of **ZF-N-M14**. (a) **ZF-N-M14** at t = 0. (b) Decaging at t = 1 h; **ZF** with PEG tag (**ZF-M14)**. * NB decomposition byproduct.

7.28 Site-specific insertion of fluorescein-5 probe to ZF protein

In a 1.5 mL Eppendorf tube, **ZF-N** (4.0 mg, 1.0 equiv., 0.52 μmol, 0.52 mM) was dissolved in 6 M Gun.HCl, 0.2 M Na_2HPO_4 buffer (1.0 mL). To this solution, fluorescein-5-maleimide (FM, 0.33 mg, 1.5 equiv., 0.78 μmol, 15 mM; based on **ZF-N**) dissolved in DMF (52 μL) was added. The pH was adjusted to 7.3 using 5 *N* NaOH. The mixture was then incubated for 30 min at 37 °C. The reaction was monitored by LC-MS (Method A described in Section 1.2). After completion of the reaction, the reaction mixture was quenched with DTT (4.8 mg, 60.0 equiv., 31.2 μmol, 1.56 M; based on **ZF-N**) dissolved in 6 M Gun.HCl, 0.2 M Na_2HPO_4 buffer (20 μL) for 30 min at 37 °C. The quenching was monitored by LC-MS (Method A described in Section 1.2). After completion of the reaction, decaging of **ZF-N-M15** was performed as described in section 7.2, affording the final product **ZF-M15** (36% isolated yield) as a yellow powder after purification by RP-HPLC (Method C, Section 1.3).

A

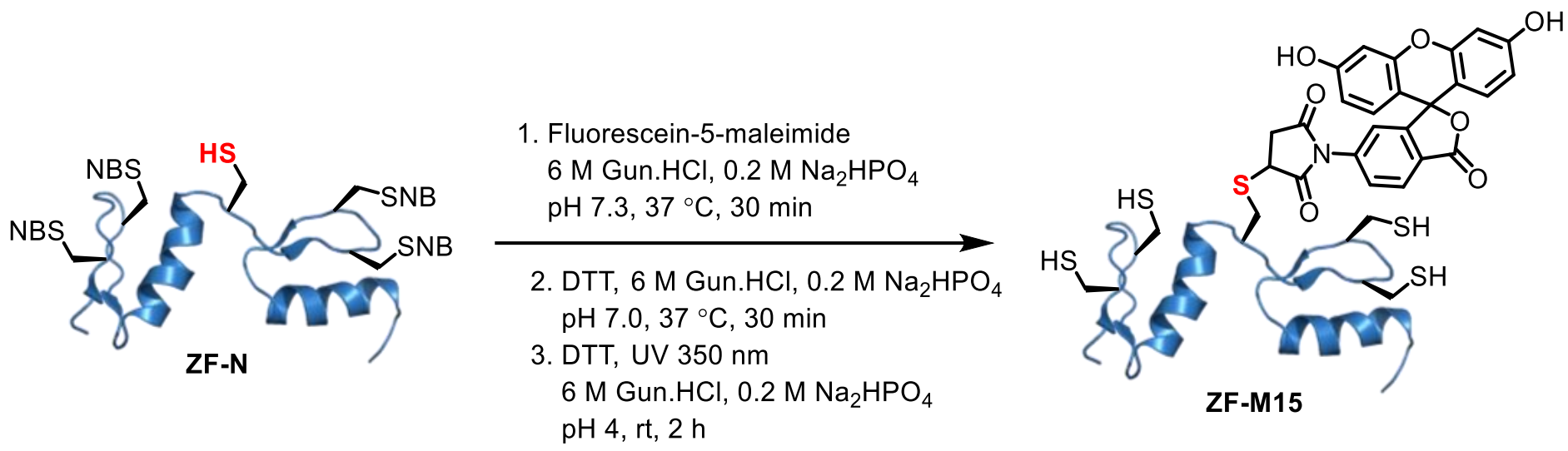

B

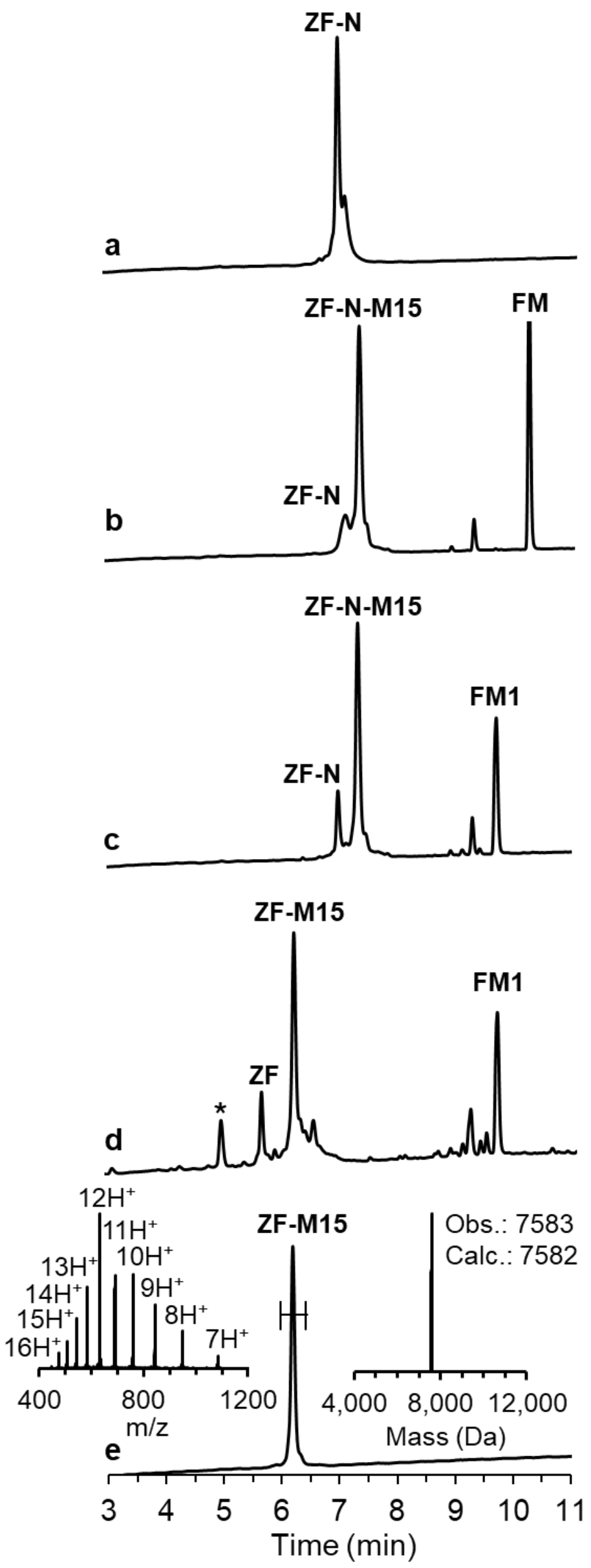

Supplementary Fig. 56 A Schematic representation of site-specific insertion of fluorescein-5 probe to **ZF-N** and decaging. **B** LC-MS analysis of one-pot fluorescein-5 modification and decaging of **ZF-N.** (a) **ZF-N** at t = 0. (b) Michael addition to **ZF-N** at t = 30 min; **ZF-N** with fluorescein-5 probe (**ZF-N-M15**). (c) Quenching at t =1 h. (d) Decaging at t = 3 h; **ZF** with fluorescein-5 probe **(ZF-M15)**. (e) Isolated **ZF-M15**. FM1 is fluorescein-5-maleimide-DTT adduct and * NB decomposition byproduct.

7.29 Site-specific insertion of SID-maleimide to ZF protein

The site-specific modification of **ZF-N** protein using SID-maleimide was performed as described in section 7.28.

A

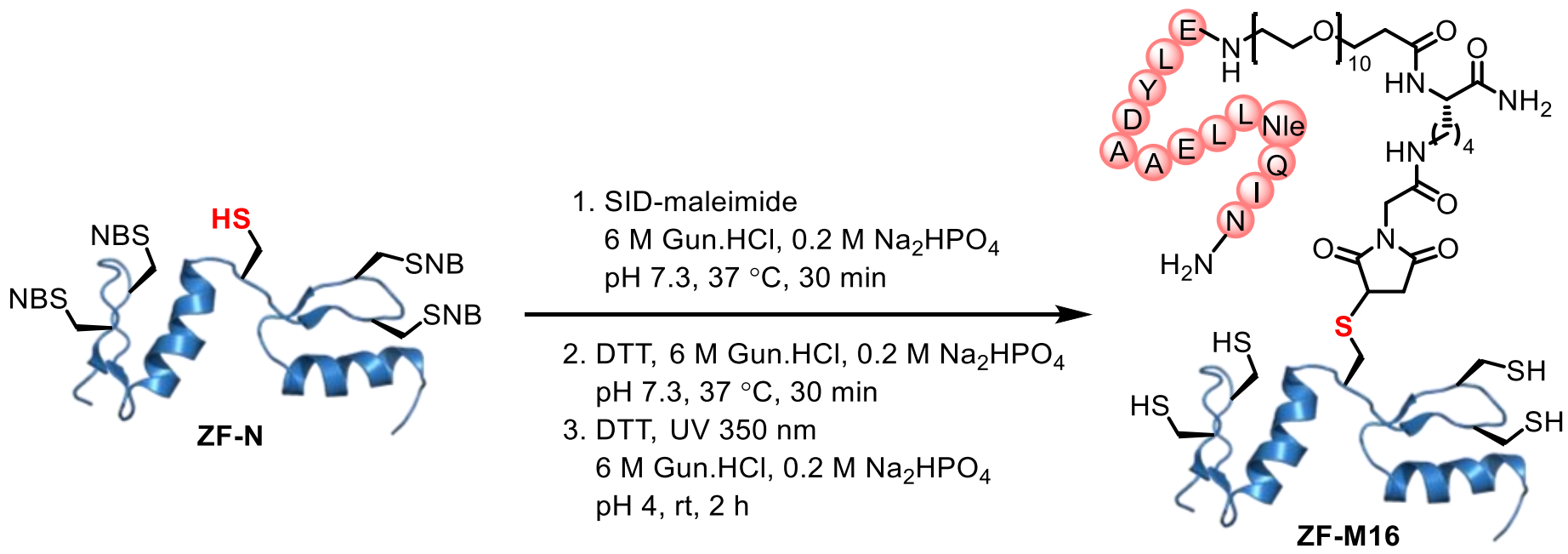

B

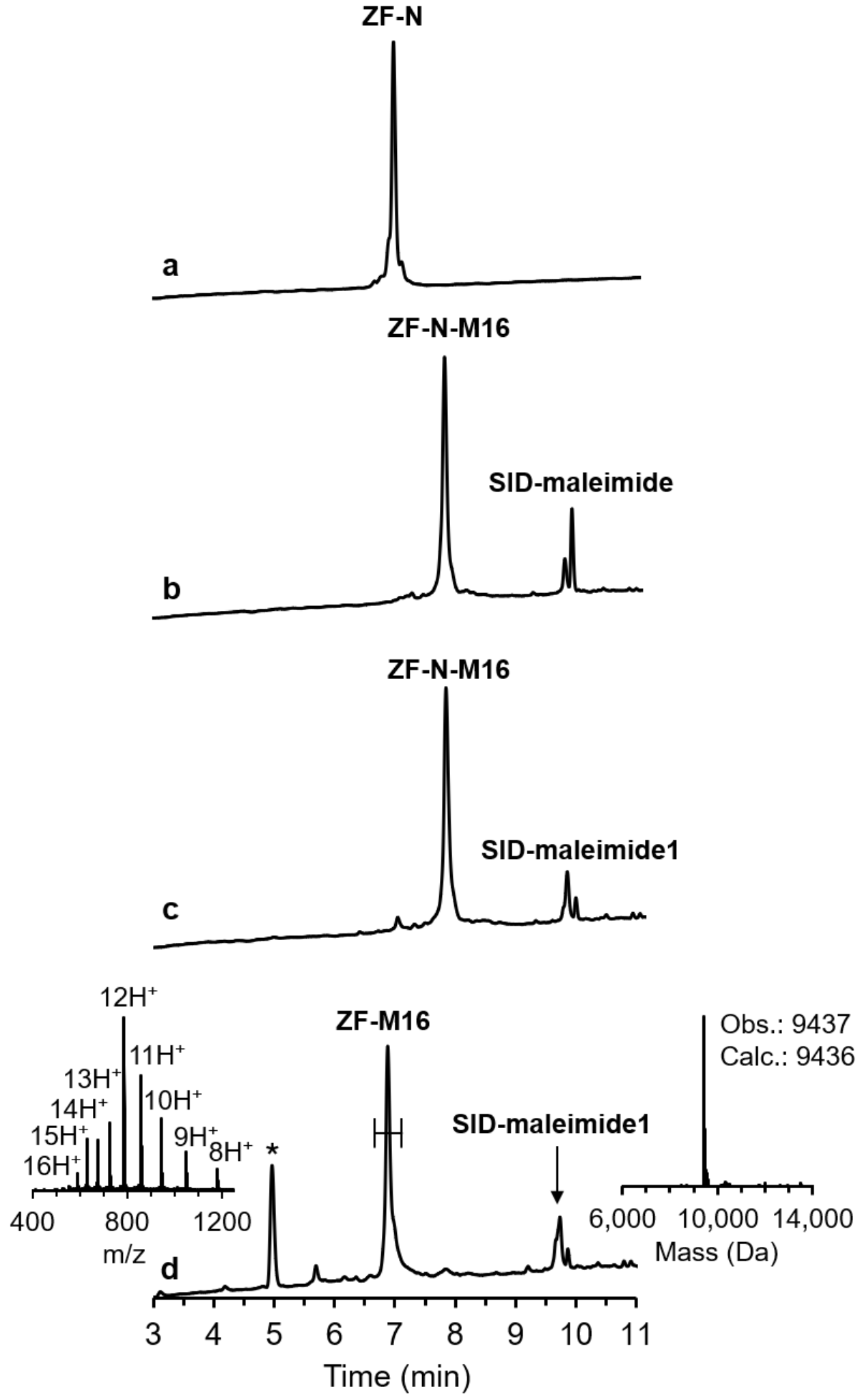

Supplementary Fig. 57 A Schematic representation of site-specific insertion of polypeptide to **ZF-N** and decaging. **B** LC-MS analysis of one-pot polypeptide insertion and decaging of **ZF-N.** (a) **ZF-N** at t = 0. (b) Michael addition to **ZF-N** at t = 30 min; **ZF-N** with peptide tag (**ZF-N-M16**). (c) Quenching at t = 1 h. (d) Decaging at t = 3 h; **ZF** with peptide tag **(ZF-M16**). SID-maleimide1 is SID-maleimide-DTT adduct and * NB decomposition byproduct.

7.30 Site-specific insertion of AHPC-maleimide to ZF protein

The site-specific modification of **ZF-N** protein using AHPC-maleimide was performed as described in section 7.28.

A

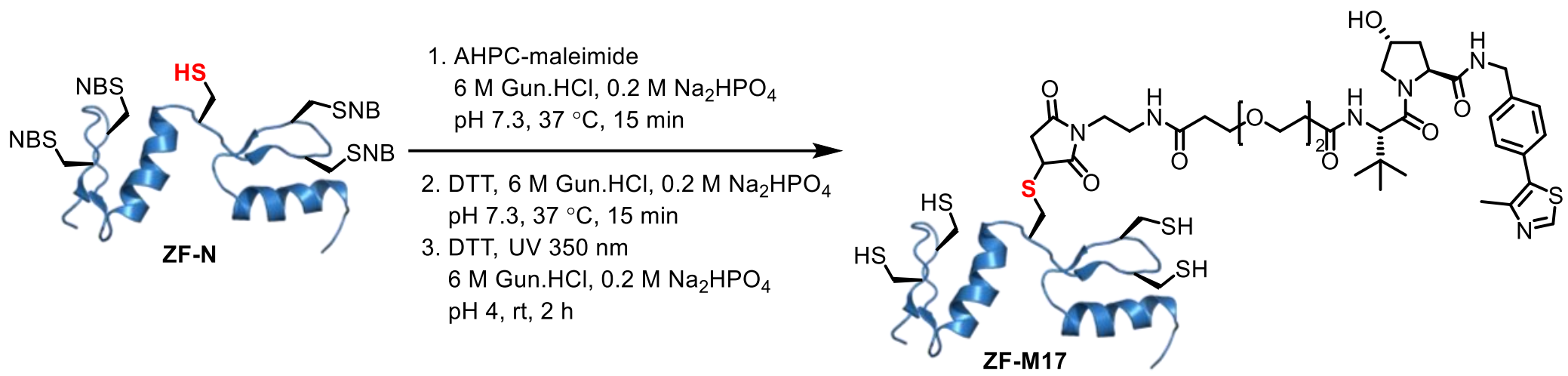

B

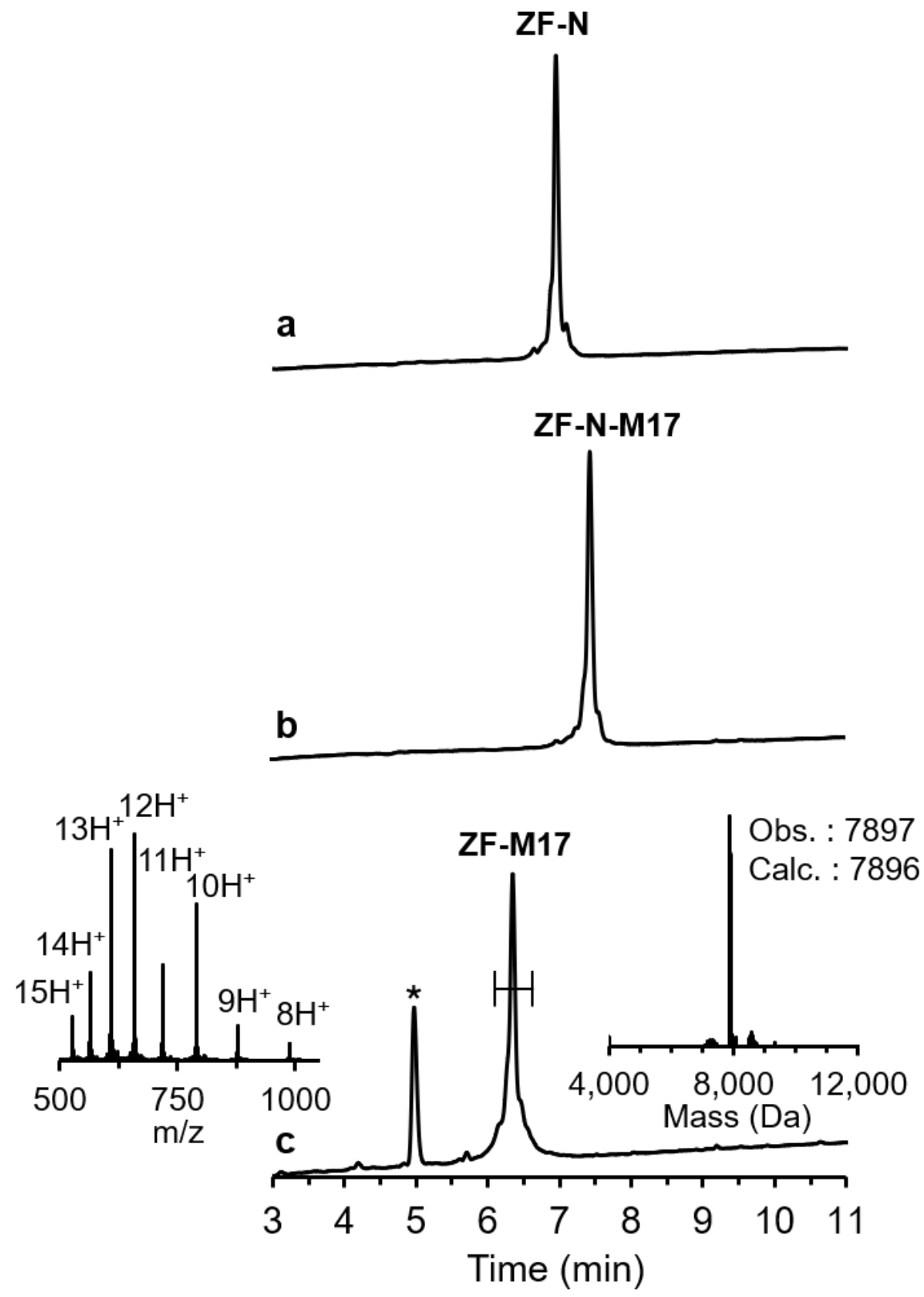

Supplementary Fig. 58 A Schematic representation of site-specific insertion of PROTAC to **ZF-N** and decaging. **B** LC-MS analysis of one-pot PROTAC modification and decaging of **ZF-N.** (a) **ZF-N** at t = 0. (b) Michael addition to **ZF-N** at t = 15 min; **ZF-N** with PROTAC (**ZF-N-M17**). (c) decaging **ZF-N-M17** at t = 2.5 h; **ZF** with PROTAC **(ZF-M17)** and * NB decomposition byproduct.

8. Site-specific modification of eZF-TF protein

8.1 Site-specific insertion of Gln-mimic to eZF-TF protein

The one-pot Gln-mimic modification and decaging of **eZF-TF-N** were carried out following the procedures described in sections 7.20 and 7.2, respectively. Reaction progress was monitored by LC-MS using Method B (Section 1.2), affording the final product, **eZF-TF1** (39% isolated yield), as a white powder after purification by RP-HPLC (Method D, Section 1.3).

A

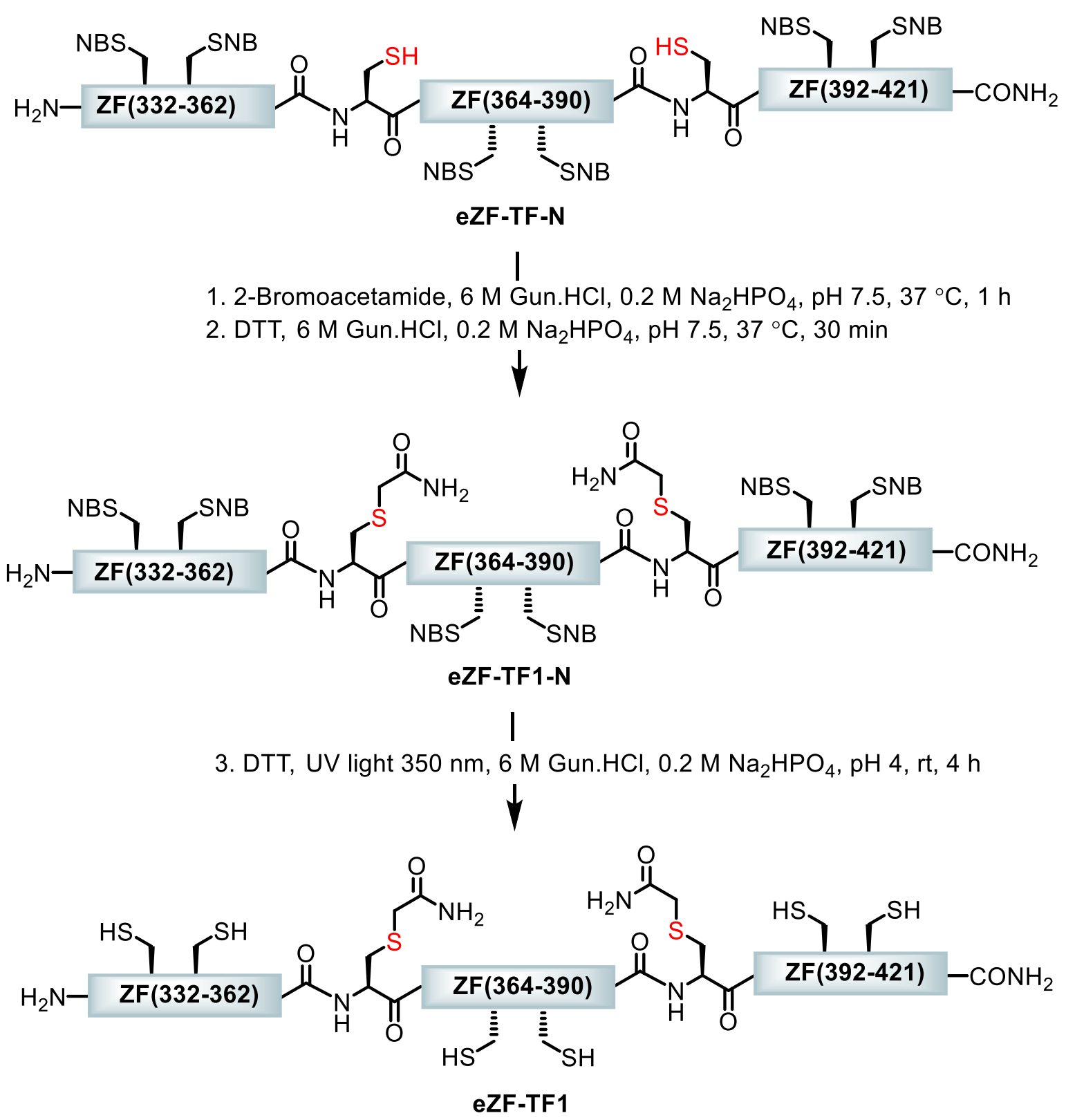

B

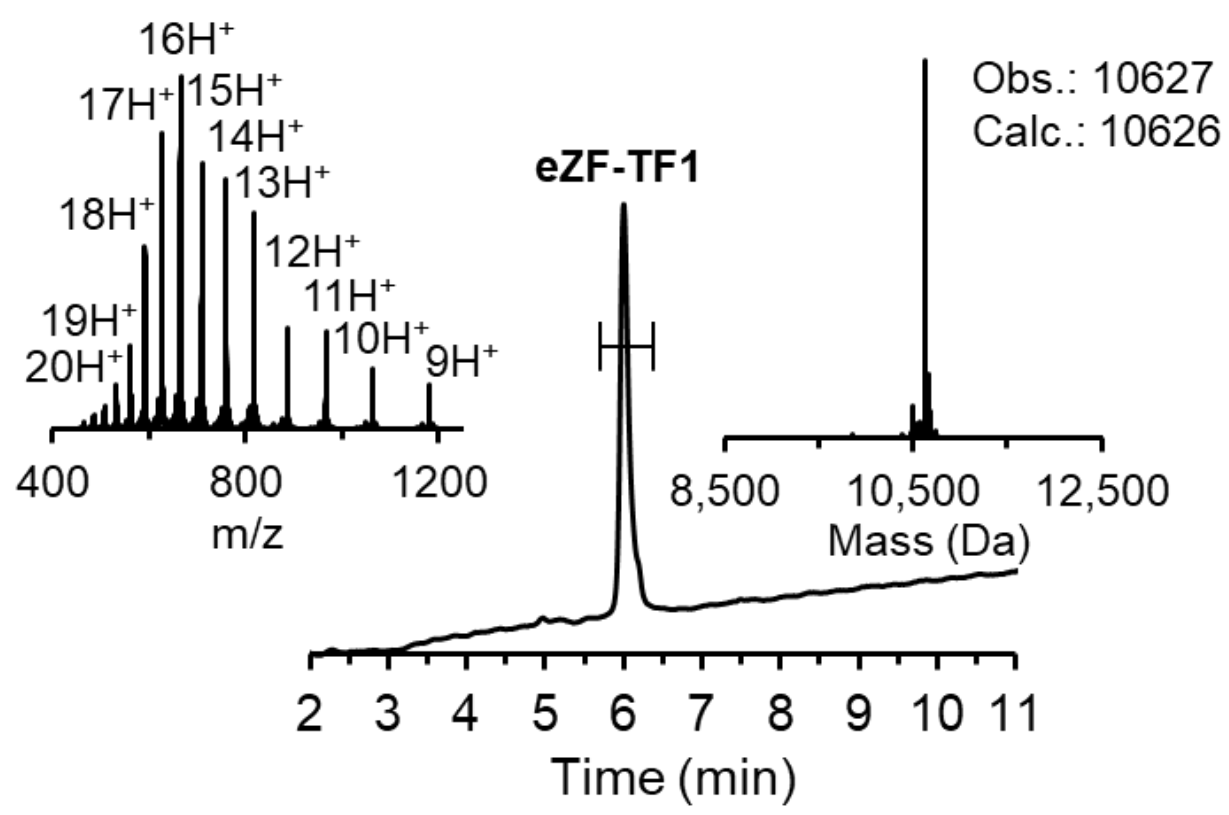

Supplementary Fig. 59 **A** Schematic representation of site-specific insertion of Gln-mimic to **eZF-TF-N** and decaging. **B** LC-MS analysis of isolated **eZF-TF1**.

8.2 Site-specific insertion of Glu-mimic to eZF-TF protein

The one-pot Glu-mimic modification and decagingof **eZF-TF-N** were carried out following the procedures described in sections 7.20 using 2-bromoacetic acid and 7.2, respectively. Reaction progress was monitored by LC-MS using Method B (Section 1.2), affording the final product, **eZF-TF2** (40% isolated yield), as a white powder after purification by RP-HPLC (Method D, Section 1.3).

A

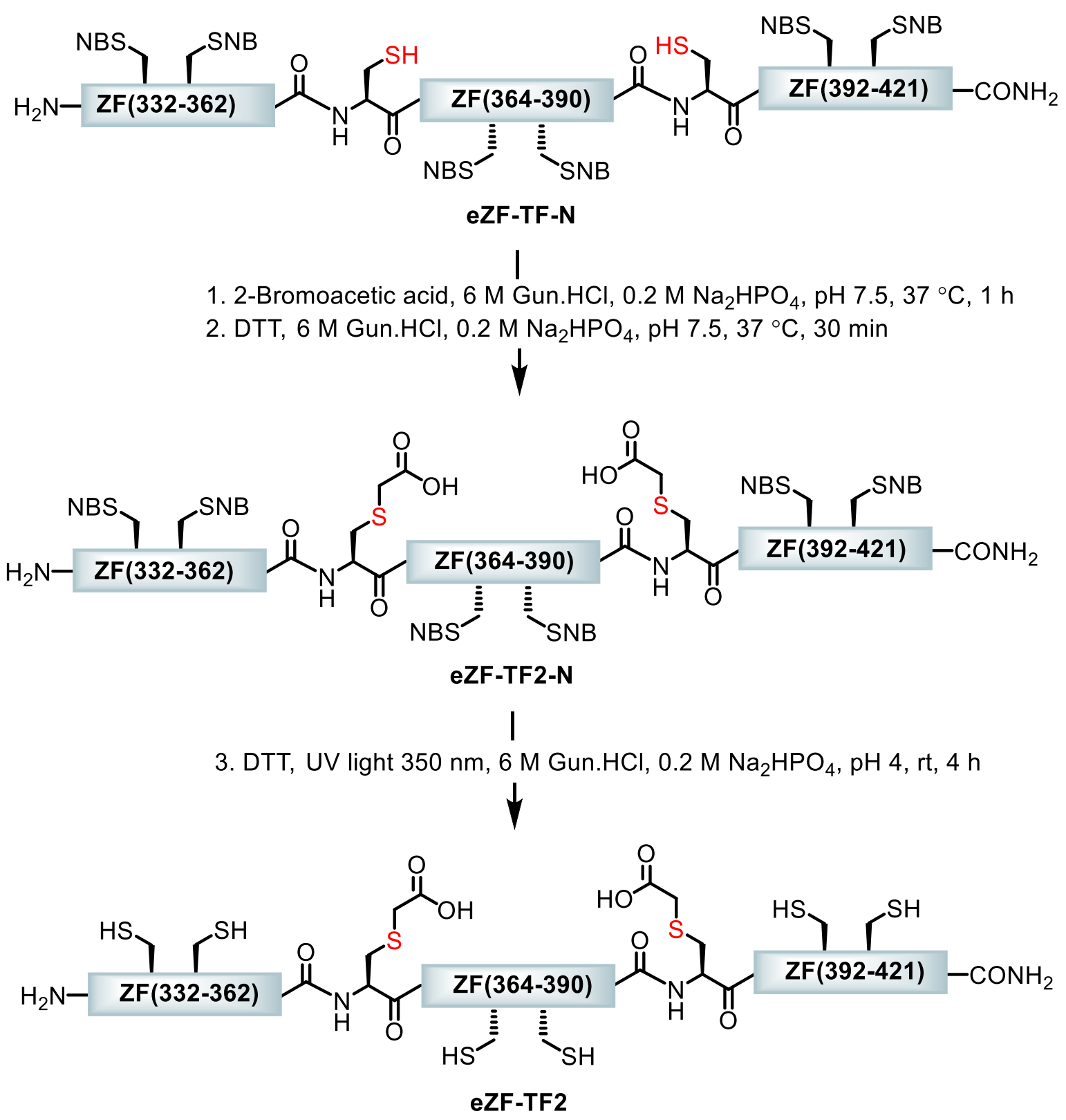

B

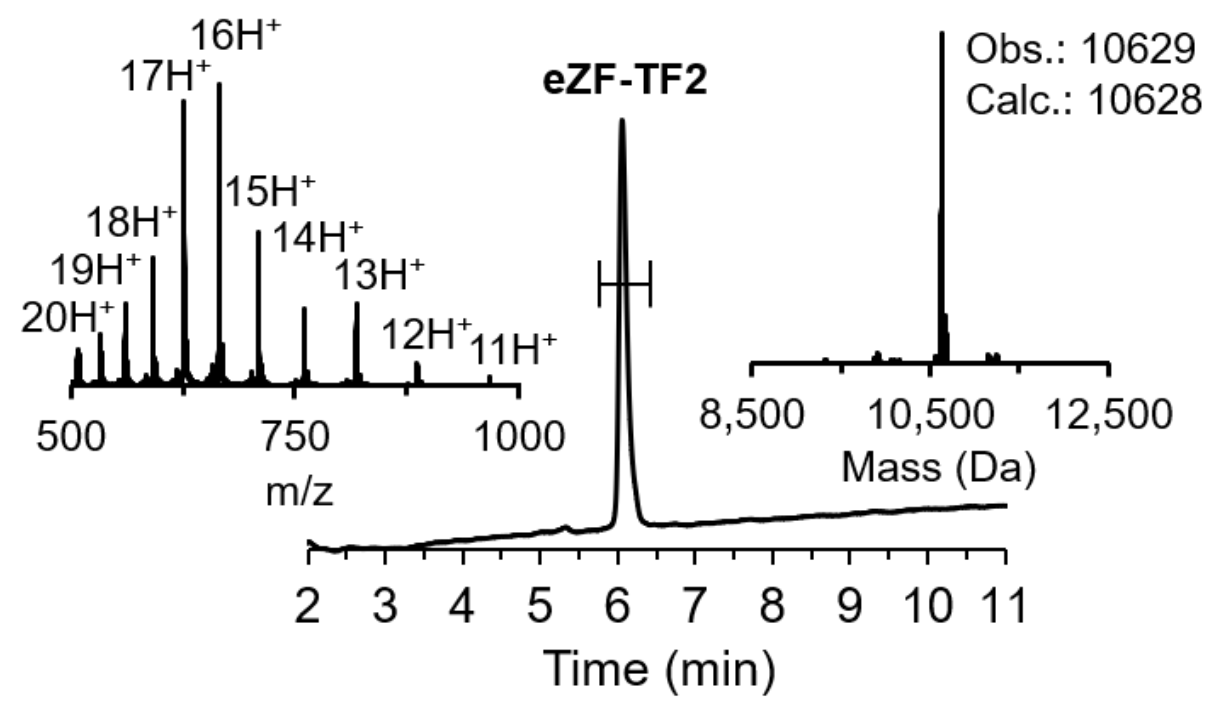

Supplementary Fig. 60 A Schematic representation of site-specific insertion of Glu-mimic to **eZF-TF-N** and decaging. **B** LC-MS analysis of isolated **eZF-TF2**.

8.3 Site-specific insertion of Lys-mimic to eZF-TF-N protein

The Lys-mimic modification of **eZF-TF-N** was carried out following the procedures described in section 7.1 using 2-bromoethylamine hydrobromide. Reaction progress was monitored by LC-MS using Method B (Section 1.2), affording the final product, **eZF-TF3-N** (40% isolated yield), as a white powder after purification by RP-HPLC (Method D, Section 1.3).

A

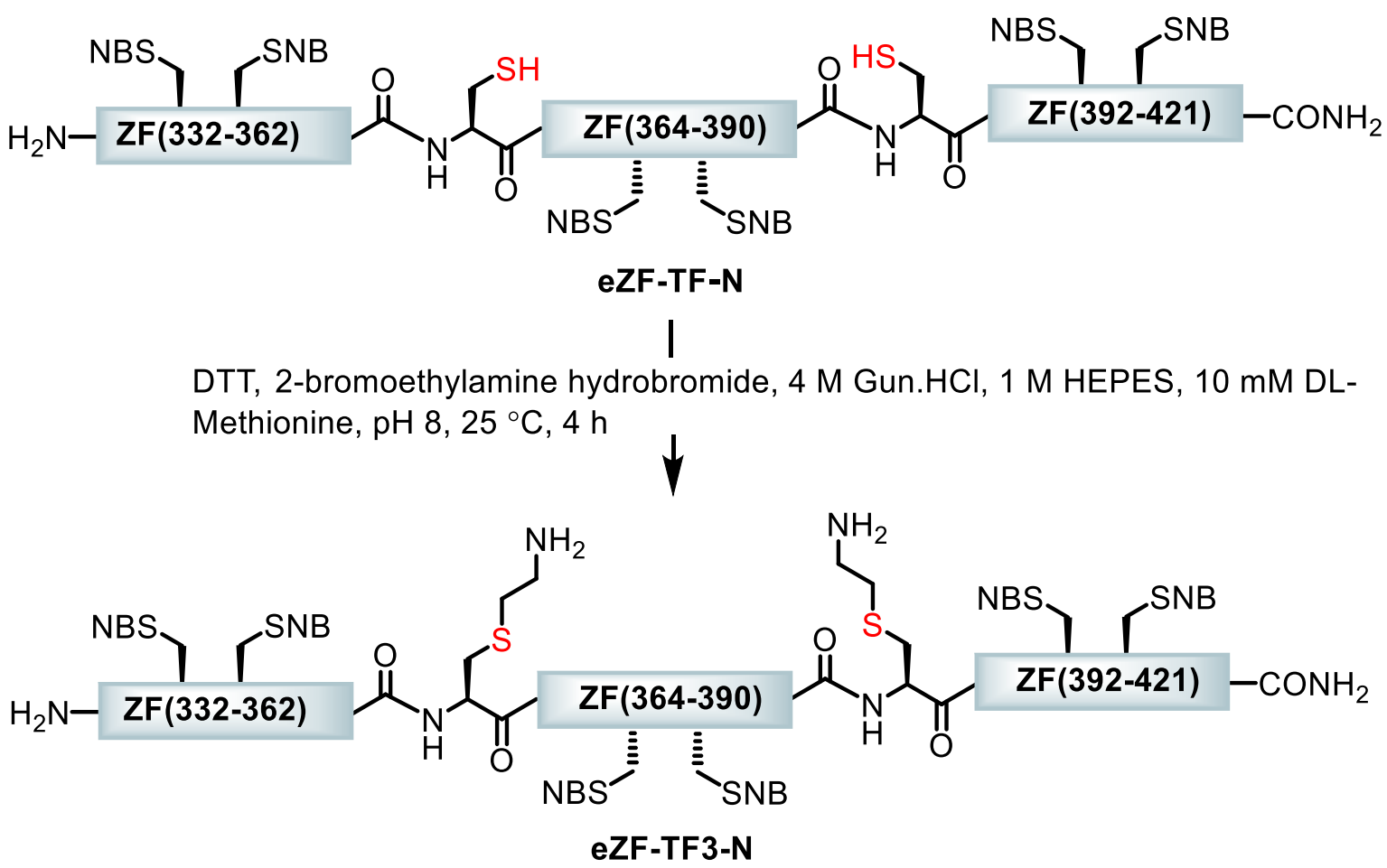

B

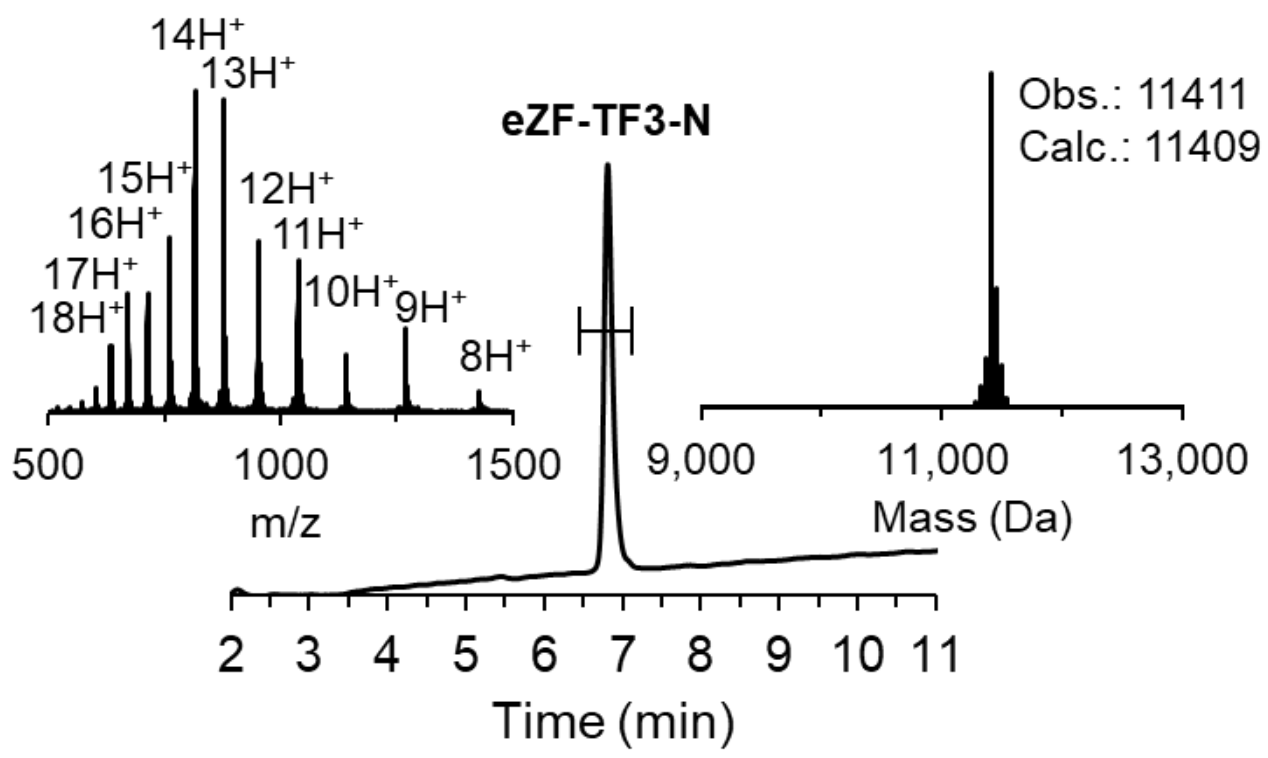

Supplementary Fig. 61 A Schematic representation of site-specific insertion of Lys-mimic to **eZF-TF-N**. **B** LC-MS analysis of isolated **eZF-TF3-N**.

8.4 Decaging of eZF-TF3-N protein

The decaging of **eZF-TF3-N** to form **eZF-TF3** was performed as described in section 7.2, affording the final product **eZF-TF3** (62% isolated yield) as a white powder after purification by RP-HPLC (Method D, Section 1.3).

A

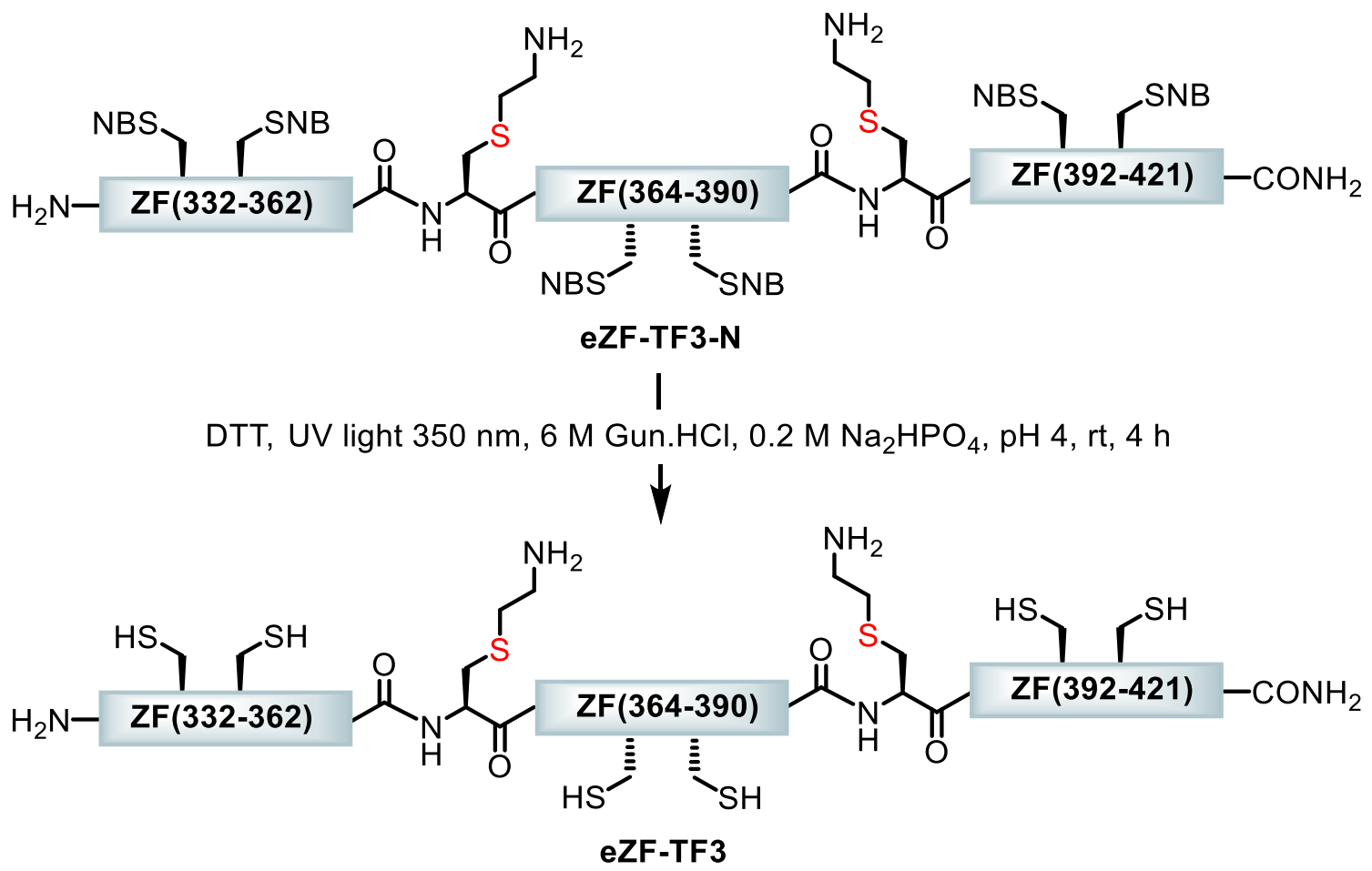

B

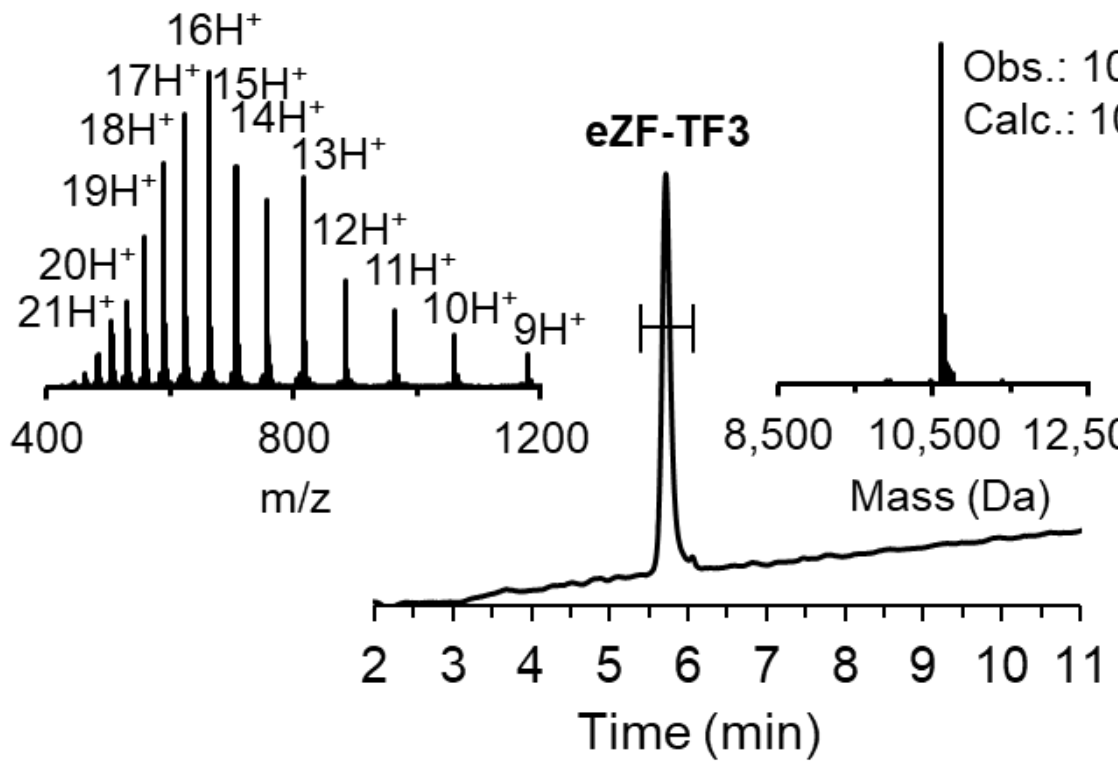

Supplementary Fig. 62 A Schematic representation of decaging of **eZF-TF3-N**. **B** LC-MS analysis of isolated **eZF-TF3**.

8.5 Site-specific insertion of monomethyl-Lys to eZF-TF protein

The one-pot monomethyl-Lys modification and decaging of **eZF-TF-N** were carried out following the procedures described in sections 7.1 using 2-chloro-N-methylethanamine hydrochloride and 7.2, respectively. Additionally, an excess amount of alkyl halide was quenched before the decaging step using 2-mercaptoethanol (2 eq. compared to alkyl halide). Reaction progress was monitored by LC-MS using Method B (Section 1.2), affording the final product, **eZF-TF4** (14% isolated yield), as a white powder after purification by RP-HPLC (Method D, Section 1.3).

A

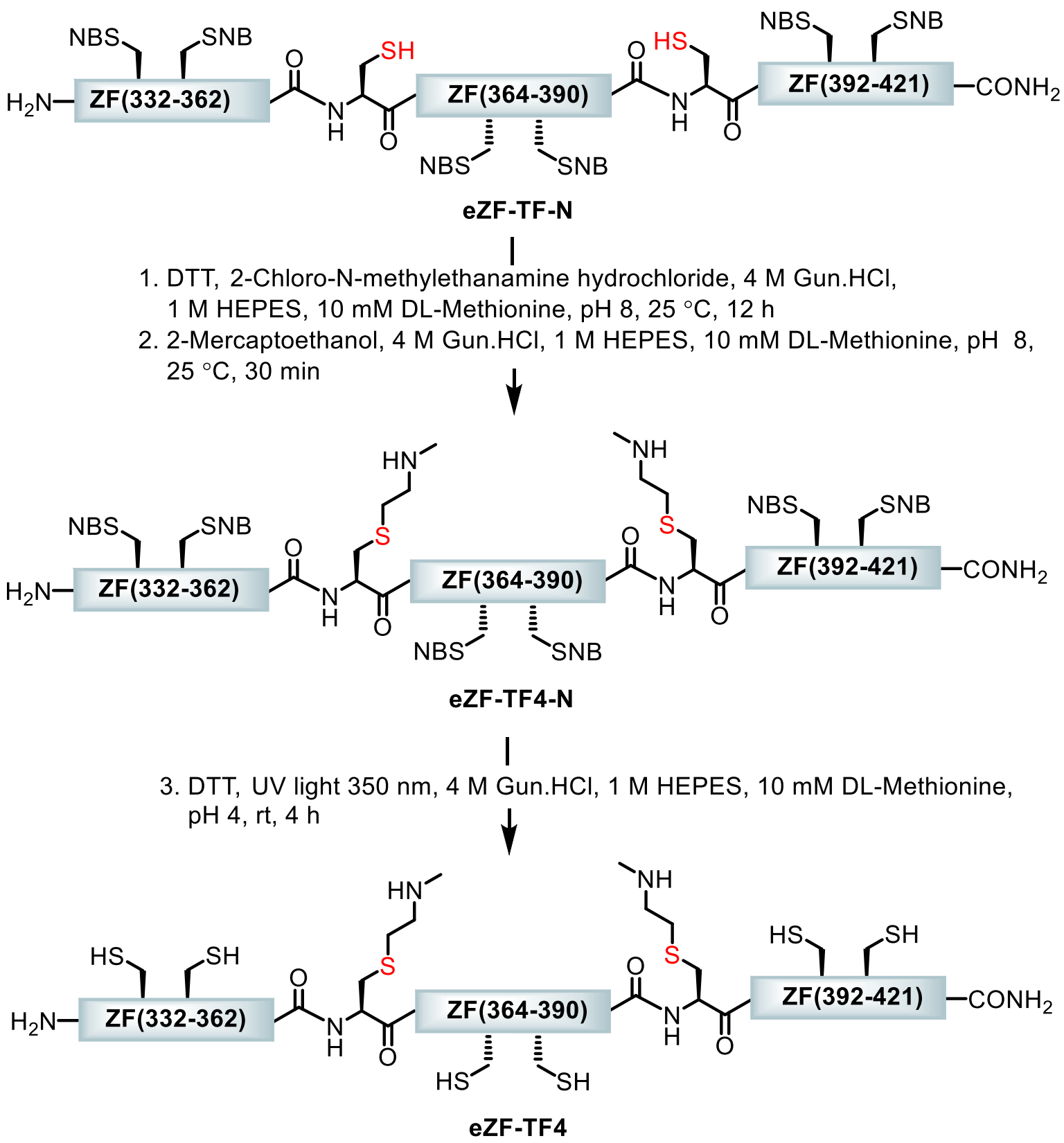

B

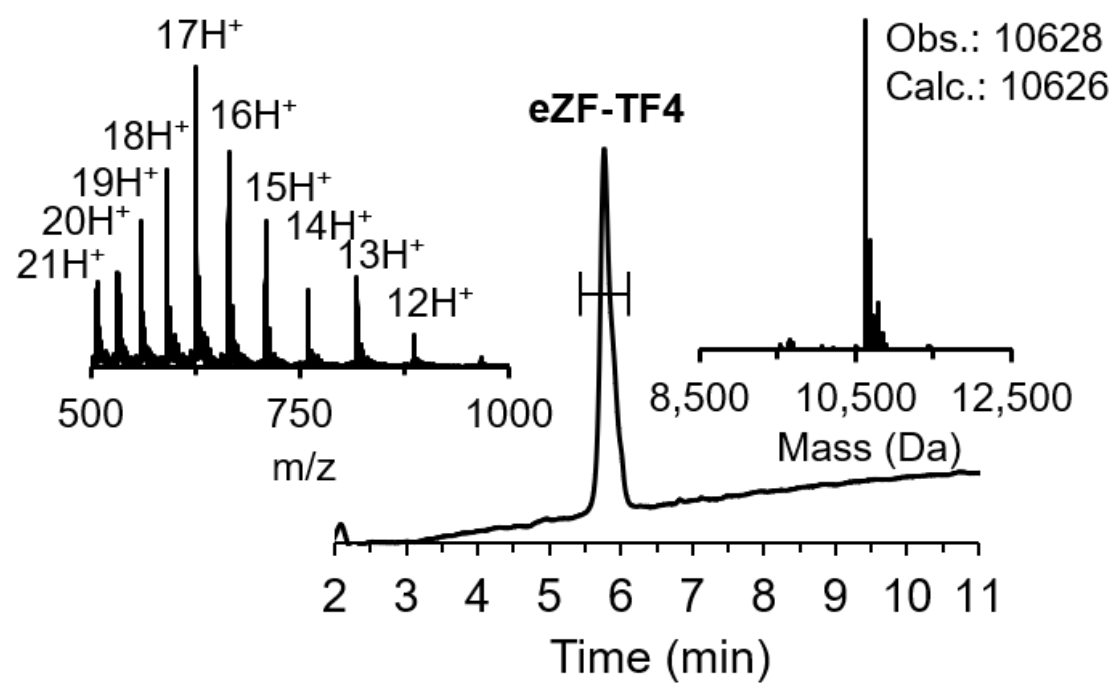

Supplementary Fig. 63 A Schematic representation of site-specific insertion of monomethyl-Lys to **eZF-TF-N** and decaging. **B** LC-MS analysis of isolated **eZF-TF4**.

8.6 Site-specific insertion of phospho-Tyr to eZF-TF-N protein

The Tyr-phosphorylation modification of **eZF-TF-N** was carried out following the procedures described in section 7.7. Reaction progress was monitored by LC-MS using Method B (Section 1.2), affording the final product, **eZF-TF5-N** (23% isolated yield), as a white powder after purification by RP-HPLC (Method D, Section 1.3).

A

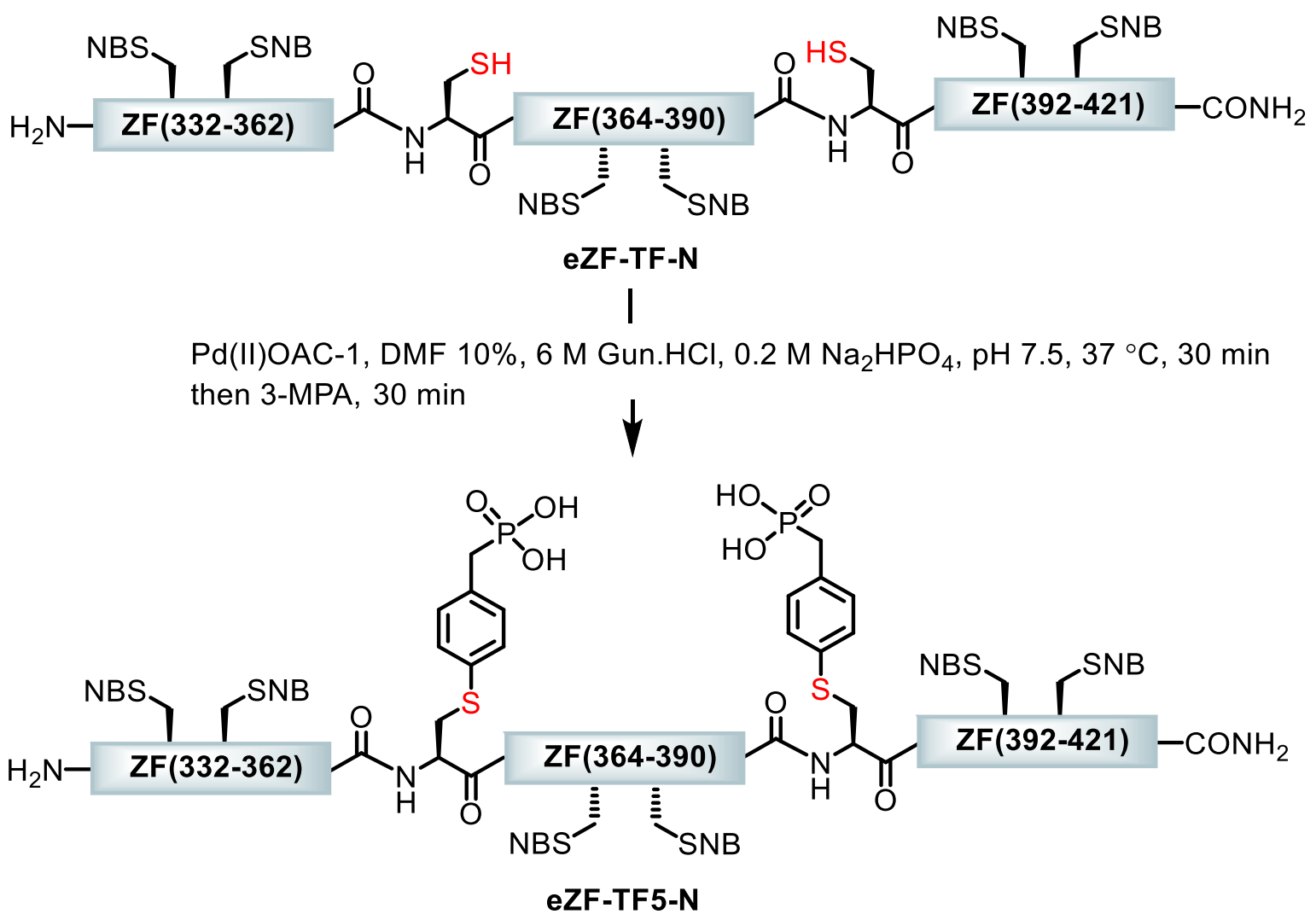

B

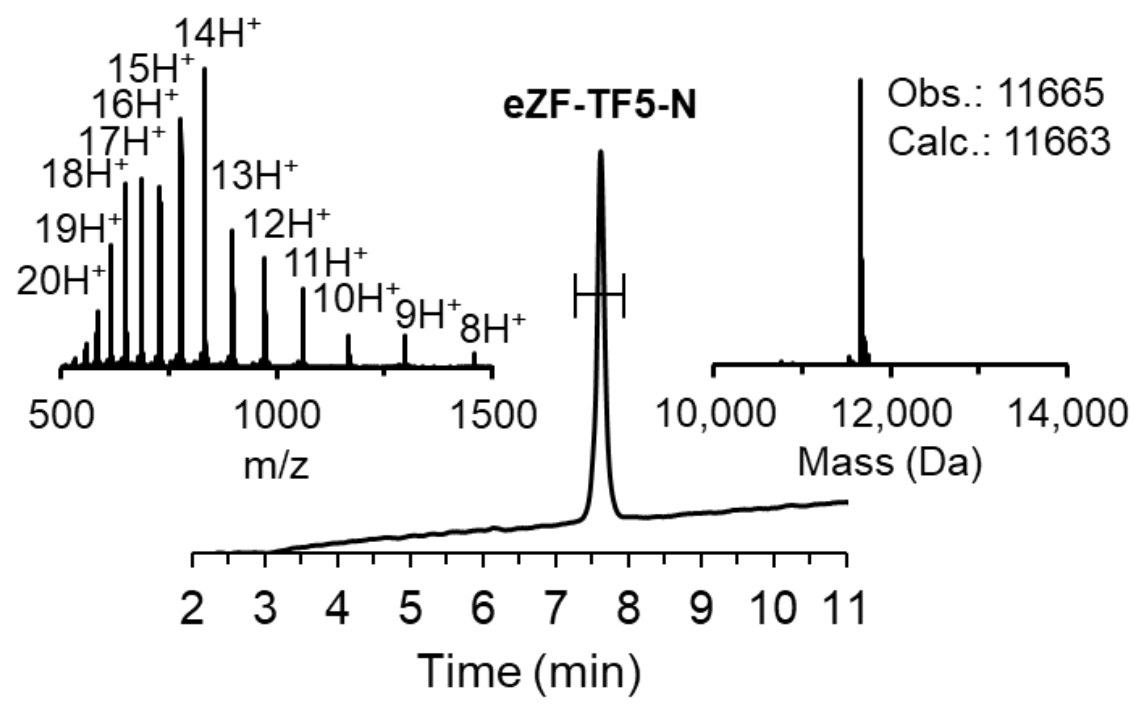

Supplementary Fig. 64 A Schematic representation of site-specific insertion of phospho-Tyr to **eZF-TF-N**. **B** LC-MS analysis of isolated **eZF-TF5-N**.

8.7 Decaging of eZF-TF5-N protein

The decaging of **eZF-TF5-N** to form **eZF-TF5** was performed as described in section 7.2, affording the final product **eZF-TF5** (54% isolated yield) as a white powder after purification by RP-HPLC (Method D, Section 1.3).

A

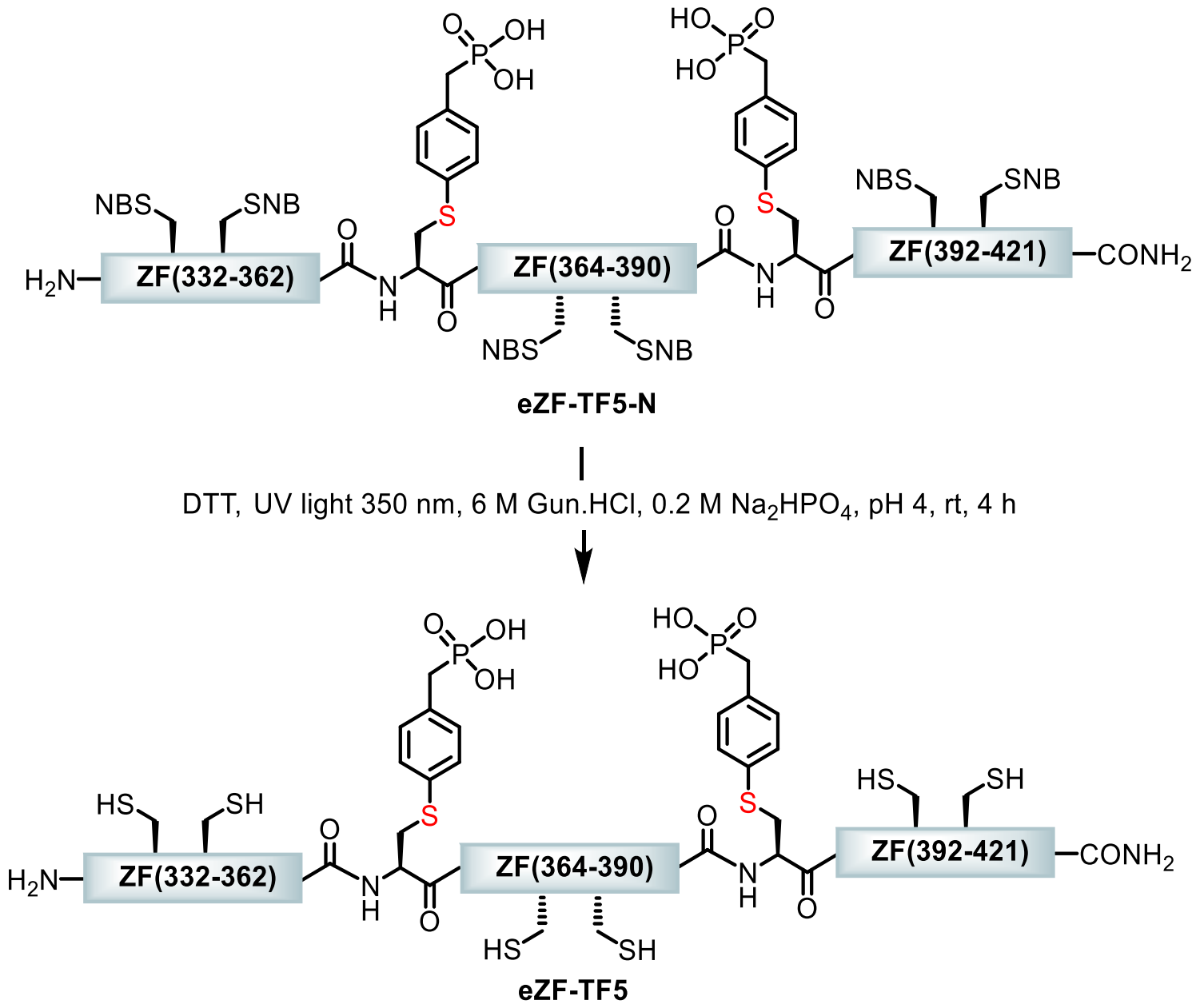

B

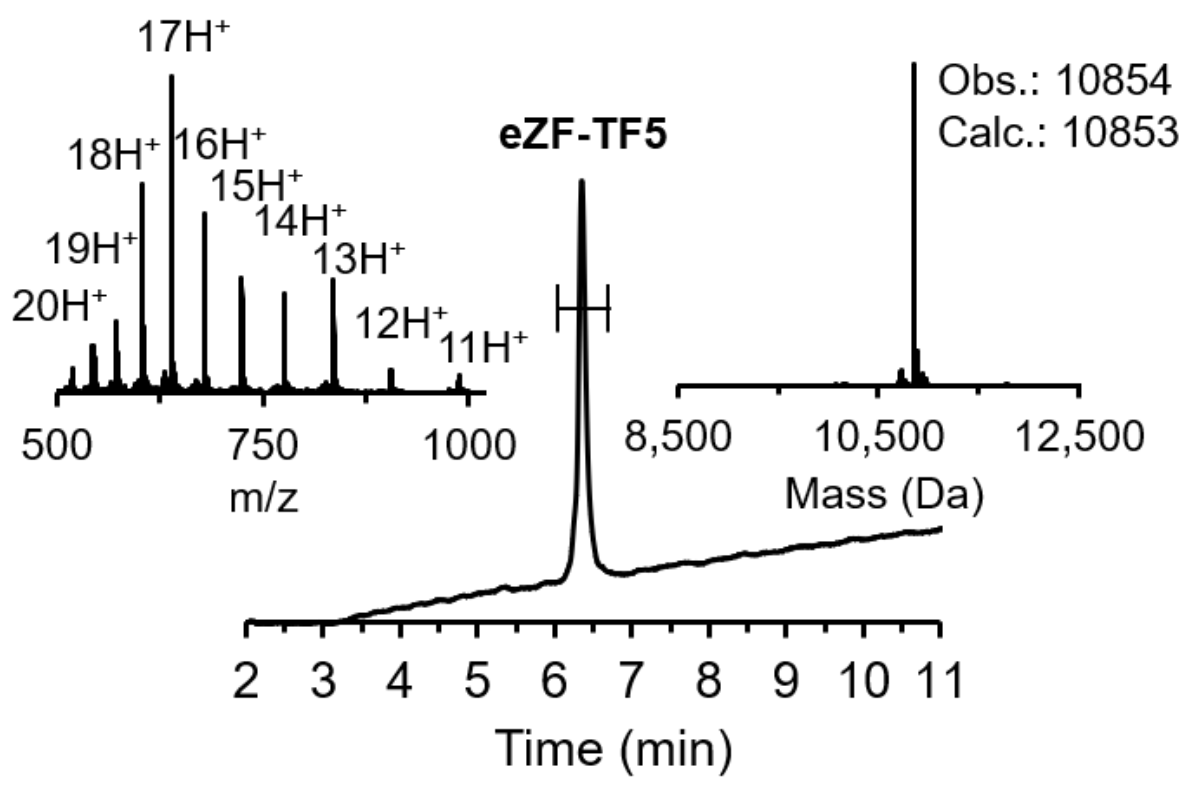

Supplementary Fig. 65 **A** Schematic representation of decaging of **eZF-TF5-N**. **B** LC-MS analysis of isolated **eZF-TF5**.

8.8 Site-specific insertion of Gln-, Glu-, and dimethyl-Lys mimics to ZF3.4-N protein

The synthesis of **ZF3.4-N** protein was carried out using NCL, following a procedure similar to that described in section 5.3. The **ZF3.4-N** protein was directly split into 3 equal portions without prior HPLC purification. The portions were treated with 2-bromoacetamide, 2-bromoacetic acid, and 2-bromo-N,N-dimethylethylamine hydrobromide to get **ZF3.4-N1, ZF3.4-N2,** and **ZF3.4-N3**, respectively. The Gln- mimic modification was performed as described in section 7.20 using 2-bromoacetamide and the Glu-mimic modification was performed as described in section 7.20 using 2-bromoacetic acid, affording the final products, **ZF3.4-N1** (45% isolated yield) and **ZF3.4-N2** (50% isolated yield), as white powder, respectively. Whereas dimethyl-Lys-mimic modification was performed as described in section 7.3 using bromo-N,N-dimethylethylamine hydrobromide, affording the final product, **ZF3.4-N3** (39% isolated yield), as a white powder.

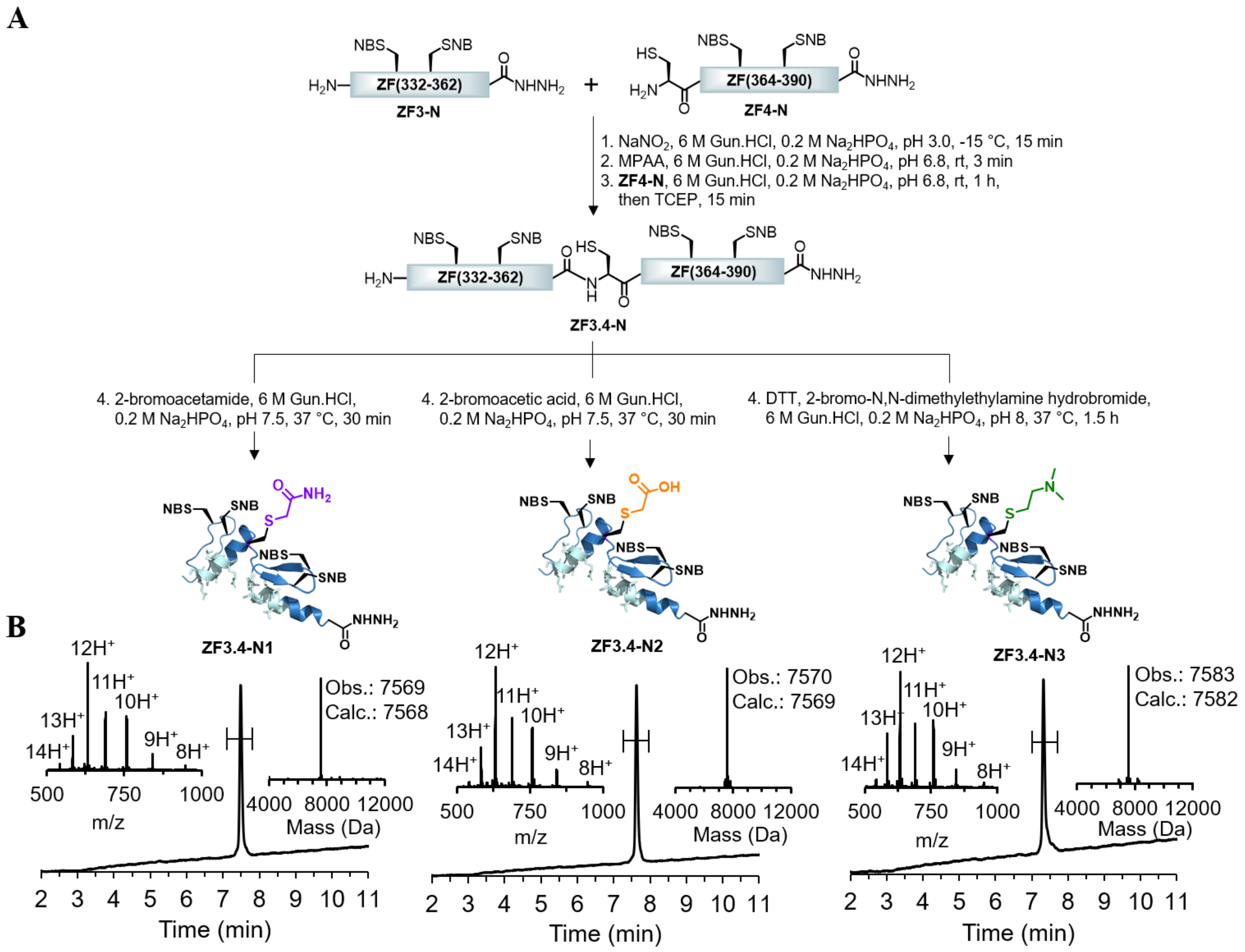

Supplementary Fig. 66 A Schematic representation of ligation and site-selective Cys modification. **B** LC-MS analysis of isolated **ZF3.4-N1**, **ZF3.4-N2** and **ZF3.4-N3**.

8.9 Site-specific insertion of Glu-mimic and fluorescein-5 probe to eZF-TF-N1 protein

The synthesis of **eZF-TF-N1** protein was carried out using NCL, following a procedure similar to that described in section 5.3. The **eZF-TF-N1** protein was directly split into 2 equal portions without prior HPLC purification. The portions were treated with 2-bromoacetic acid and fluorescein-5-maleimide, followed by a decaging step to obtain **eZF-TF6** and **eZF-TF7**, respectively. The Glu-mimic modification and decaging were performed as described in sections 7.20 using 2-bromoacetic acid and 7.2, respectively, affording the final product, **eZF-TF6** (30% isolated yield) as a white powder. The fluorescein-5 probe insertion and decaging were performed as described in section 7.28 to obtain **eZF-TF7** (35% isolated yield), as a white powder.

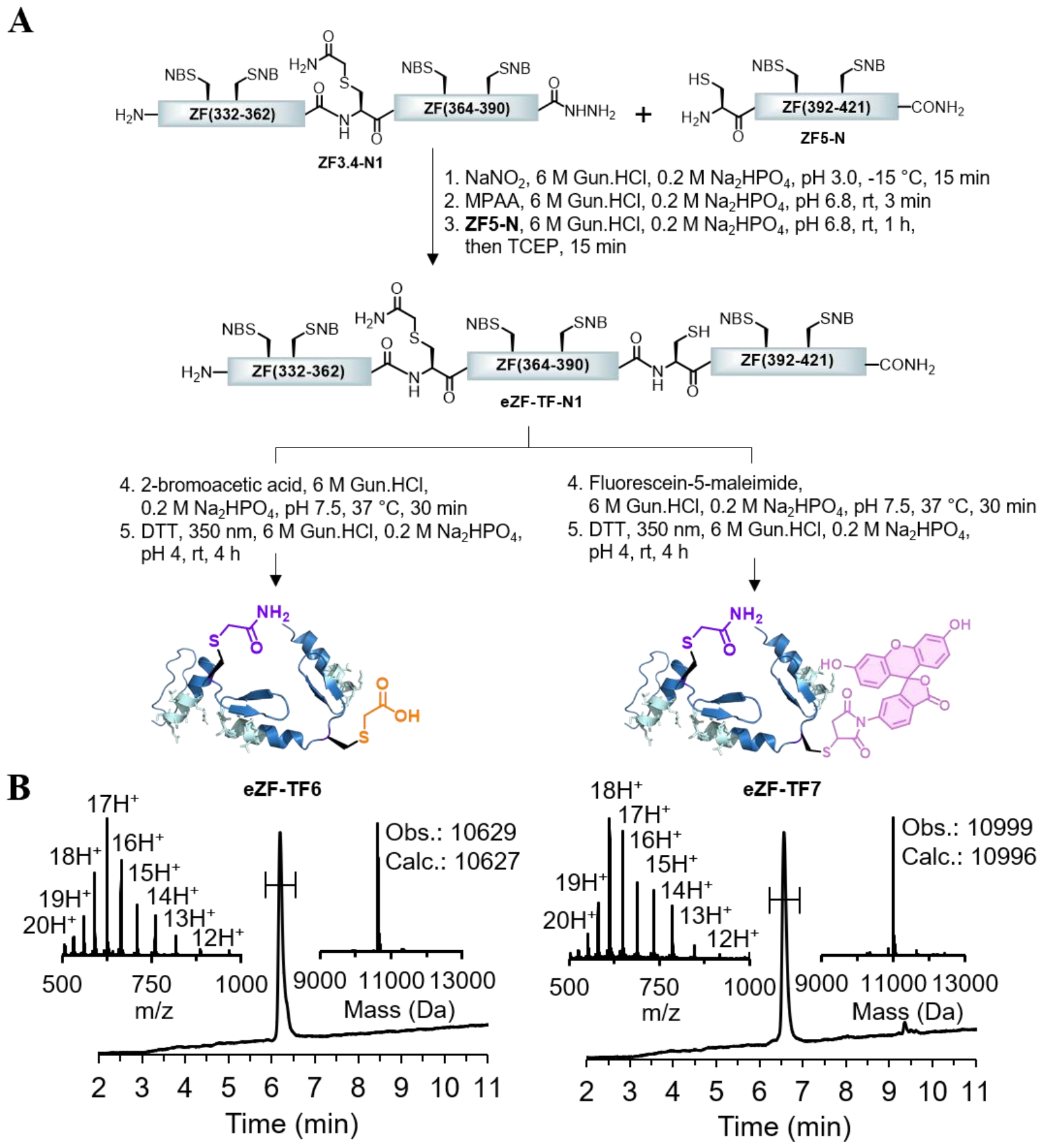

Supplementary Fig. 67 **A** Schematic representation of ligation and site-selective Cys modification. **B** LC-MS analysis of isolated **eZF-TF6** and **eZF-TF7**.

8.10 Site-specific insertion of dimethyl-Lys and biotin tag to eZF-TF-N2 protein

The synthesis of **eZF-TF-N2** protein was carried out using NCL, following a procedure similar to that described in section 5.3. The **eZF-TF-N2** protein was directly split into 2 equal portions without prior HPLC purification. The portions were treated with 2-bromo-N,N-dimethylethylamine hydrobromide and biotin polyethyleneoxide iodoacetamide, followed by a decaging step to obtain **eZF-TF8** and **eZF-TF9**, respectively. The dimethyl-Lys mimic modification and decaging were performed as described in sections 7.3 and 7.2, respectively, affording the final product, **eZF-TF8** (27% isolated yield) as a white powder. The biotin tag insertion and decaging were performed as described in sections 7.20 using biotin polyethyleneoxide iodoacetamide and 7.2, respectively, to obtain **eZF-TF9** (45% isolated yield), as a white powder.

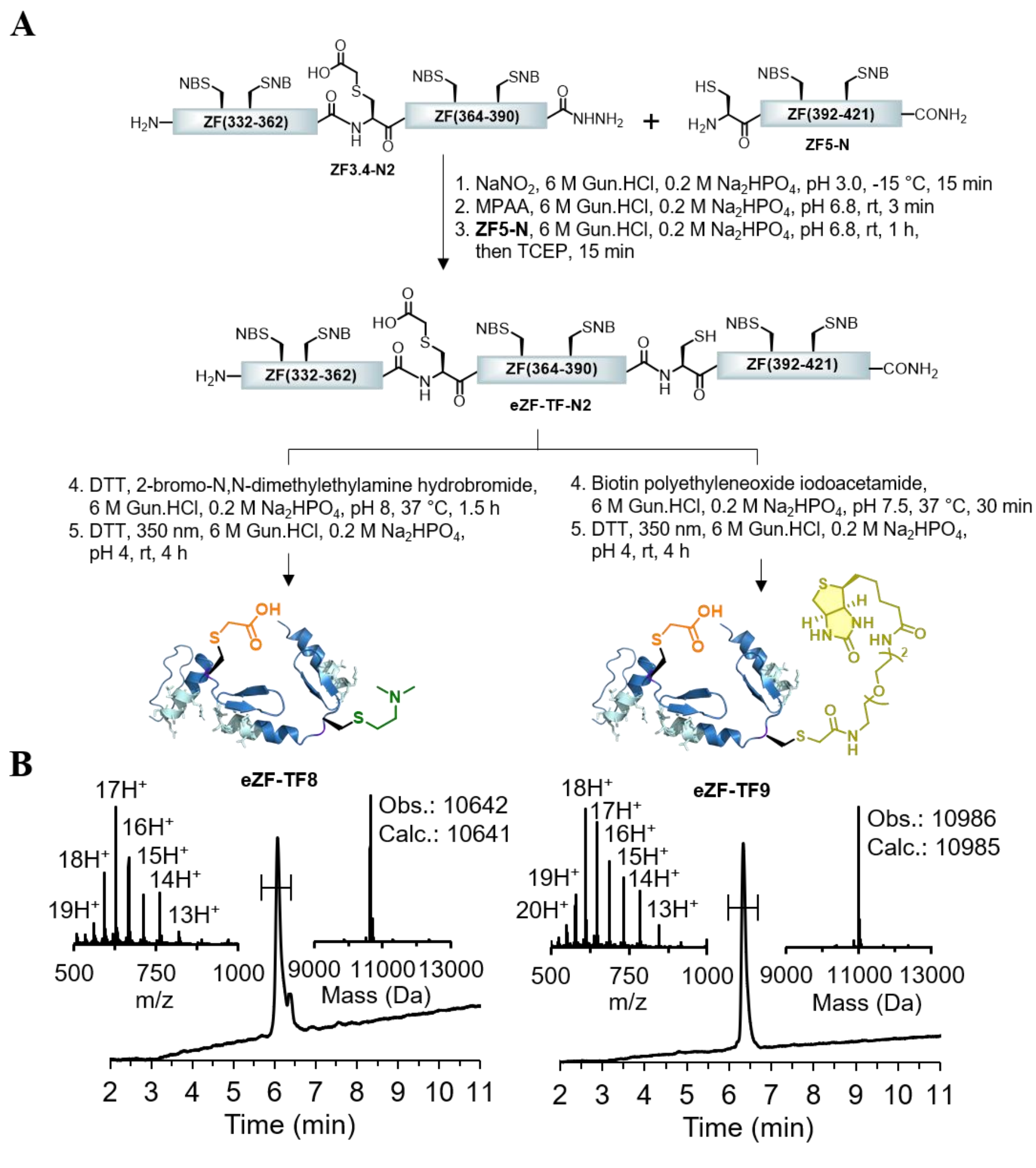

Supplementary Fig. 68 A Schematic representation of ligation and site-selective Cys modification. **B** LC-MS analysis of isolated **eZF-TF8** and **eZF-TF9**.

8.11 Site-specific insertion of trimethyl-Lys and phospho-Tyr to eZF-TF-N3 protein

The synthesis of **eZF-TF-N3** protein was carried out using NCL, following a procedure similar to that described in section 5.3. The **eZF-TF-N3** protein was directly split into 2 equal portions without prior HPLC purification. The portions were treated with (2-bromoethyl)trimethylammonium bromide and Pd(II)OAC-2, followed by a decaging step to obtain **eZF-TF10** and **eZF-TF11**, respectively. The trimethyl-Lys mimic modification and decaging were performed as described in sections 7.1 using (2-bromoethyl)trimethylammonium bromide and 7.2, respectively, affording the final product, **eZF-TF10** (17% isolated yield) as a white powder. The phospho-Tyr mimic insertion and decaging were performed as described in sections 7.7 using Pd(II)OAC-2 and 7.2, respectively, to obtain the final product **eZF-TF11** (26% isolated yield), as a white powder.

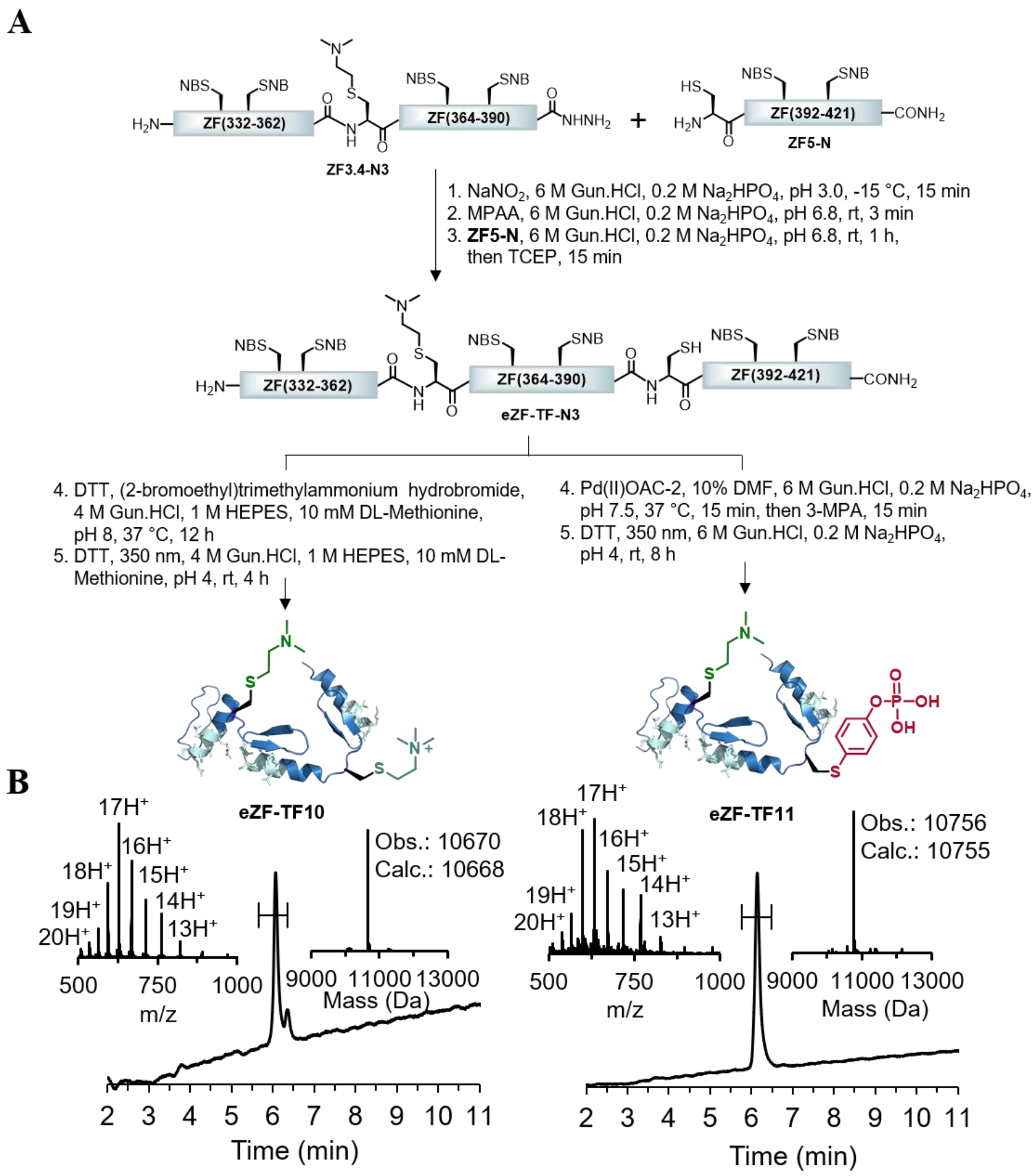

Supplementary Fig. 69 A Schematic representation of ligation and site-selective Cys modification. **B** LC-MS analysis of isolated **eZF-TF10** and **eZF-TF11**.

9. SDS-PAGE analysis

The analysis was performed using Bolt™ 4-12% Bis-Tris Gels (10-wells) at 165 V for 45 min utilizing pre-stained Invitrogen SeeBlue™ Plus2 molecular weight (MW) standard with Bolt™ LDS sample buffer (4X). **eZF-TFwt** and **eZF-TF1-5** analogs were mixed with 5% 2-mercaptoethanol (BME) and LDS sample buffer, followed by incubation at 90 °C for 5 min. ~ 0.5 μg of **eZF-TFwt** and **eZF-TF1-5** analogs were loaded onto the gel. The bands were visualized by Coomassie blue staining.

A

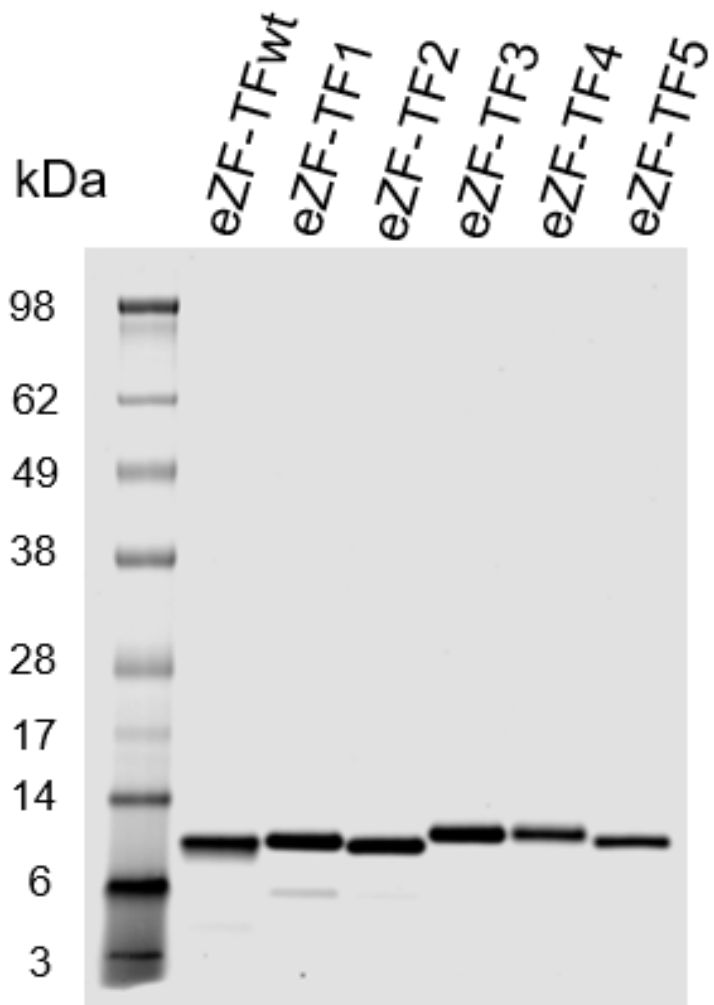

B

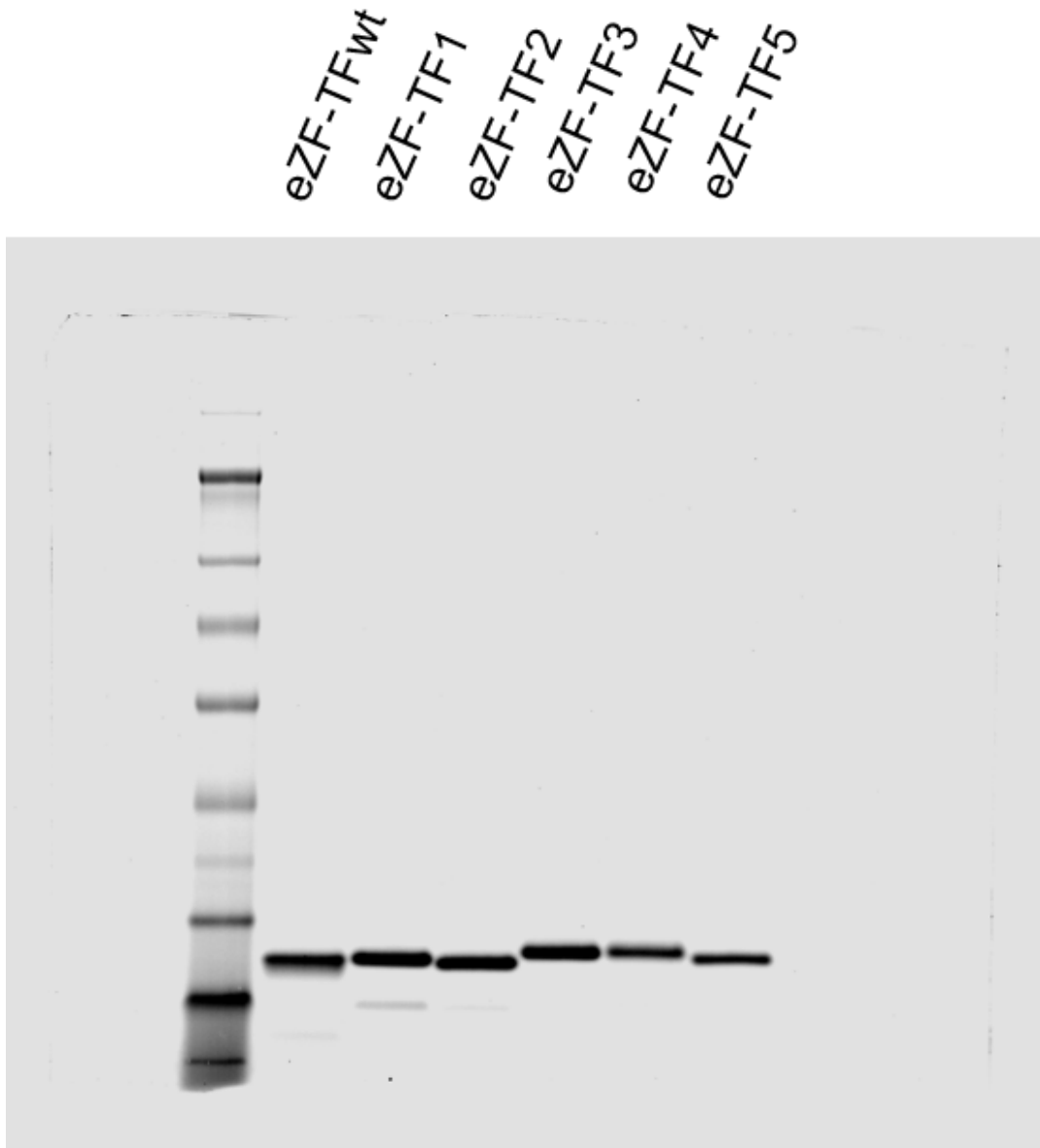

Supplementary Fig. 70 A SDS-PAGE analysis of **eZF-TFwt** and **eZF-TF1-5** analogs. **B** Uncropped gel scan for SDS-PAGE analysis of **eZF-TFwt** and **eZF-TF1-5** analogs.

10. Circular Dichroism (CD) Analysis

CD analysis was performed using a Chirascan circular dichroism spectrometer with a 0.1 mm path-length quartz cuvette. **eZF-TF** variants were prepared at a concentration of 20 µM in 50.0 µL of 10 mM Tris.HCl, 50 mM NaCl, 100 mM $ZnCl_2$, 1 mM TCEP, 5% glycerol buffer (pH 7.3). CD spectra of all samples were recorded in triplicate at 20 °C from 190 nm to 260 nm in a 1.0 nm step with a 3.0 nm slit bandwidth and an averaging time of three seconds per wavelength. The triplicate measurements were obtained from the same sample.

11. Electrophoretic Mobility-Shift Assay (EMSA)

EMSA analysis of eZF-TF1-5 analogs

To a 0.2 mL Eppendorf tube, 36.0 µL of 10 mM Tris.HCl, 50 mM NaCl, 75 µM $ZnCl_2$, 1 mM DTT, 5% glycerol buffer pH=7.3 (folding buffer) were added (except for the well containing only DNA, in this case contained 38.0 µL of the folding buffer). E-box DNA probe (2.0 µL, 10.0 µM; 1 equiv.) as a solution in the folding buffer and **eZF-TFwt**, **eZF-TF1-5** analogs (2.0 µL, 40.0 µM; 4 equiv.) as a solution in the folding buffer were added. The final incubation concentration of the major components was the following: DNA (0.5 µM) and protein (2.0 µM) in the folding buffer. The Eppendorf tube was closed, mixed, and incubated at rt for 30 min. Next, the DNA-binding activity of each analog was analyzed by EMSA: 5.0 µL of each DNA-protein mixture were mixed with 1.0 µL DNA Loading Dye (6X). 5.0 µL of each mixture was loaded onto a 10% TBE gel, 1.0 mm x 10 well and ran in 1xTBE at 90V for 45 min. The gel was washed three times with water for 30 seconds and then stained using Ethidium Bromide in 1xTBE buffer for 15 min at rt. Bands were visualized on A2S Vilber Fusion FX imager. EMSA analysis of **eZF-TFwt** and **eZF-TF1-5** analogs was performed in duplicate.

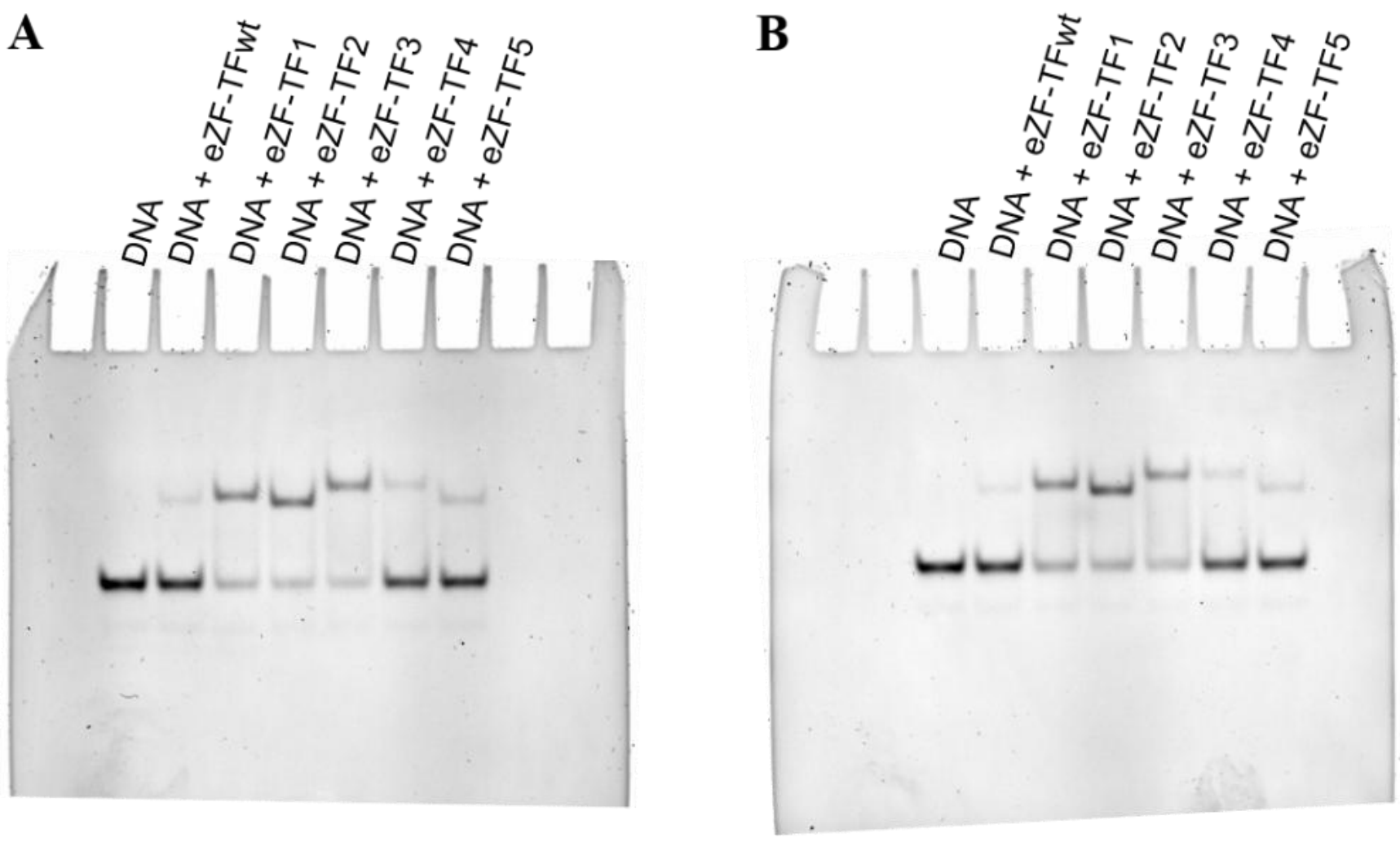

Supplementary Fig. 71 A Uncropped gel scan for EMSA analysis of **eZF-TFwt** and **eZF-TF1-5** analogs. **B.** Duplicate of the experiment. The experiments were performed from distinct samples.

12. Octet BioLayer Interferometry Binding Assay (BLI)

Biolayer interferometry (BLI) assay was performed using an Octet Red R4 System (ForteBio; Menlo Park, CA) in 96 well plates. Streptavidin Octet biosensors (ForteBio; Menlo Park, CA) were dipped into 200 µL of 0.1% BSA, 0.02% Tween-20, 1x PBS (kinetic buffer) for 10 min and then 60 more seconds in the kinetic buffer to obtain the baseline. Then the sensors were dipped into 200 µL of 65 nM biotinylated E-box DNA probe in the kinetic buffer for the loading step (300 sec). Sensors were then dipped into the kinetic buffer for 120 sec. Next, the tips were loaded with folded **eZF-TF** analog prepared in kinetic buffer at the indicated concentrations for 210 seconds to obtain the association curve. Then the tips were dipped into the kinetic buffer for 150 seconds to obtain the dissociation curve. Measurements were carried out at 25 °C. Data were analyzed within the ForteBio Octet Data Analysis software. The association and dissociation curves were fitted with Fortebio Octet Biosystems with a 1:1 Binding Model (global fitting algorithm) to obtain the K_D.

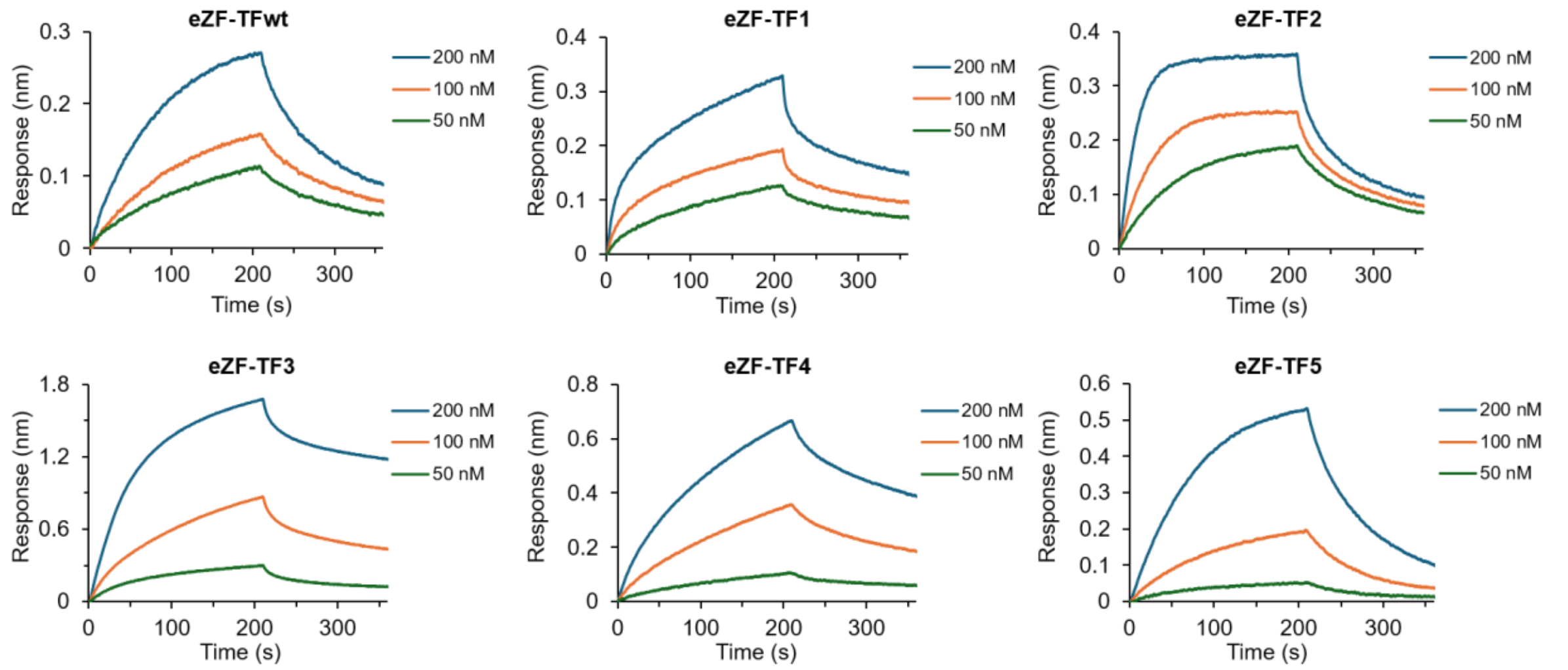

Supplementary Fig. 72 BLI analysis of **eZF-TF** analogs with the E-box DNA probe. The association and dissociation curves of proteins **eZF-TFwt** and **eZF-TF1-5** analogs with the E-box DNA probe.

13. Supplementary References

1. Zheng, J.-S., Tang, S., Qi, Y.-K., Wang, Z.-P. & Liu, L. Chemical synthesis of proteins using peptide hydrazides as thioester surrogates. *Nat. Protoc.* **8**, 2483–2495 (2013).

2. Bang, D. & Kent, S. B. H. A One-Pot Total Synthesis of Crambin. *Angew. Chem. Int. Ed.* **43**, 2534–2538 (2004).

3. Dawson, P. E., Muir, T. W., Clark-Lewis, I. & Kent, S. B. H. Synthesis of Proteins by Native Chemical Ligation. *Science (1979).* **266**, 776–779 (1994).

4. Fang, G. *et al.* Protein Chemical Synthesis by Ligation of Peptide Hydrazides. *Angew. Chem. Int. Ed.* **50**, 7645–7649 (2011).

5. Vinogradova, E. V., Zhang, C., Spokoyny, A. M., Pentelute, B. L. & Buchwald, S. L. Organometallic palladium reagents for cysteine bioconjugation. *Nature* **526**, 687–691 (2015).

6. Lin, X. *et al.* A Versatile Method for Site-Specific Chemical Installation of Aromatic Posttranslational Modification Analogs into Proteins. *J. Am. Chem. Soc.* **146**, 25788–25798 (2024).

7. Simon, M. D. *et al.* The Site-Specific Installation of Methyl-Lysine Analogs into Recombinant Histones. *Cell* **128**, 1003–1012 (2007).

8. Chalker, J. M. *et al.* Methods for converting cysteine to dehydroalanine on peptides and proteins. *Chem. Sci.* **2**, 1666 (2011).

9. Chalker, J. M., Lercher, L., Rose, N. R., Schofield, C. J. & Davis, B. G. Conversion of Cysteine into Dehydroalanine Enables Access to Synthetic Histones Bearing Diverse Post-Translational Modifications. *Angew. Chem. Int. Ed.* **51**, 1835–1839 (2012).

10. Mandal, S. *et al.* A Site-Specific Organometallic Approach for Installing Tyrosine Phosphorylation Mimics to Decipher the Role of Phosphorylation in Alpha-Synuclein (aSyn) Aggregation and Seeding. *J. Am. Chem. Soc.* **148**, doi.org/10.1021/jacs.6c12345 (2026).

11. Lin, X., Nithun, R. V., Samanta, R., Harel, O. & Jbara, M. Enabling Peptide Ligation at Aromatic Junction Mimics via Native Chemical Ligation and Palladium-Mediated S-Arylation. *Org. Lett.* **25**, 4715–4719 (2023).
