# Supplementary material for: Site-specific protein functionalization by late-stage reversible cysteine masking
Source: Nat Commun. 2026 Sep 10;17:10669. doi: 10.1038/s41467-026-77718-3 (PMC13655035; doi:10.1038/s41467-026-77718-3)
Supplement: Supplementary file 3 [file 41467_2026_77718_MOESM3_ESM.pdf]

nature portfolio

Peer Review File

Site-Specific Protein Functionalization by Late-Stage Reversible Cysteine Masking

Corresponding Author: Professor Muhammad Jbara

This file contains all reviewer reports in order by version, followed by all author rebuttals in order by version.

Version 0:

Reviewer comments:

Reviewer #1

(Remarks to the Author)
Authors report a reversible cysteine masking strategy for site-selective functionalization of cysteine-rich peptides and proteins. The authors employ temporary photolabile protection of native cysteine residues, followed by native chemical ligation, selective modification of a newly exposed cysteine residue, and subsequent decaging to furnish site-specifically modified products. The methodology is applied to model peptides, zinc finger proteins, and engineered zinc finger transcription factors.
The chemistry is presented here is well executed and addresses an important challenge in site selective protein modification.
However, I have several concerns regarding the conceptual framing and the scope of the methodology before I think this work is suitable for publication.

Major Issues:
1. The manuscript is presented as a solution to the challenge of site-selective protein functionalization. However, the selectivity achieved here arises from synthetic reconstruction of the protein sequence rather than selective modification of an intact protein containing multiple competing Cys sites. The strategy effectively creates a protein containing a single accessible cysteine rather than selectively differentiating among multiple native cysteines within an intact protein. I believe the manuscript needs to be reframed, otherwise it risks misleading the readers.
2. Native chemical ligation and protein semisynthesis have long enabled site-specific incorporation of post-translational modifications, unnatural amino acids, and synthetic probes. The manuscript does not clearly establish what unique capabilities are enabled by this work relative to established semisynthetic strategies. Why would one choose this strategy instead of directly incorporating the desired modification within a synthetic peptide fragment prior to ligation? A discussion of the advantages and limitations of this strategy relative to conventional semisynthesis is needed.
3. From the data provides, structural integrity of zinc finger proteins is not demonstrated. This should be demonstrated at least by one spectroscopic technique (eg., at minimum, the authors should provide a circular dichroism spectrum of the conjugate vs. unmodified zinc finger.)
Minor Comments
1. Writing quality needs to be elevated in several parts of the manuscript, especially in the introduction (see above).
2. More state of the field general citations needed to back up claims, especially in the introduction (see above).
3. Figures need better visual clarity. While many of the graphics showcased throughout the manuscript are of high quality, their layouts especially in figures 1 and 2 are difficult to follow. It would be helpful if (a,b,c,etc) could be added for clarity.

Reviewer #2

(Remarks to the Author)
Singh et al. present a synthetic strategy for accessing site-selectively modified proteins by combining a photolabile protecting-group approach with late-stage cysteine bioconjugation. The authors assemble small proteins via native chemical ligation of peptide fragments in which all cysteines except the N-terminal ligation site are masked with the 2-nitrobenzyl (oNB) group. Therefore, only the cysteine at the ligation site is available for downstream modification using established cysteine-targeting chemistries. After the labeling step, UV-mediated deprotection restores the remaining cysteines, yielding site-selectively modified proteins.
The manuscript is carefully executed and presents an impressive scope of modified proteins alongside thorough

characterization of the synthesized constructs. However, the work represents a thoughtful combination of existing technologies rather than a fundamental methodological advance.
While the quality and execution of the work are not in question, the manuscript in its current form may be better suited to a more specialized journal such as Communications Chemistry.

General Comments:
- The o-nitrobenzyl (oNB) group is a well-established temporary protecting group for cysteine in peptide and protein chemistry (Smith et al., Org. Lett. 2002; Karas et al., Chem. Eur. J. 2014; Laps et al., ACIE 2021; Xie et al., Org. Lett. 2023) and has previously been applied to the site-selective modification of intact proteins (Osgood et al., Bioconjugate Chem. 2024) and does not represent a methodological advance presented in this manuscript.

- The claim of single-site protein modification requires qualification. In the described late-stage modification of eZF-TF, the chosen synthetic strategy introduces two modifications simultaneously. While this still represents a high degree of selectivity, it shows that larger proteins requiring multisegment ligation remain a notable limitation of this approach.

Specific Questions:
- The authors use a tedious procedure, where isolated peptides are modified with o-nitro-benzyl-bromide. Why did the authors not employ an on-resin modification strategy, this would allow for increased throughput with less time-intensive purification steps.

- In Figure 5E the authors impressively demonstrate that late-stage modification of eZF-TF gives access to transcription factors with enhanced binding affinity towards the E-box DNA sequence. Beyond the general speculation on sterics and salt-bridge/hydrogen-bond interactions, have the authors attempted to obtain data that rationalizes this observation? Computational or structural approaches such as molecular docking, X-ray crystallography, or NMR might be informative in this regard.

Version 1:

Reviewer comments:

Reviewer #1

(Remarks to the Author)
Authors have revised the manuscript per my comments, publication is now recommended.

Reviewer #2

(Remarks to the Author)
In the revised version of the manuscript, Singh et al. present a more detailed characterization of the synthesized protein constructs, verifying overall structural integrity. Additionally, the authors demonstrate that their synthetic strategy can be adapted to synthesize cysteine-rich proteins bearing two distinct site-selective modifications.
While this amounts to an impressive 30 synthetically accessed and (more or less) site-selectively modified zinc finger domains, they all correspond to a single protein, Zif268, and no other example is provided. The authors also suggest that this strategy could be applied to expressed protein ligation; however, no such example is included.

Altogether, I maintain my previous assessment. In my opinion, the work presented is of high quality. However, I am not sure if the data provided justifies publication in Nature Communications, given that broader generalizability remains undemonstrated. Therefore, I leave the final decision to the editor.

Open Access This Peer Review File is licensed under a Creative Commons Attribution 4.0 International License, which permits use, sharing, adaptation, distribution and reproduction in any medium or format, as long as you give appropriate credit to the original author(s) and the source, provide a link to the Creative Commons license, and indicate if changes were made.
In cases where reviewers are anonymous, credit should be given to 'Anonymous Referee' and the source.
The images or other third party material in this Peer Review File are included in the article's Creative Commons license, unless indicated otherwise in a credit line to the material. If material is not included in the article's Creative Commons license and your intended use is not permitted by statutory regulation or exceeds the permitted use, you will need to obtain permission directly from the copyright holder.
To view a copy of this license, visit https://creativecommons.org/licenses/by/4.0/

Point-by-point response to the reviewers

Reviewer #1 (Remarks to the Author):

Authors report a reversible cysteine masking strategy for site-selective functionalization of cysteine-rich peptides and proteins. The authors employ temporary photolabile protection of native cysteine residues, followed by native chemical ligation, selective modification of a newly exposed cysteine residue, and subsequent decaging to furnish site-specifically modified products. The methodology is applied to model peptides, zinc finger proteins, and engineered zinc finger transcription factors.

The chemistry is presented here is well executed and addresses an important challenge in site selective protein modification.

However, I have several concerns regarding the conceptual framing and the scope of the methodology before I think this work is suitable for publication.

We sincerely thank the referee for the positive evaluation of our manuscript and for recommending its publication after addressing the requested revisions. We greatly appreciate the constructive comments and the careful consideration given to our work throughout the review process. In response to these valuable suggestions, we have substantially revised the manuscript. Specifically, we performed additional experiments, including circular dichroism (CD) analyses, biolayer interferometry (BLI) measurements, and expanded the ReCag approach to generate new advanced analogs bearing orthogonally introduced distinct modifications. We also revised the Abstract, Introduction, Results, and Discussion sections to address the referee's comments and to better emphasize the significance and broader implications of our findings. We believe that these revisions have strengthened the manuscript and improved the clarity, and interpretation of our results.

Major Issues:

1. The manuscript is presented as a solution to the challenge of site-selective protein functionalization. However, the selectivity achieved here arises from synthetic reconstruction of the protein sequence rather than selective modification of an intact protein containing multiple competing Cys sites. The strategy effectively creates a protein containing a single accessible cysteine rather than selectively differentiating among multiple native cysteines within an intact protein. I believe the manuscript needs to be reframed, otherwise it risks misleading the readers.

We thank the reviewer for this insightful observation. To better define the scope of our work, we have revised the manuscript, including the abstract, introduction, and discussion, to clearly distinguish our methodology from direct intact protein bioconjugation strategies. We now emphasize that our approach enables late-stage site-selective functionalization

through synthetic reconstruction of the protein sequence using a temporary Cys masking process. Throughout the manuscript, we now emphasize that Cys masking, combined with native chemical ligation, enables the generation of a common synthetic intermediate containing predefined reactive Cys residue(s) that can subsequently undergo a broad range of Cys-based transformations. Finally, we now also discuss the current limitations of the methodology and state that the development of approaches capable of directly differentiating among multiple native Cys residues in intact proteins represents an important future direction (MS, pages 3-4 and 11-12). We believe these revisions more accurately position the conceptual advance of the work while clearly defining its scope and applicability.

2. Native chemical ligation and protein semisynthesis have long enabled site-specific incorporation of post-translational modifications, unnatural amino acids, and synthetic probes. The manuscript does not clearly establish what unique capabilities are enabled by this work relative to established semisynthetic strategies. Why would one choose this strategy instead of directly incorporating the desired modification within a synthetic peptide fragment prior to ligation? A discussion of the advantages and limitations of this strategy relative to conventional semisynthesis is needed.

Thank you for pointing this out. While pre-installation of modifications into synthetic peptide segments followed by native chemical ligation is a well-established approach for generating modified proteins, each new protein variant requires the independent synthesis, purification, and ligation of a new peptide segment. Consequently, introducing different modifications at the same site typically requires repeating the entire synthetic workflow, making the preparation of multiple protein variants both time-consuming and labor-intensive. In contrast, our approach generates a common synthetic intermediate containing a selectively reactive Cys residue that can subsequently be diversified through a broad range of transformations. This modular workflow enables rapid late-stage diversification from the same peptide building blocks, substantially simplifying the preparation of libraries of site-specifically modified proteins while providing greater flexibility for protein engineering, probe development, and structure-activity relationship studies.
To better highlight these advantages, we have expanded the introduction and discussion to clarify the advantages of our strategy relative to conventional ligation-based approaches (MS, pages 3-4 and 11-12). We also discuss the current limitations of the methodology. While our approach enables the installation of diverse chemical modifications and probes into predefined Cys residues, it relies on temporary Cys masking and subsequent decaging to achieve this selectivity. We have now highlighted that an important direction for future research is the development of approaches that enable site-selective Cys functionalization in the presence of multiple native Cys residues in intact proteins (MS, pages 11–12).

3. From the data provides, structural integrity of zinc finger proteins is not demonstrated. This should be demonstrated at least by one spectroscopic technique (eg., at minimum, the authors should provide a circular dichroism spectrum of the conjugate vs. unmodified zinc finger.)

Thank you for your comment. We have now performed CD analysis on engineered zinc finger analogs to evaluate their structural integrity. As shown in the revised manuscript (Figure 5d), all analogs exhibited the characteristic CD signature of a properly folded zinc-finger protein containing Cys2-His2-type zinc-finger domains, with the expected double minima at 208 and 222 nm, confirming that the overall synthetic eZF-TF fold is preserved. Notably, analogs eZF-TF1-3 displayed increased structural stability, as evidenced by enhanced CD signal intensity, in agreement with their improved DNA-binding activity observed by EMSA. In contrast, analogs eZF-TF4 and eZF-TF5 exhibited a minor effect on the zinc-finger fold upon incorporation of the methyl-lysine and phosphotyrosine analogs. These observations correlate well with the DNA-binding activity observed in the EMSA. These new data have been incorporated into Figure 5d and are discussed in the revised manuscript (MS, page 9). Finally, to further investigate the impact of the different modifications on ZF–DNA interactions, we performed additional BLI experiments to determine the DNA-binding affinities of all analogs. The results revealed enhanced DNA-binding affinity for analogs eZF-TF1-3 (K_D= 54.4 nM, 54.6 nM, and 39.3 nM were determined for eZF-TF1–3, respectively), whereas analogs eZF-TF4-5 exhibited a clear reduction in binding affinity (K_D= 121 nM and 3.56 μM were determined for eZF-TF4-5, respectively). These new data have been incorporated into Figure 5f and are discussed in the revised manuscript (MS, page 10).

Minor Comments

1. Writing quality needs to be elevated in several parts of the manuscript, especially in the introduction (see above).
2. More state of the field general citations needed to back up claims, especially in the introduction (see above).

We thank the reviewer for this valuable suggestion. The introduction, results, and discussion sections have been revised to improve clarity and readability. In addition, we have incorporated several recent references covering advances in protein synthesis and site-selective protein functionalization as requested by the reviewer (references 16-28, 66-67).

3. Figures need better visual clarity. While many of the graphics showcased throughout the manuscript are of high quality, their layouts especially in figures 1 and 2 are difficult to follow. It would be helpful if (a,b,c,etc) could be added for clarity.

Thank you for pointing this out. We have labeled each panel in the relevant figures to further improve clarity.

Reviewer #2 (Remarks to the Author):

Singh et al. present a synthetic strategy for accessing site-selectively modified proteins by combining a photolabile protecting-group approach with late-stage cysteine bioconjugation. The authors assemble small proteins via native chemical ligation of peptide fragments in which all cysteines except the N-terminal ligation site are masked with the 2-nitrobenzyl (oNB) group. Therefore, only the cysteine at the ligation site is available for downstream modification using established cysteine-targeting chemistries. After the labeling step, UV-mediated deprotection restores the remaining cysteines, yielding site-selectively modified proteins.

The manuscript is carefully executed and presents an impressive scope of modified proteins alongside thorough characterization of the synthesized constructs. However, the work represents a thoughtful combination of existing technologies rather than a fundamental methodological advance.

While the quality and execution of the work are not in question, the manuscript in its current form may be better suited to a more specialized journal such as Communications Chemistry.

We sincerely thank the referee for the positive evaluation of our manuscript. We greatly appreciate the constructive comments and the careful consideration given to our work throughout the review process. In response to these valuable suggestions, we have substantially revised the manuscript. Specifically, we performed additional experiments, including circular dichroism (CD) analyses, biolayer interferometry (BLI) measurements, and expanded the ReCag approach to generate new advanced analogs bearing orthogonally introduced distinct modifications. We also revised the results and discussion sections to address the referee's comments and to better emphasize the significance and broader implications of our findings. We believe that these revisions have strengthened the manuscript and improved the clarity and interpretation of our results.

In this work, our primary goal was to develop a rapid and versatile strategy for the site-selective functionalization of Cys-rich proteins with high precision. Although our approach employs state-of-the-art transformations, e.g., NCL, the developed ReCag strategy enables the generation of novel site-specifically modified proteins that would be difficult to achieve using conventional synthetic approaches. For instance, **while pre-installation of modifications into synthetic peptide segments followed by NCL is a well-established approach for generating site-specifically modified proteins, each new protein variant requires the independent synthesis, purification, and ligation of a new peptide segment. Consequently, introducing different modifications at the same site typically requires repeating the entire synthetic workflow, making the preparation of multiple protein variants both time-consuming and labor-intensive. In contrast, our approach generates a common synthetic intermediate containing a selectively reactive Cys residue that can subsequently be diversified through a broad range of Cys-based transformations. This modular workflow**

enables rapid late-stage diversification from the same peptide building blocks, substantially simplifying the preparation of libraries of site-specifically modified proteins while providing greater flexibility for protein engineering, probe development, and structure-activity relationship studies.

Key advantages of the ReCag approach:

- **Modular late-stage diversification. Starting from the same peptide segments, our strategy generates a common synthetic intermediate that can be diversified into a library of site-specifically modified proteins, eliminating the need to repeatedly synthesize, purify, and ligate multiple peptide variants.** ***Remarkably, in this work, we were able to generate more than 30 site-specifically modified analogs from the same 2-3 peptide segments.***
- **Broad compatibility with diverse chemistries. The developed approach enables a wide range of selective Cys-based transformations, including alkylation, arylation, and elimination reactions, enabling rapid functionalization of proteins for the preparation of diverse protein libraries and providing a versatile platform for applications in protein engineering and structure-activity relationship studies.**
- **Efficient late-stage diversification of complex transcription factor proteins. ReCag enables efficient, site-selective late-stage diversification of Cys-rich proteins, providing a powerful platform for probing the structural diversity of key domains that govern protein–DNA interactions. This approach facilitates the discovery of potent engineered zinc finger transcription factor (eZF-TF) analogs with enhanced DNA binding activity.**
- **Potential compatibility with recombinant protein segments. Because the reversible Cys masking is performed in solution, the strategy can potentially be extended to recombinantly expressed peptide fragments. Combined with native or expressed protein ligation, this strategy could significantly expand the applicability of late-stage protein functionalization to larger and more complex protein targets.**

To better highlight these advantages, we have expanded the introduction and discussion to clarify the unique capabilities of our strategy relative to conventional ligation-based synthetic approaches. We also explicitly discuss the current limitations of the methodology. (MS, pages 3-4 and 11-12)

General Comments:
- The o-nitrobenzyl (oNB) group is a well-established temporary protecting group for cysteine in peptide and protein chemistry (Smith et al., Org. Lett. 2002; Karas et al., Chem. Eur. J. 2014; Laps et al., ACIE 2021; Xie et al., Org. Lett. 2023) and has previously been applied to the site-selective modification of intact proteins (Osgood et al., Bioconjugate

Chem. 2024) and does not represent a methodological advance presented in this manuscript.

Thank you for this comment. Our work introduces the strategic integration of temporary Cys masking with protein ligation to generate a versatile intermediate for the late-stage diversification of Cys-rich proteins via a broad range of transformations. This approach provides a versatile and orthogonal platform for the site-selective functionalization of proteins with a wide variety of chemical transformations and molecular probes. We have revised the manuscript to more clearly emphasize this conceptual advance and the unique capabilities of the ReCag platform. Regarding the previous use of the o-nitrobenzyl (oNB) group, we agree that oNB is a well-established photolabile protecting group for Cys and has been employed in a variety of applications. We have incorporated additional relevant references into the revised manuscript (references 33-35). Notably, previous applications of oNB have been mostly used for directing the formation of disulfide bonds in polypeptides and have rarely been applied to late-stage protein functionalization and editing. To the best of our knowledge, late-stage reversible Cys masking has not previously been employed to control protein ligation and enable site-selective chemical transformations for the generation of modified native Cys-rich proteins. This modular strategy enables the rapid preparation of libraries of site-specifically modified proteins from the same peptide building blocks, providing capabilities that are difficult to achieve using conventional synthetic approaches.

- The claim of single-site protein modification requires qualification. In the described late-stage modification of eZF-TF, the chosen synthetic strategy introduces two modifications simultaneously. While this still represents a high degree of selectivity, it shows that larger proteins requiring multisegment ligation remain a notable limitation of this approach.

We thank the reviewer for this important comment. Our methodology relies on two-segment native chemical ligation to generate a uniquely accessible Cys residue for site-selective functionalization. We agree that, for proteins assembled by multisegmented ligation, more than one transiently exposed Cys residue may be generated, necessitating the controlled installation of multiple modifications. To further demonstrate and expand the versatility of the ReCag approach for the preparation of more complex analogs bearing multiple site-specific modifications, we have performed additional experiments showing that a diverse range of distinct transformations, including probes, amino acid residues, and post-translational modification mimics, can be introduced in a late-stage manner using the ReCag strategy. Specifically, starting from three peptide segments, we generated six new protein analogs containing two distinct modifications using a shared synthetic intermediate. These new results have been incorporated into the revised manuscript (MS, page 10) and are presented in the new Figure 6. Finally, we have expanded the results and discussion to emphasize that although multisegmented assembly may require the simultaneous

modification of multiple ligation-derived Cys, the methodology remains highly modular and provides an efficient route to access site-selectively functionalized proteins. We further note in the manuscript that future implementation in combination with expressed protein ligation should enable the extension of this strategy to larger protein targets assembled from two segments while retaining the advantages of late-stage diversification.

Specific Questions:

- The authors use a tedious procedure, where isolated peptides are modified with o-nitro-benzyl-bromide. Why did the authors not employ an on-resin modification strategy, this would allow for increased throughput with less time-intensive purification steps.

We thank the reviewer for this valuable suggestion. We agree that on-resin installation of the oNB group represents an attractive alternative for the preparation of masked synthetic peptides. However, we developed the late-stage oNB installation in solution because it offers significantly broader applicability beyond solid-phase synthesis and synthetic peptides. Importantly, solution-phase masking has the potential to be extended to peptide segments obtained by recombinant expression, thereby enabling selective masking of recombinantly produced protein fragments prior to semisynthetic assembly. We believe this flexibility can substantially expand the scope of the methodology beyond purely synthetic peptides and provides a general strategy for the late-stage diversification of semisynthetic proteins. We have highlighted in the revised discussion the key advantages of the developed solution-phase late-stage masking strategy, which offers significantly broader applicability. In particular, it could be leveraged to selectively mask recombinantly expressed peptide or protein fragments, thereby extending this methodology beyond fully synthetic peptides and enabling the late-stage semisynthetic functionalization of more complex protein targets (MS, pages 11-12).

- In Figure 5E the authors impressively demonstrate that late-stage modification of eZF-TF gives access to transcription factors with enhanced binding affinity towards the E-box DNA sequence. Beyond the general speculation on sterics and salt-bridge/hydrogen-bond interactions, have the authors attempted to obtain data that rationalizes this observation? Computational or structural approaches such as molecular docking, X-ray crystallography, or NMR might be informative in this regard.

We thank the reviewer for this insightful suggestion. While additional structural studies could provide insights into the interactions between the engineered TF and DNA targets, the primary objective of this work was to establish a robust and general late-stage synthetic platform for the site-selective functionalization of Cys-rich peptides and proteins with a broad range of transformations. As a representative application of this methodology, we engineered a series of zinc finger transcription factors (eZF-TFs) and demonstrated that the resulting analogs can be efficiently diversified, leading to the discovery of new advanced

analogs. Additional structural investigations of these analogs require substantial additional investigations and are beyond the scope of the present work. However, to further investigate the impact of the introduced modifications on the structural integrity and function of the engineered eZF-TF, we have now performed additional circular dichroism (CD) and biolayer interferometry (BLI) analyses. The CD measurements confirmed that all analogs exhibited the characteristic CD signature of a properly folded zinc-finger protein containing Cys2-His2-type zinc-finger domains, with the expected double minima at 208 and 222 nm, confirming that the overall synthetic eZF-TF fold is preserved. Notably, eZF-TF1-3 analogs displayed increased structural stability, as evidenced by enhanced CD signal intensity, in agreement with their improved DNA-binding activity observed by EMSA. In contrast, eZF-TF4 and eZF-TF5 exhibited a minor effect on the zinc-finger fold upon incorporation of the methyl-lysine and phosphotyrosine analogs. These observations correlate well with the DNA-binding activity observed in the EMSA. These new data have been incorporated into Figure 5d and are discussed in the revised manuscript (MS, page 9). Finally, to further investigate the impact of the different modifications on ZF–DNA interactions, we performed additional BLI experiments to determine the DNA-binding affinities of all analogs. The results revealed enhanced DNA-binding affinity for analogs eZF-TF1-3 (K_D= 54.4 nM, 54.6 nM, and 39.3 nM were determined for eZF-TF1–3, respectively), whereas analogs eZF-TF4-5 exhibited a clear reduction in binding affinity (K_D= 121 nM and 3.56 μM, respectively). These findings are in good agreement with the EMSA results, further supporting our conclusion that the modifications introduced in analogs eZF-TF1–3 confer potent DNA-binding activity toward the E-box DNA.

EDITORIAL REQUESTS AND FORMATTING

We have addressed all editorial requests and implemented the recommended formatting changes throughout the revised manuscript.
